# Supplementary material for: Using single‐plant‐omics in the field to link maize genes to functions and phenotypes
Source: Mol Syst Biol. 2020 Dec 21;16(12):e9667. doi: 10.15252/msb.20209667 (PMC7751767; doi:10.15252/msb.20209667)
Supplement: Supplementary file 1 — Appendix [file MSB-16-e9667-s001.pdf]

APPENDIX

Table of contents

Appendix Figure S1. SNP analysis of individual plants.

Appendix Figure S2. Chromosomal locations of SNPs significantly associated with the genetic subpopulation structure.

Appendix Figure S3. Transcriptome, metabolome and phenotype PCA plots mapped to the field.

Appendix Figure S4. Normalized expression CV distributions in the single-plant dataset for diurnally varying genes versus non-diurnally varying genes.

Appendix Figure S5. Gene expression variability in maize single-plant dataset versus Arabidopsis single-plant dataset of Cortijo et al. (2019).

Appendix Figure S6. Proportion of variance in the LME model residuals explained by *i.i.d.* 'noise' and spatial covariance.

Appendix Figure S7. Average expression of spatially autocorrelated transcript clusters mapped to the field.

Appendix Figure S8. Average expression of spatially autocorrelated metabolite clusters mapped to the field.

Appendix Figure S9. Significant correlations of the average expression profiles of spatially autocorrelated transcript clusters with phenotypes.

Appendix Figure S10. Significant correlations of the average profiles of spatially autocorrelated metabolite clusters with phenotypes.

Appendix Figure S11. Metabolite level variability in the maize single-plant dataset.

Appendix Figure S12. Hierarchical clustering of the maize B73 leaf transcriptome datasets obtained from the SRA database.

Appendix Figure S13. Gene function prediction performance for coexpression networks of different size.

Appendix Figure S14. Gene function prediction performance plots for the GO categories listed in Dataset EV9.

Appendix Figure S15. Manhattan and Q-Q plots for GWAS on single-plant phenotype data.

Appendix Figure S16. Range estimates for each phenotype.

Appendix Table S1. Number of transcripts, metabolites and phenotypes with significant batch, DOH, SNP or SAC effects at $q \leq 0.01$.

Appendix Table S2. Genomic characteristics of highly variable genes and lowly variable genes in the single-plant transcriptome dataset.

Appendix Table S3. Top-10 novel regulators predicted to be involved in the response to chitin based on the single-plant data.

Appendix Table S4. Top-10 novel regulators predicted to be involved in the response to water deprivation based on the single-plant data.

Appendix Table S5. Top-10 novel regulators predicted to be involved in C_4 photosynthesis based on the single-plant data.

Appendix Table S6. Genetic variants identified in the maize single-plant RNA-seq dataset.

Appendix Table S7. Performance of SNP e-net and random forest models for trait prediction.

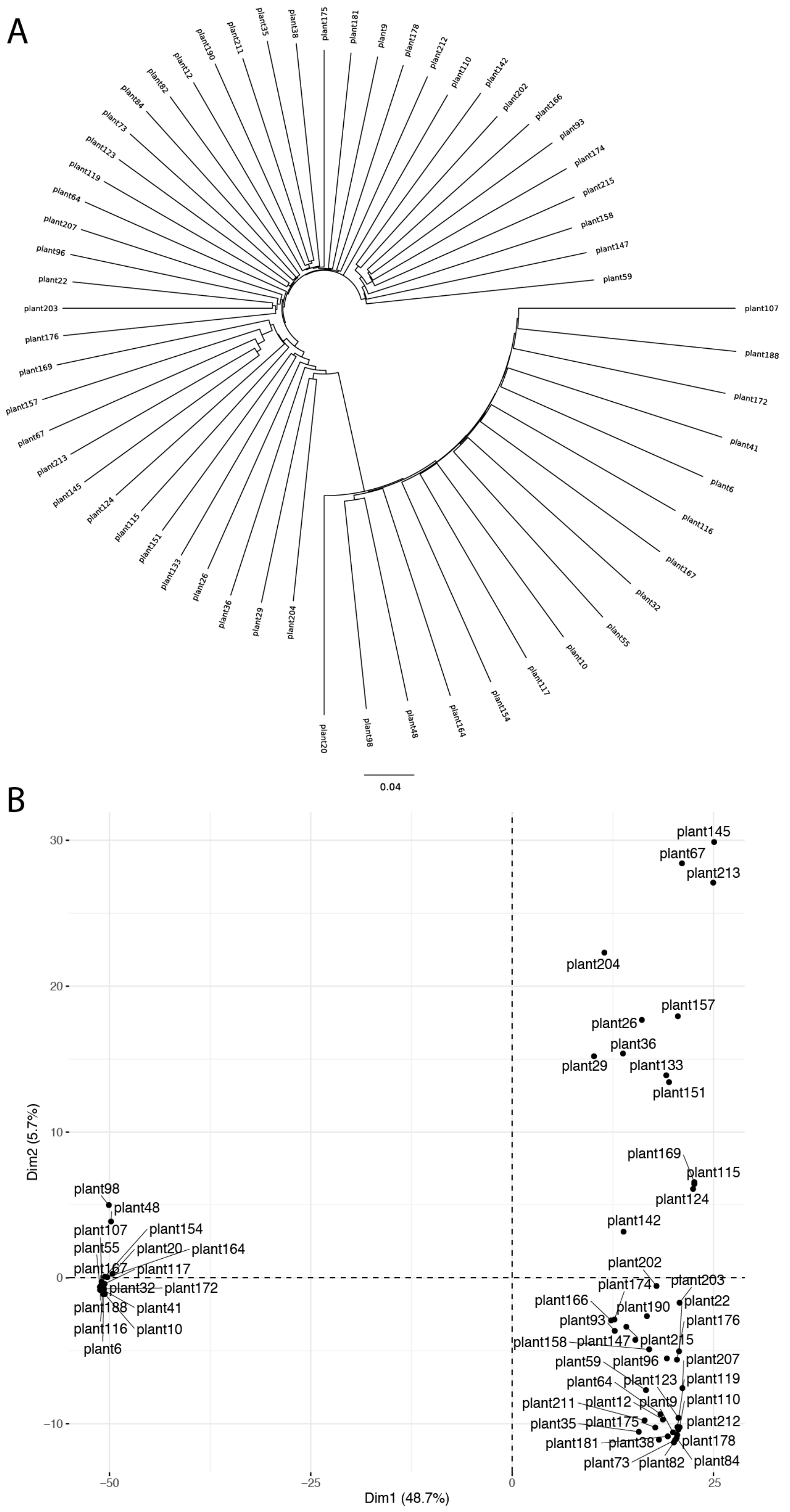

Appendix Figure S1. SNP analysis of individual plants. (A) Neighbor-joining tree for the individual plants based on biallelic SNPs in the RNA-seq data. Branch lengths are proportional to 1 - IBS (identity by state). (**B**) PCA plot for the individual plants based on their biallelic SNP profiles.

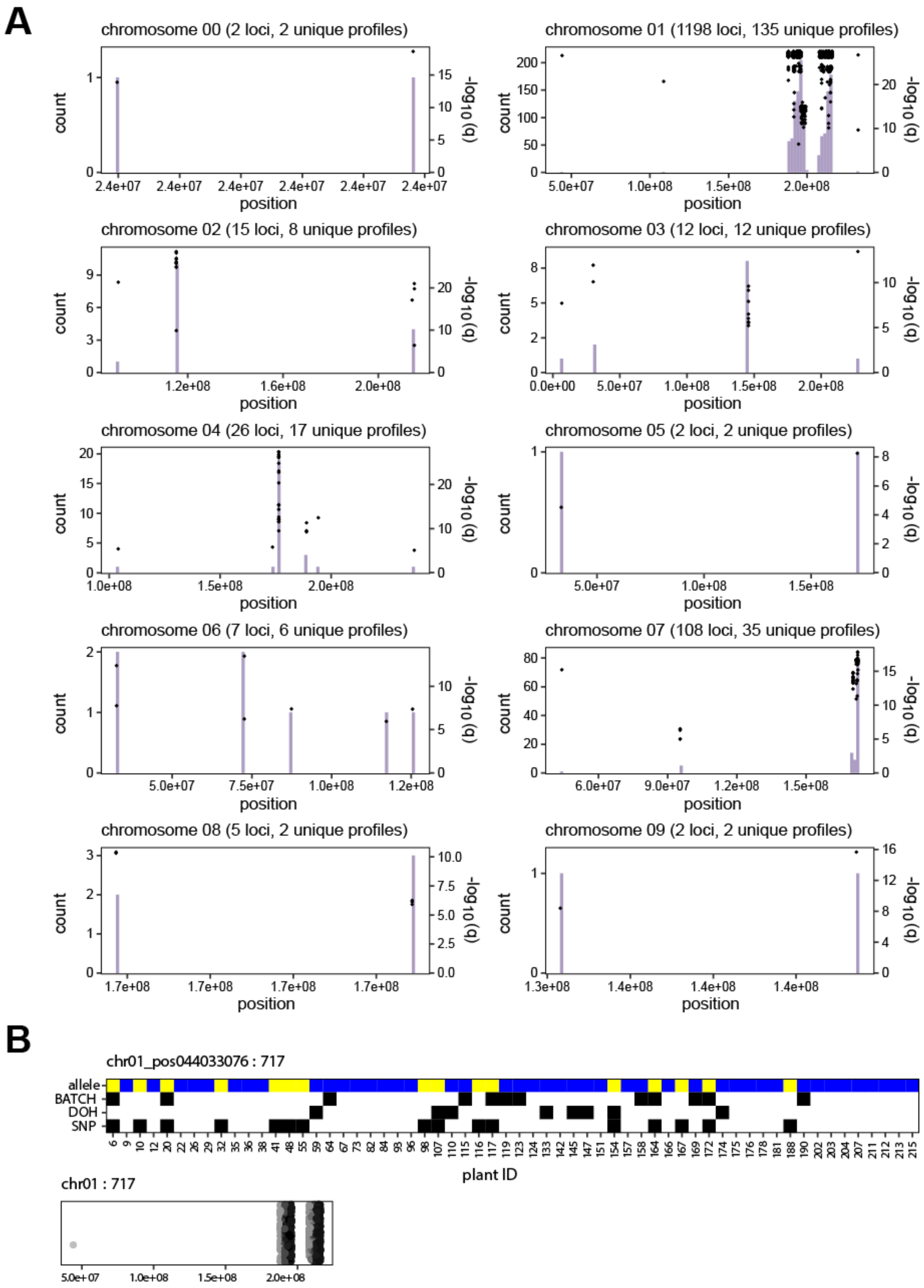

Appendix Figure S2. Chromosomal locations of SNPs significantly associated with the genetic subpopulation structure. See legend on next page.

Appendix Figure S2. Chromosomal locations of SNPs significantly associated with the genetic subpopulation structure. (A) Each subplot displays one chromosome, with positions in base pairs on the x-axis. SNPs that are significantly associated with the subpopulation structure are shown as black dots. A slight vertical jitter is added to help visualize the density of overlapping dots. Hypergeometric test *q*-values for the association of each SNP with subpopulation structure are indicated on the right y-axis (only approximately due to jitter). The grey histogram in each subplot displays the distribution of subpopulation-associated SNPs across the chromosome in 100 equal-sized bins (left y-axis). The total number of subpopulation structure-associated SNP loci on each chromosome is shown at the top of the subplots together with the number of unique SNP profiles across plants (some groups of SNPs share the same profile). **(B)** Example group of biallelic SNPs on chromosome 1 with exactly the same allele profile across plants. The top subplot displays the allele profile of the SNP group (top row) compared to the profiles of the sequencing batch (BATCH), day-of-harvest (DOH) and SNP subpopulation (SNP) variables. Blue, white or yellow squares in the allele profile indicate that the plant concerned is homozygous for the major allele, heterozygous or homozygous for the minor allele, respectively, for all SNPs in the group. The first locus (in numerical order) that fits the allele profile of the SNP group and the number of loci in the SNP group are shown above the plot. The bottom subplot in panel **(B)** shows the location on the genome (x-axis) for all loci in the SNP group. A random jitter was applied to the y-axis to visualize overlapping points, and point-dense regions are colored darker than sparse regions.

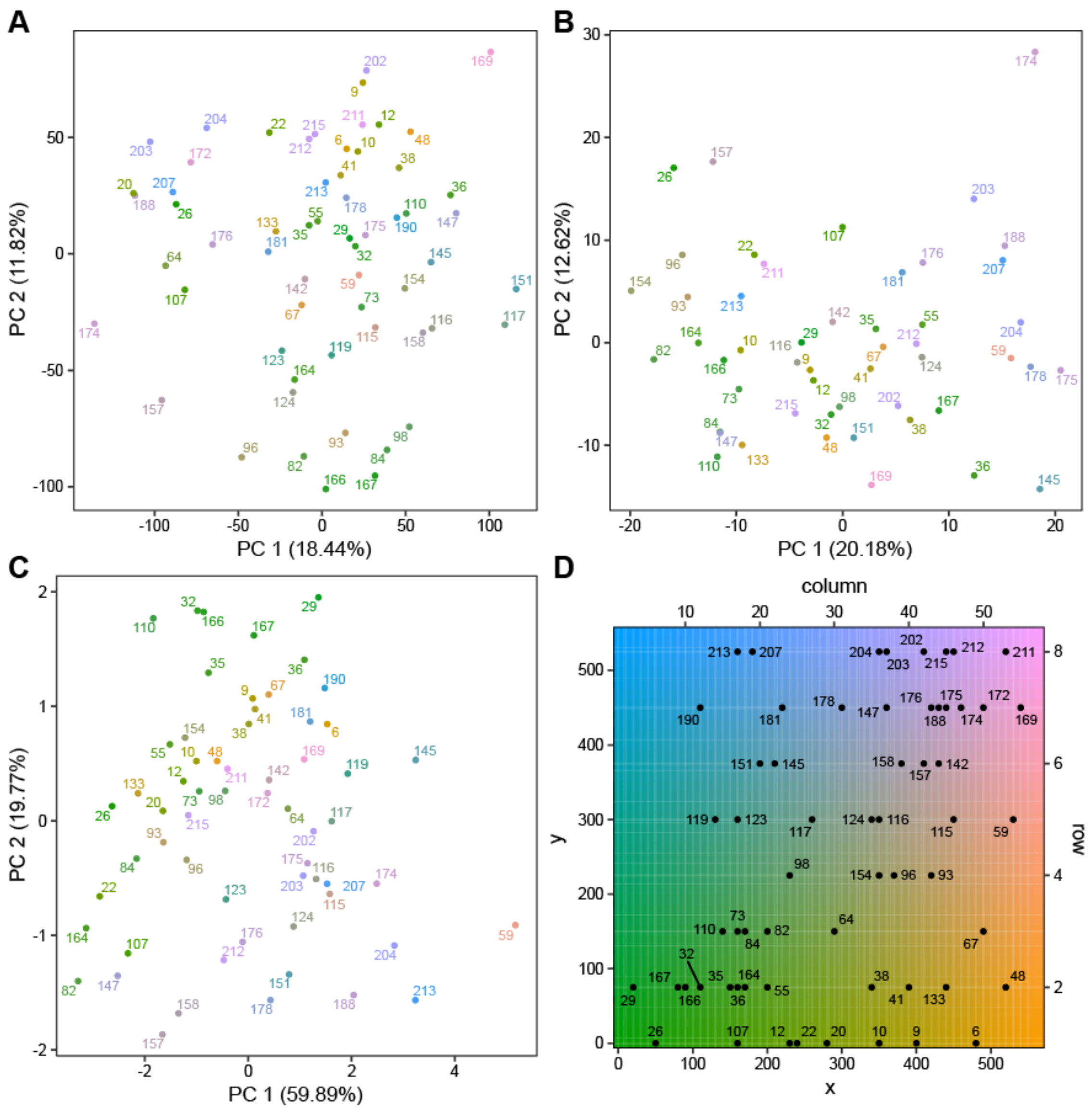

Appendix Figure S3. Transcriptome, metabolome and phenotype PCA plots mapped to the field. (A) Plot showing the first two principal components (PCs) in a PCA of the 60 single-plant transcriptomes. **(B)** Plot showing the first two PCs in a PCA of the 50 single-plant metabolomes. **(C)** Plot showing the first two PCs in a PCA of the plant phenomes for the 60 plants that were RNA-sequenced. The plants in panels **(A)-(C)** are colored according to their field position, as indicated in panel **(D)**. Dimensions in panel **(D)** are to scale, the distance between rows and between columns is 75 cm and 10 cm, respectively.

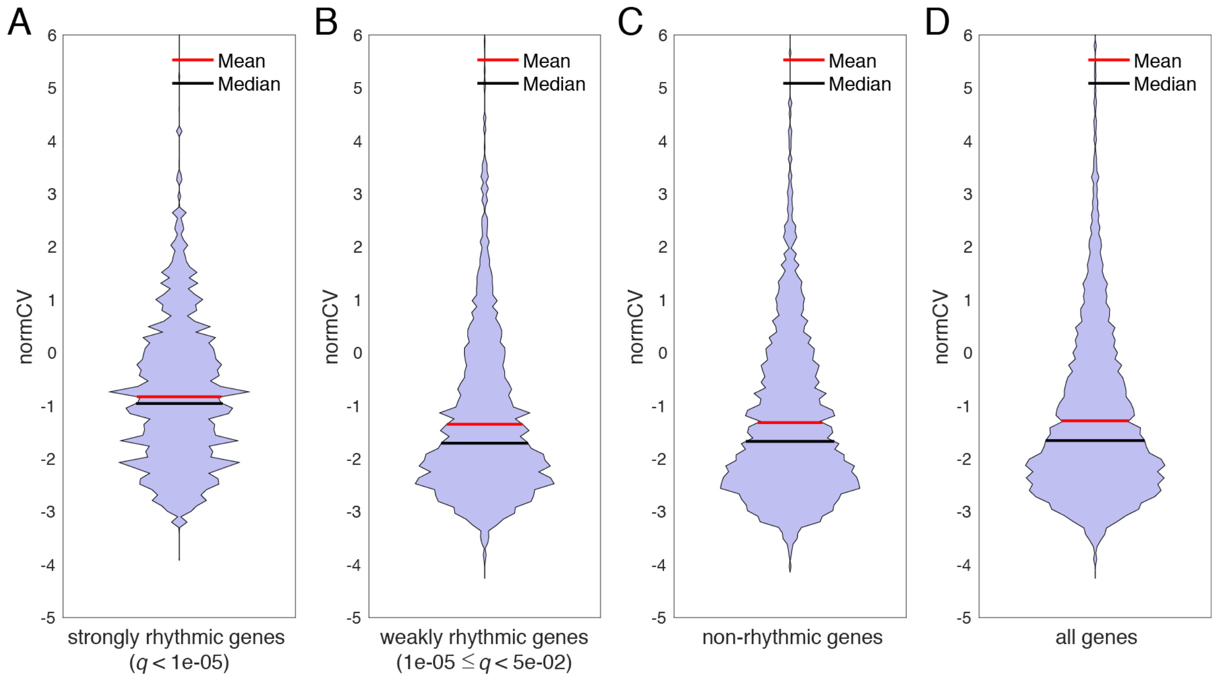

Appendix Figure S4. Normalized expression CV distributions in the single-plant dataset for diurnally varying genes versus non-diurnally varying genes. Violin plots of normalized CV distributions are shown for genes identified in Lai et al. (2020) as strongly rhythmic **(A)**, weakly rhythmic **(B)** or non-rhythmic **(C)**, and for all genes **(D)**.

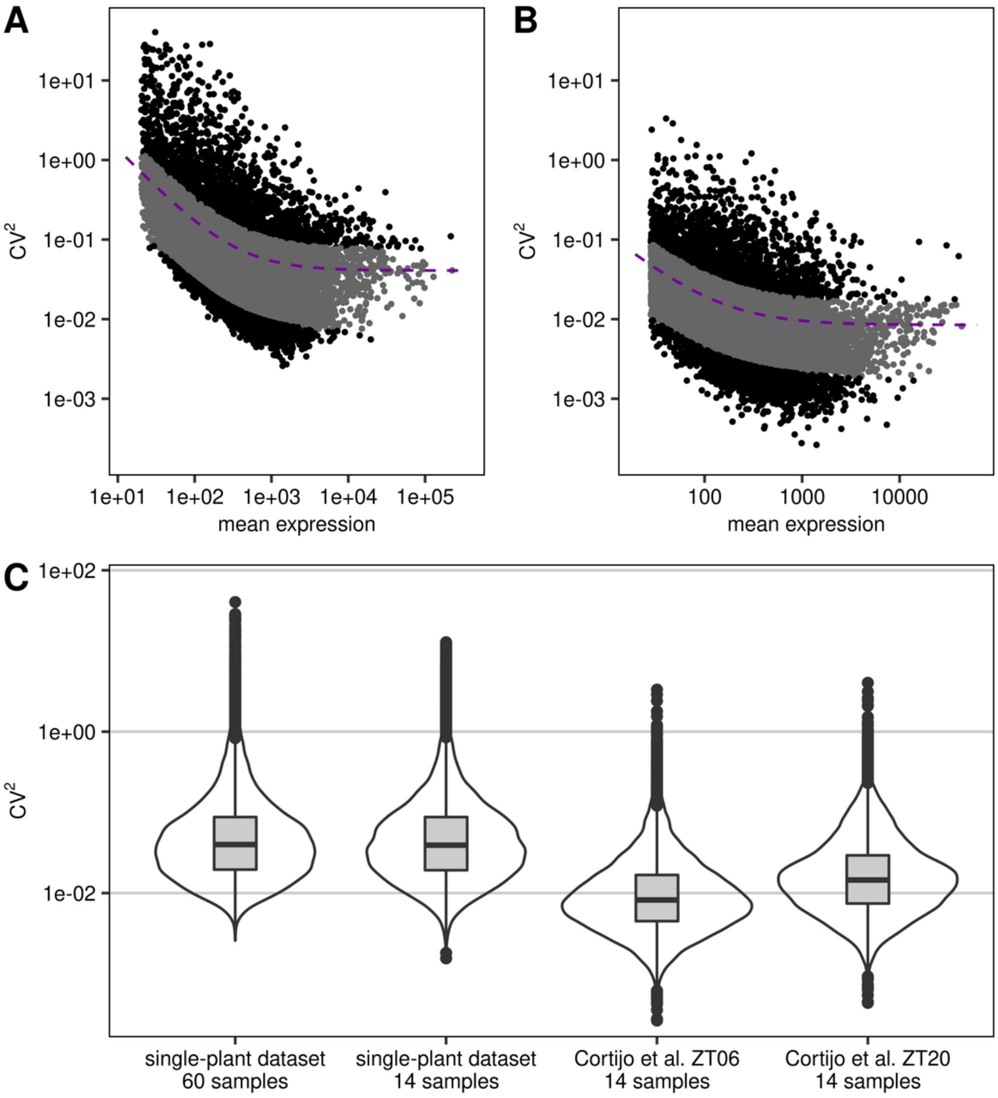

Appendix Figure S5. Gene expression variability in maize single-plant dataset versus Arabidopsis single-plant dataset of Cortijo et al. (2019). (A) Plot of the squared CV versus expression mean for genes in the maize single-plant dataset (60 samples) after removing DOH, BATCH and SNP effects (see Methods) and excluding the 5% lowest-expressed genes. **(B)** Plot of the squared CV versus expression mean for all except the 5% lowest-expressed genes in the *A. thaliana* single-plant dataset of Cortijo et al. (2019) for time point ZT06 (14 samples), which most closely matches the harvesting time point for the field-grown maize plants. In both panels **(A)** and **(B)**, a fitted trendline (see Methods) is shown in purple, and the top and bottom 10% of genes ranked by normalized CV (see Methods) are shown in black. **(C)** Violin plots of the squared CV distribution for, from left to right, the full maize dataset, a representative random subset of 14 samples from the maize dataset, the ZT06 time point of the Cortijo et al. (2019) dataset, and the ZT20 time point of the Cortijo et al. (2019) dataset. The ZT20 time point is the time point for which most expression variability was observed by Coritijo et al. (2019). Boxes stretch from the first to the third quartile, with the median indicated as a horizontal line. Data points outside 1.5 times the interquartile range are shown as dots.

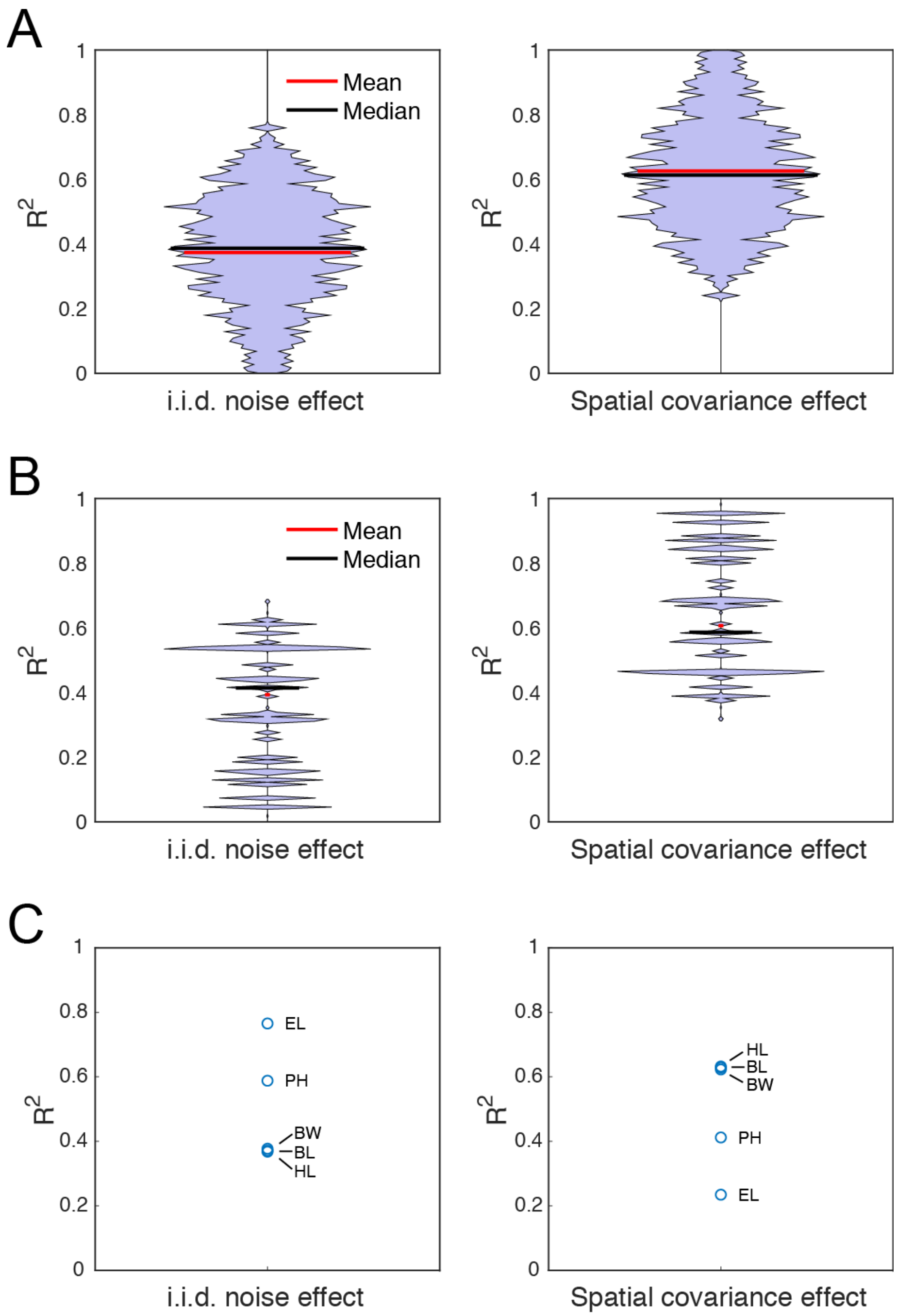

Appendix Figure S6. Proportion of variance in the LME model residuals explained by *i.i.d.* 'noise' and spatial covariance. Violin plots of R^2 value distributions are shown for the independent and identically distributed (*i.i.d.*) and spatially covarying portions of the LME model residuals for transcripts **(A)** and metabolites **(B)** with significant spatial patterning, as judged from Moran's I ($q \leq 0.01$), and for all phenotypes **(C)**. BL = leaf 16 blade length, BW = leaf 16 blade width, HL = husk leaf length, EL = ear length and PH = plant height.

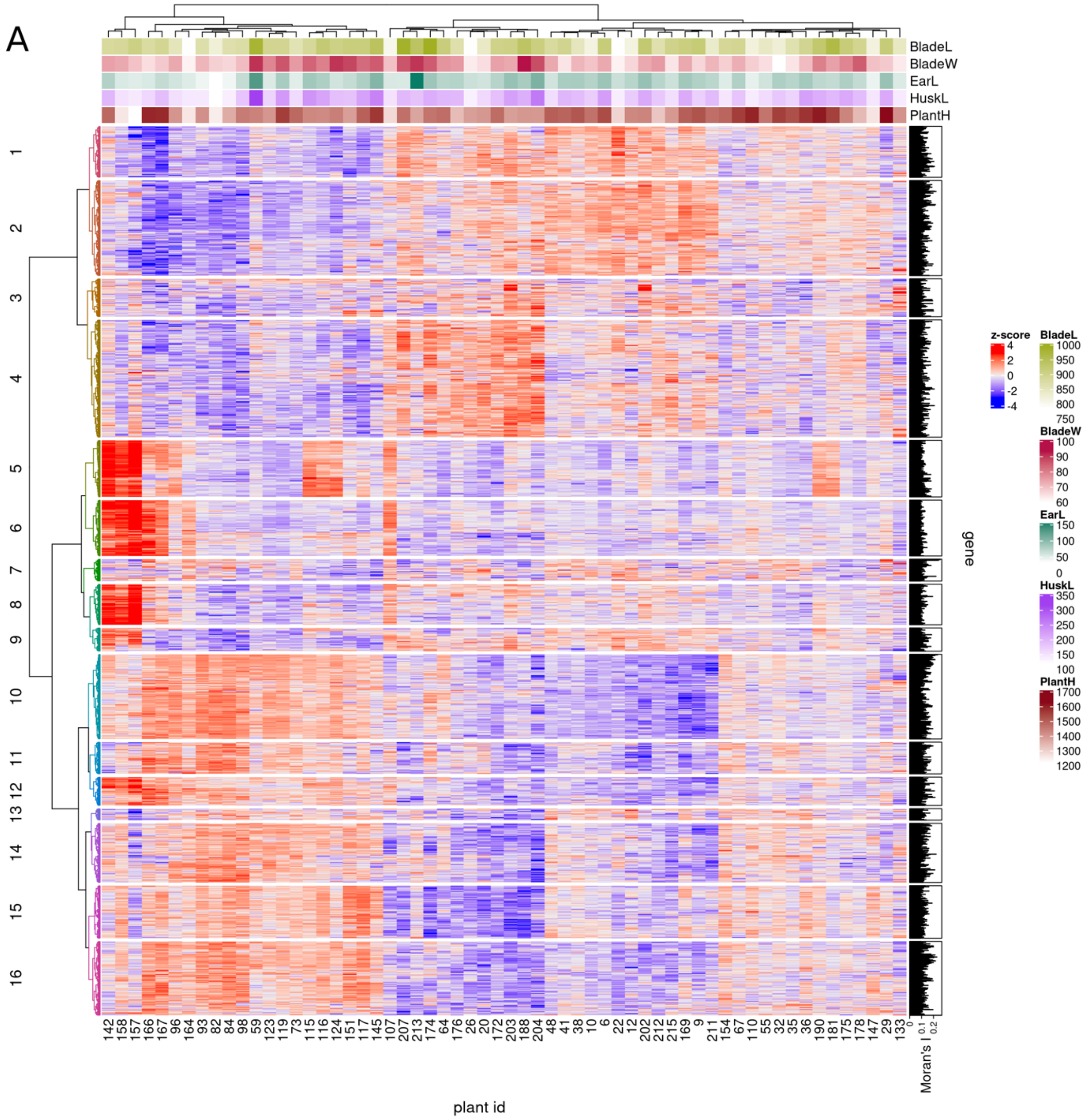

Appendix Figure S7. Average expression of spatially autocorrelated transcript clusters mapped to the field. (A) Hierarchical clustering of spatially autocorrelated transcriptome profiles. Cluster numbers are indicated on the left. Phenotype profiles are shown on top. BladeL = leaf 16 blade length, BladeW = leaf 16 blade width, EarL = ear length, HuskL = husk leaf length, PlantH = plant height. **(B)** Each plot on the following pages displays the averaged z-scored gene expression profile of a spatially autocorrelated transcript cluster mapped to the field. Shown on top of each plot are the Moran's I of the average cluster profile (computed using ape::Moran.I in R, see Methods) and the corresponding *p*-value and *q*-value (computed using the Benjamini-Hochberg method across all clusters). The scales on the top and to the right of the field maps give field plot dimensions in cm.

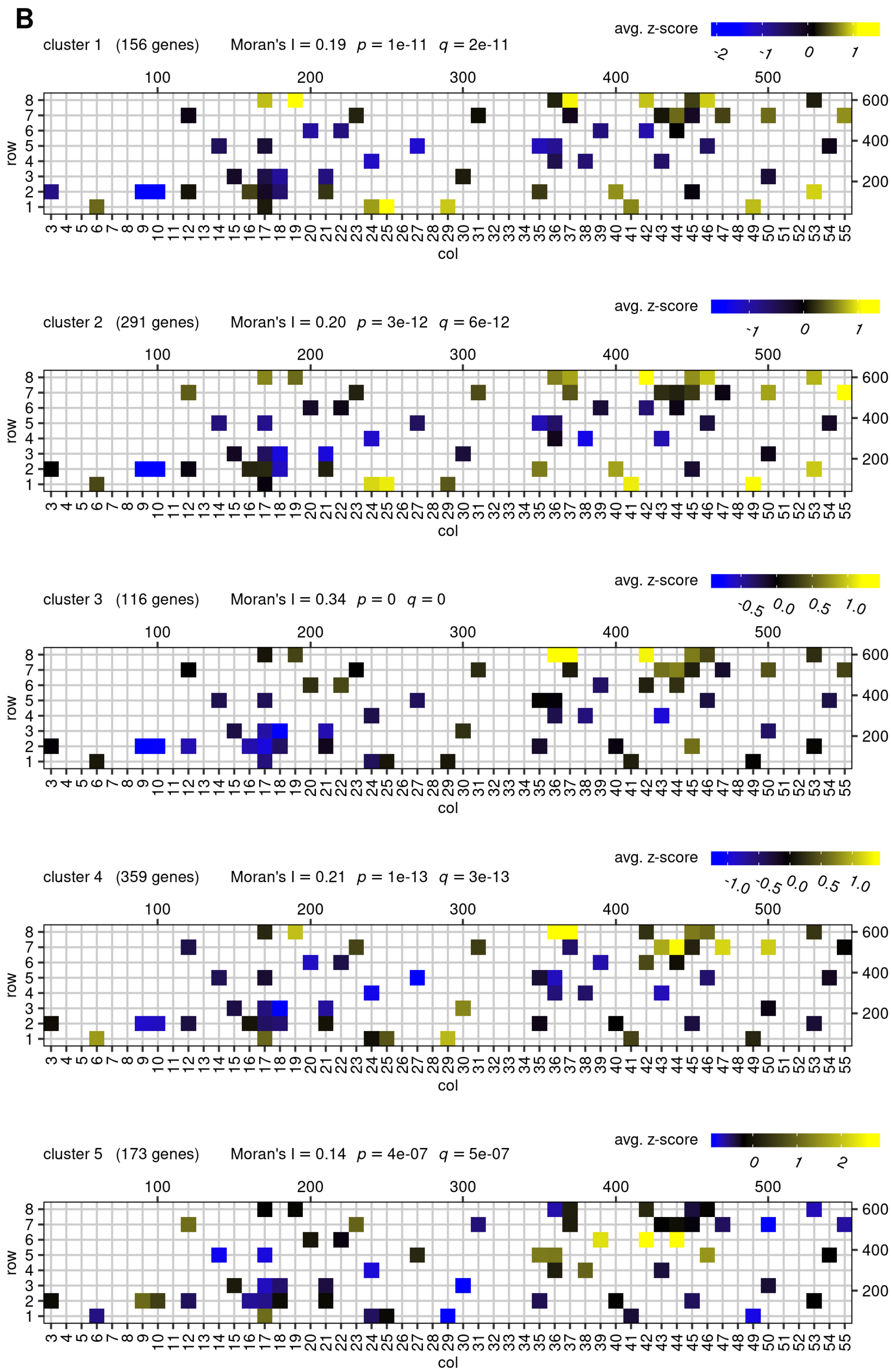

Appendix Figure S7. (continued)

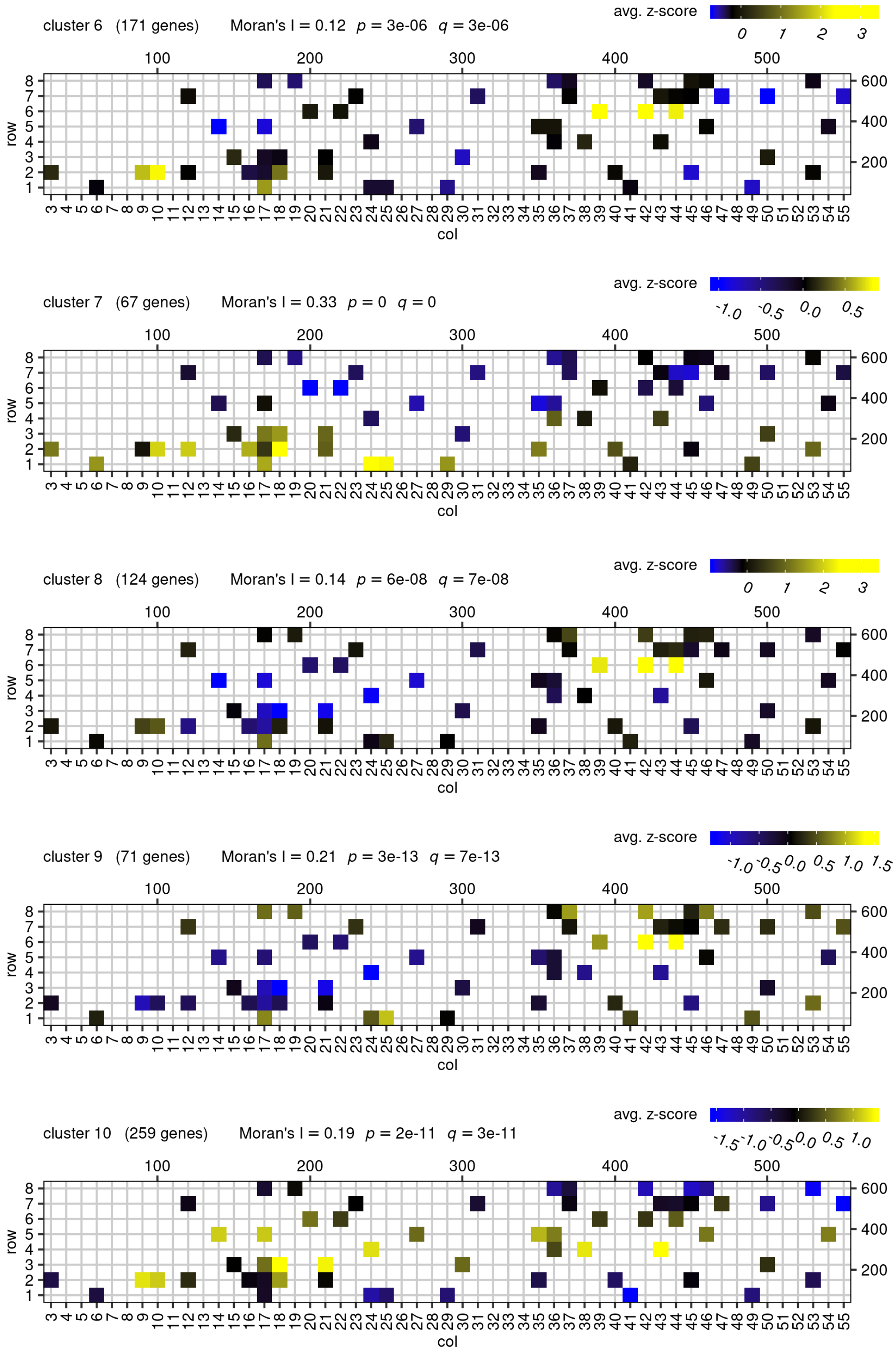

Appendix Figure S7. (continued)

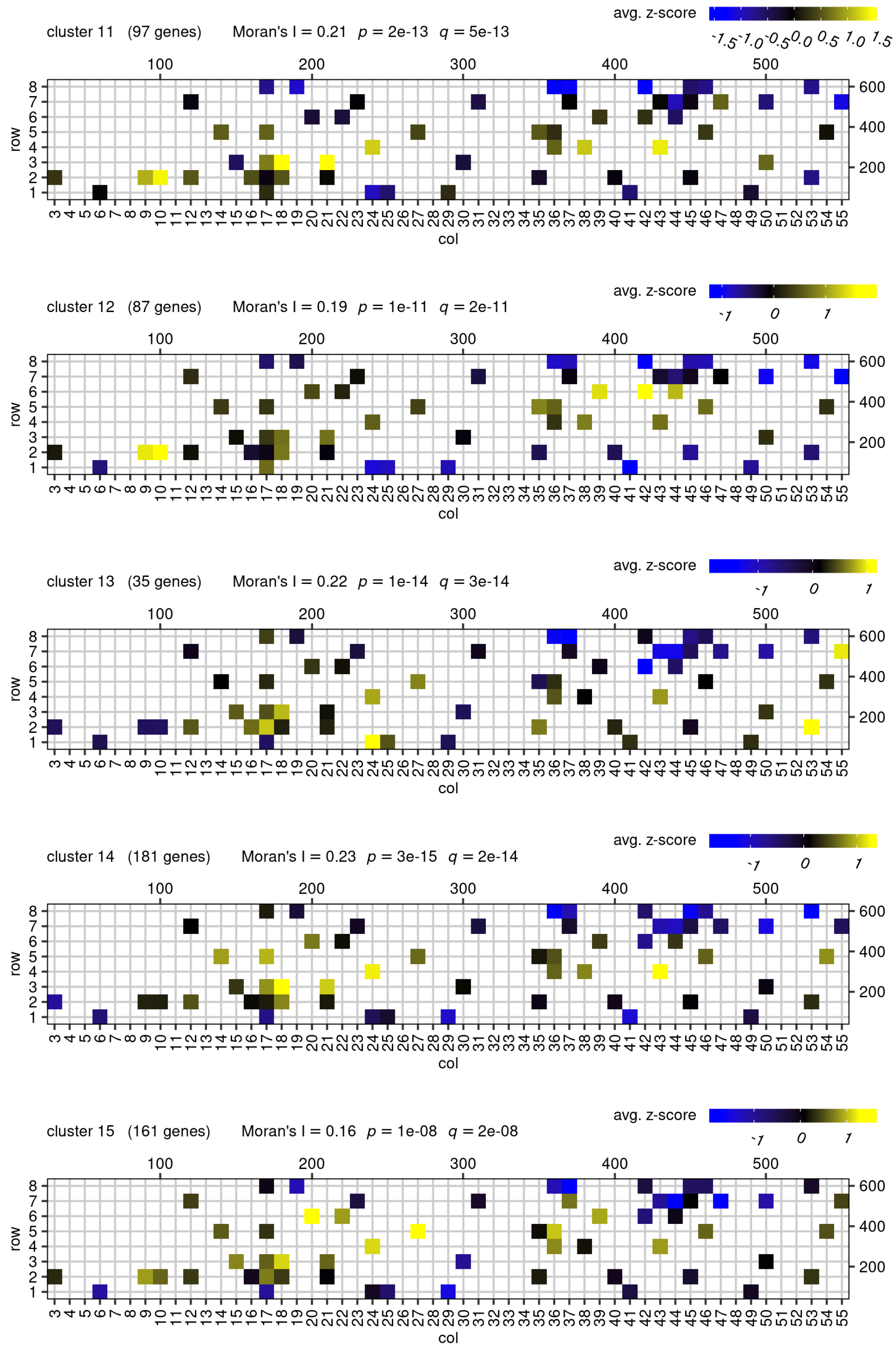

Appendix Figure S7. (continued)

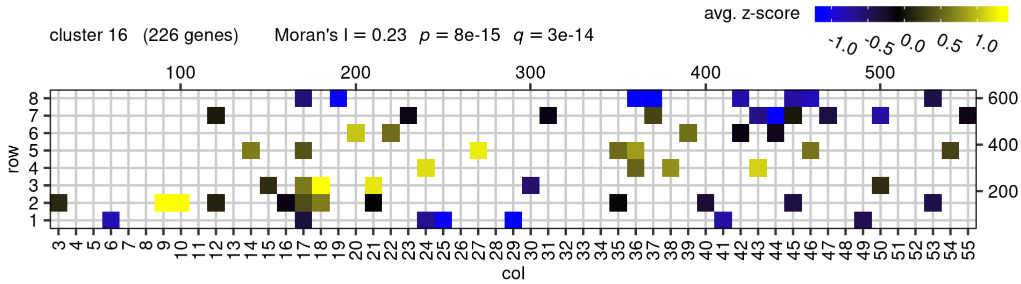

Appendix Figure S7. (continued)

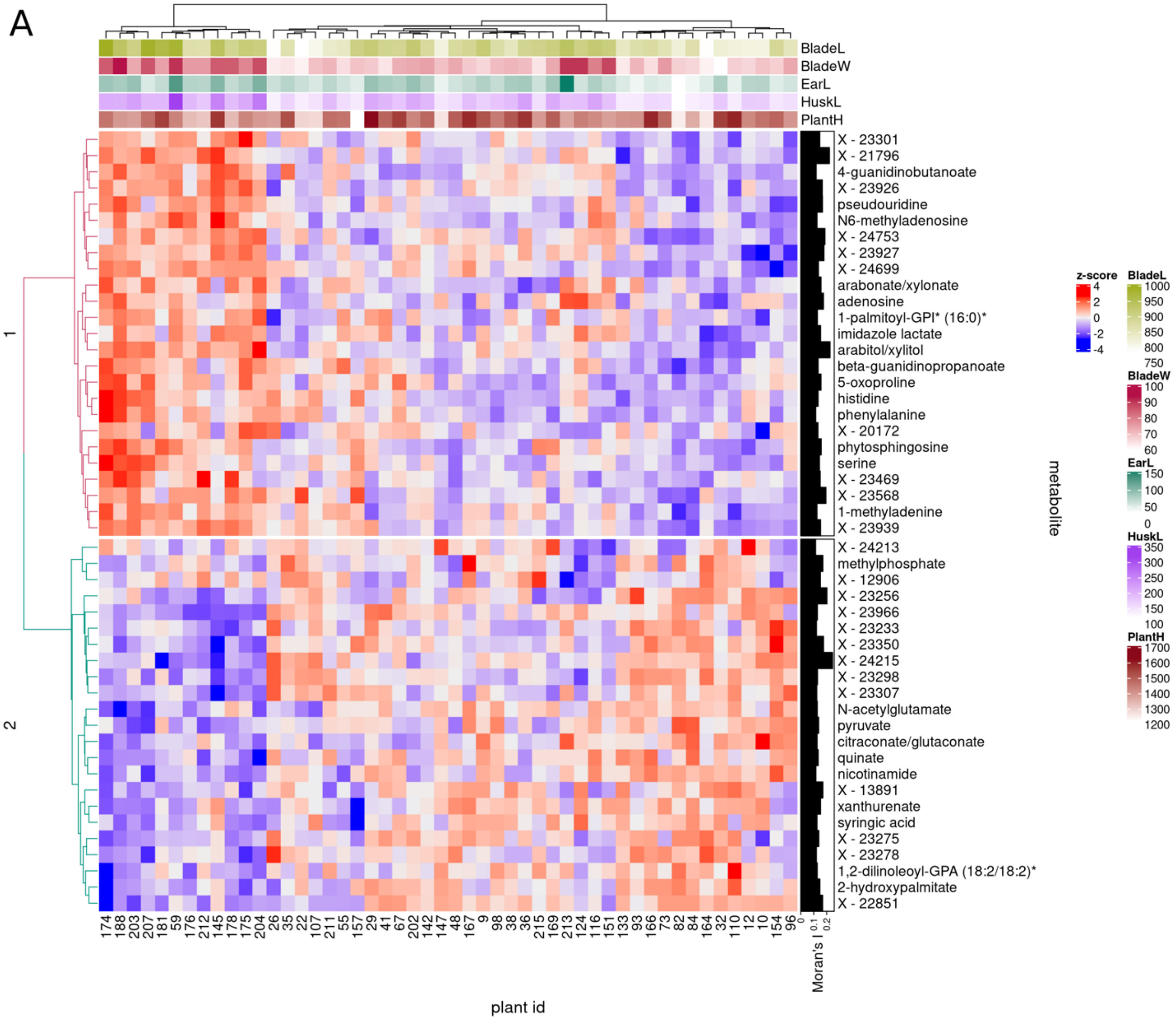

Appendix Figure S8. Average expression of spatially autocorrelated metabolite clusters mapped to the field. (A) Hierarchical clustering of spatially autocorrelated metabolome profiles. Cluster numbers are indicated on the left. Phenotype profiles are shown on top. BladeL = leaf 16 blade length, BladeW = leaf 16 blade width, EarL = ear length, HuskL = husk leaf length, PlantH = plant height. **(B)** Each plot on the following page displays the averaged z-scored metabolite expression profile of a spatially autocorrelated metabolite cluster mapped to the field. Shown on top of each plot are the Moran's I of the average cluster profile (computed using ape::Moran.I in R, see Methods) and the corresponding *p*-value and *q*-value (computed using the Benjamini-Hochberg method across all clusters). The scales on the top and to the right of the field maps give field plot dimensions in cm.

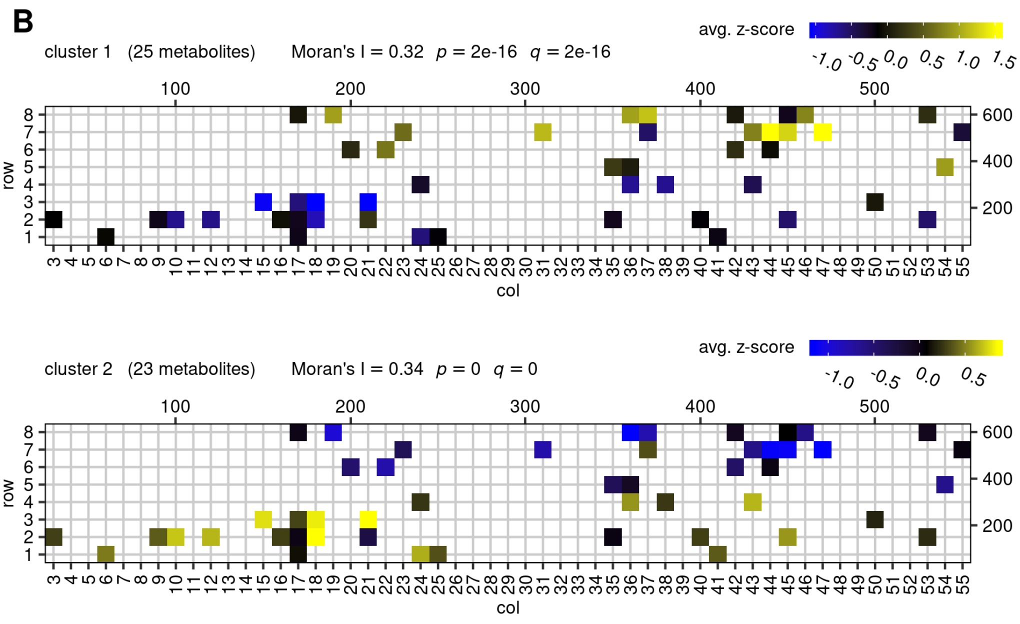

Appendix Figure S8. (continued)

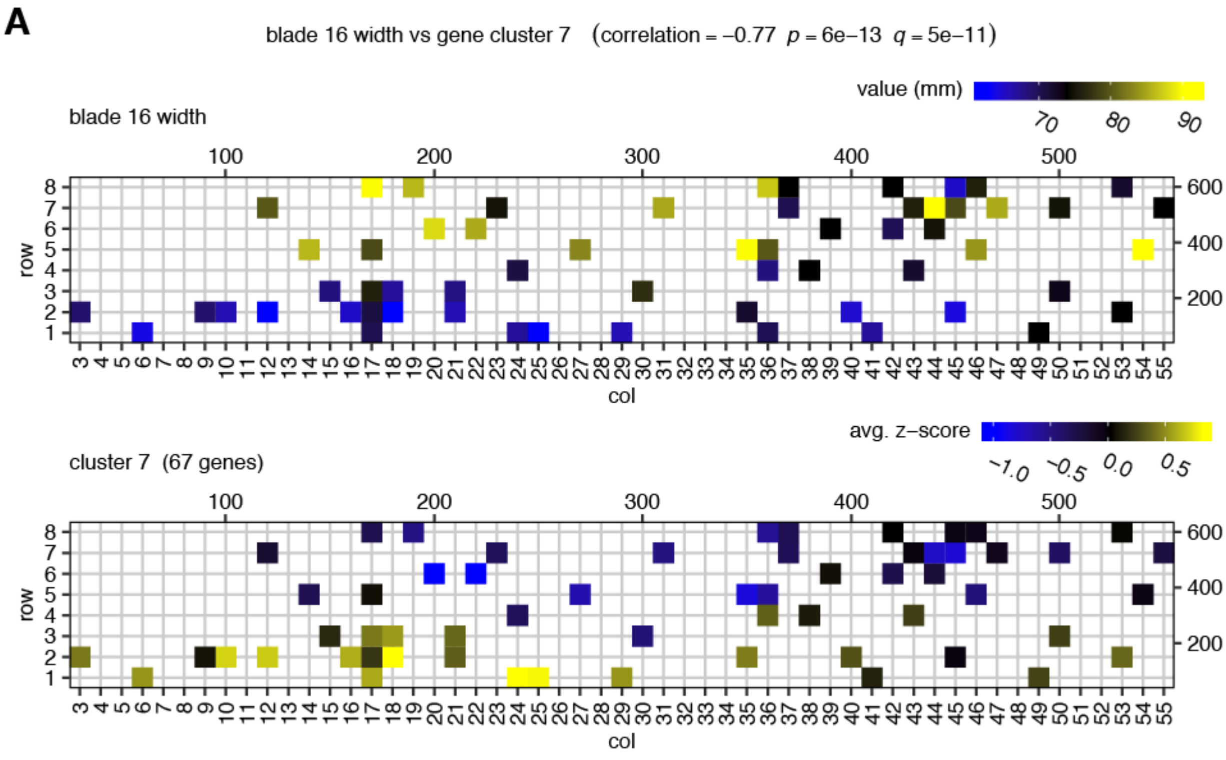

Appendix Figure S9. Significant correlations of the average expression profiles of spatially autocorrelated transcript clusters with phenotypes. Each panel (see also following pages) contains two plots. The bottom plot displays the average z-scored gene expression profile of a spatially autocorrelated transcript cluster mapped to the field. The top plot displays a phenotype that correlates significantly with this average expression profile ($q \leq 0.05$). Shown on top of each panel are the Pearson's correlation between the average cluster expression profile and the phenotype, the corresponding *p*-value (computed using cor.test in R) and the corresponding *q*-value (computed using the Benjamini-Hochberg method on all comparisons per phenotype). The scales on the top and to the right of the field maps give field plot dimensions in cm.

B

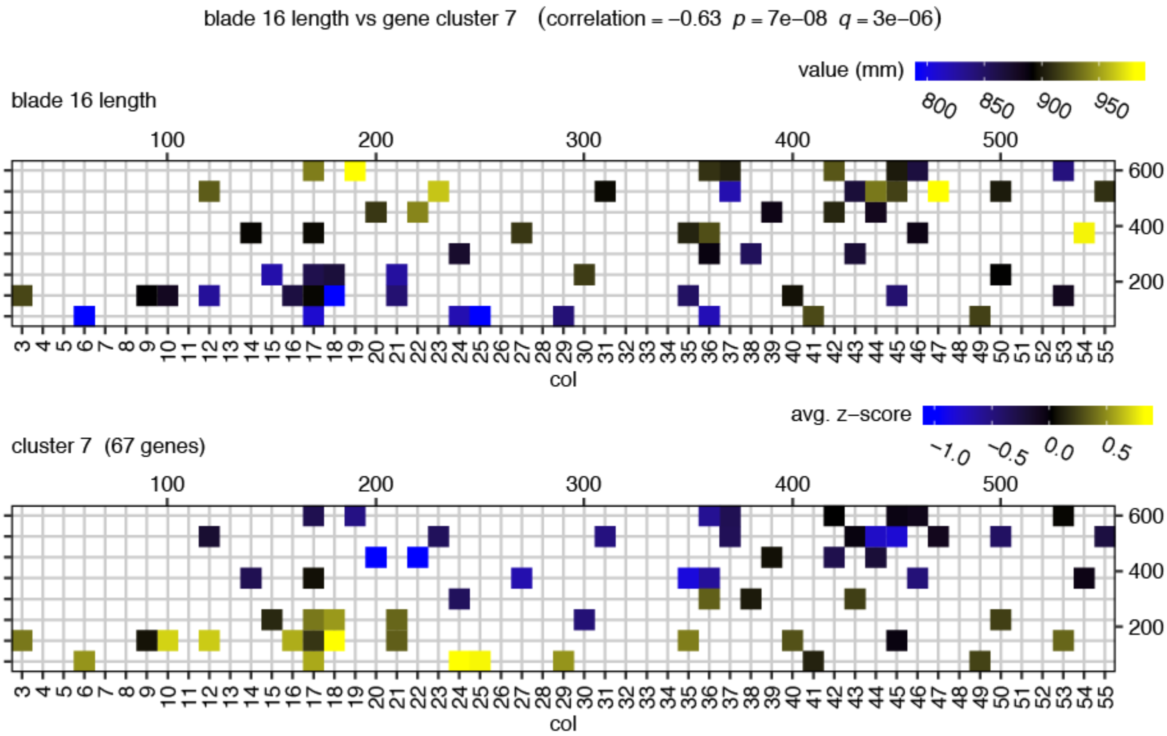

C

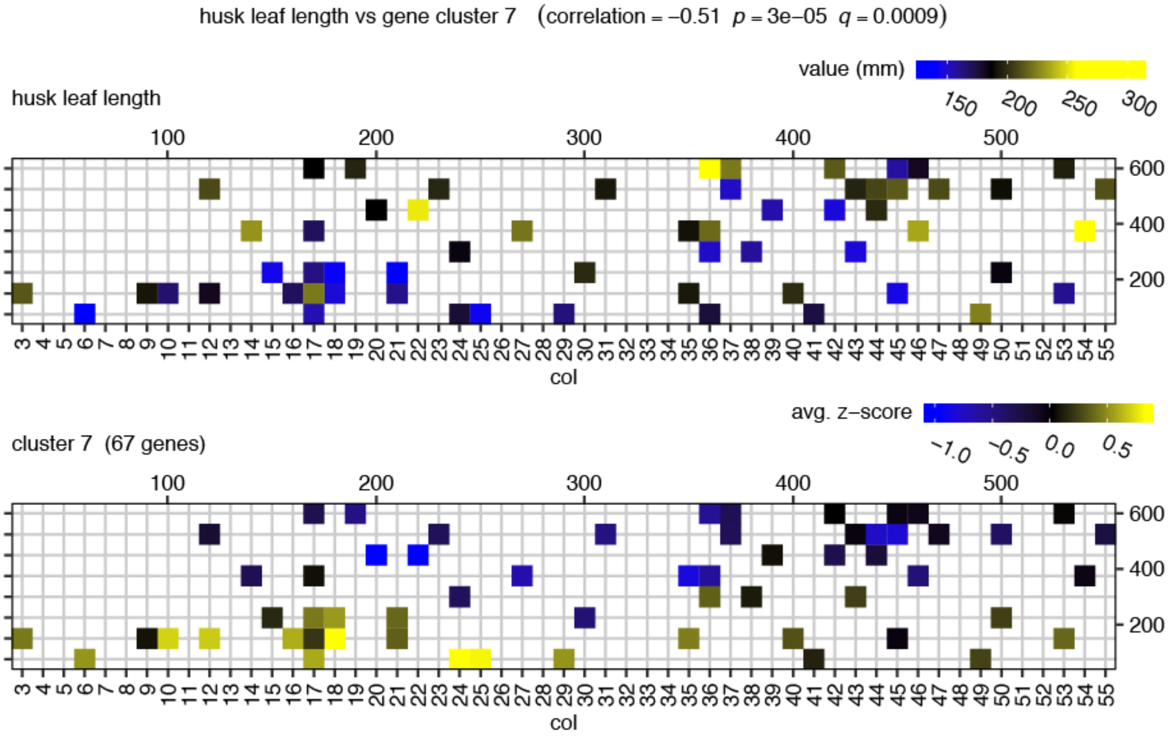

Appendix Figure S9. (continued)

D

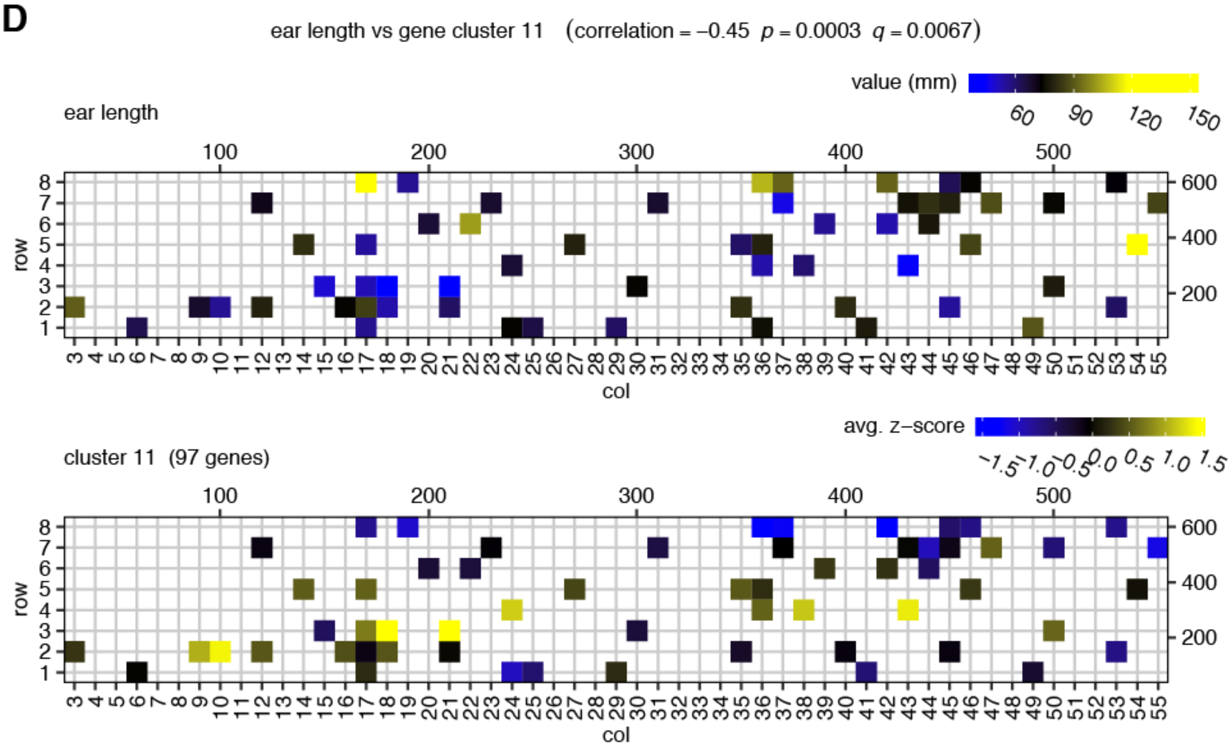

E

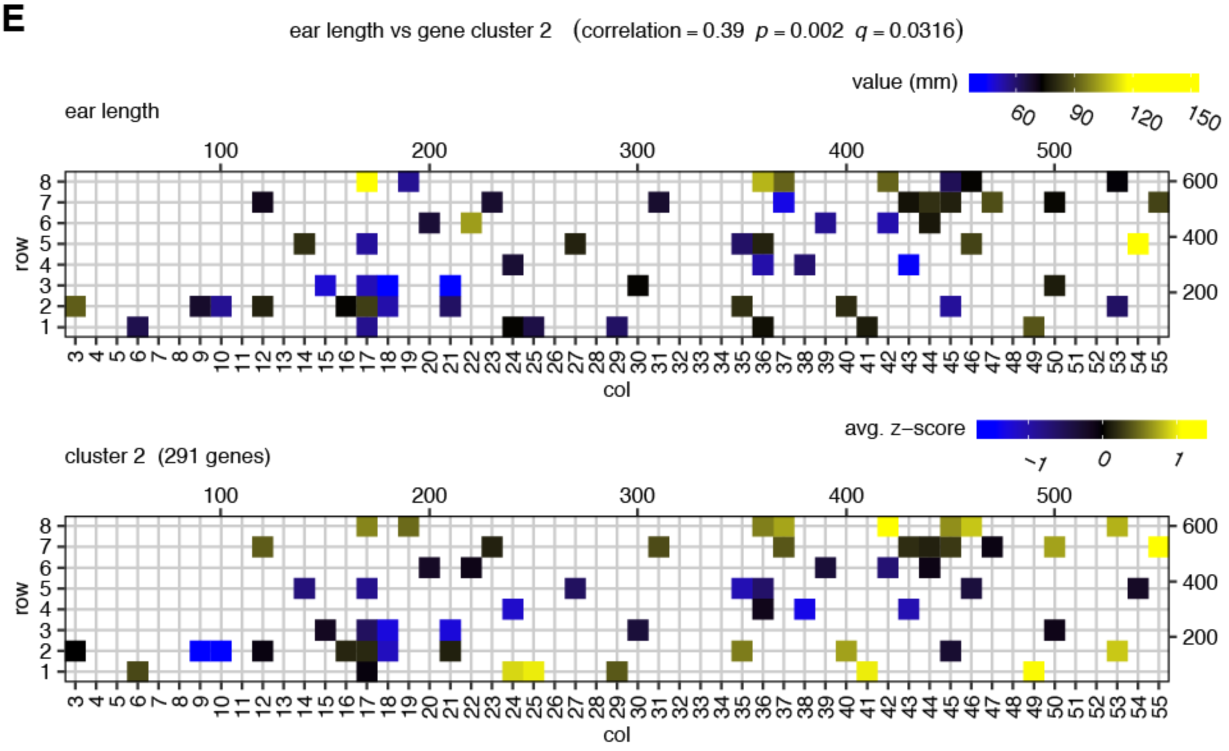

Appendix Figure S9. (continued)

A

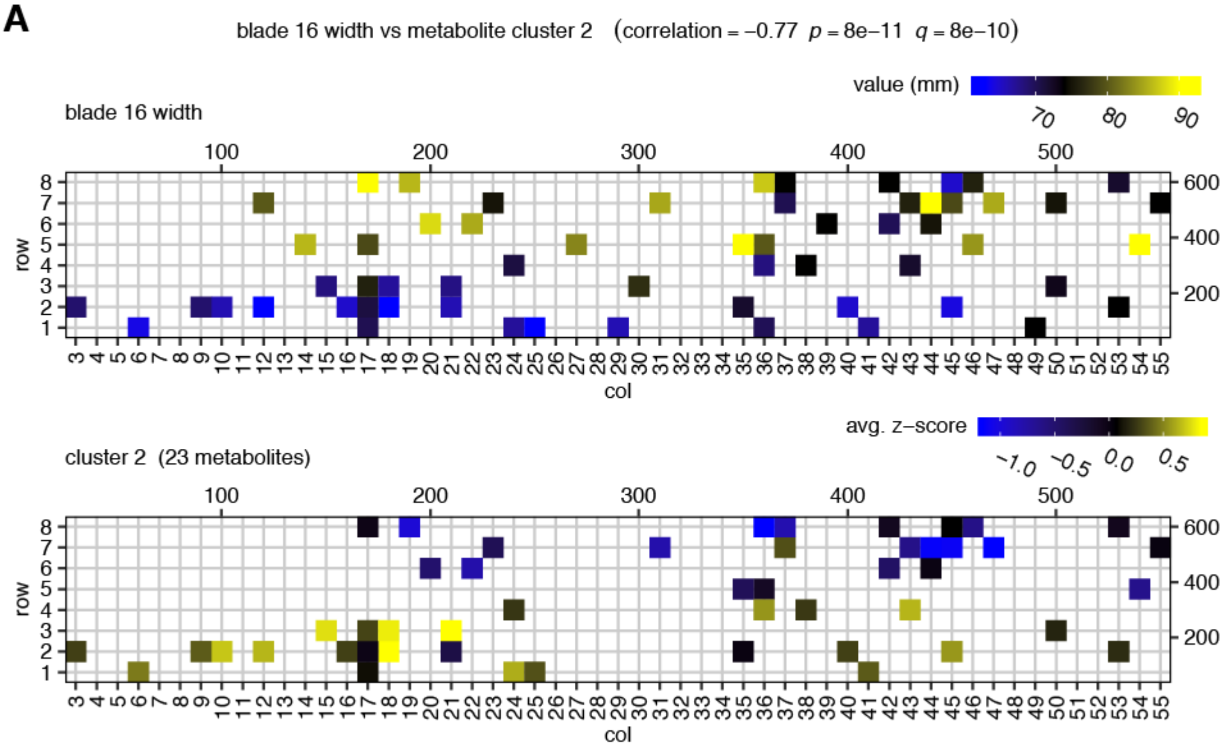

Appendix Figure S10. Significant correlations of the average profiles of spatially autocorrelated metabolite clusters with phenotypes. Each panel (see also following pages) contains two plots. The bottom plot displays the average z-scored metabolite profile of a spatially autocorrelated metabolite cluster mapped to the field. The top plot displays a phenotype that correlates significantly with this average profile ($q \leq 0.05$). Shown on top of each panel are the Pearson's correlation between the average cluster profile and the phenotype, the corresponding p-value (computed using cor.test in R) and the corresponding q-value (computed using the Benjamini-Hochberg method on all comparisons per phenotype). The scales on the top and to the right of the field maps give field plot dimensions in cm.

B

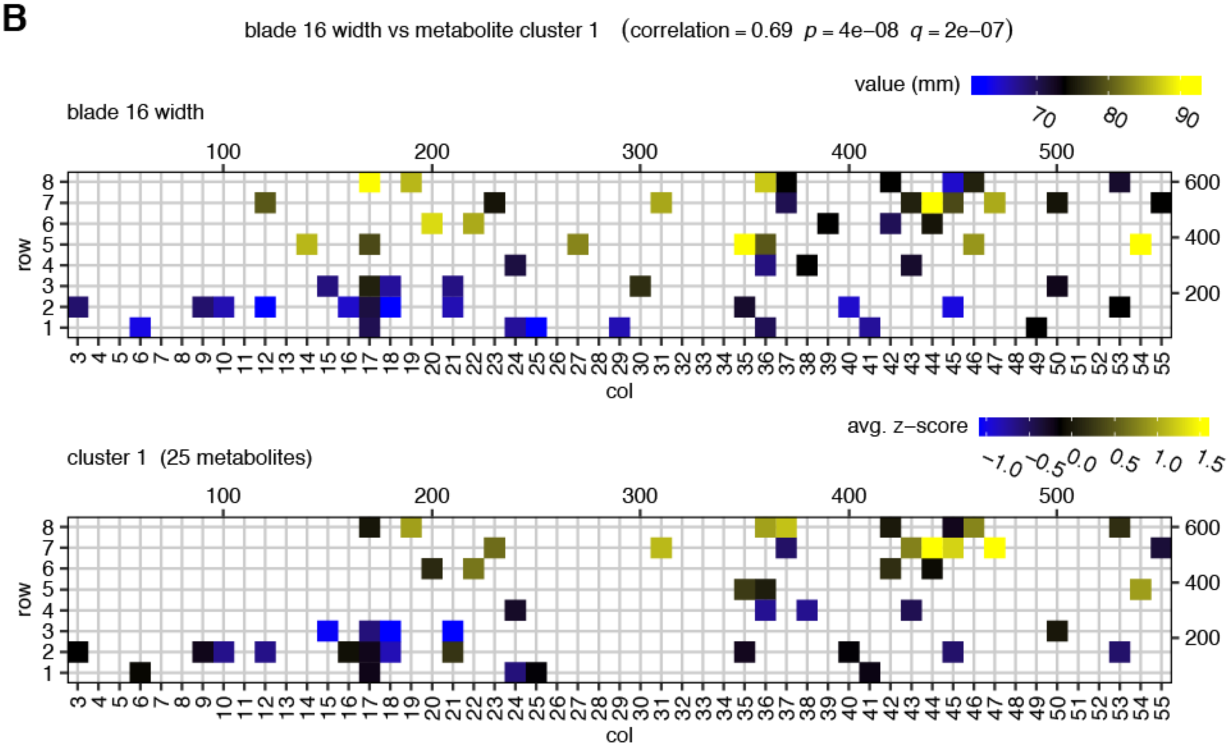

C

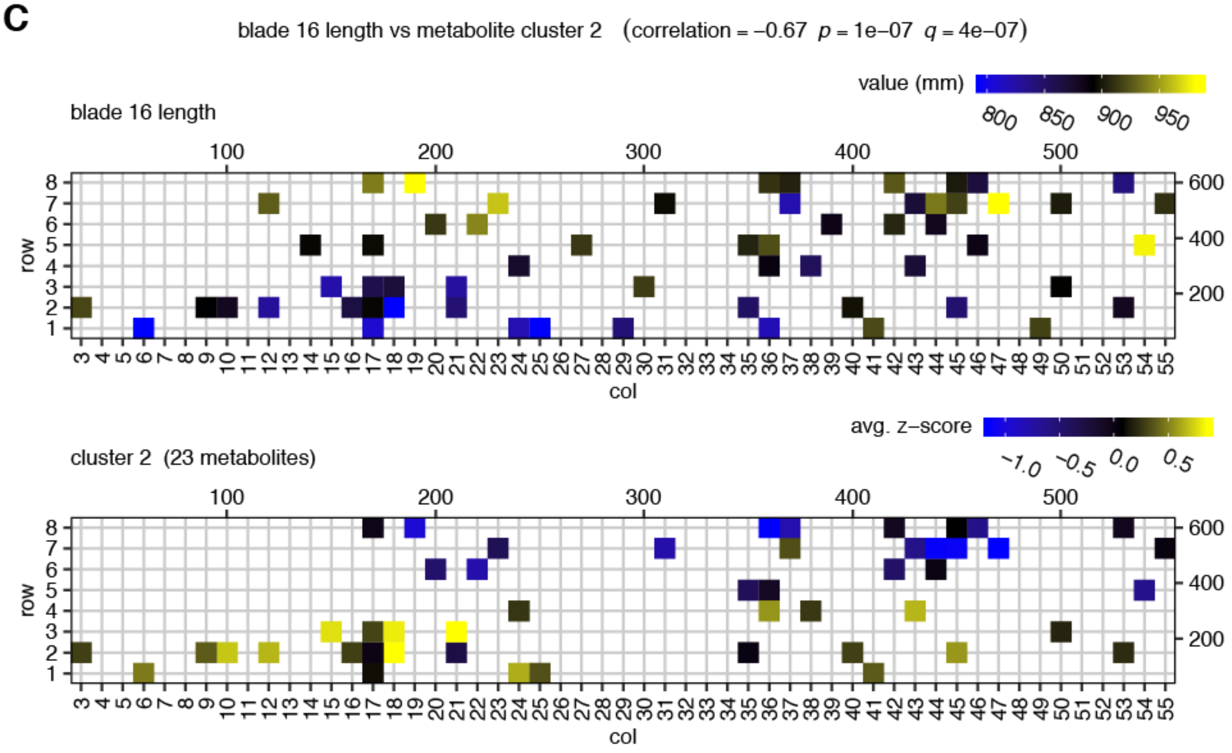

Appendix Figure S10. (continued)

D

husk leaf length vs metabolite cluster 2 (correlation = −0.66 p = 2e−07 q = 6e−07)

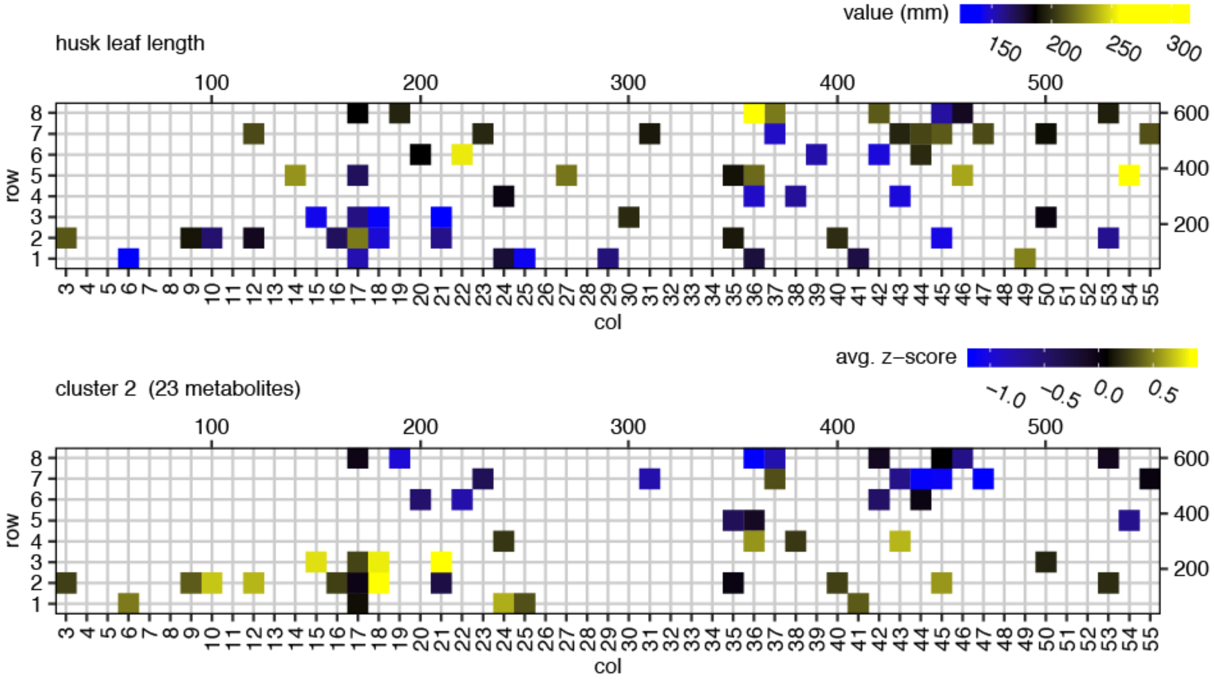

E

husk leaf length vs metabolite cluster 1 (correlation = 0.64 p = 5e−07 q = 9e−07)

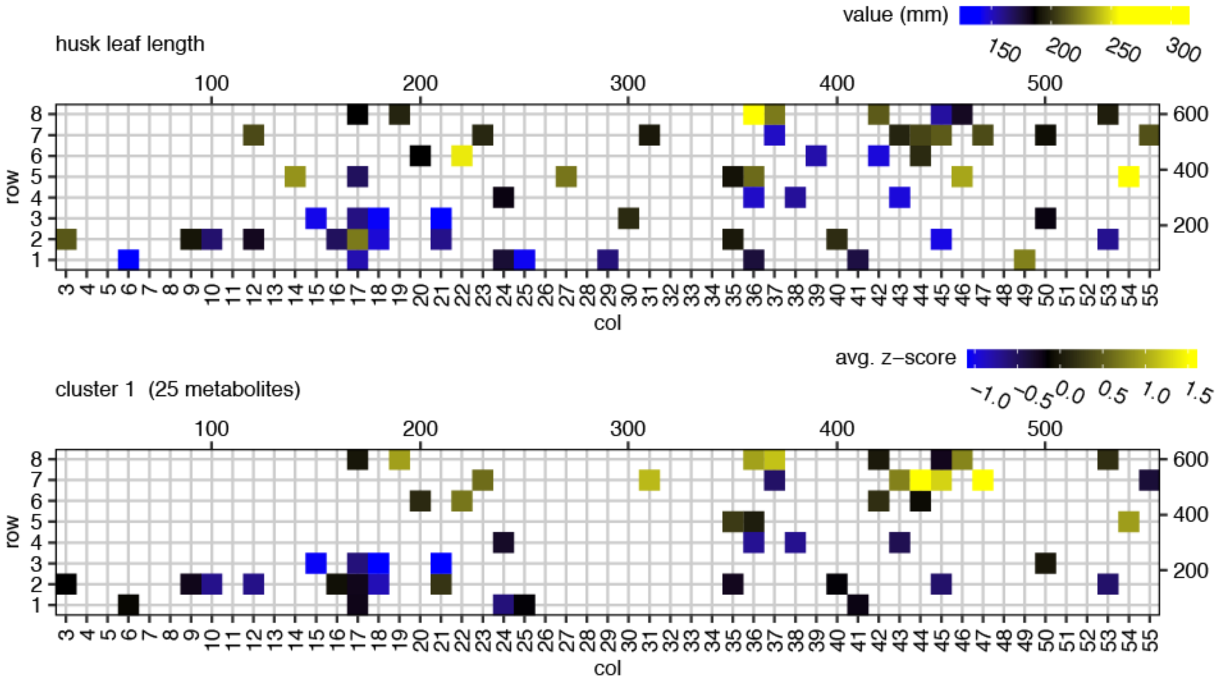

Appendix Figure S10. (continued)

F

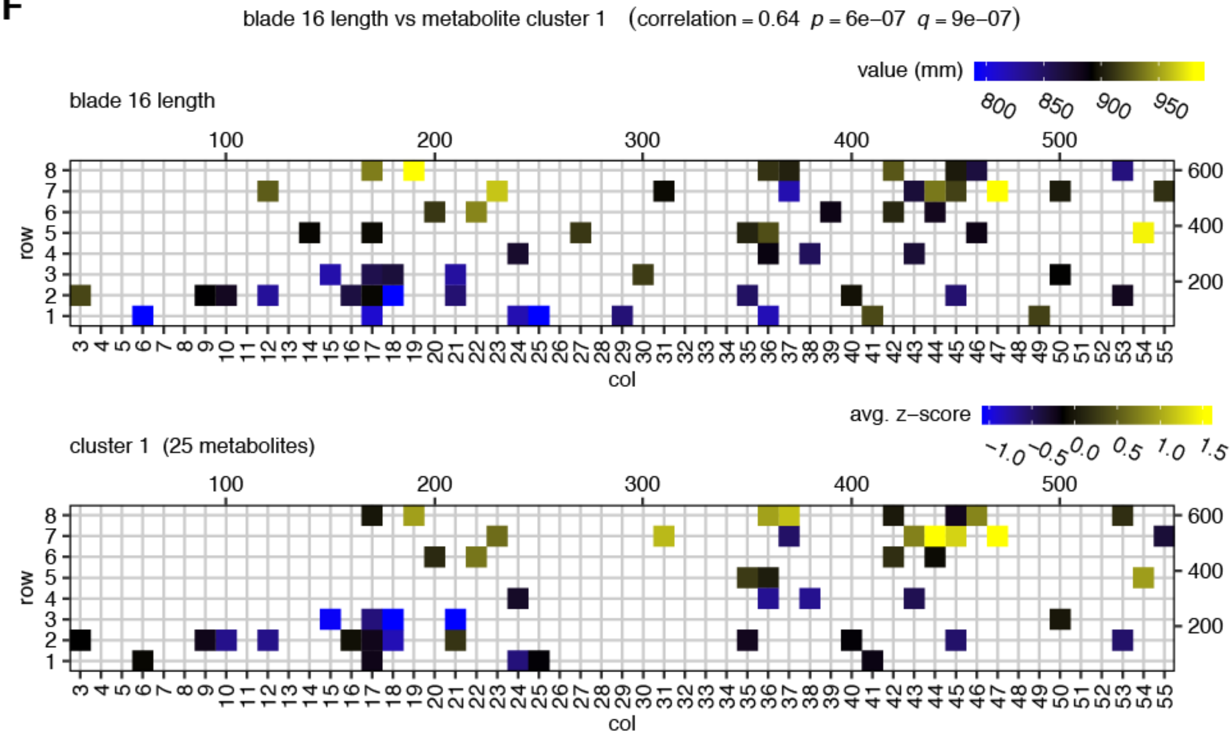

G

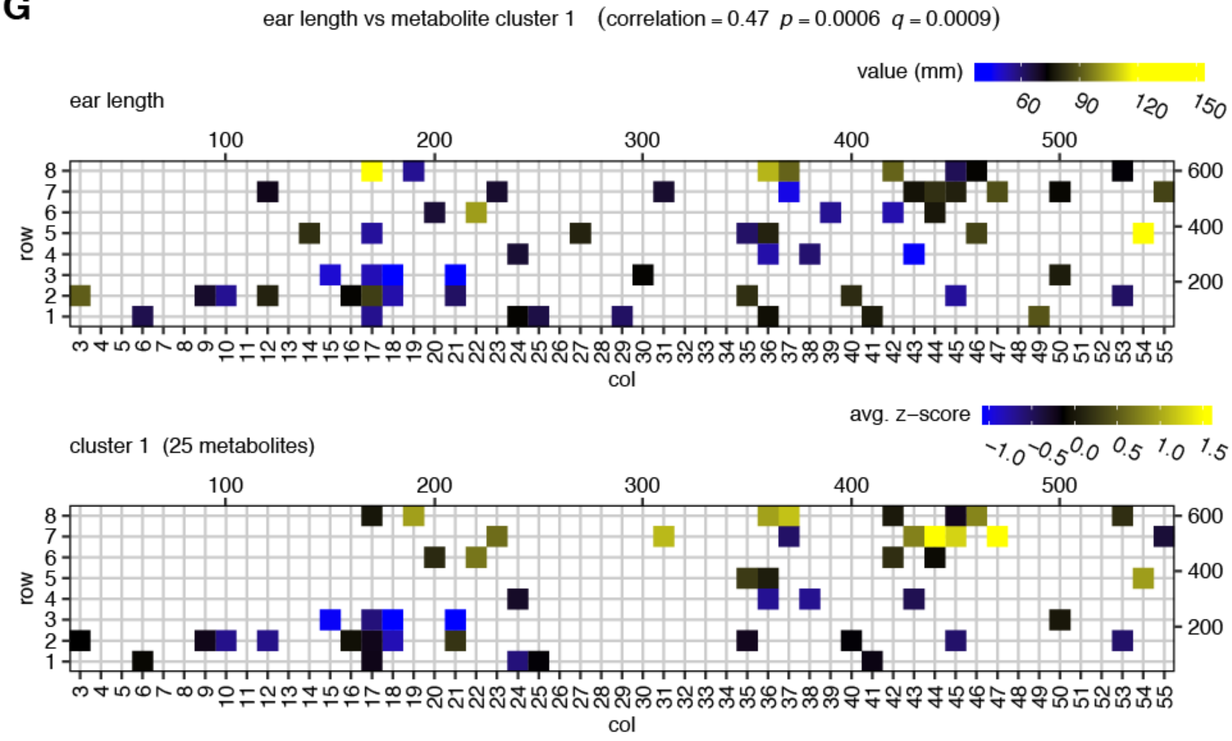

Appendix Figure S10. (continued)

H

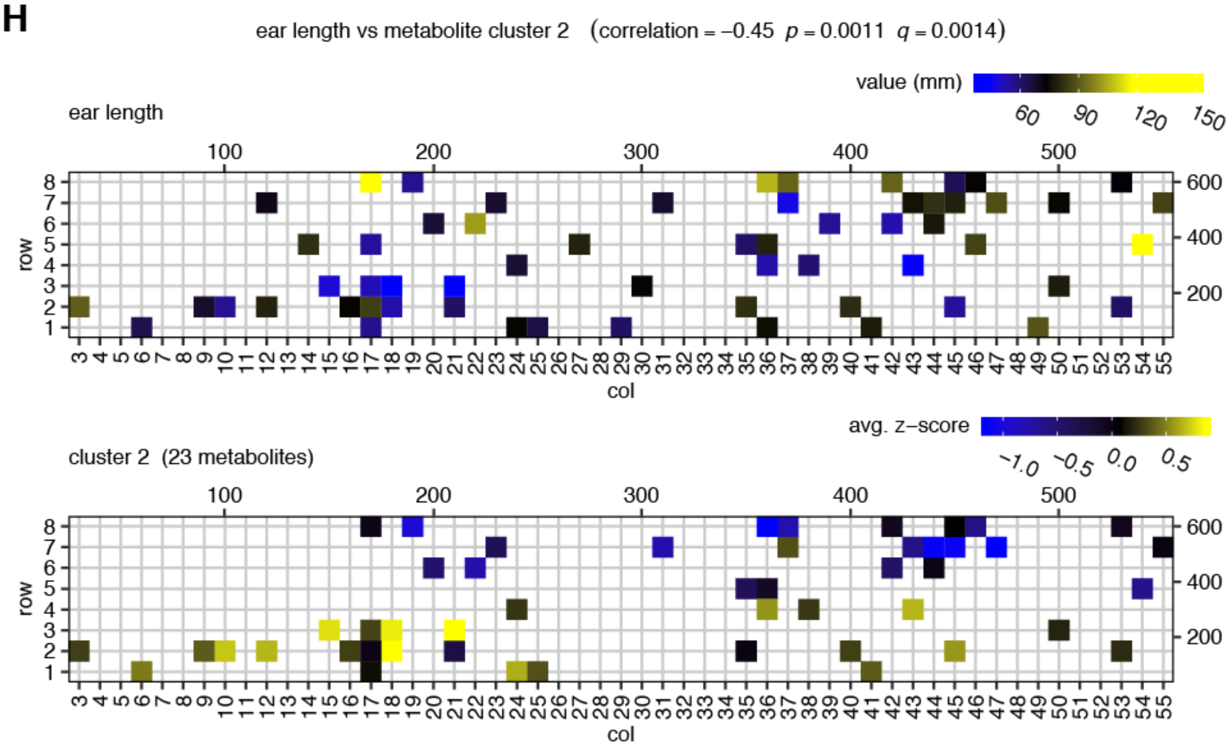

Appendix Figure S10. (continued)

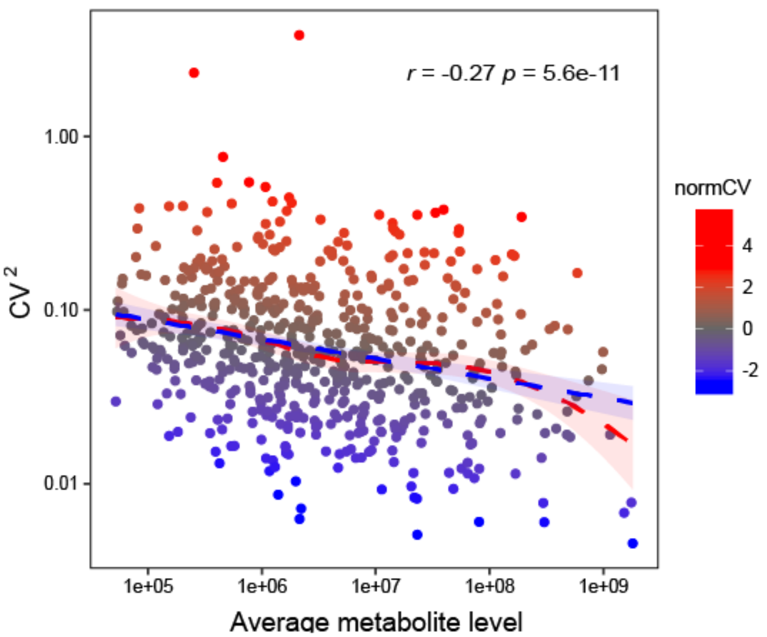

Appendix Figure S11. Metabolite level variability in the maize single-plant dataset. Shown is a plot of the squared CV versus average level for all metabolites in the maize single-plant dataset (50 samples) after removing DOH, BATCH and SNP effects (see Methods). The blue dashed line is a linear regression trendline in log space. The regression coefficient and its *p*-value are shown in the top right corner. The red dashed line is a LOESS fit showing that the relationship between both log-transformed variables is indeed roughly linear. Data points are colored according to their normalized CV value (*normCV*, color scale on the right).

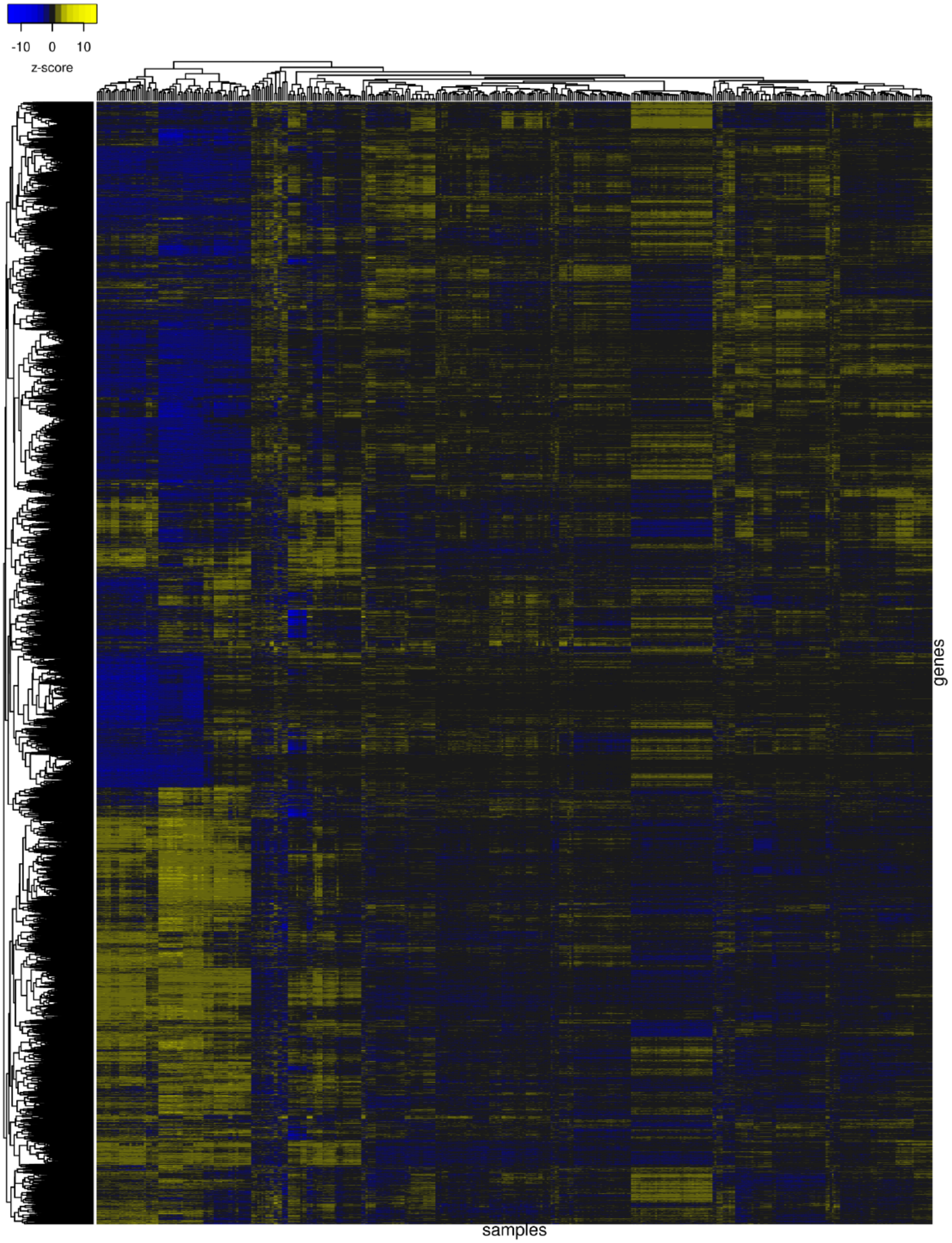

Appendix Figure S12. Hierarchical clustering of the maize B73 leaf transcriptome datasets obtained from the SRA database. Rows are gene expression profiles and columns are SRA sample expression profiles. Transcript profiles were z-scaled to make them comparable.

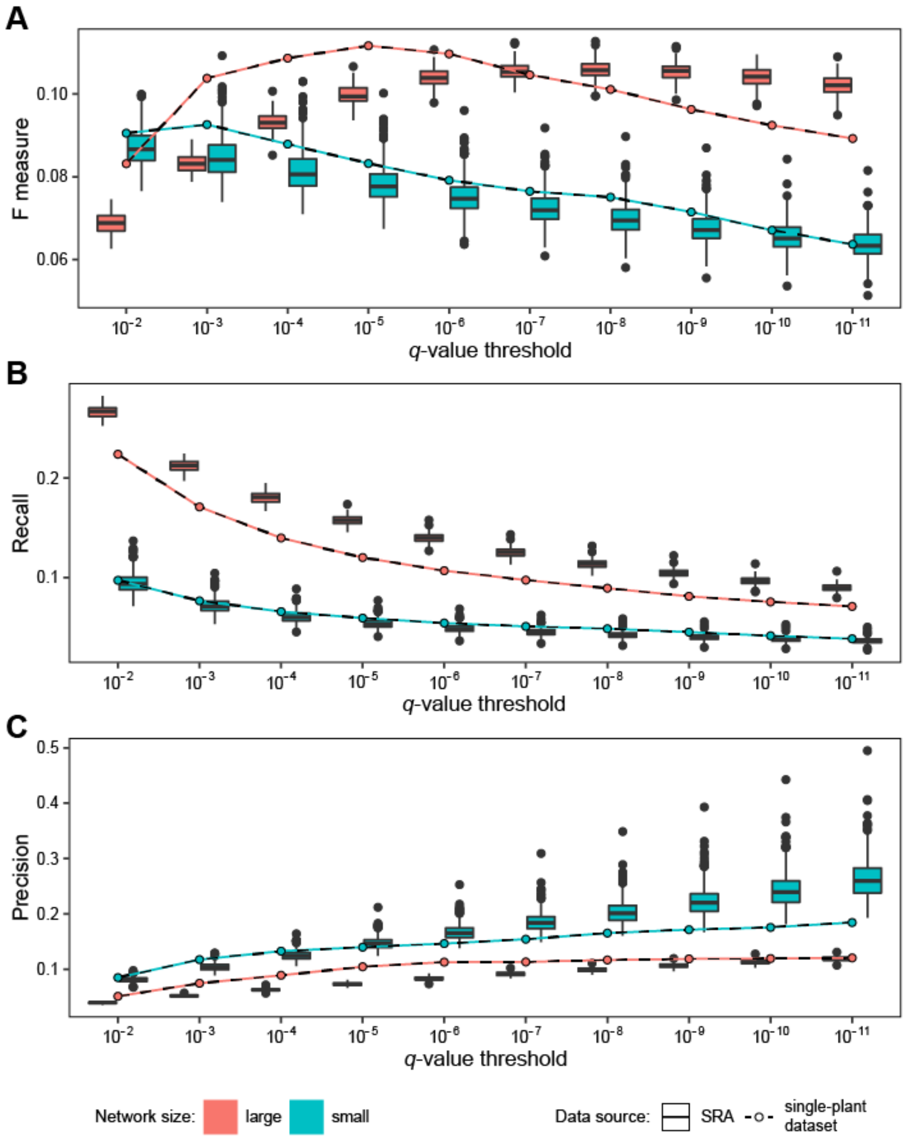

Appendix Figure S13. Gene function prediction performance for coexpression networks of different size. Gene function prediction was done on networks learned from the single-plant data and from sampled SRA datasets (see Methods). For the small network performance stats (depicted in blue-green), both the single-plant network and the 500 sampled networks were thresholded to contain 878,079 edges (the same number as in the single-plant network at a Bonferroni-corrected *p*-value cutoff of 0.01 for edge significance). For the large network performance stats, 100 sampled networks were generated with 4,680,819 edges (the average amount of edges obtained for the sampled networks at a Bonferroni-corrected *p*-value cutoff of 0.01), and the single-plant network was thresholded to contain the same amount of edges. Panels **(A)** to **(C)** depict F-measure, recall and precision curves for the resulting networks at various FDR thresholds, as in Fig 5. Except at lower confidence thresholds ($q \geq 10^{-3}$), larger networks generally have higher gene function prediction performance, as measured by the F-measure.

Beta-glucan metabolic process. Very Poor

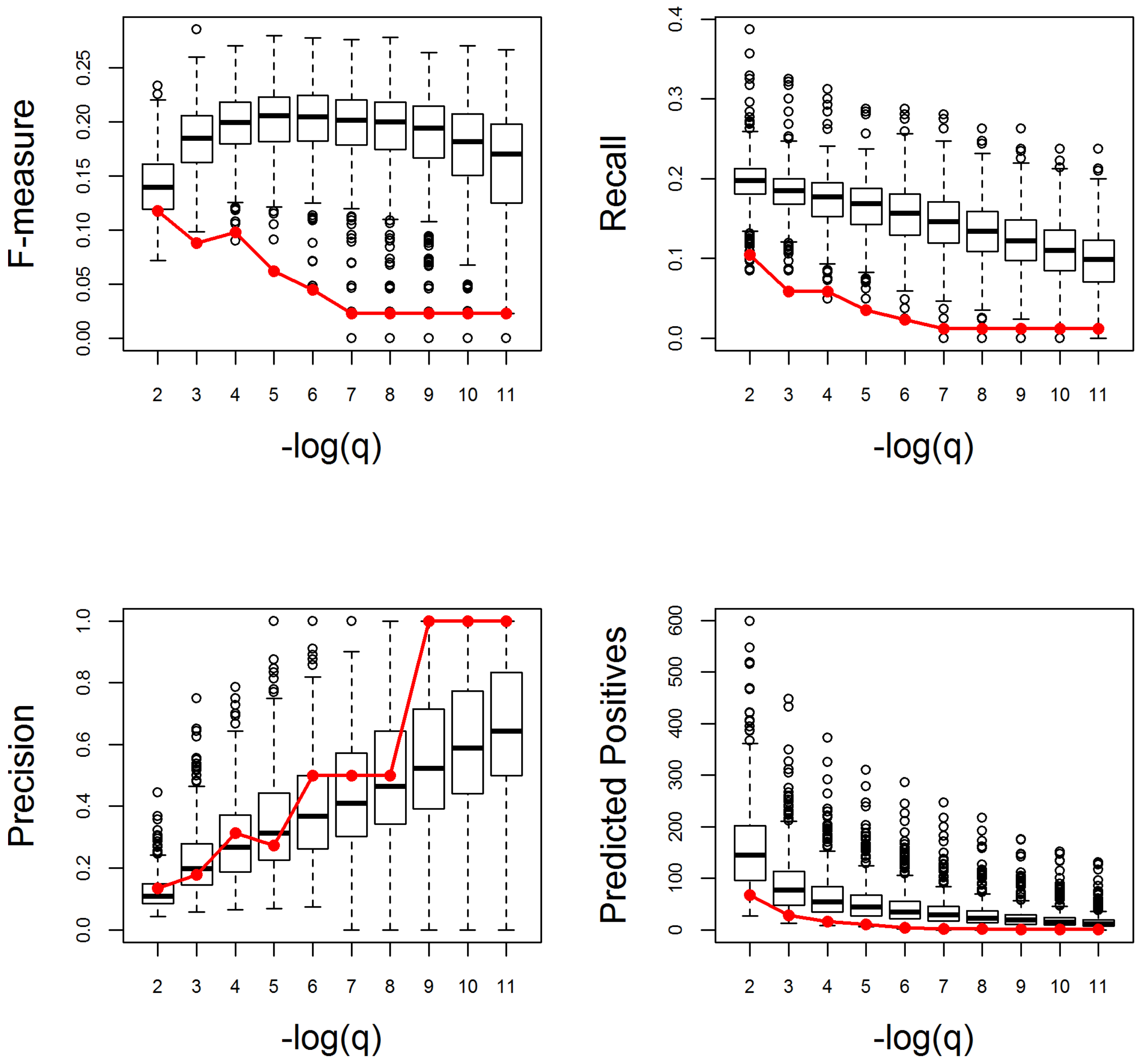

Appendix Figure S14. Gene function prediction performance plots for the GO categories listed in Dataset EV9. For each GO category, four panels are shown depicting the F-measure, recall, precision and the number of predicted positives (true positives + false positives) for the single plant network (red line) and the sampled SRA networks (box-and-whisker plots) for FDR thresholds ranging from 10^{-2} to 10^{-11}. Very general and uninformative GO categories, such as ‘biological process’, are not plotted. In addition, GO categories for which the single plant network and over half of the sampled networks produced no predictions at $q \leq 0.01$ are omitted (see Methods). When a network produced zero predictions at a certain FDR threshold, the F-measure, recall and precision were set to zero in order to make a fair comparison to other networks that do have predictions at this FDR threshold. Boxes extend from the 25th to the 75th percentile, with the median indicated by the central black line. Whiskers extend from each end of the box to the

most extreme values within 1.5 times the interquartile range from the respective end. Data points beyond this range are displayed as little black circles. The label next to the GO name on top of each plot (Very Good, Good, Average, Poor, Very Poor) indicates how well the single plant network scored compared to the sampled SRA networks (see Methods).

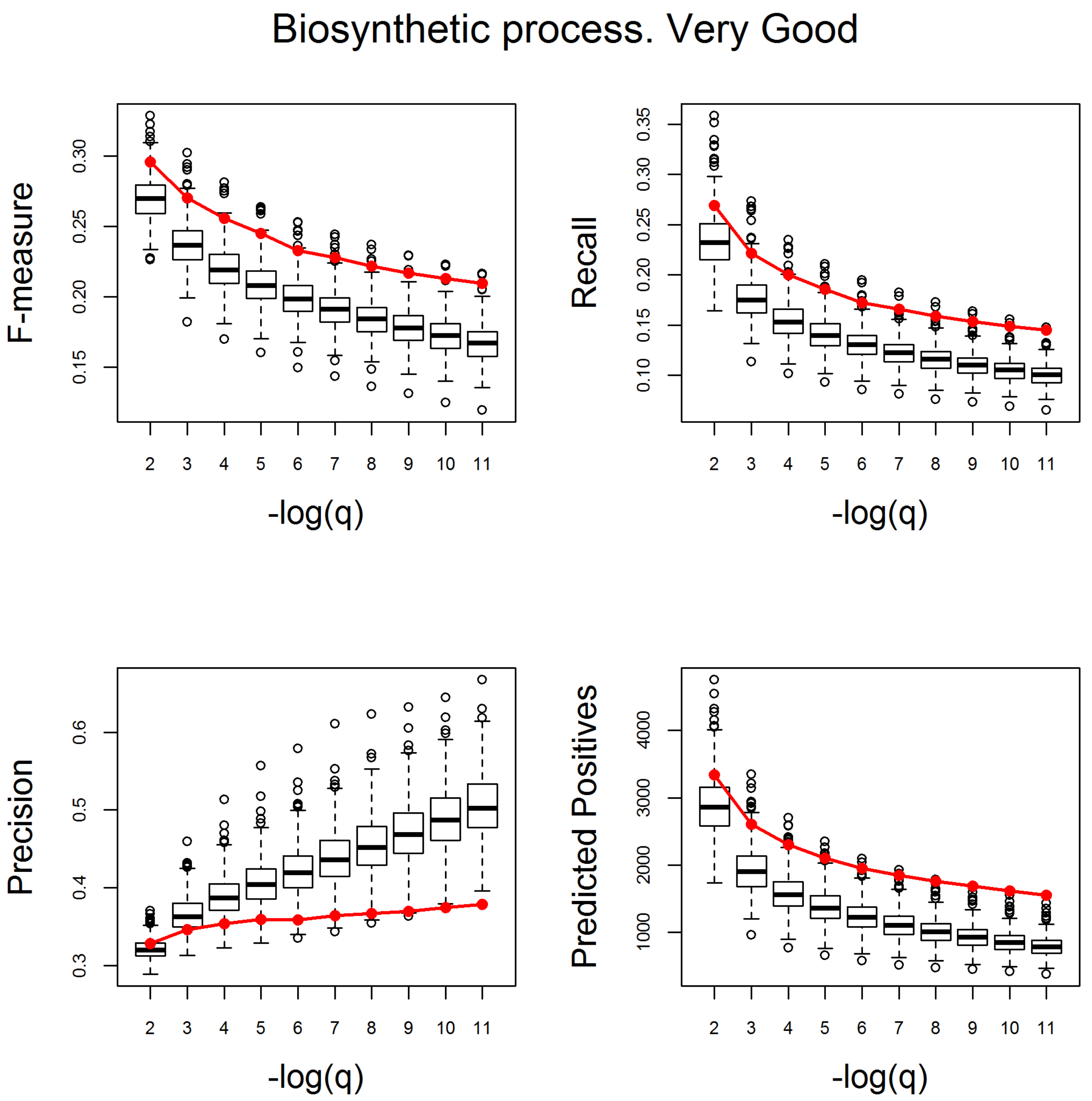

Appendix Figure S14. (continued)

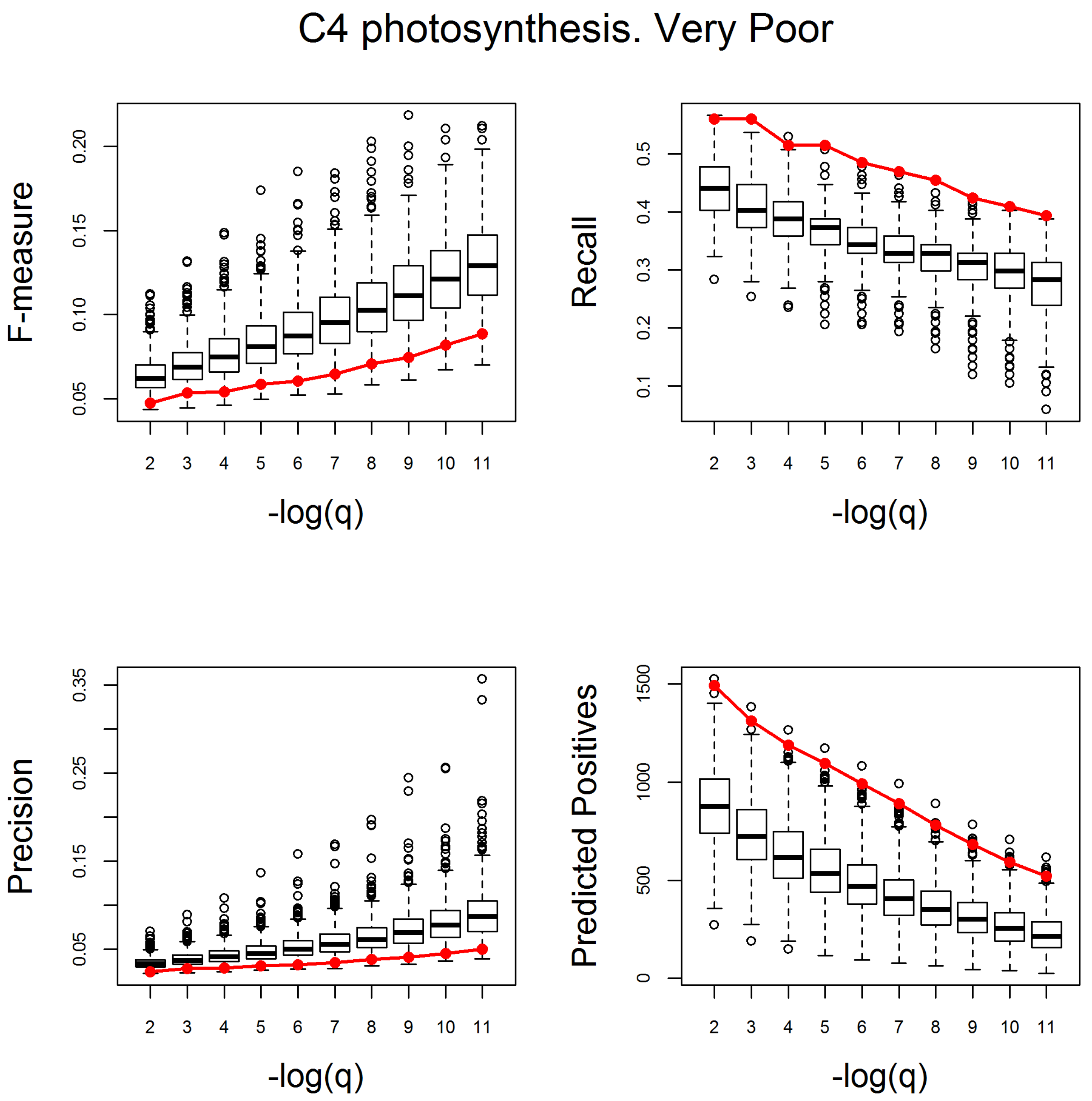

Appendix Figure S14. (continued)

Carbohydrate metabolic process. Good

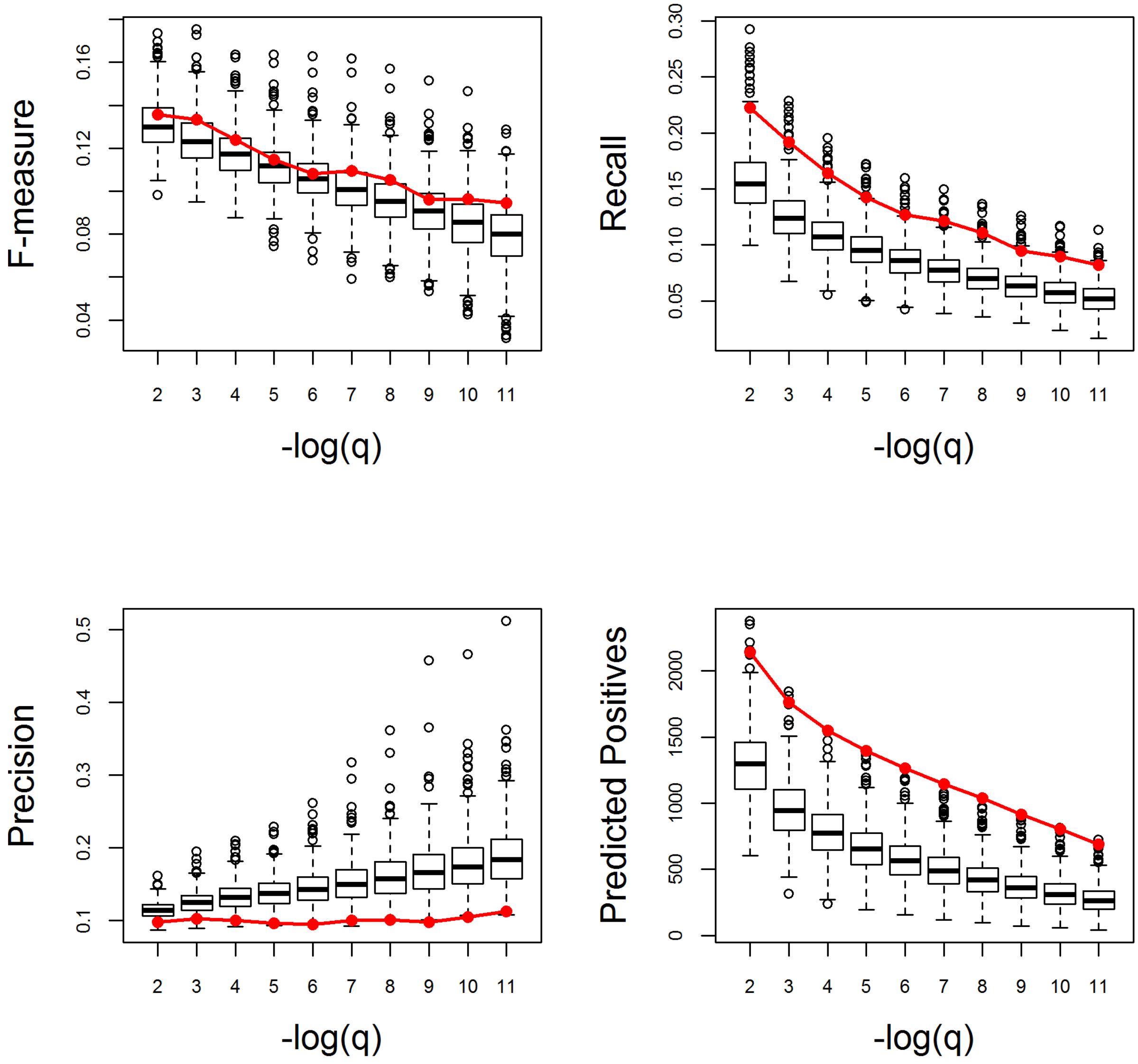

Appendix Figure S14. (continued)

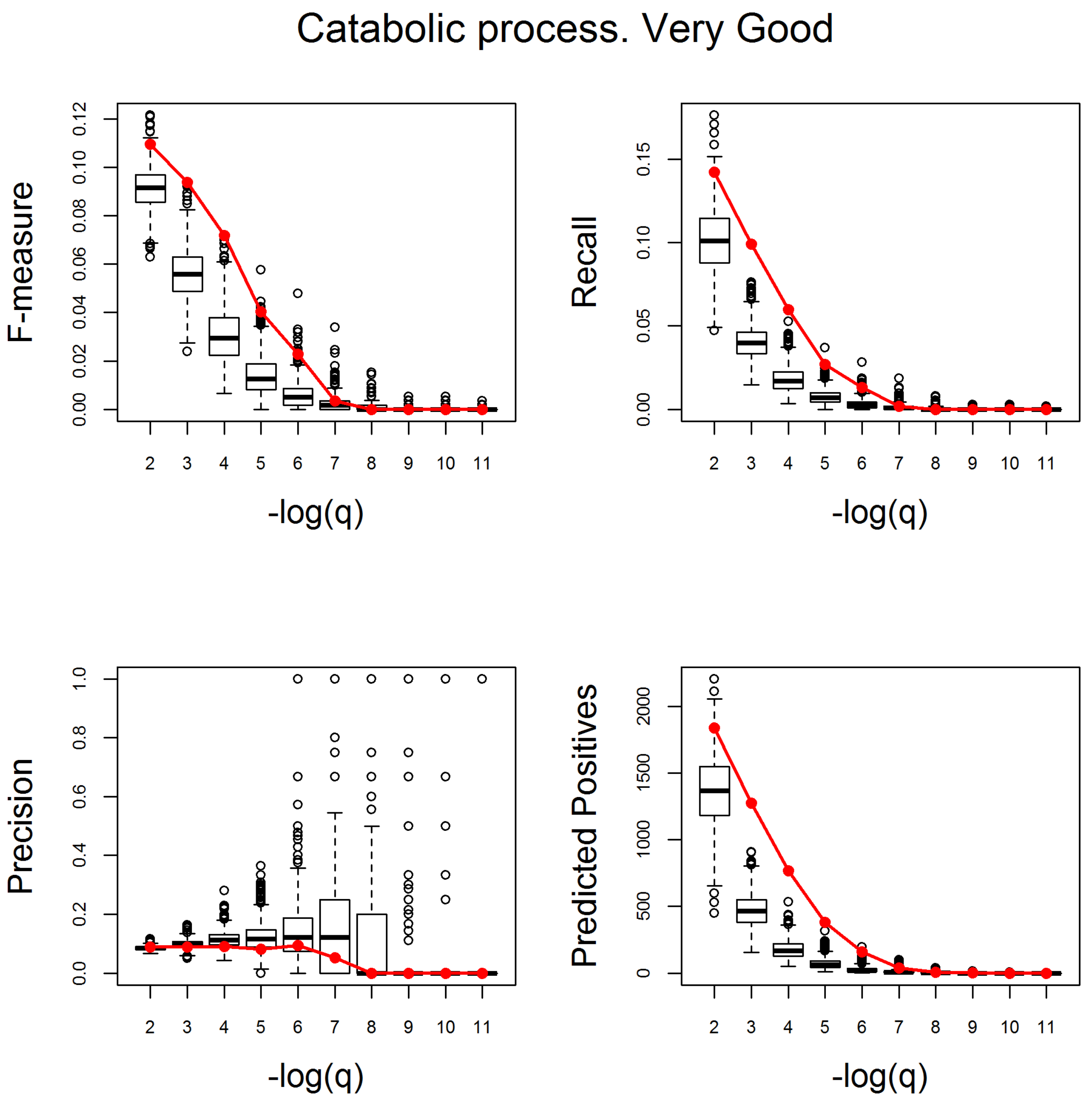

Appendix Figure S14. (continued)

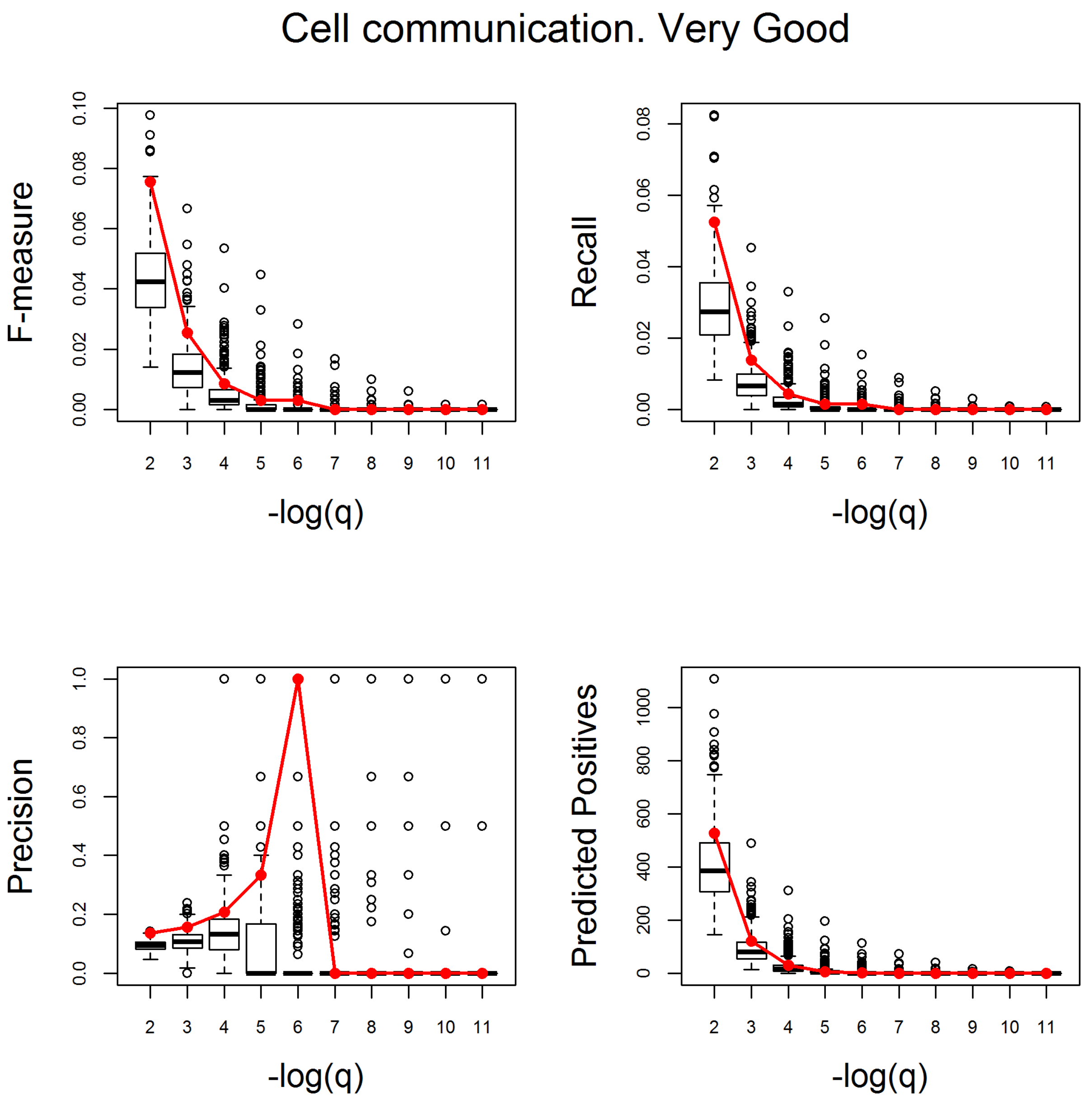

Appendix Figure S14. (continued)

Cell cycle. Very Poor

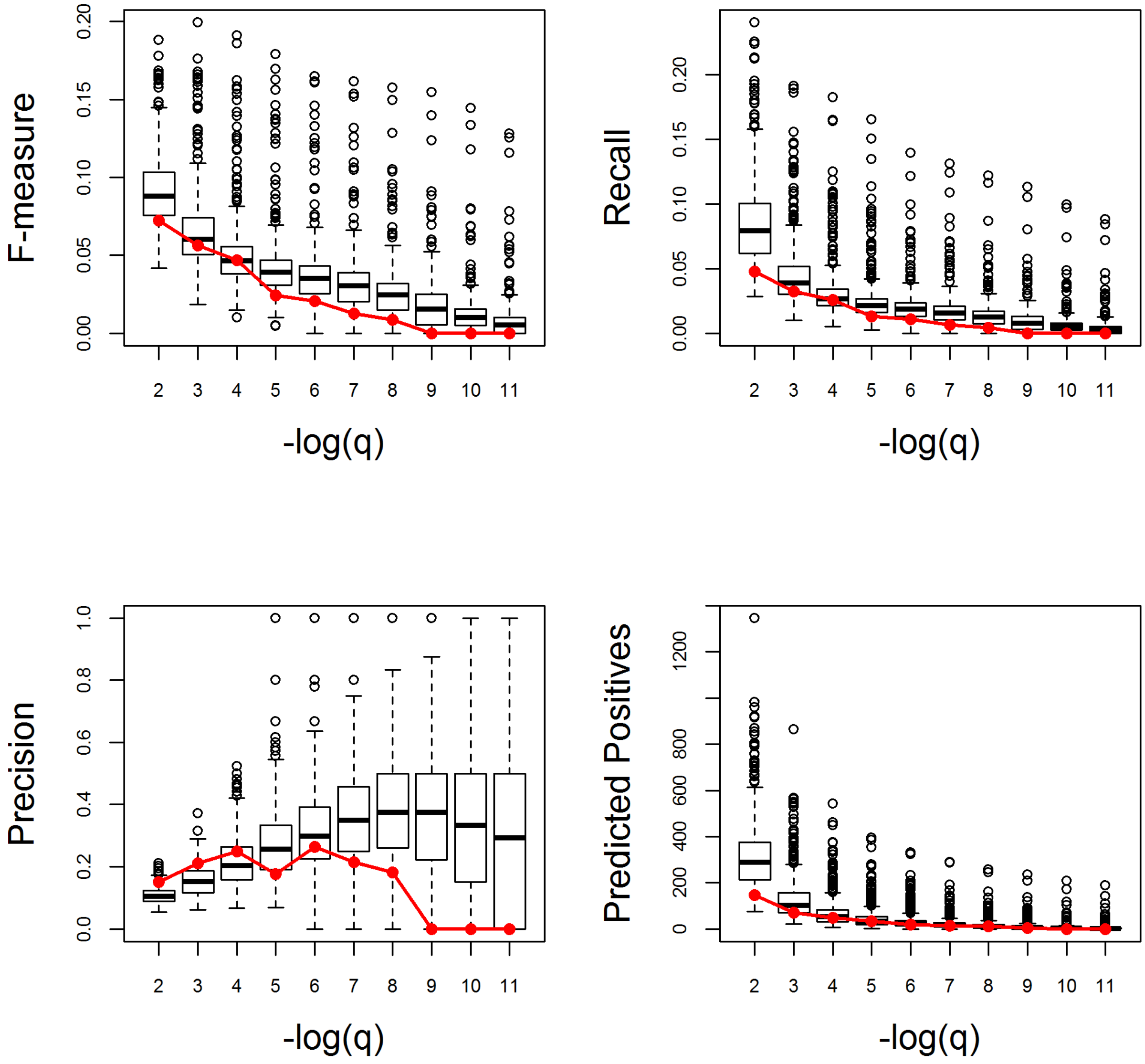

Appendix Figure S14. (continued)

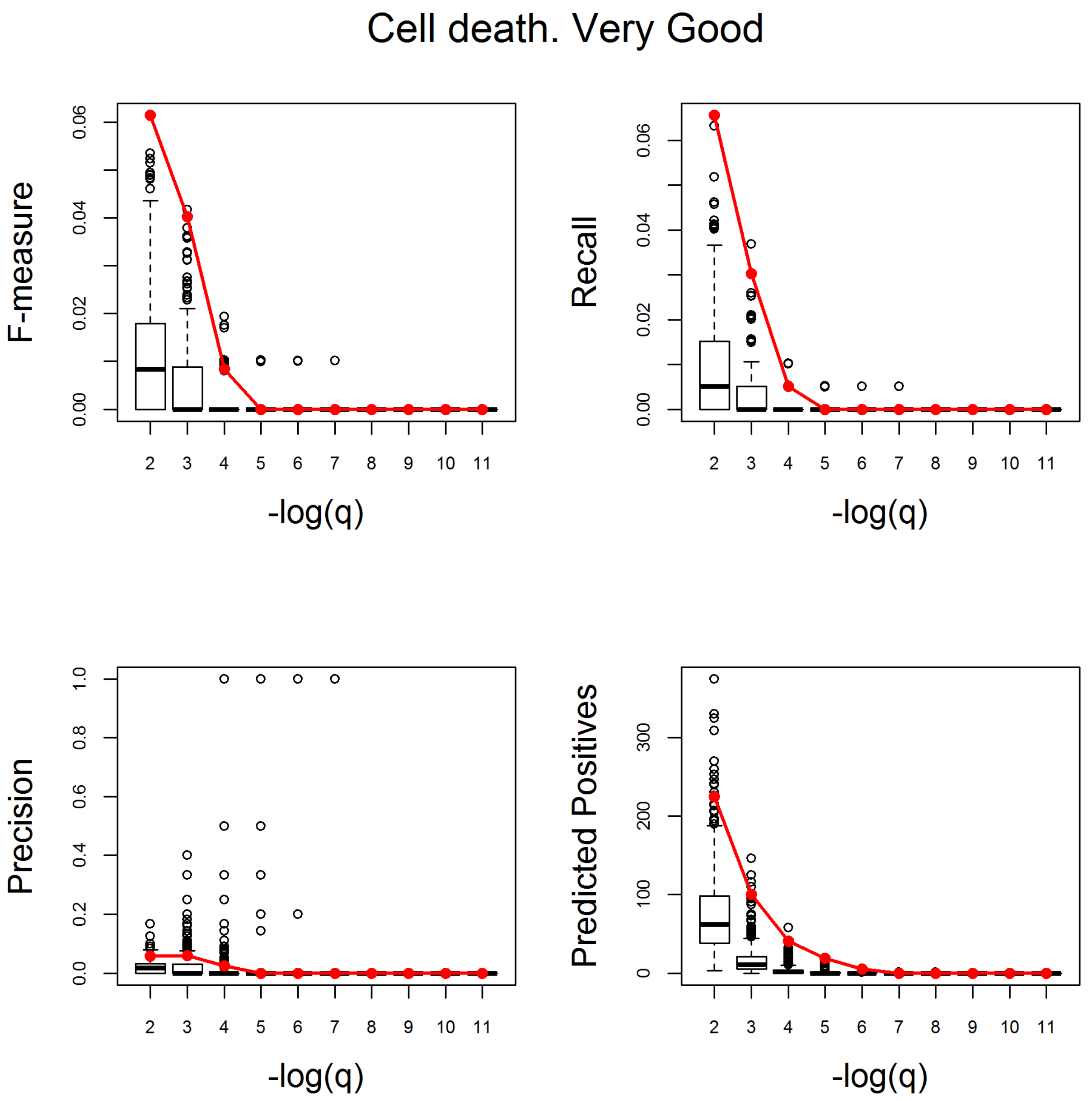

Appendix Figure S14. (continued)

Cell differentiation. Poor

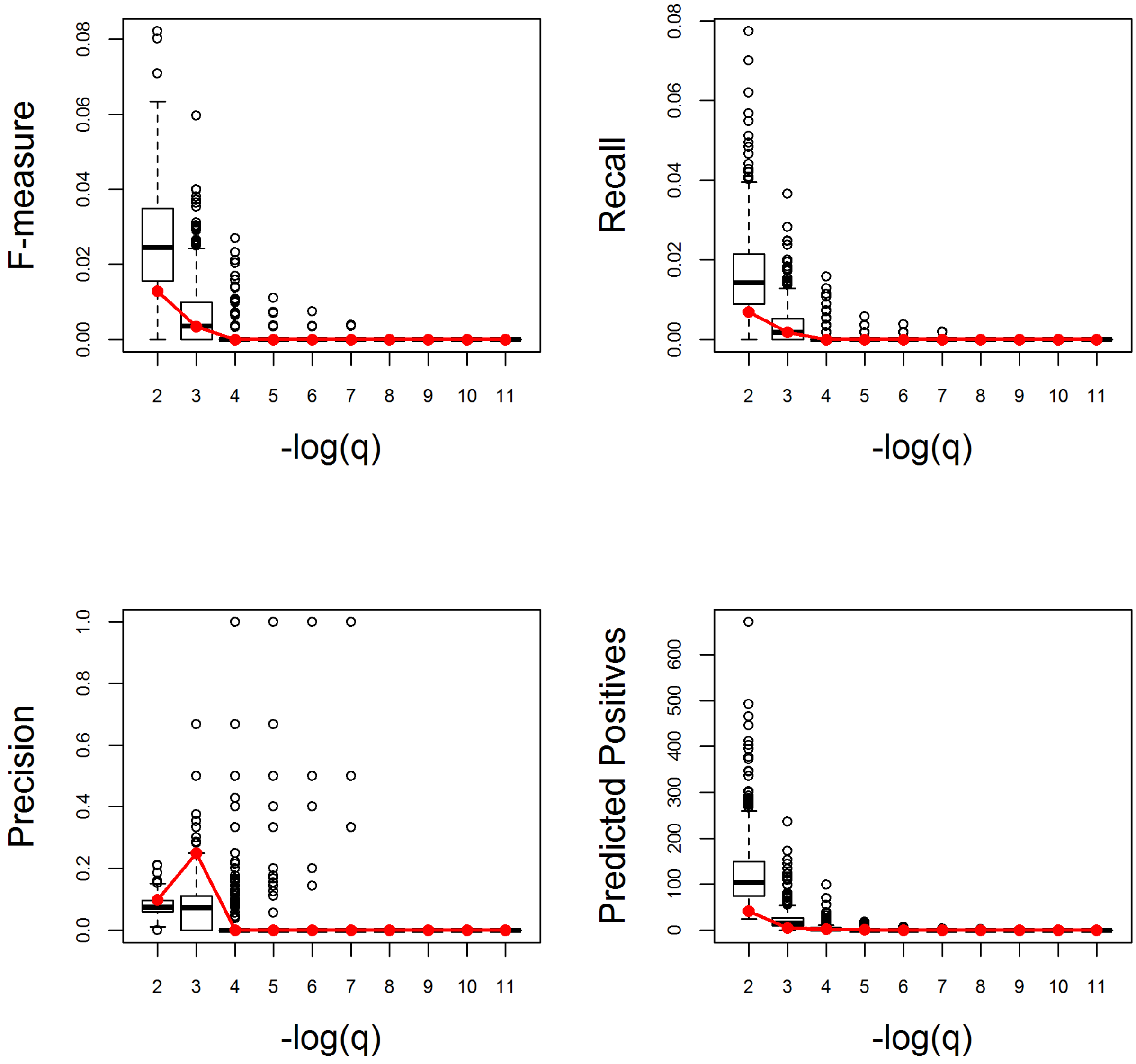

Appendix Figure S14. (continued)

Cell division. Very Poor

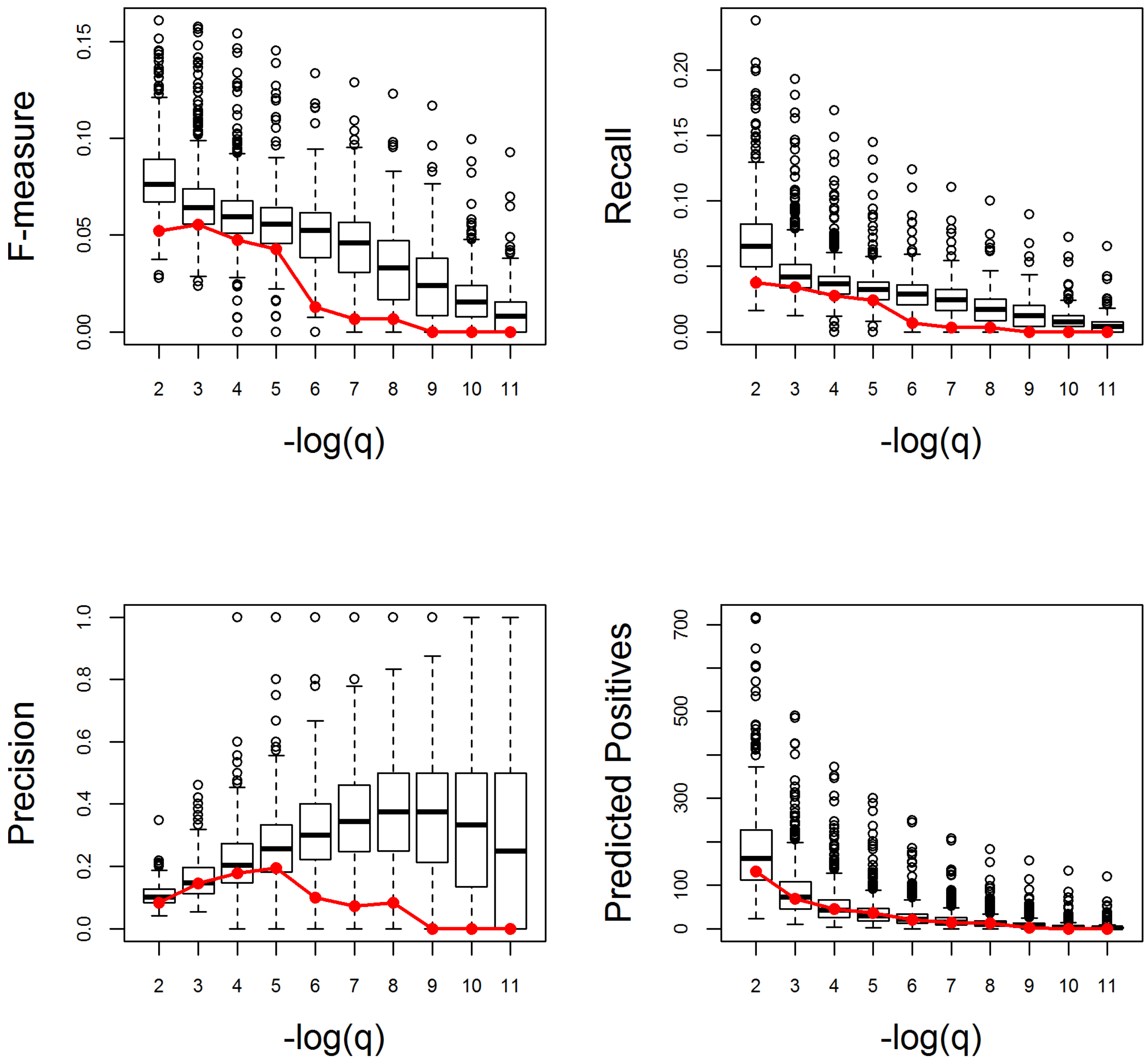

Appendix Figure S14. (continued)

Cell growth. Very Poor

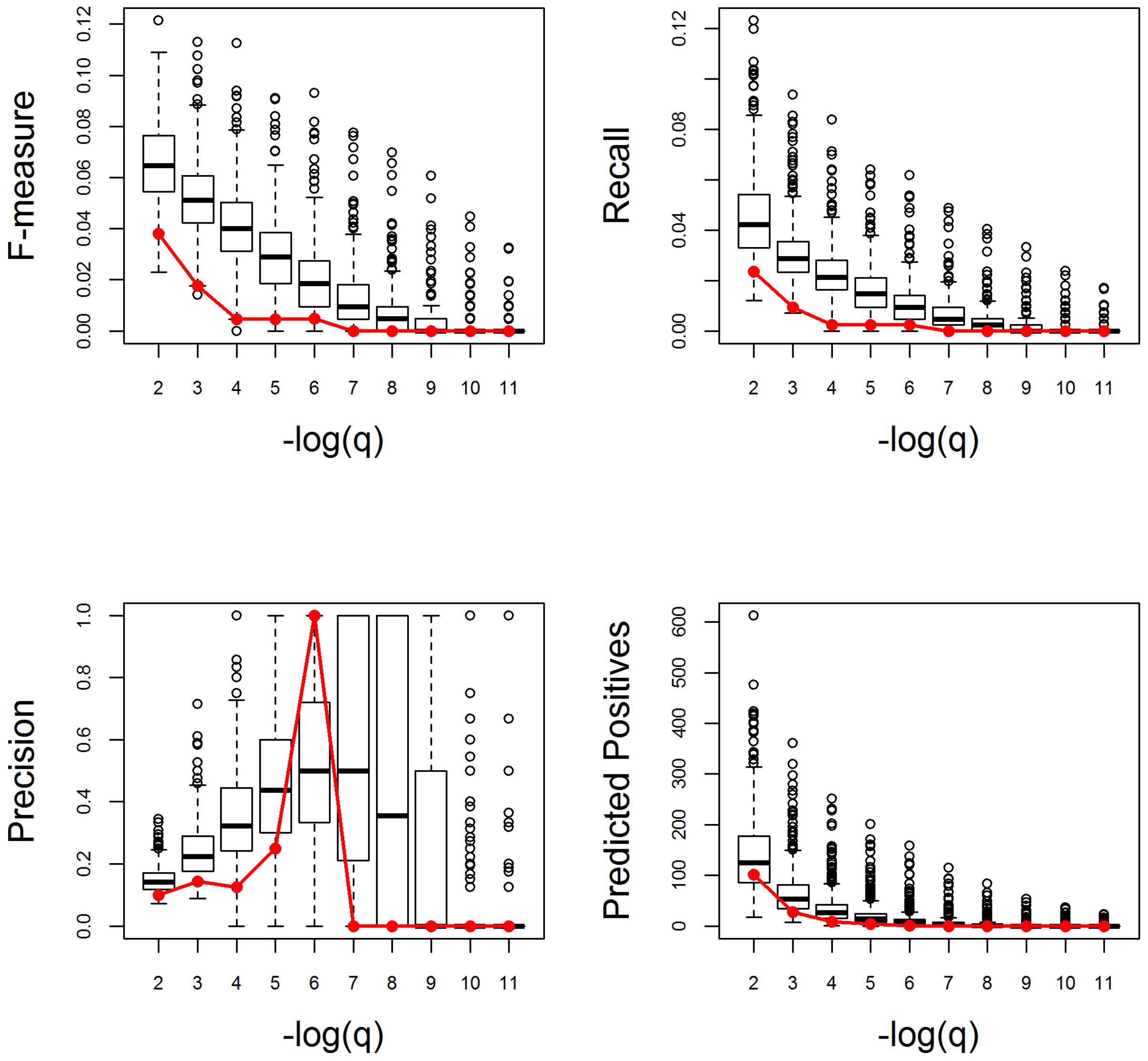

Appendix Figure S14. (continued)

Cell surface receptor signaling pathway. Average

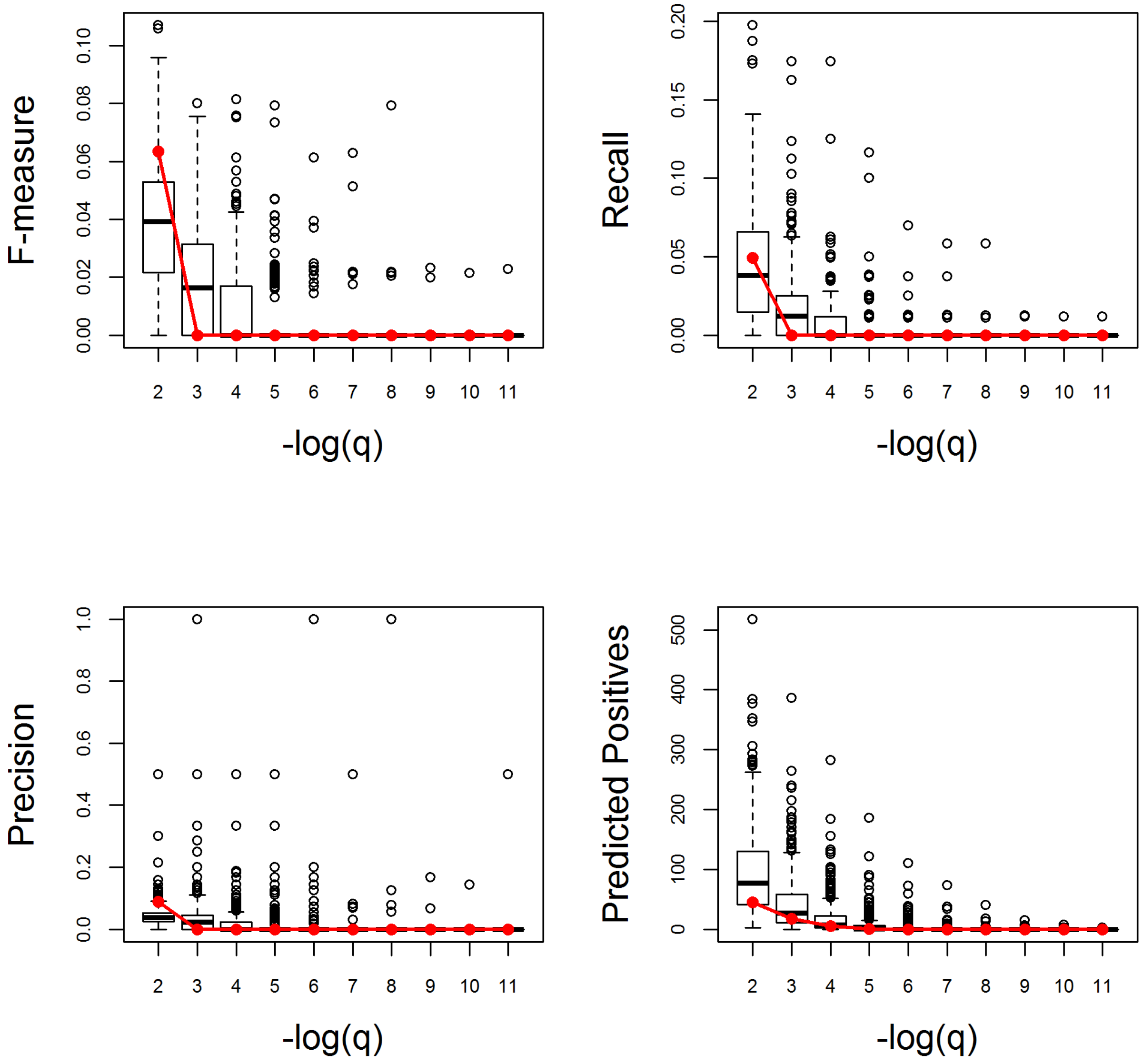

Appendix Figure S14. (continued)

Cell wall macromolecule metabolic process. Very Good

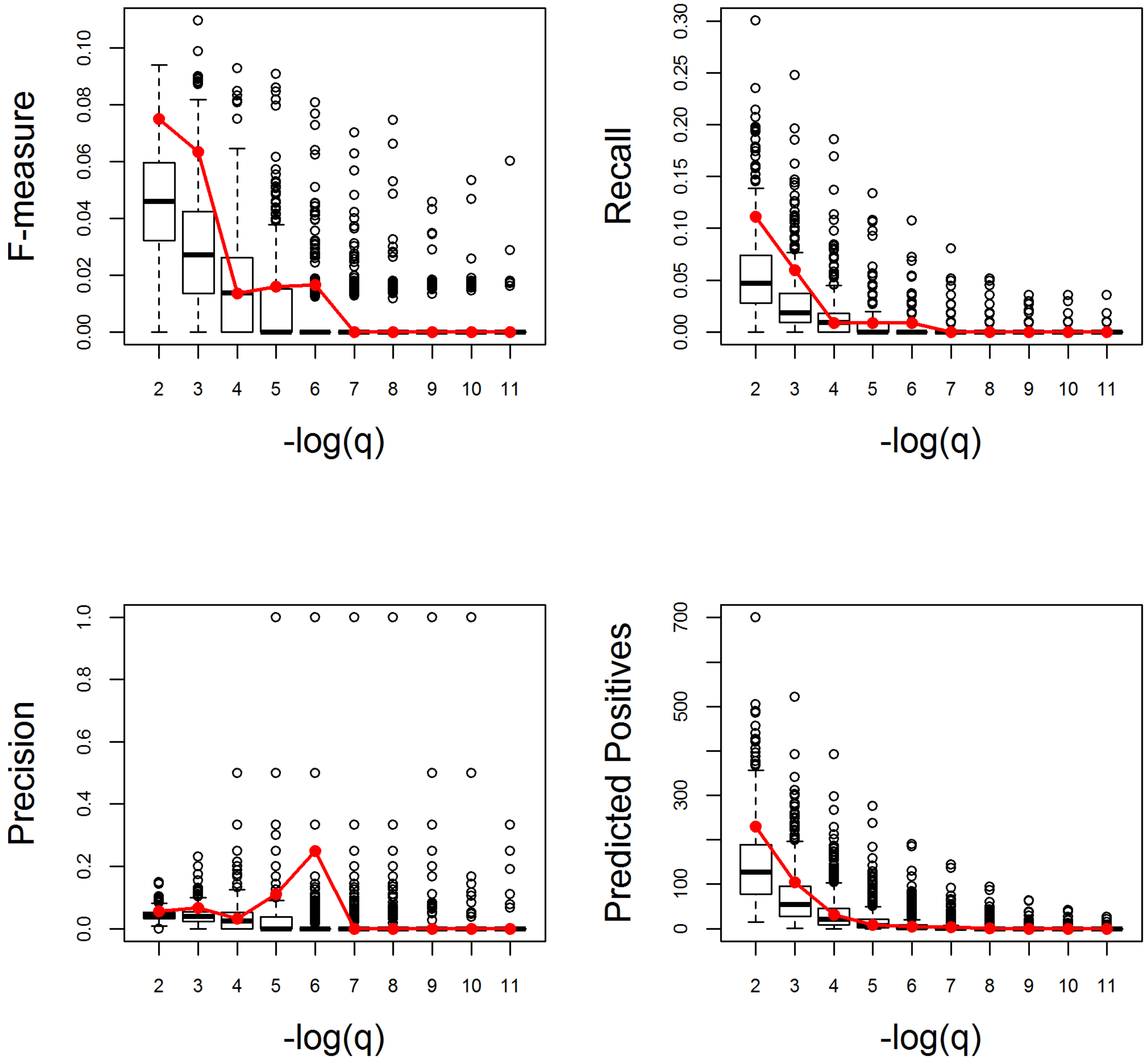

Appendix Figure S14. (continued)

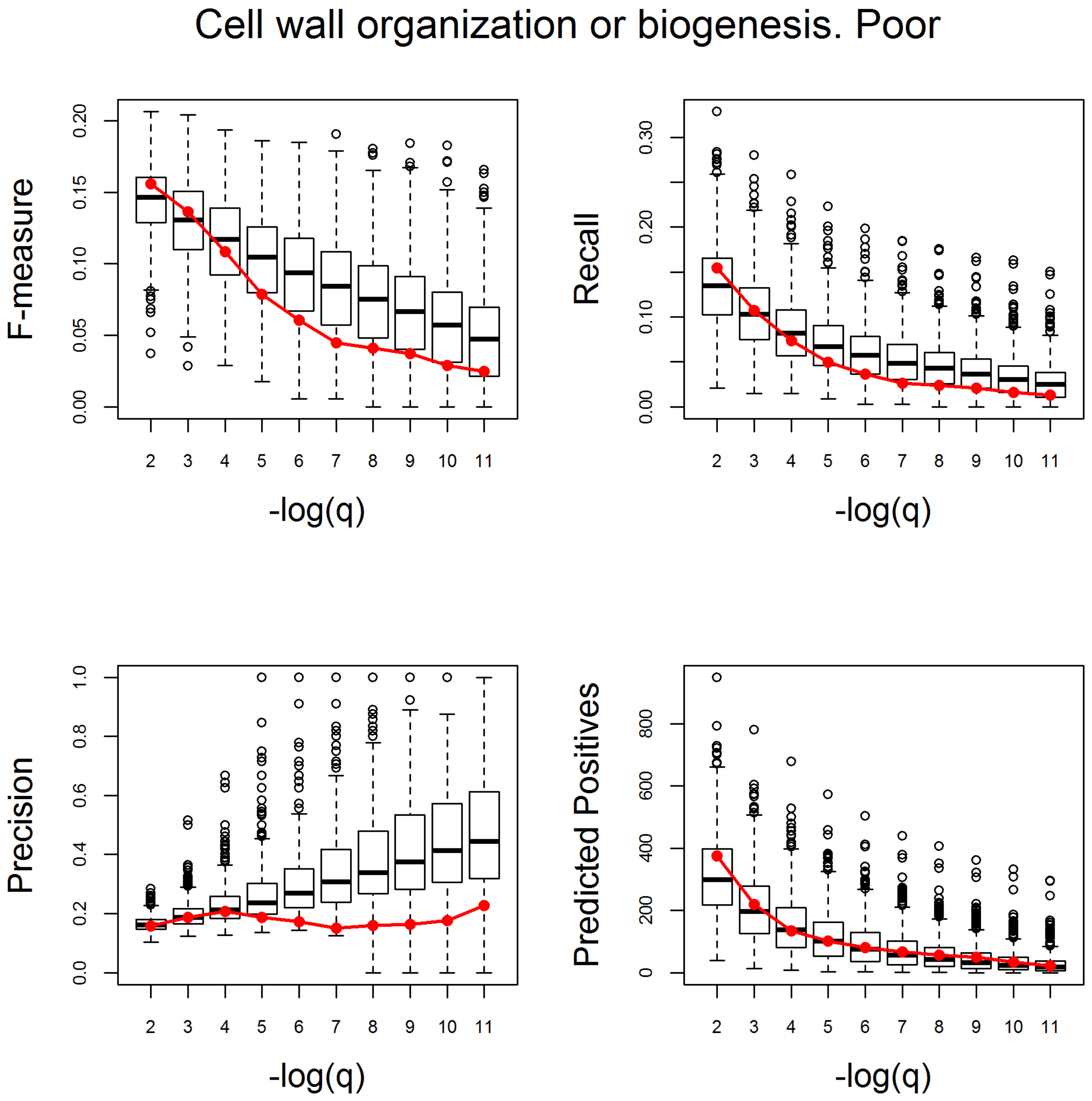

Appendix Figure S14. (continued)

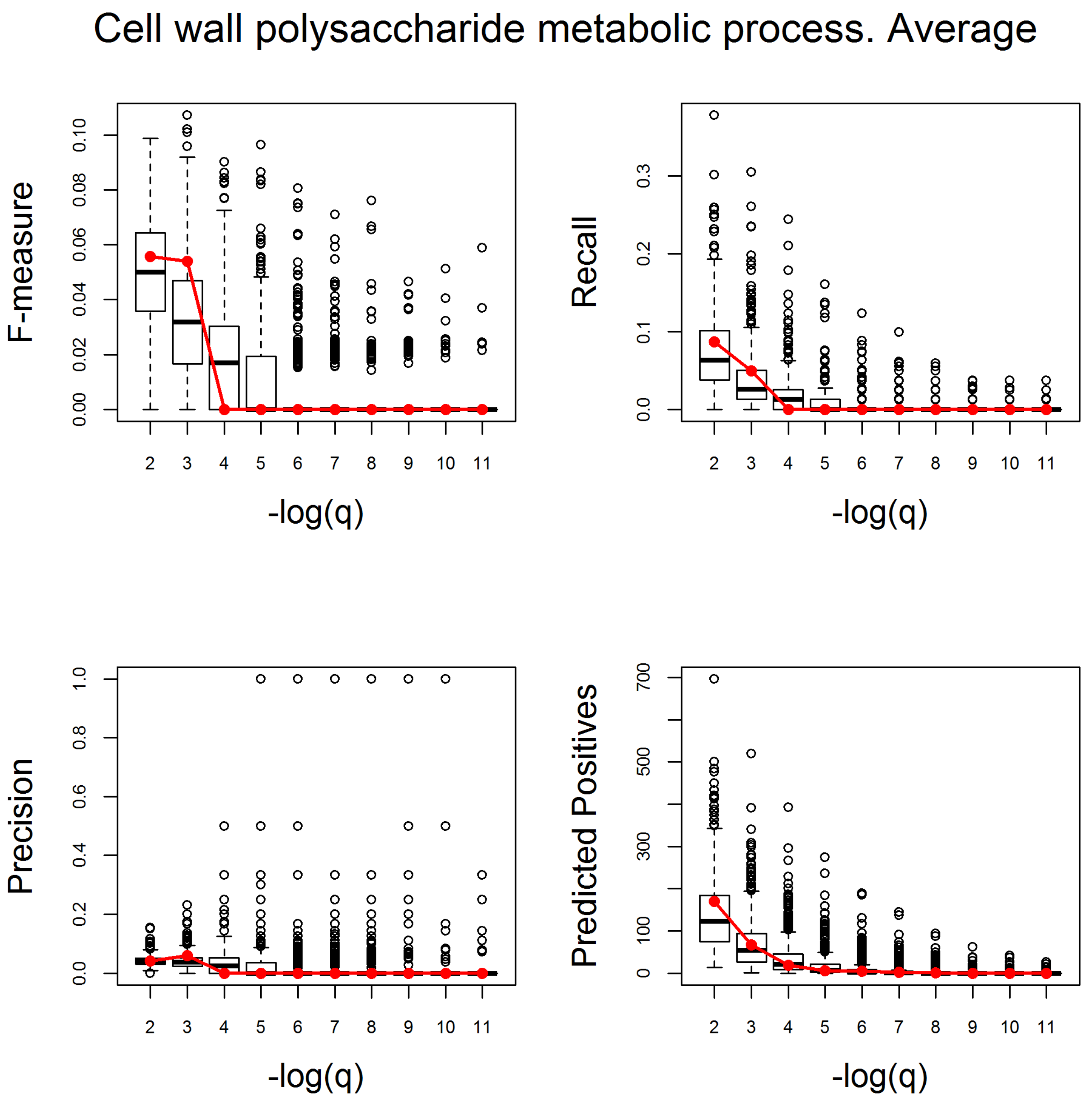

Appendix Figure S14. (continued)

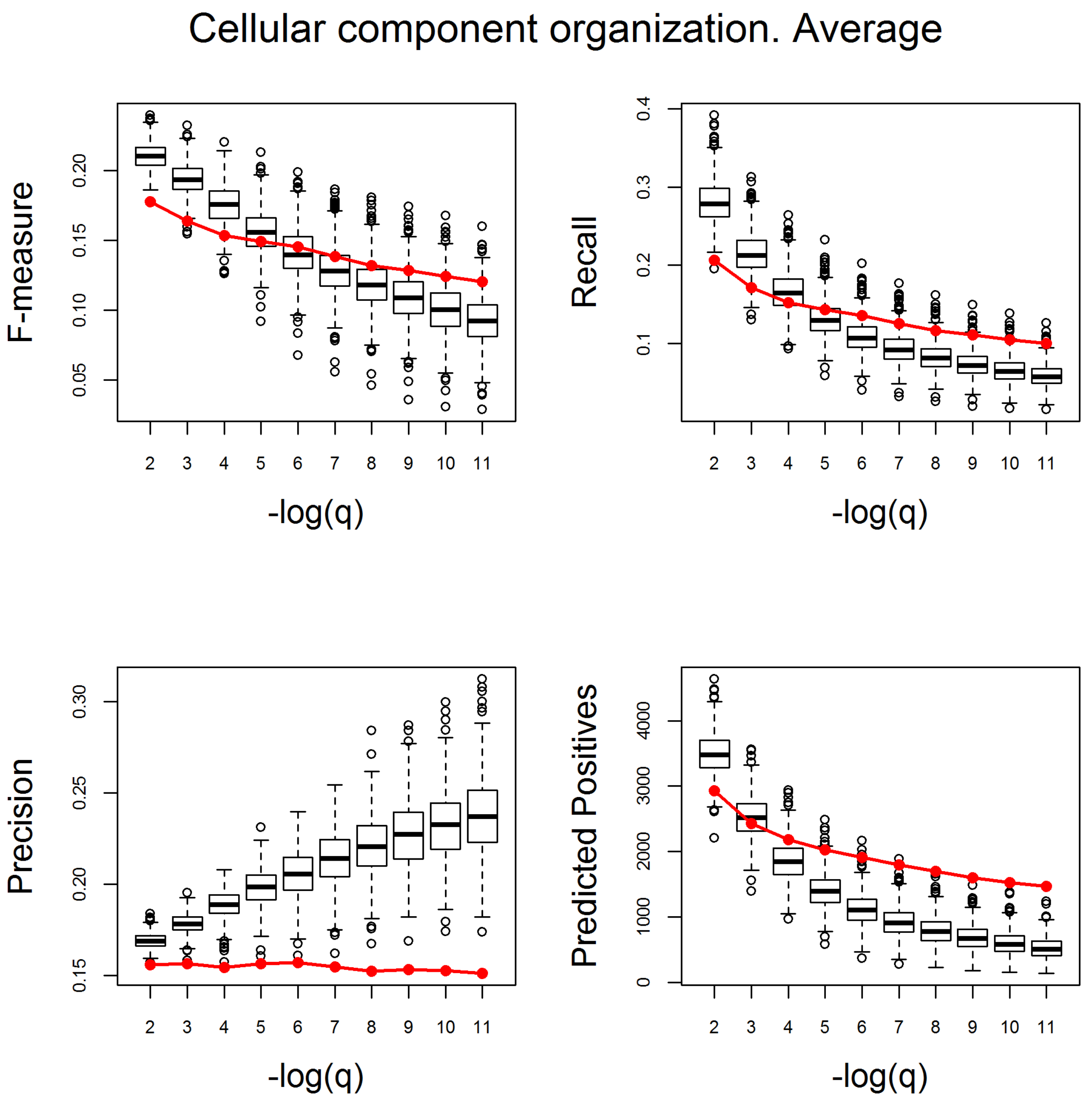

Appendix Figure S14. (continued)

Cellular homeostasis. Very Good

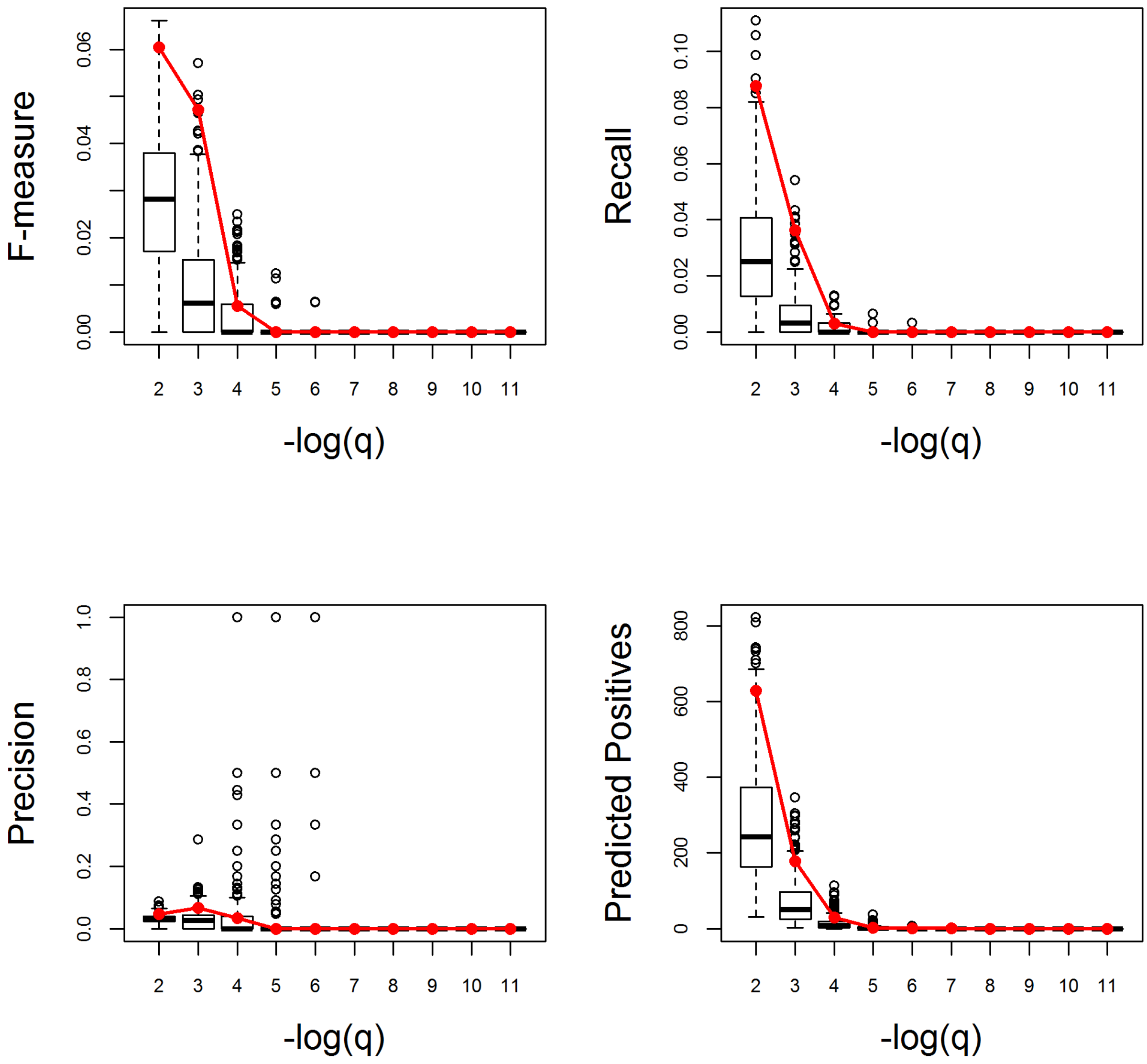

Appendix Figure S14. (continued)

Cellular protein modification process. Very Poor

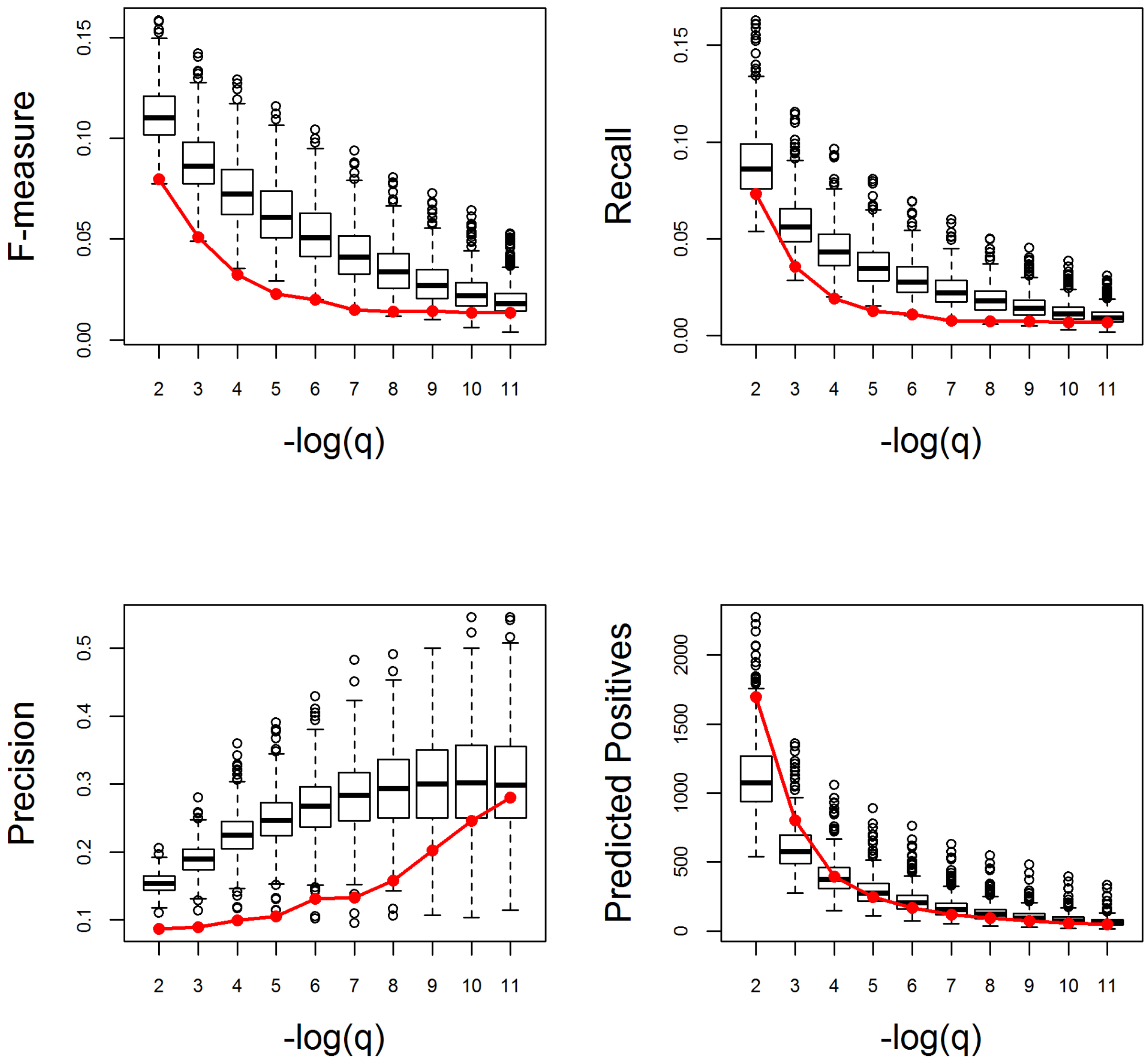

Appendix Figure S14. (continued)

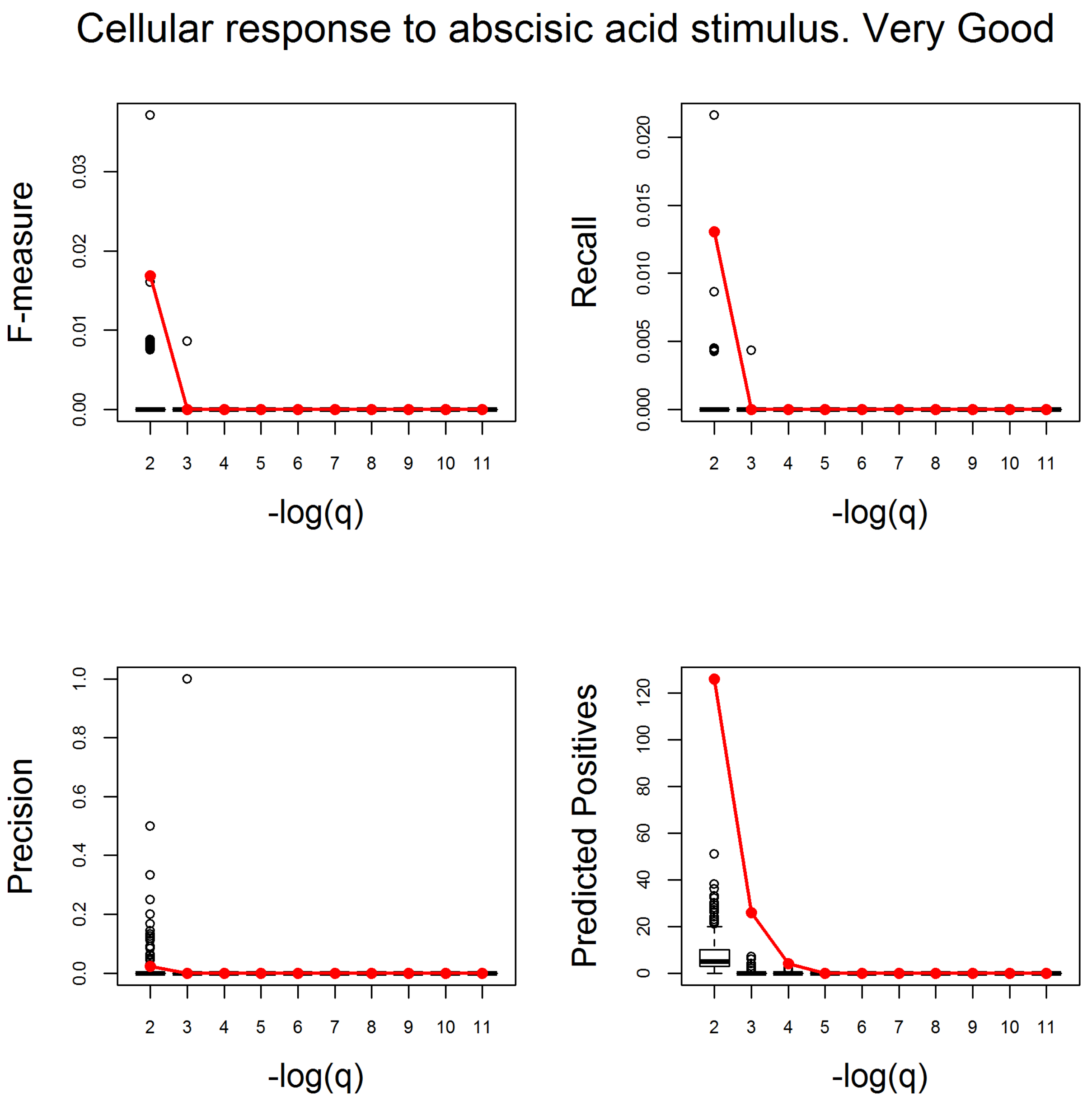

Appendix Figure S14. (continued)

Cellular response to alcohol. Very Good

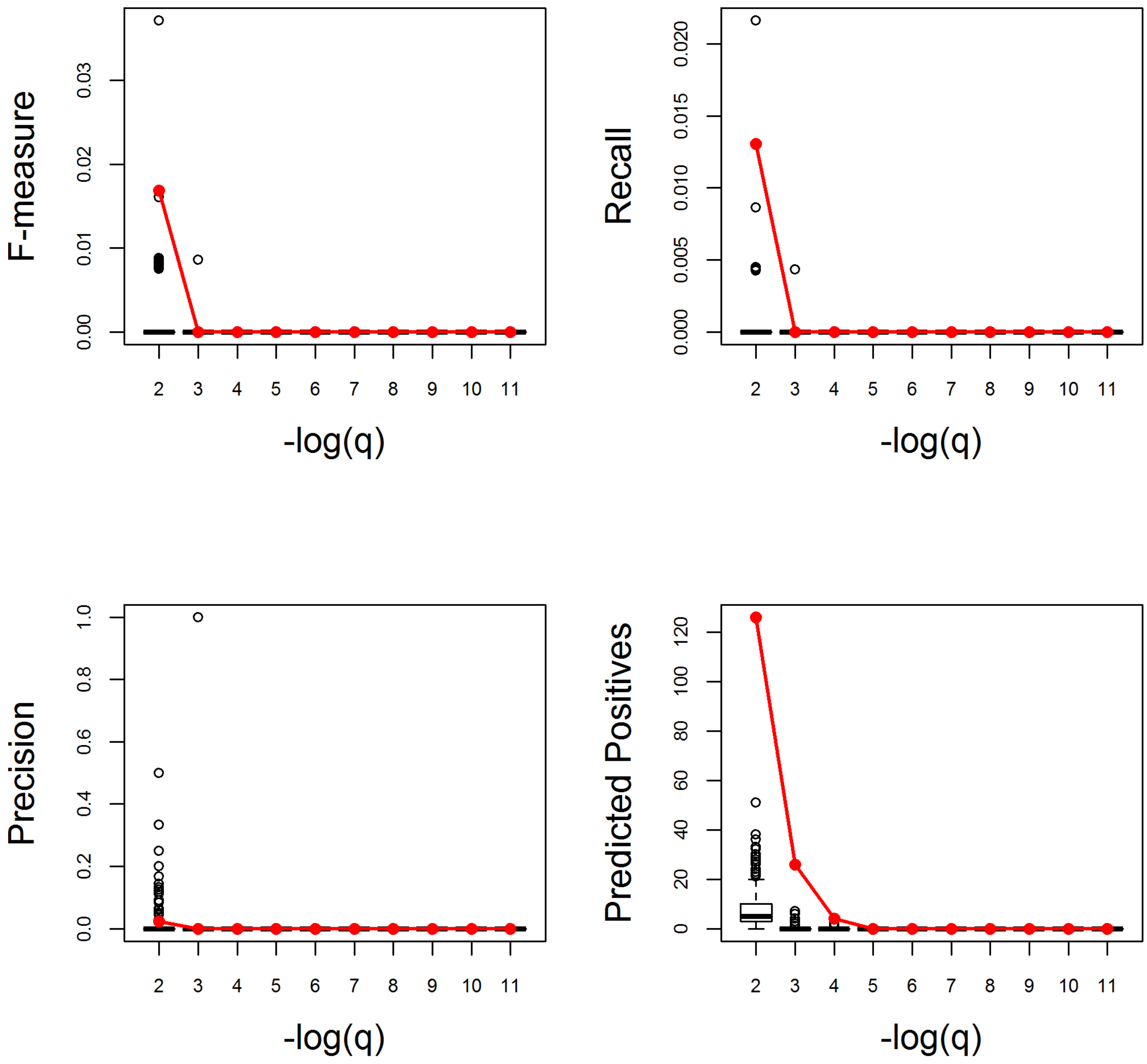

Appendix Figure S14. (continued)

Cellular response to DNA damage stimulus. Very Poor

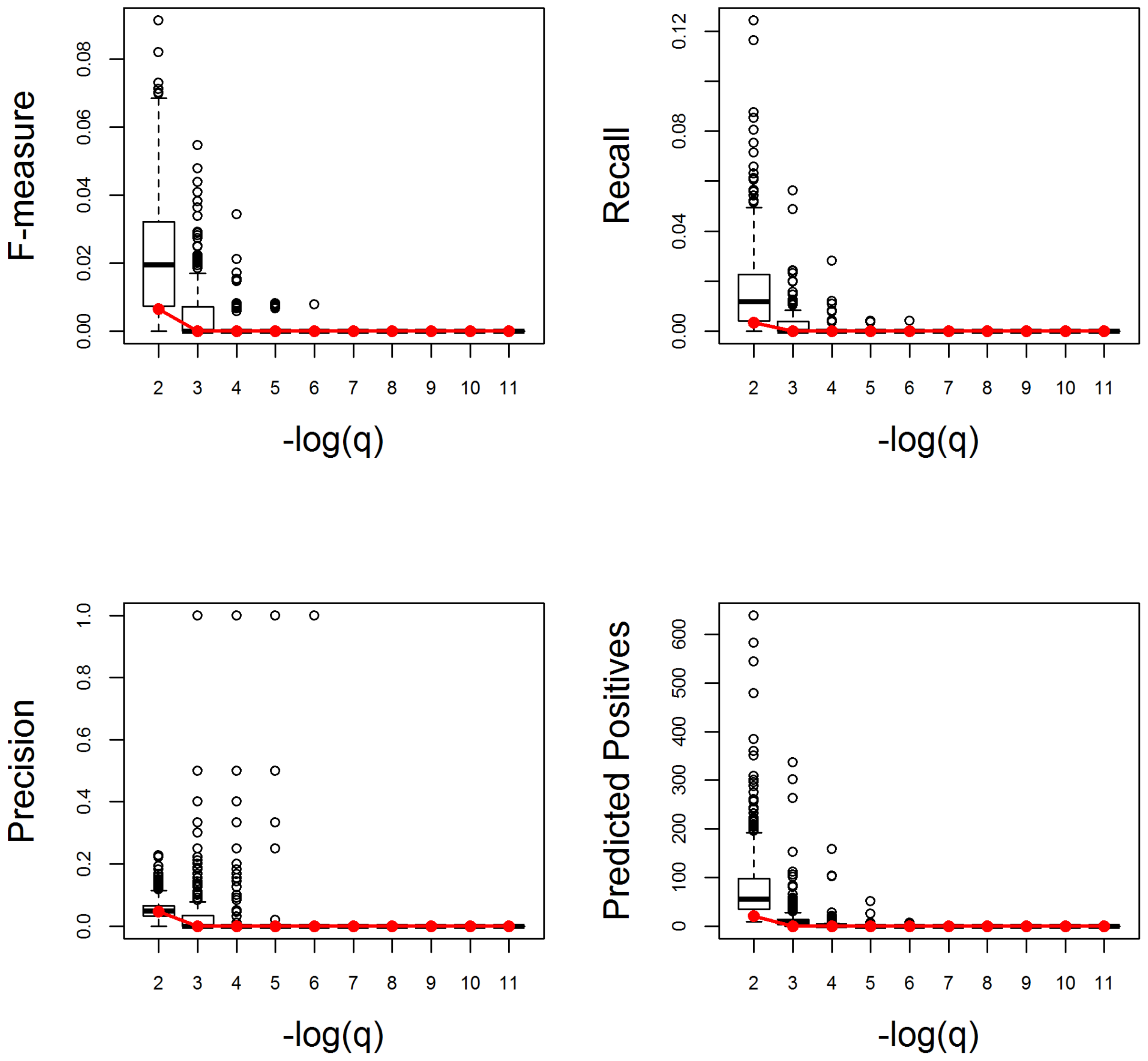

Appendix Figure S14. (continued)

Cellulose metabolic process. Very Poor

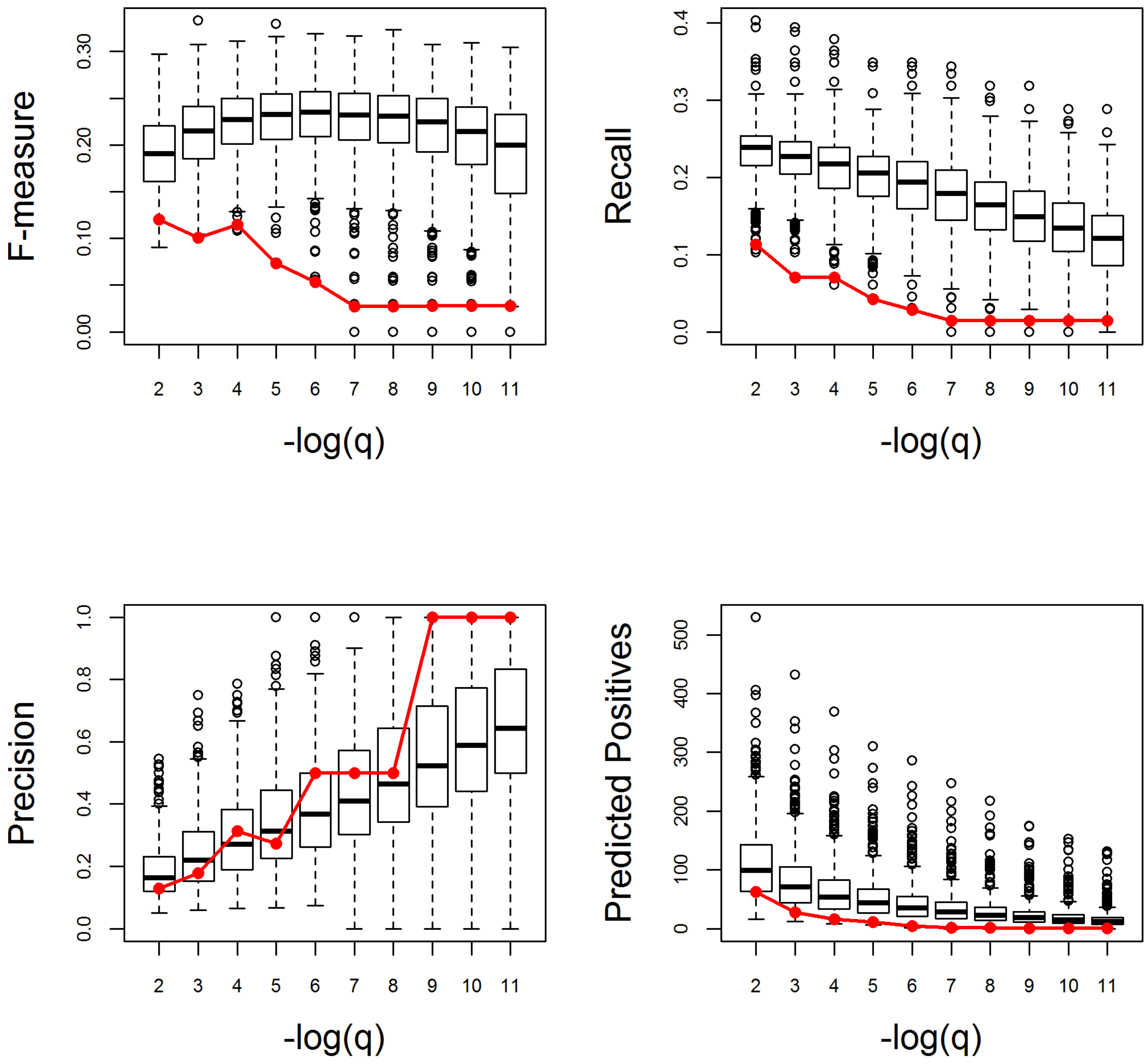

Appendix Figure S14. (continued)

Cofactor metabolic process. Very Good

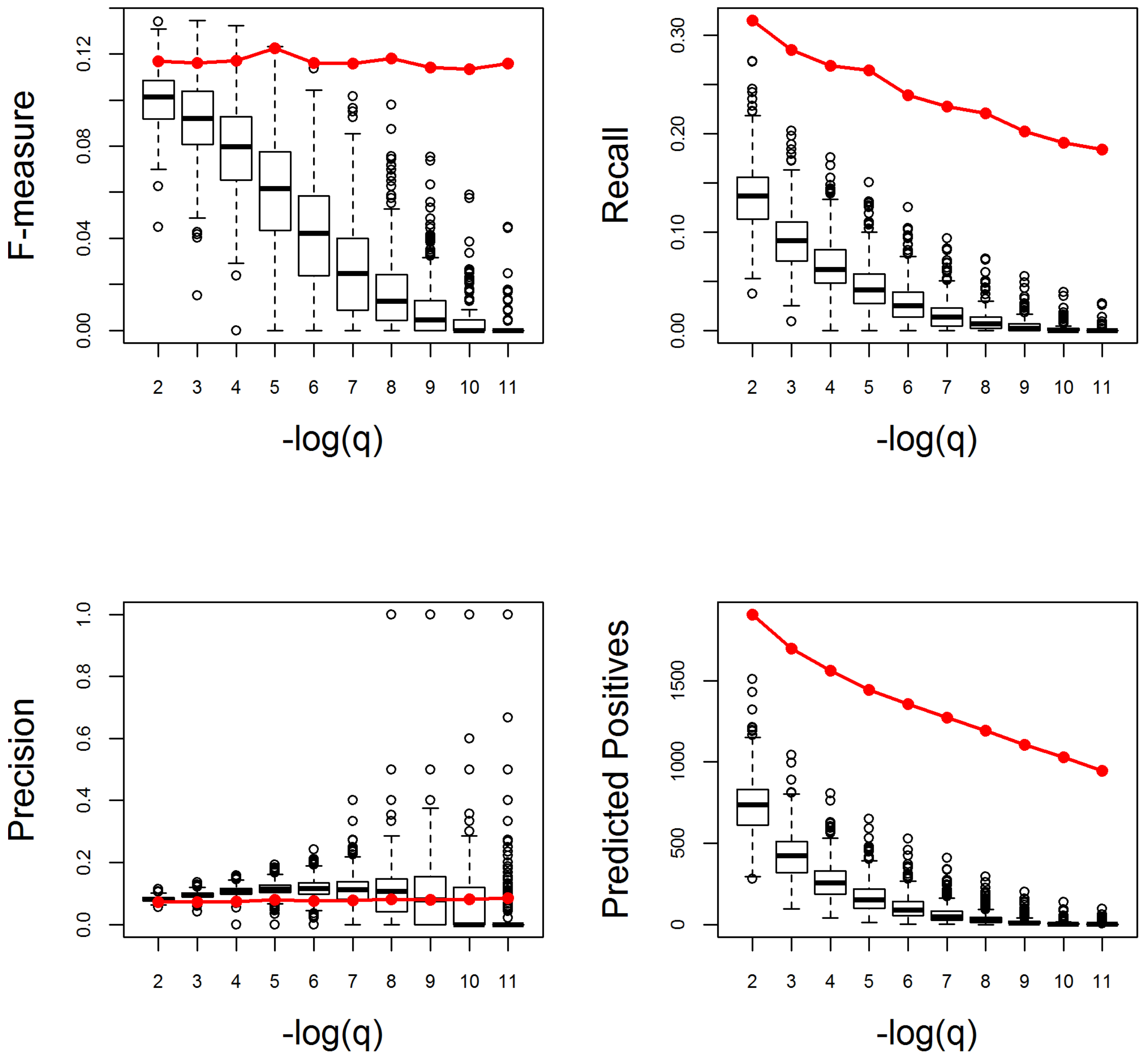

Appendix Figure S14. (continued)

Defense response to bacterium. Very Good

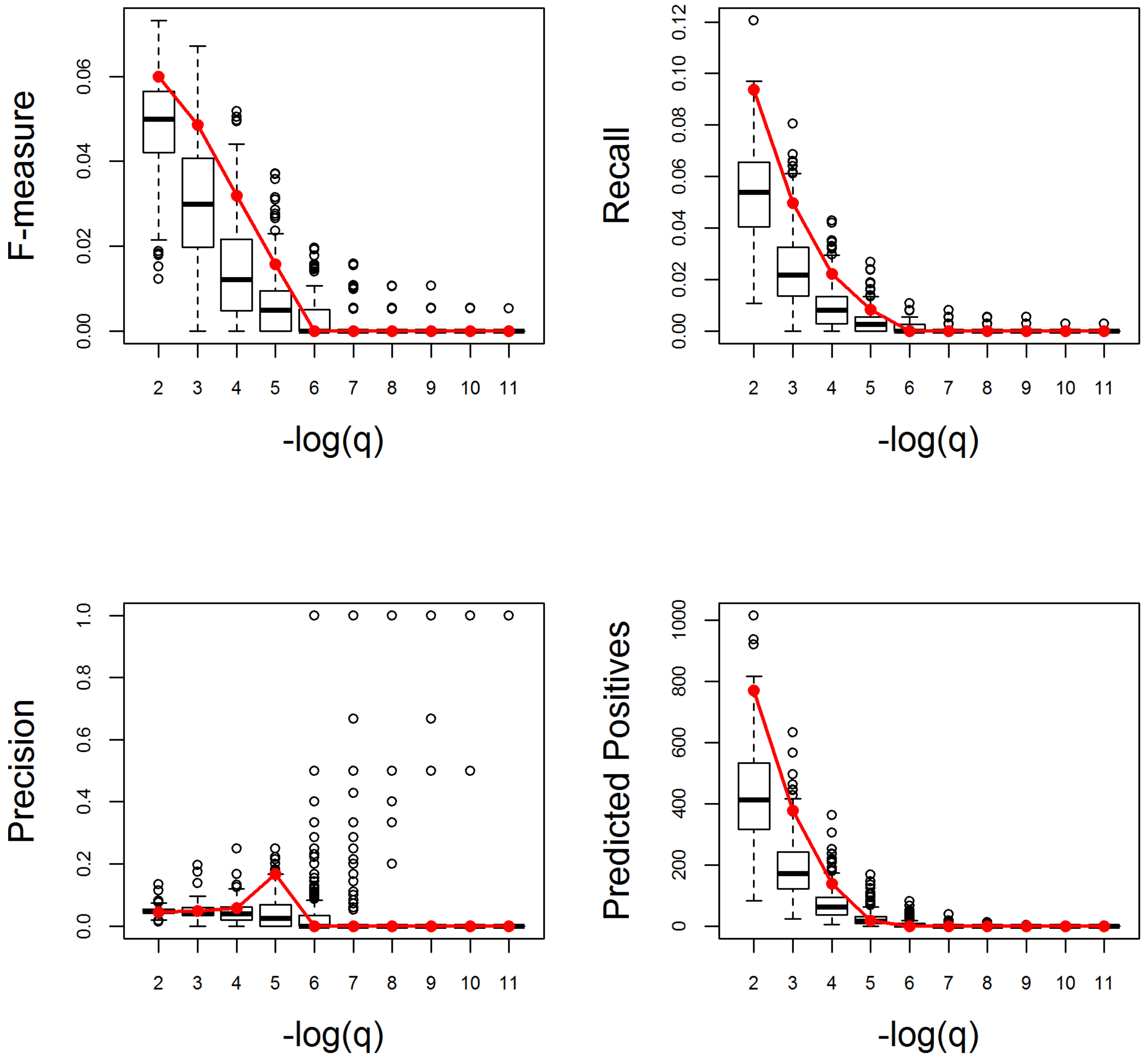

Appendix Figure S14. (continued)

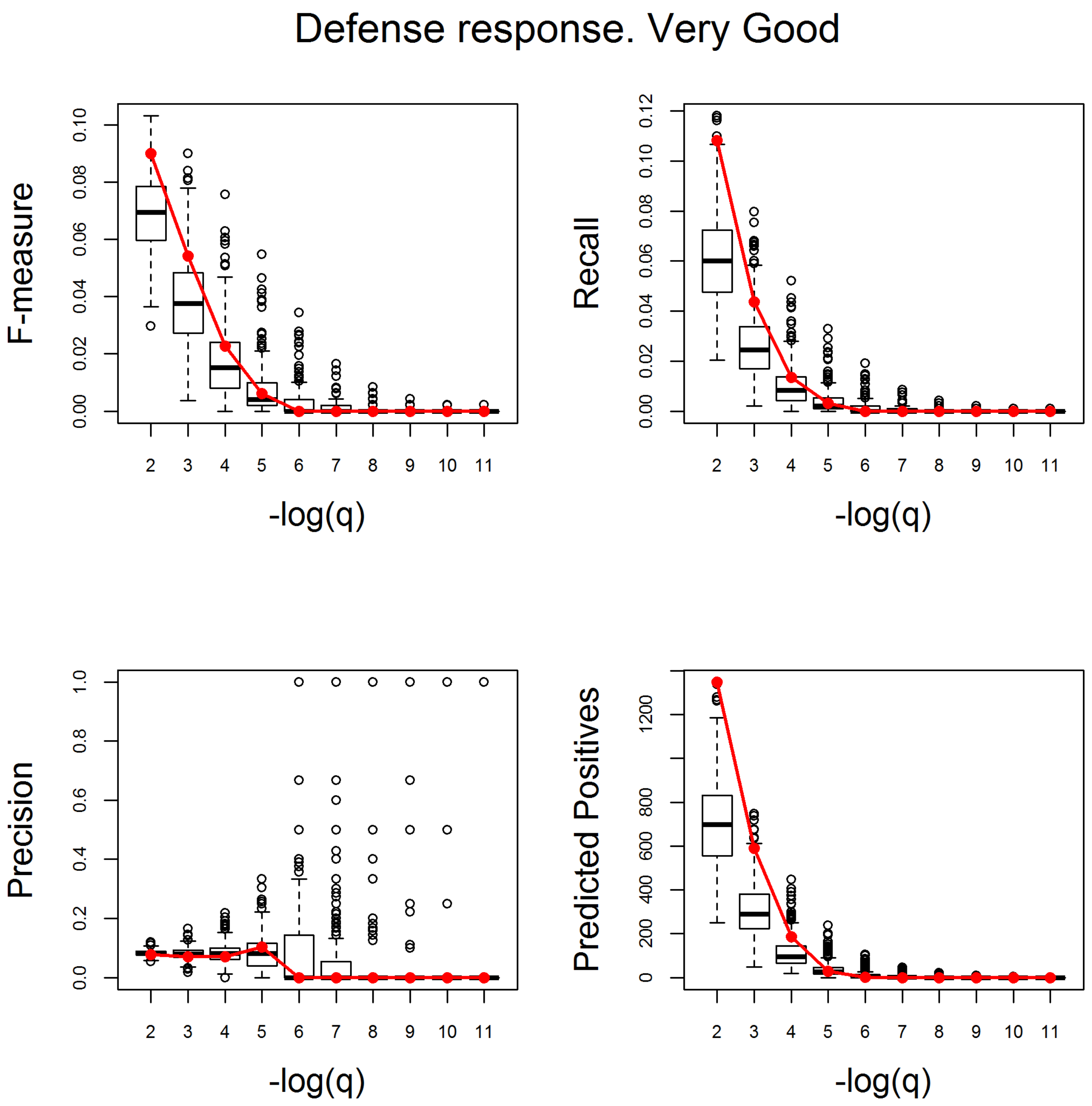

Appendix Figure S14. (continued)

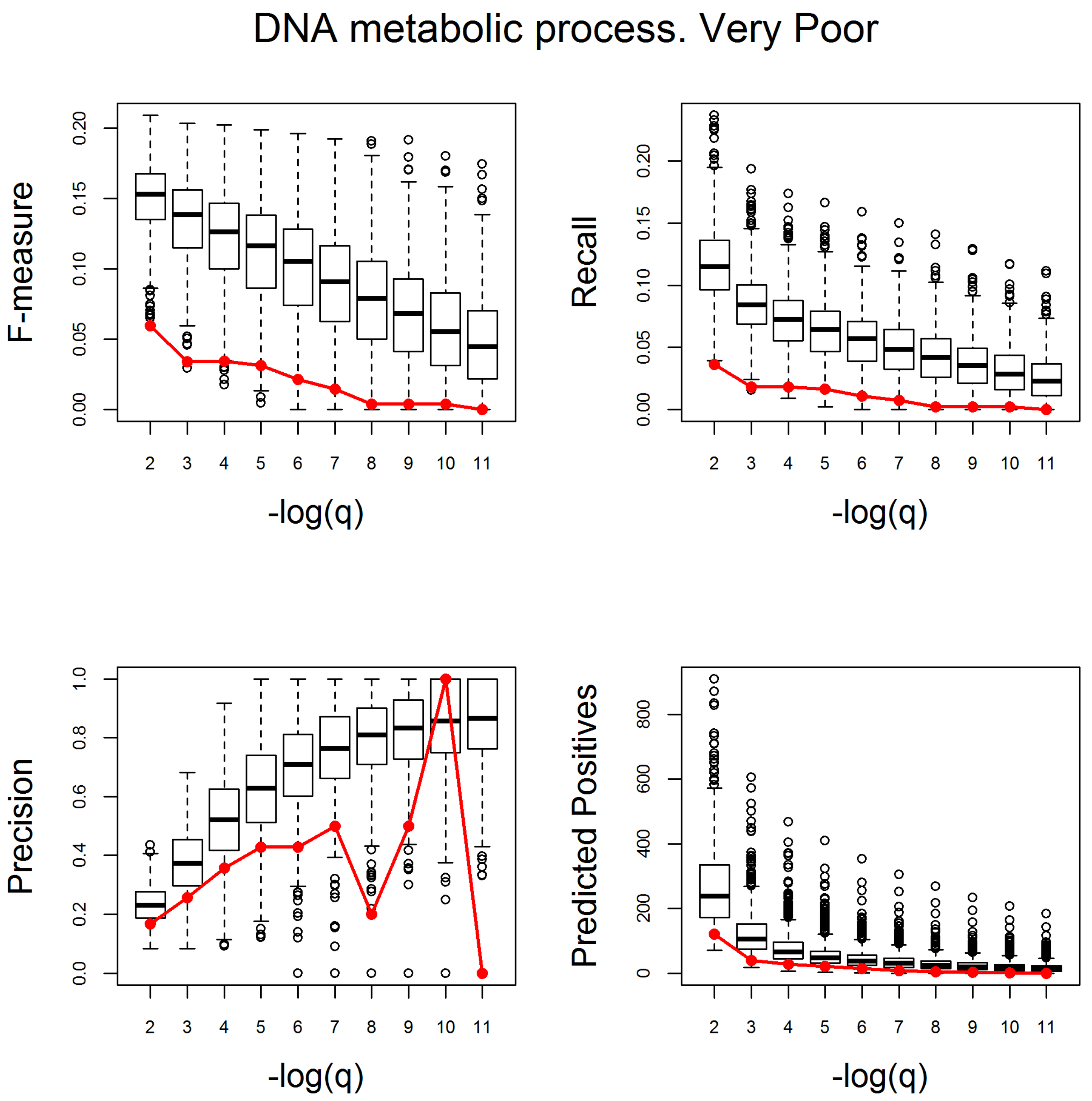

Appendix Figure S14. (continued)

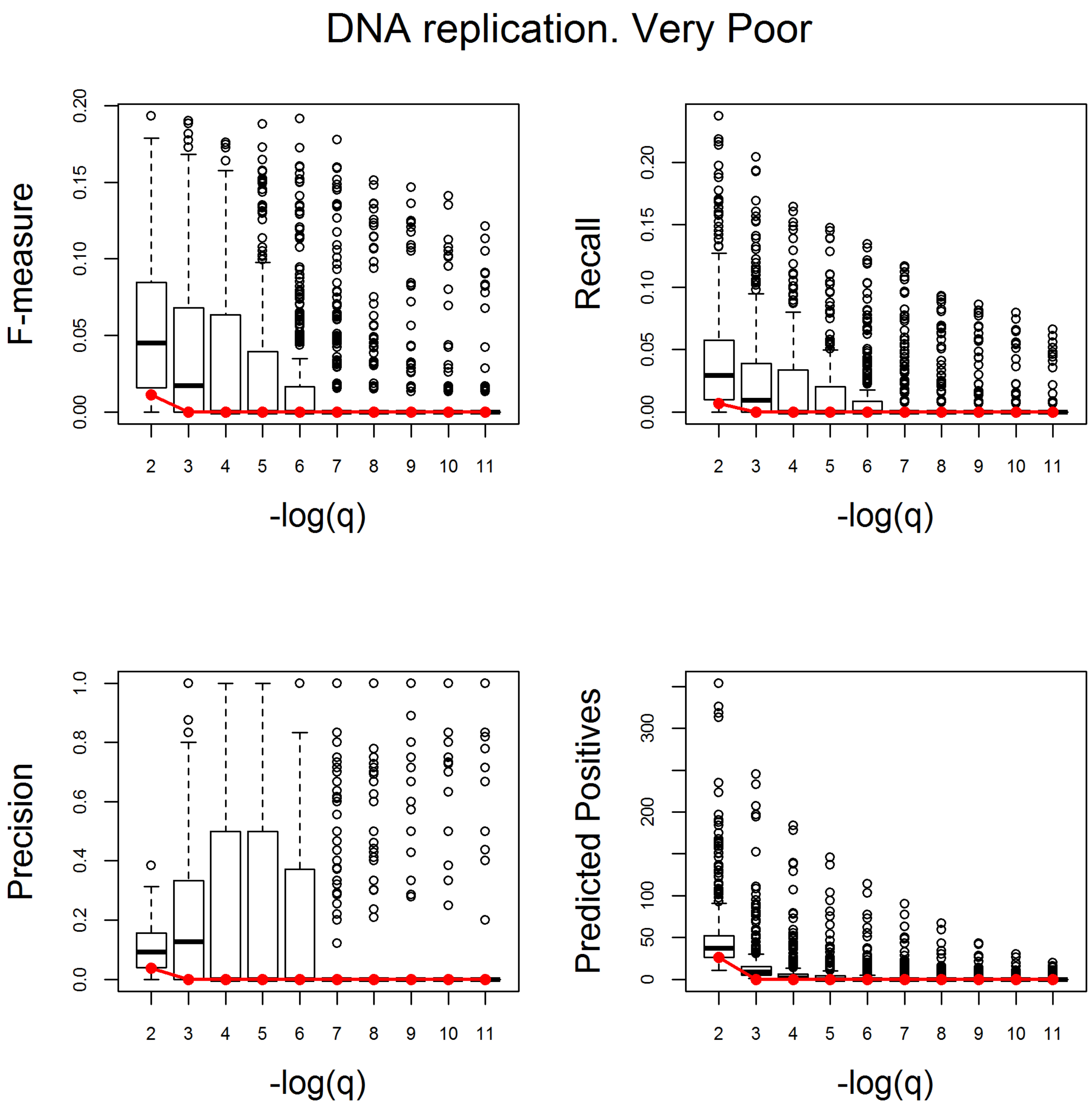

Appendix Figure S14. (continued)

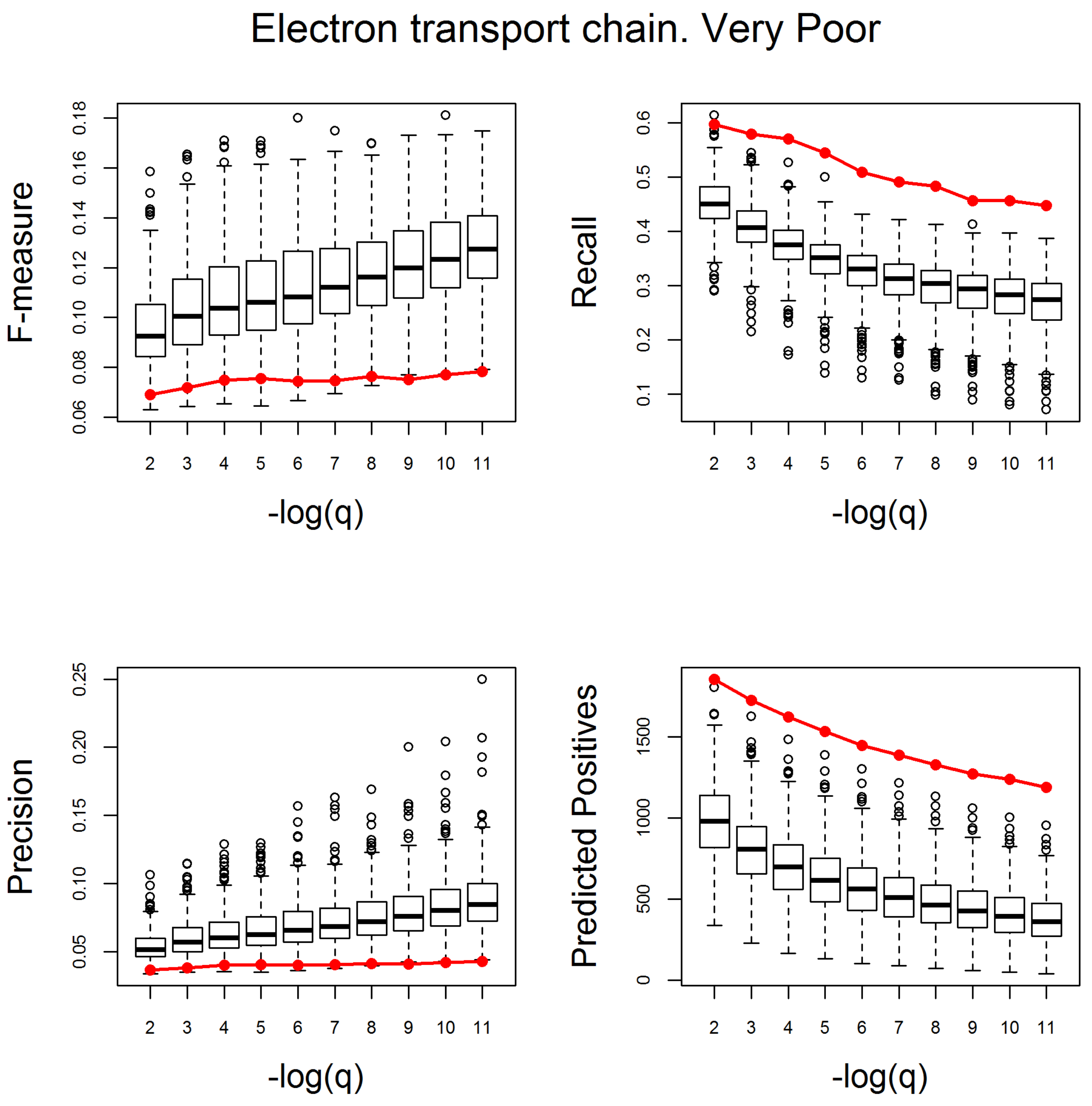

Appendix Figure S14. (continued)

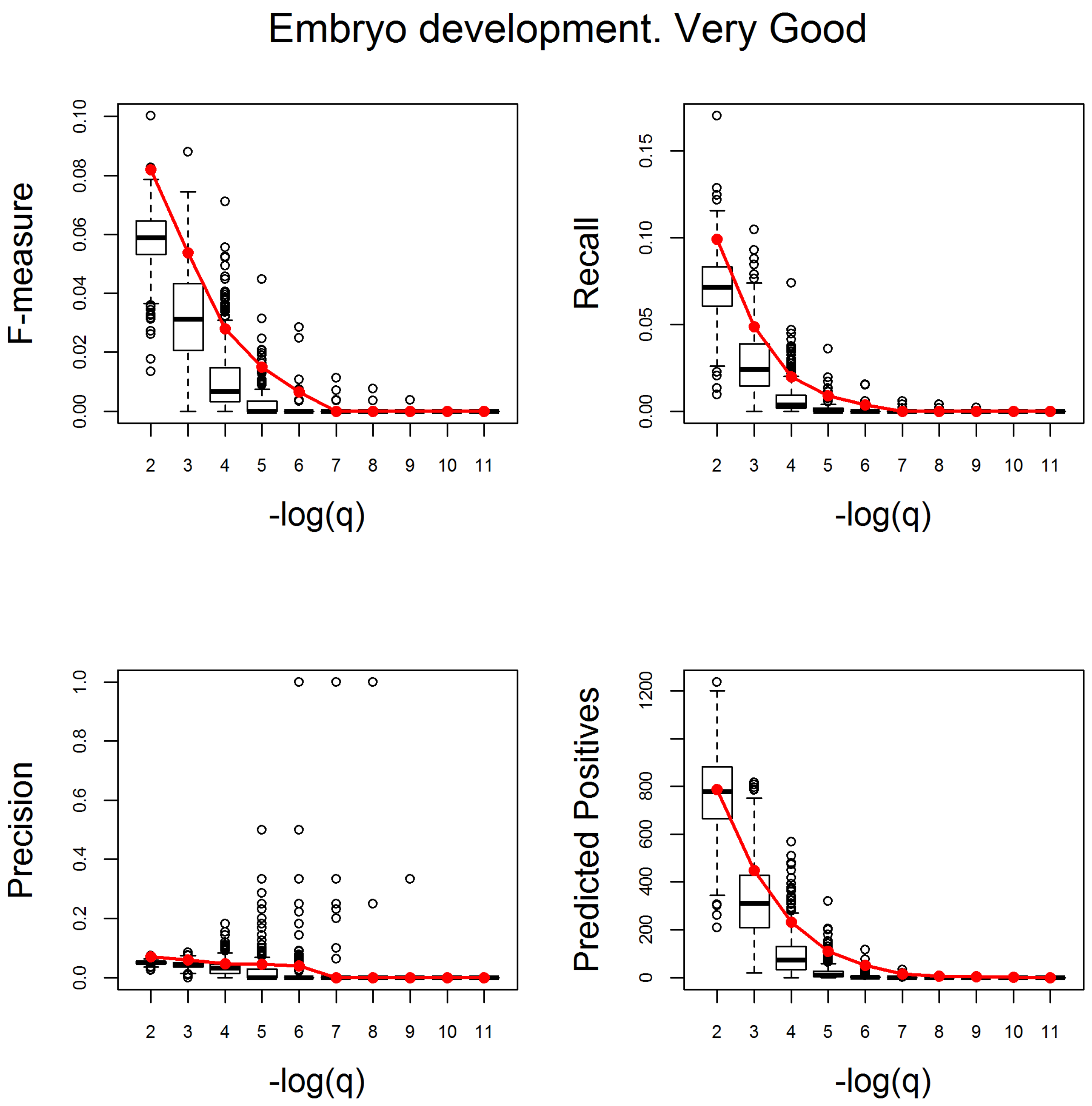

Appendix Figure S14. (continued)

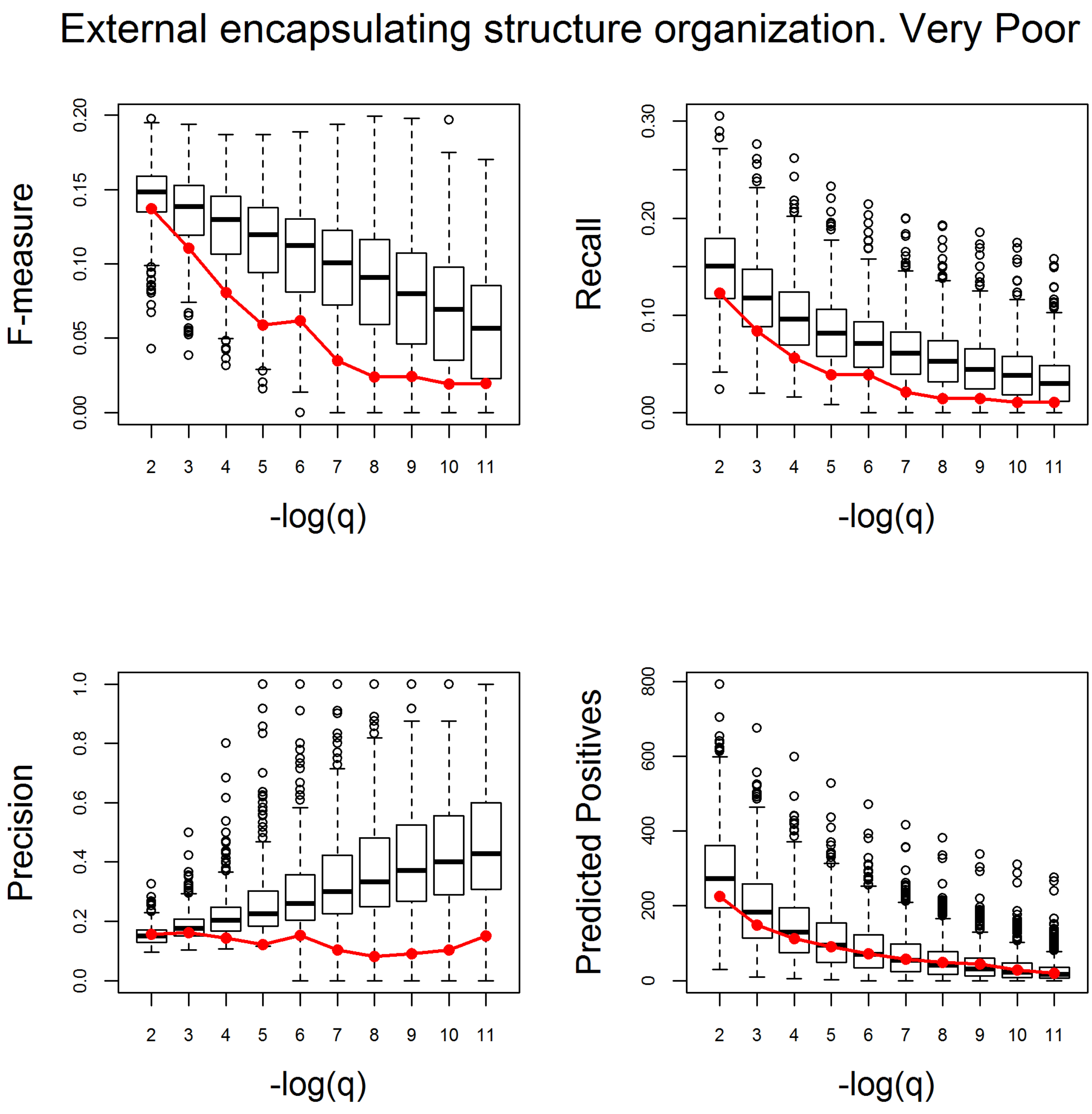

Appendix Figure S14. (continued)

Flower development. Very Poor

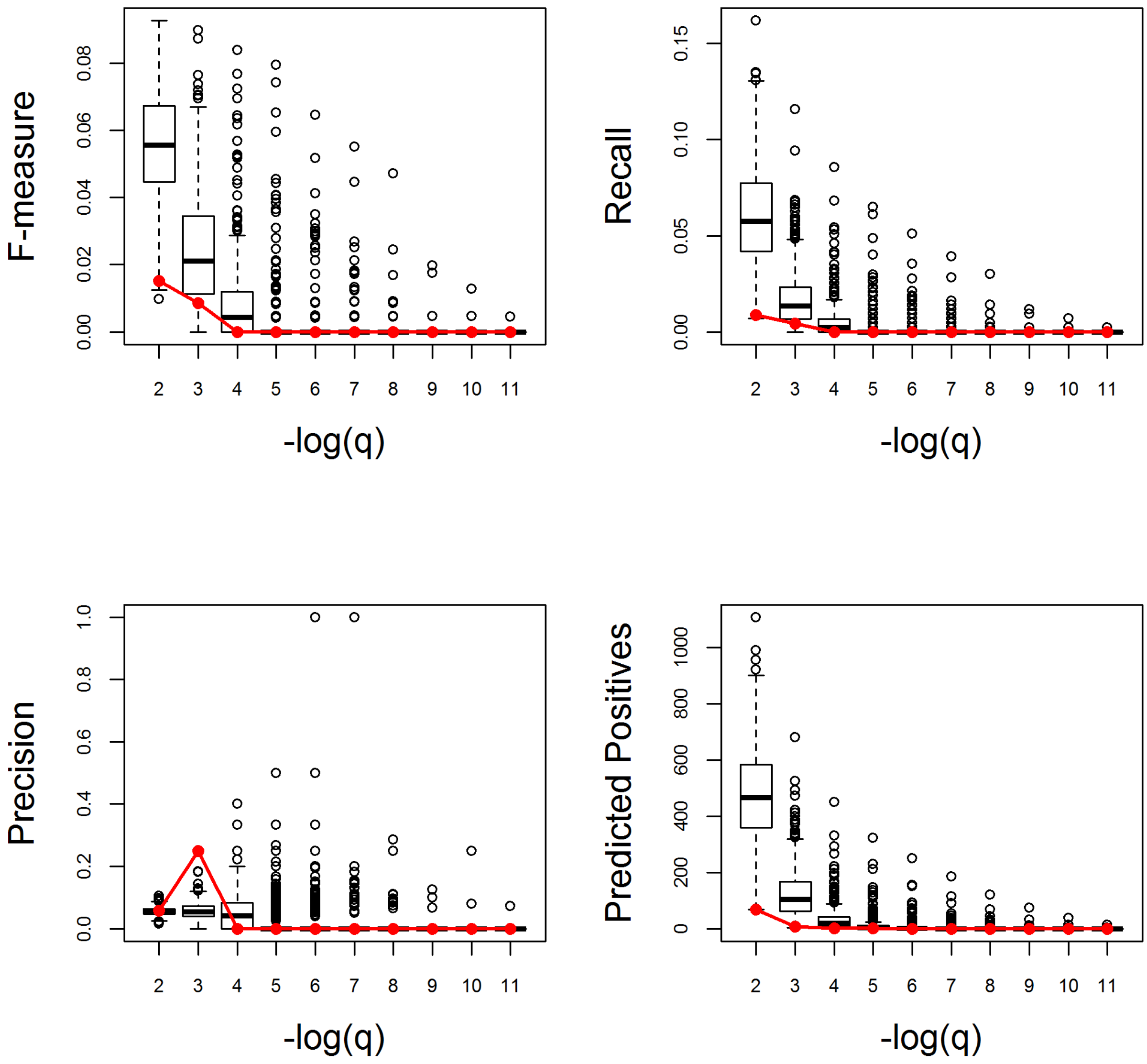

Appendix Figure S14. (continued)

Fruit development. Very Poor

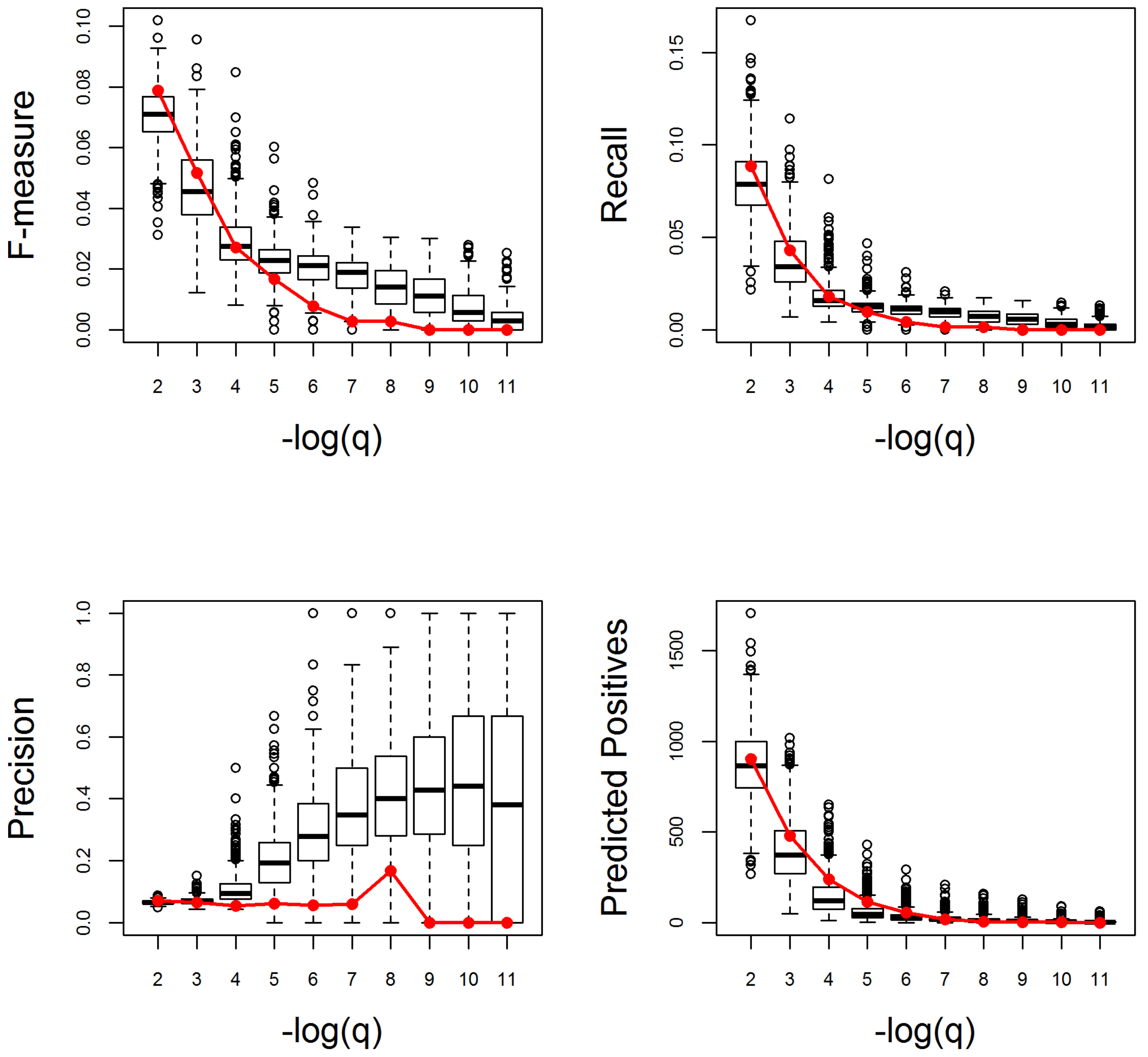

Appendix Figure S14. (continued)

Generation of precursor metabolites and energy. Very Poor

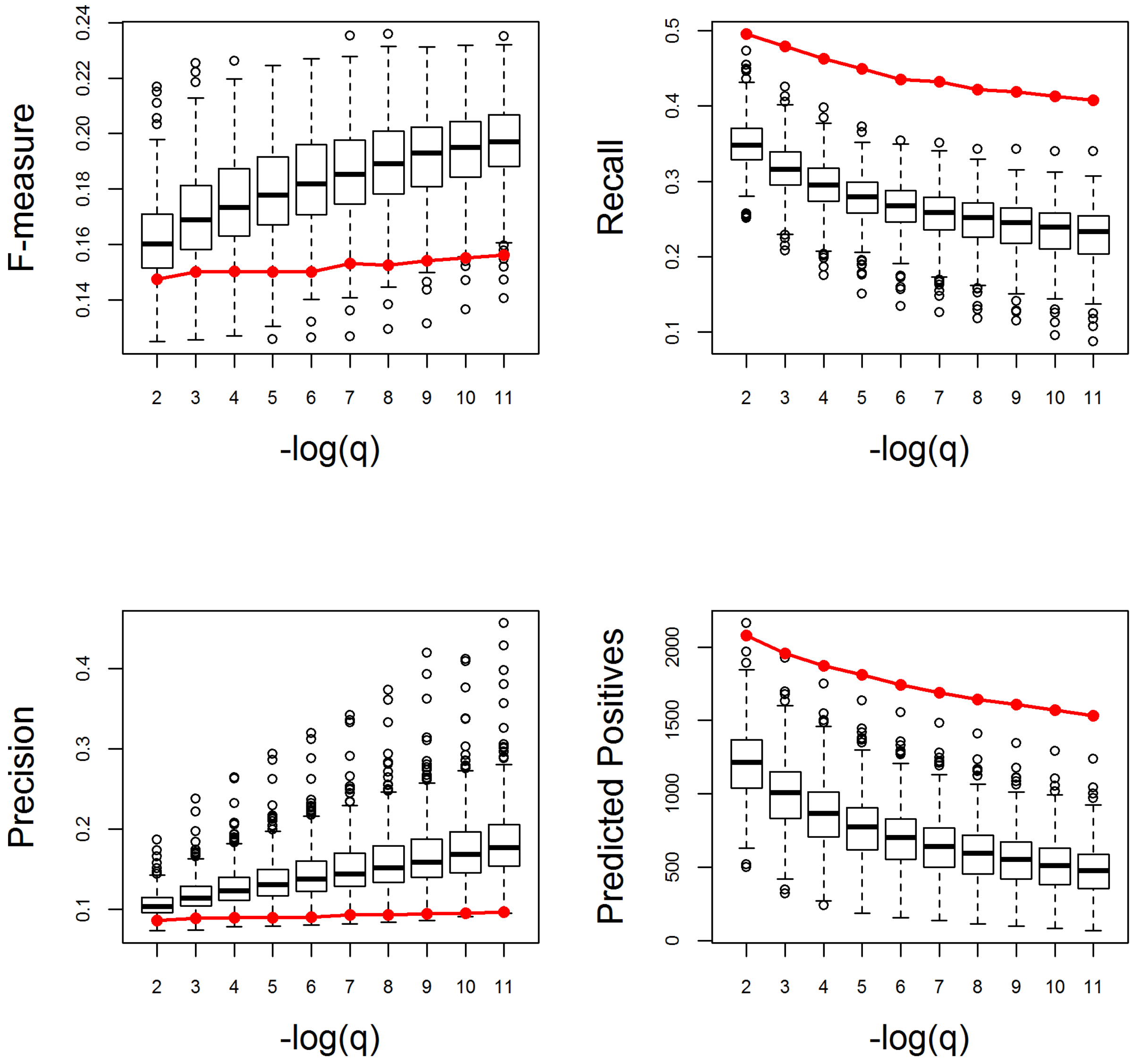

Appendix Figure S14. (continued)

Glucan metabolic process. Very Poor

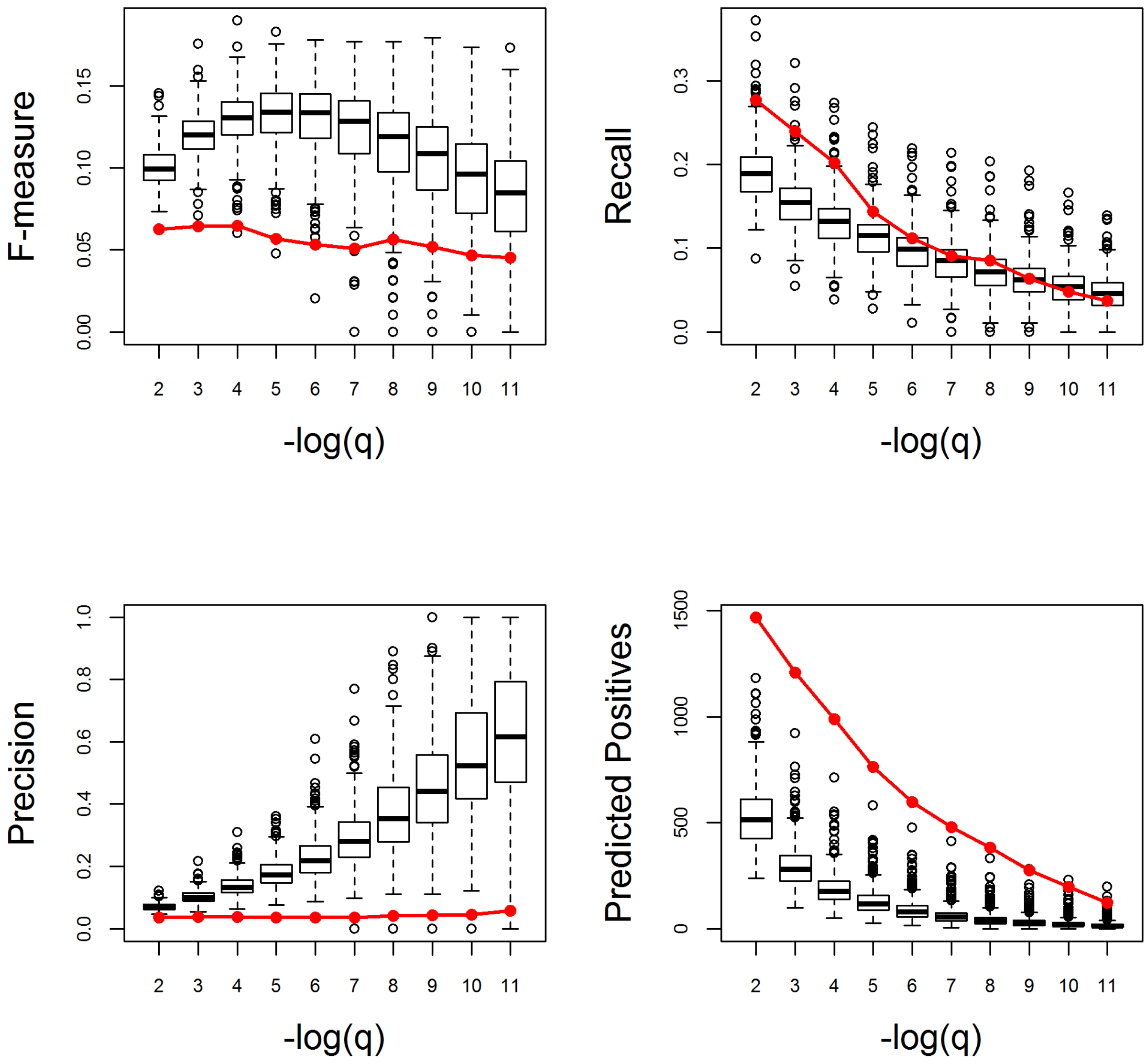

Appendix Figure S14. (continued)

Growth. Very Poor

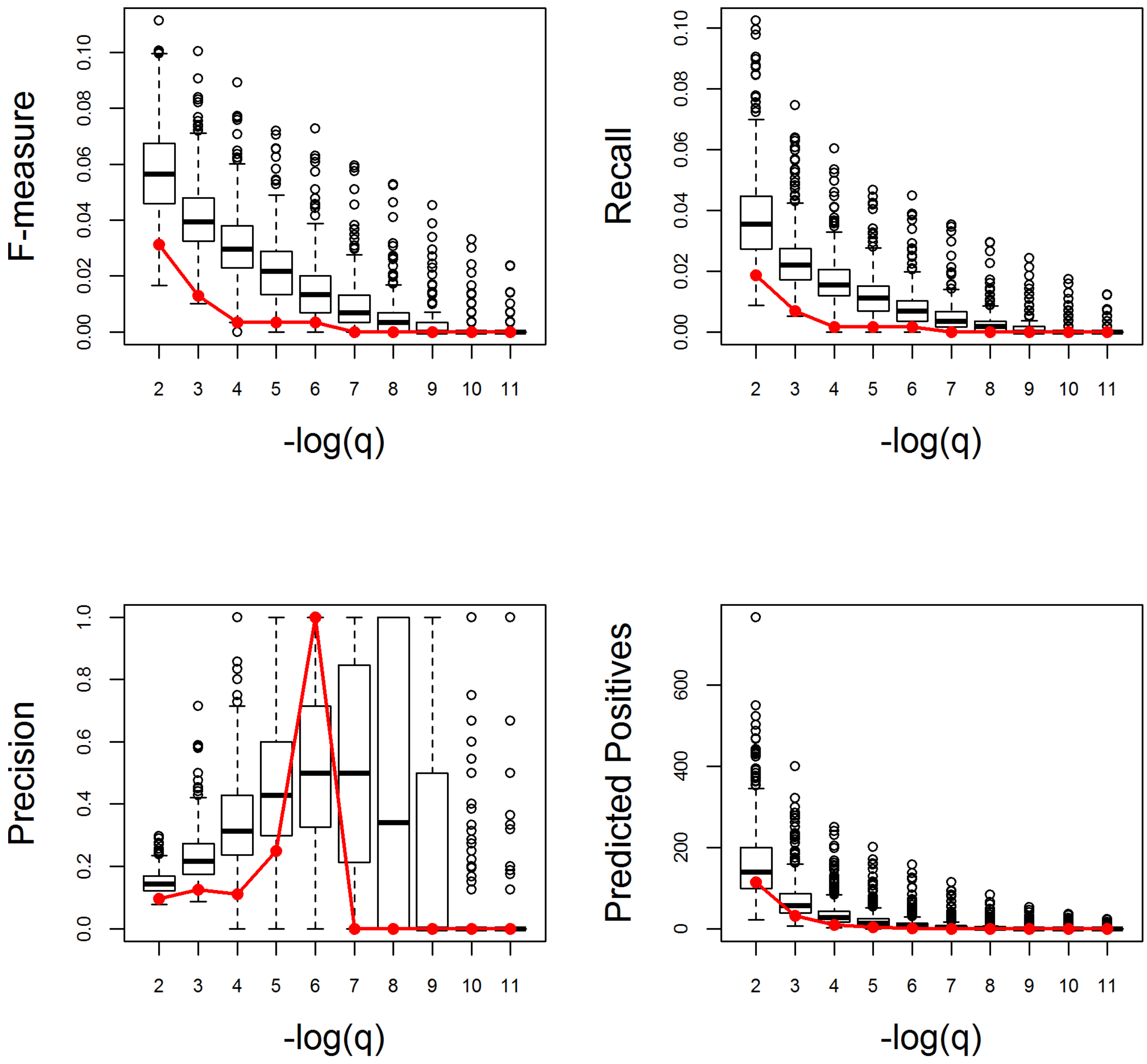

Appendix Figure S14. (continued)

Hemicellulose metabolic process. Very Good

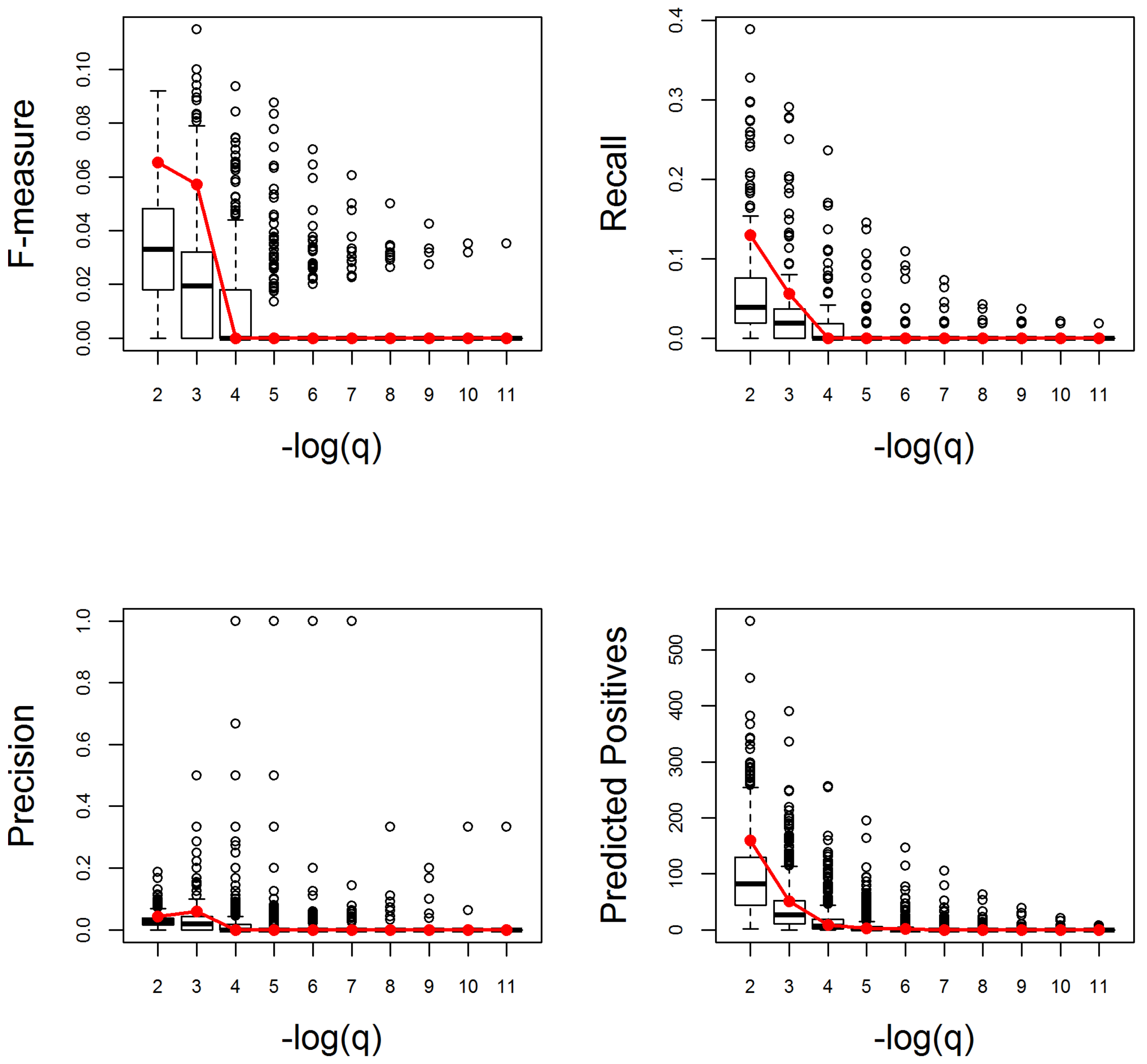

Appendix Figure S14. (continued)

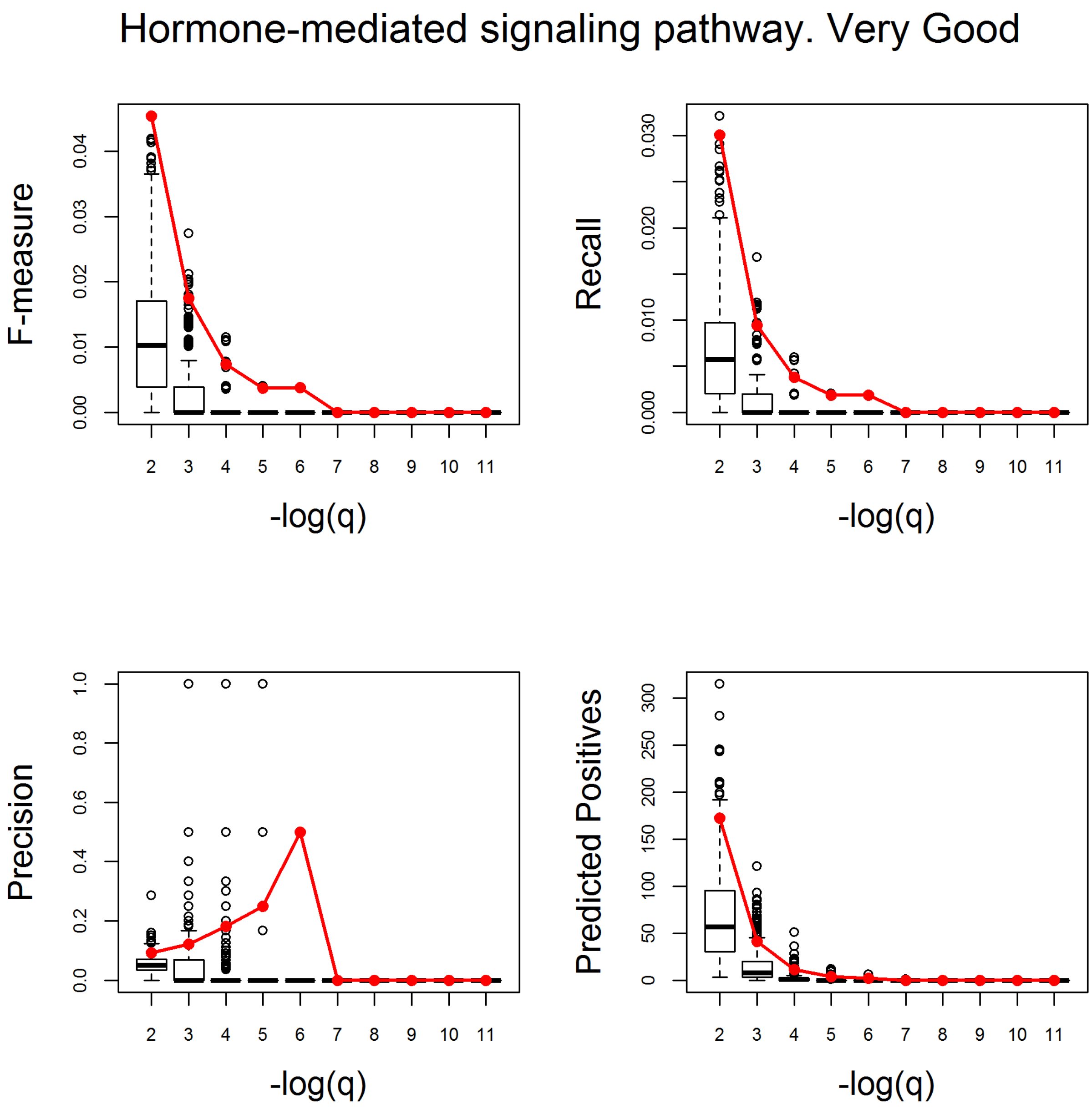

Appendix Figure S14. (continued)

Hyperosmotic response. Very Poor

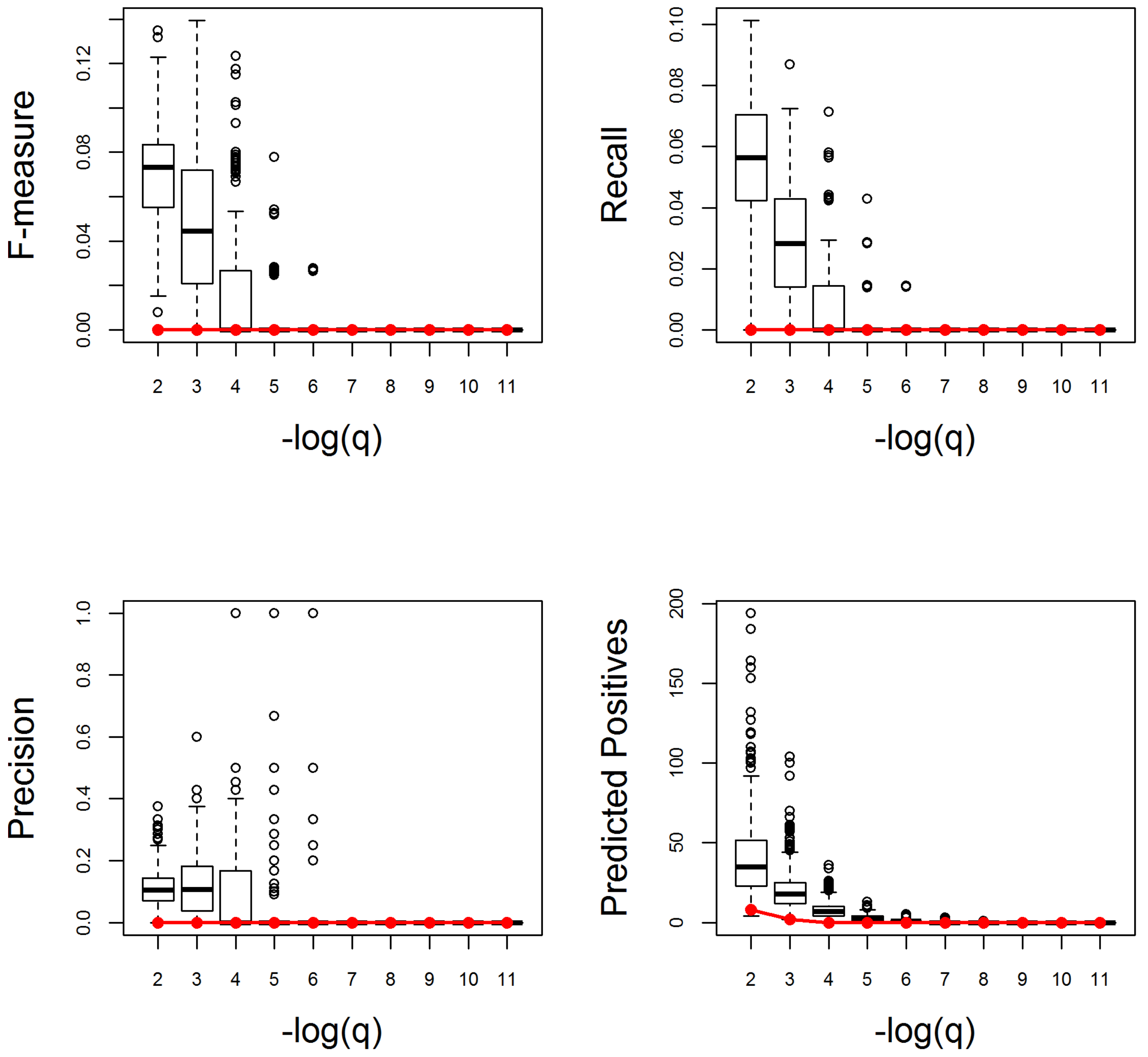

Appendix Figure S14. (continued)

Intracellular receptor signaling pathway. Very Good

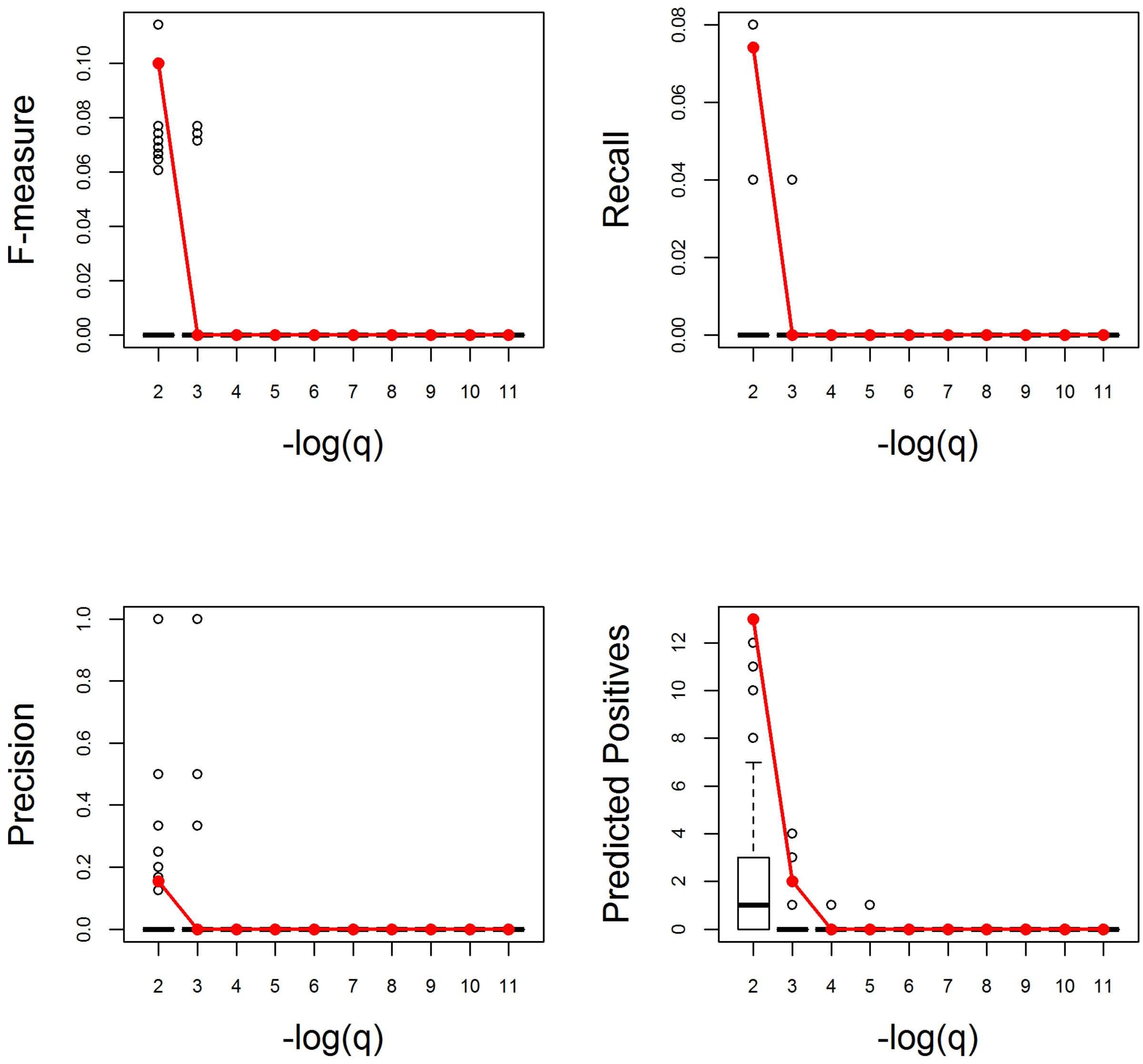

Appendix Figure S14. (continued)

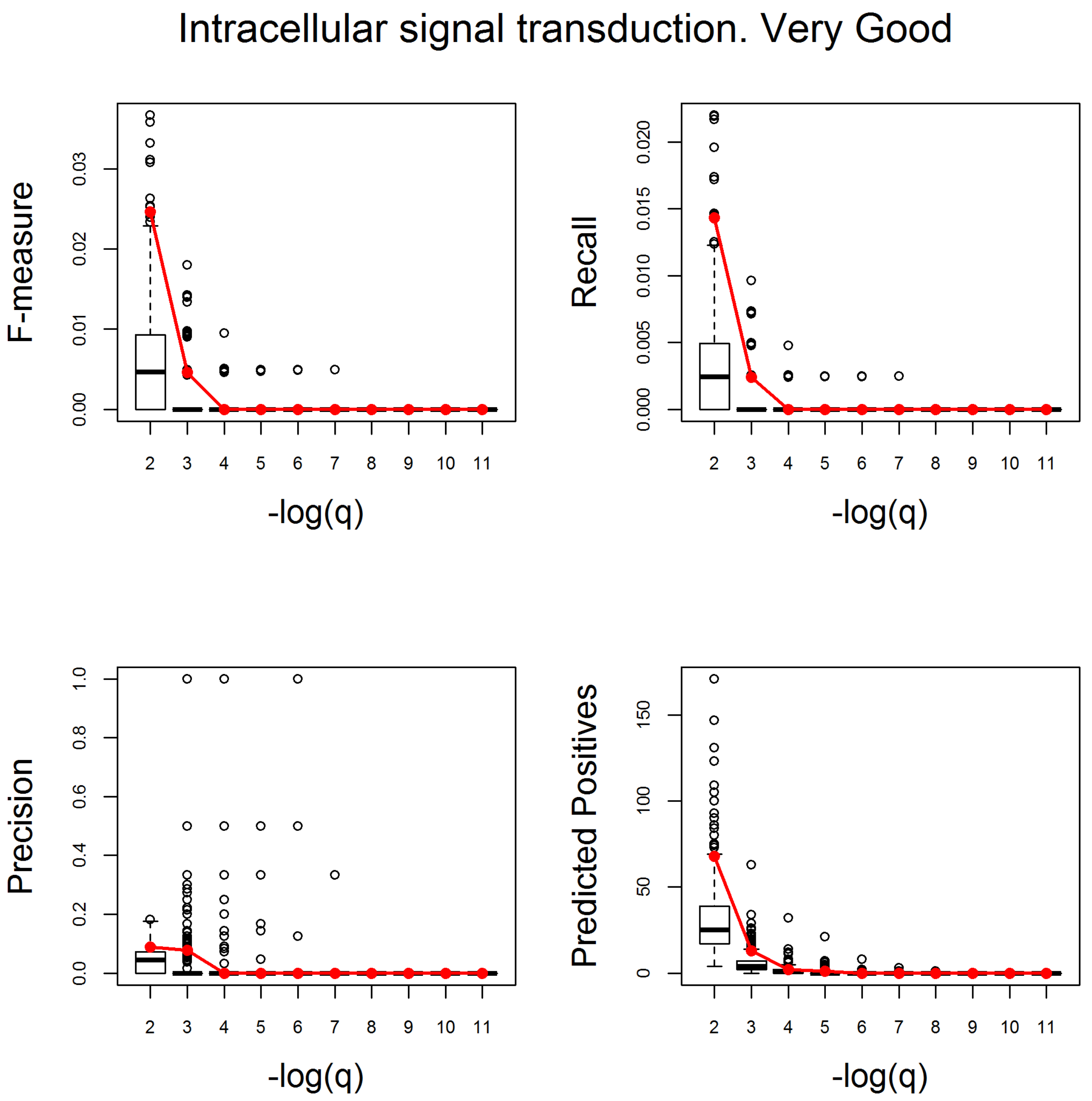

Appendix Figure S14. (continued)

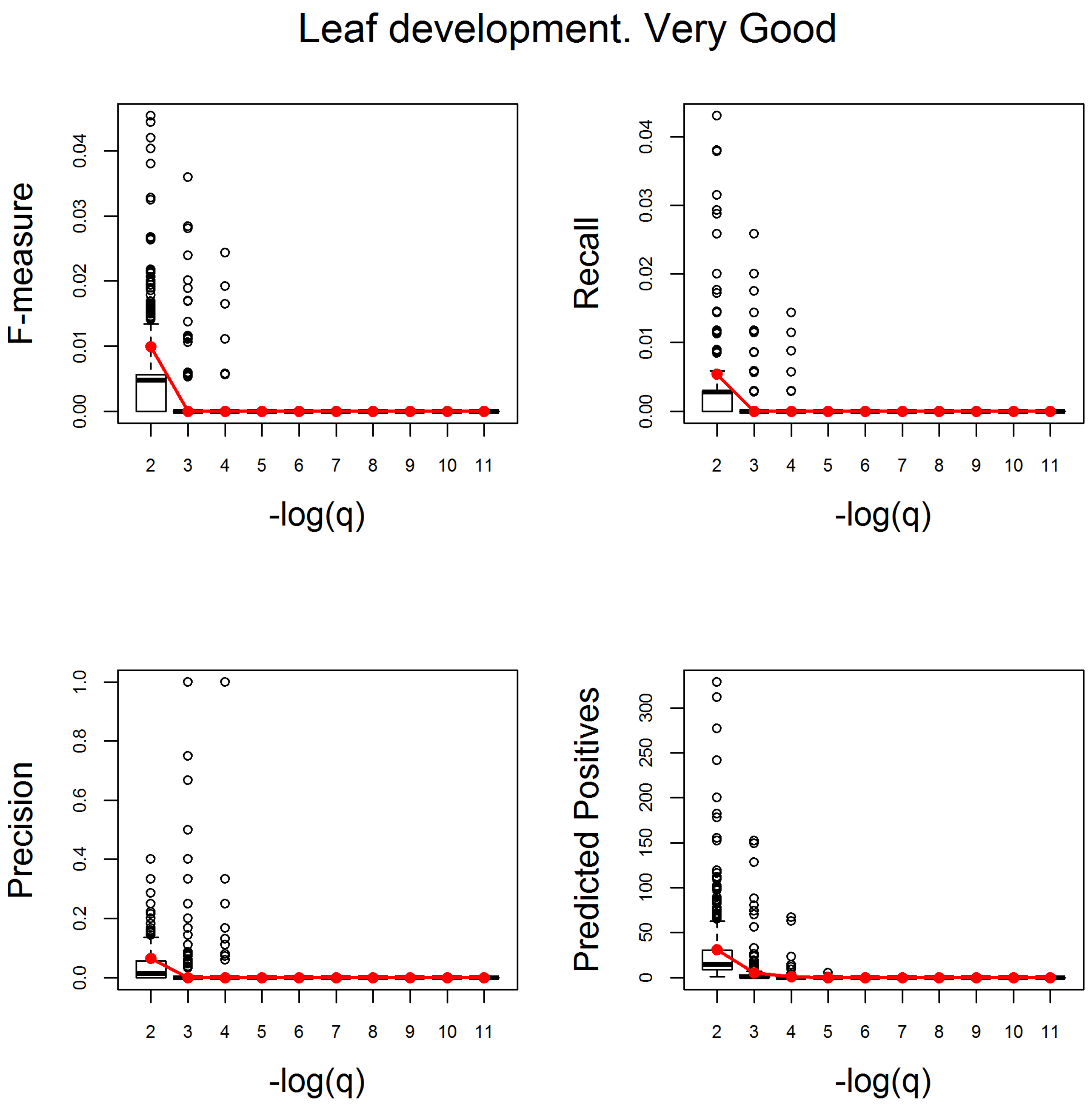

Appendix Figure S14. (continued)

Lipid metabolic process. Very Good

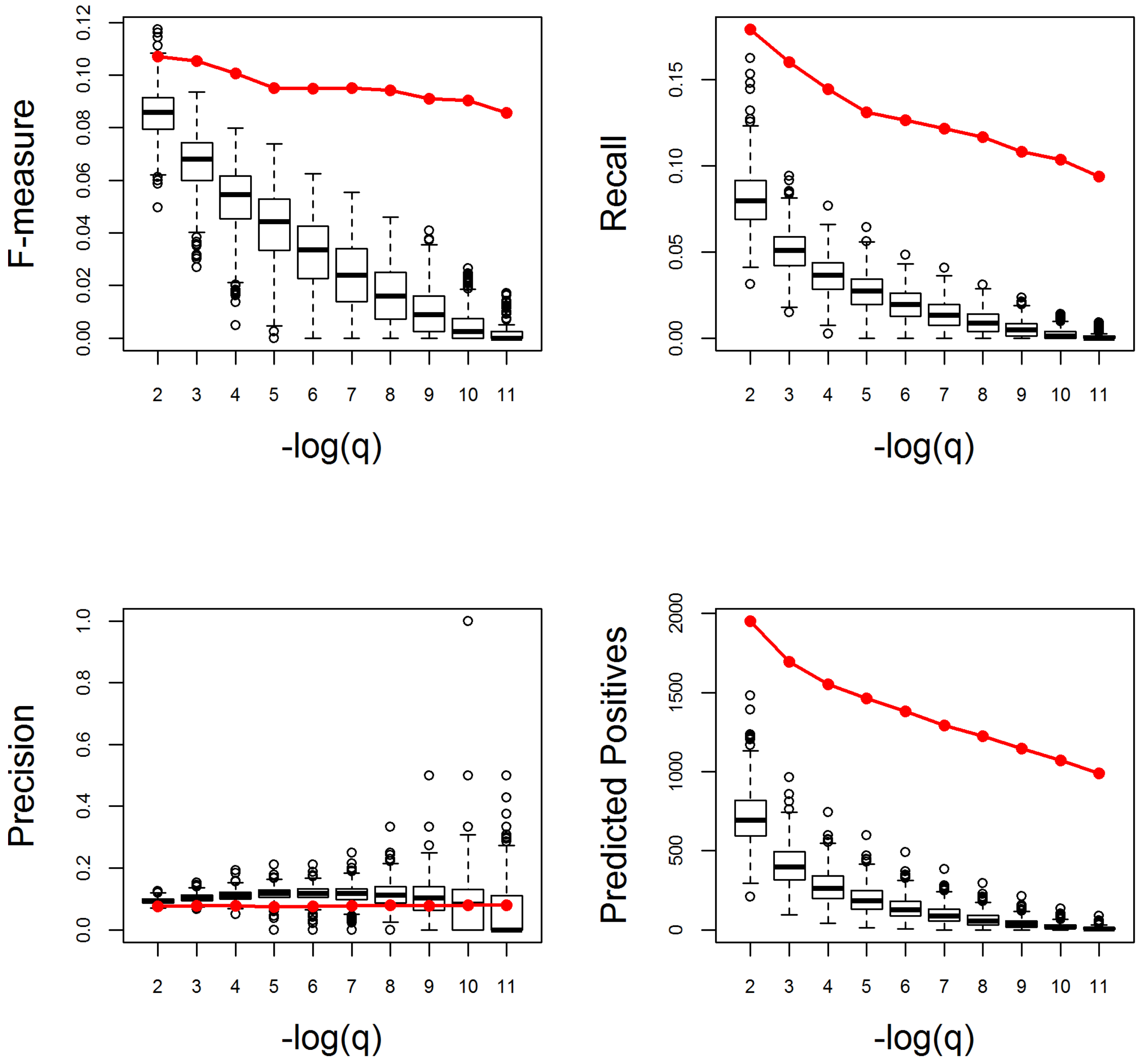

Appendix Figure S14. (continued)

Nucleobase-containing compound metabolic process. Very Good

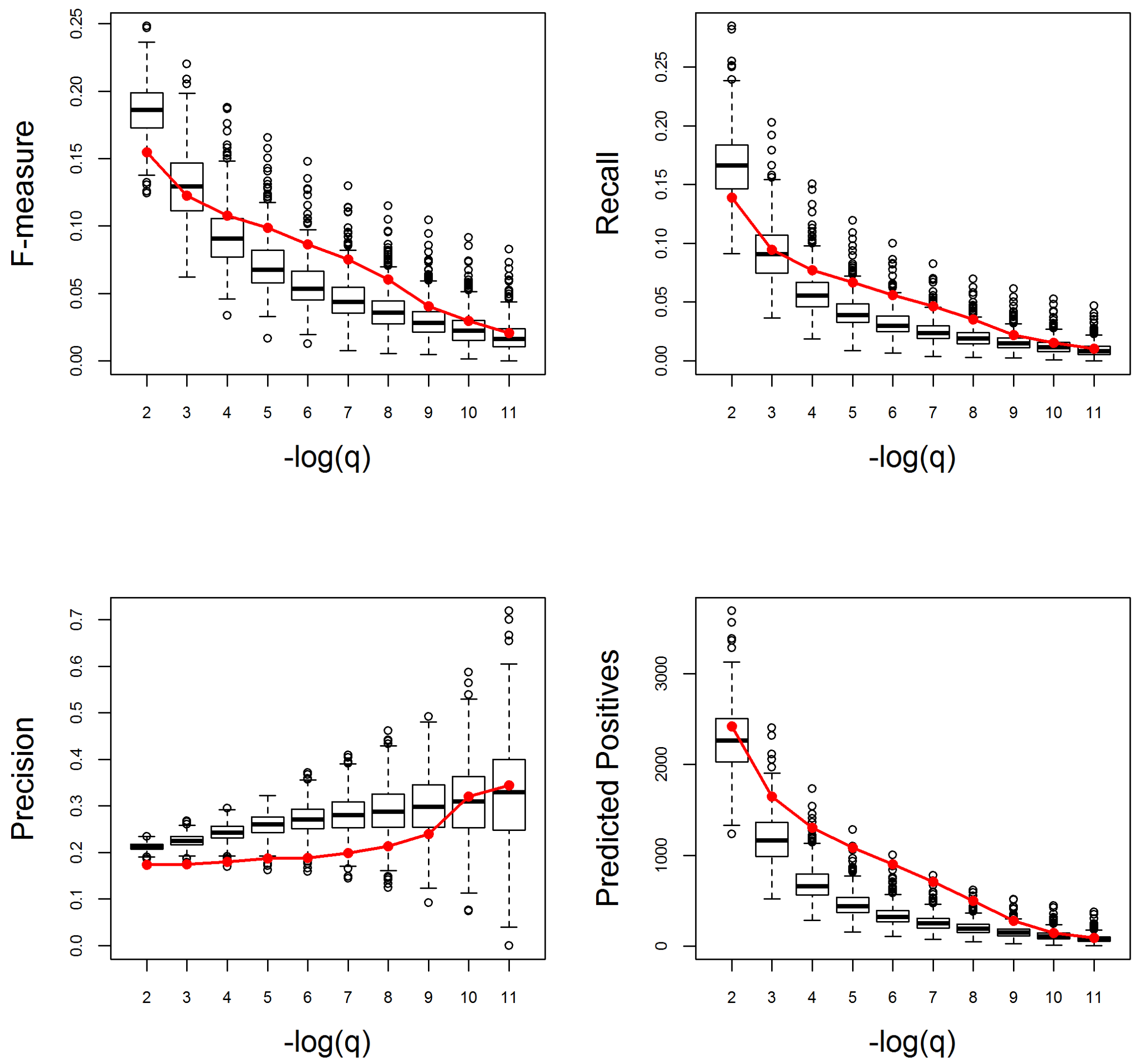

Appendix Figure S14. (continued)

Oxidation-reduction process. Very Good

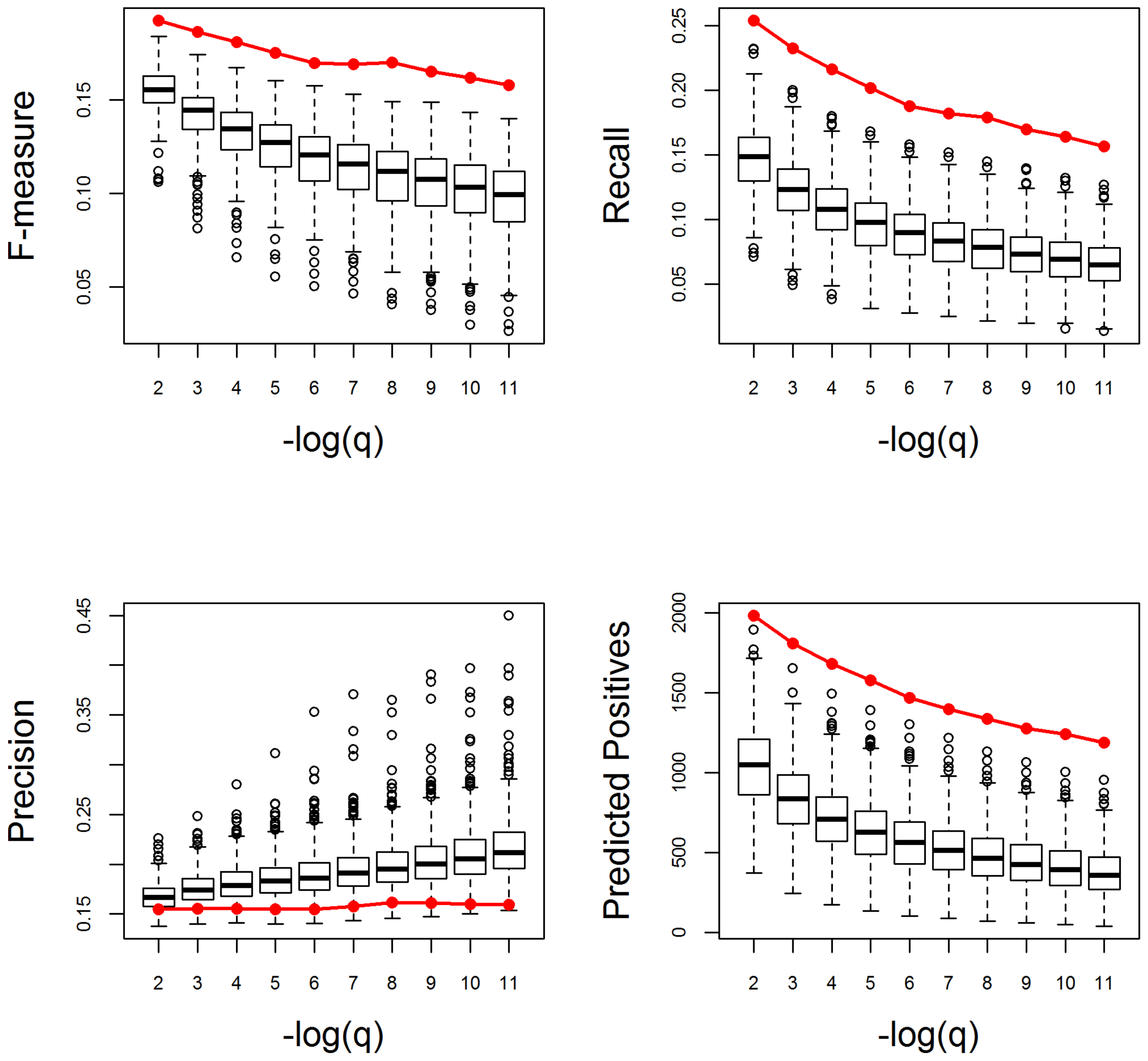

Appendix Figure S14. (continued)

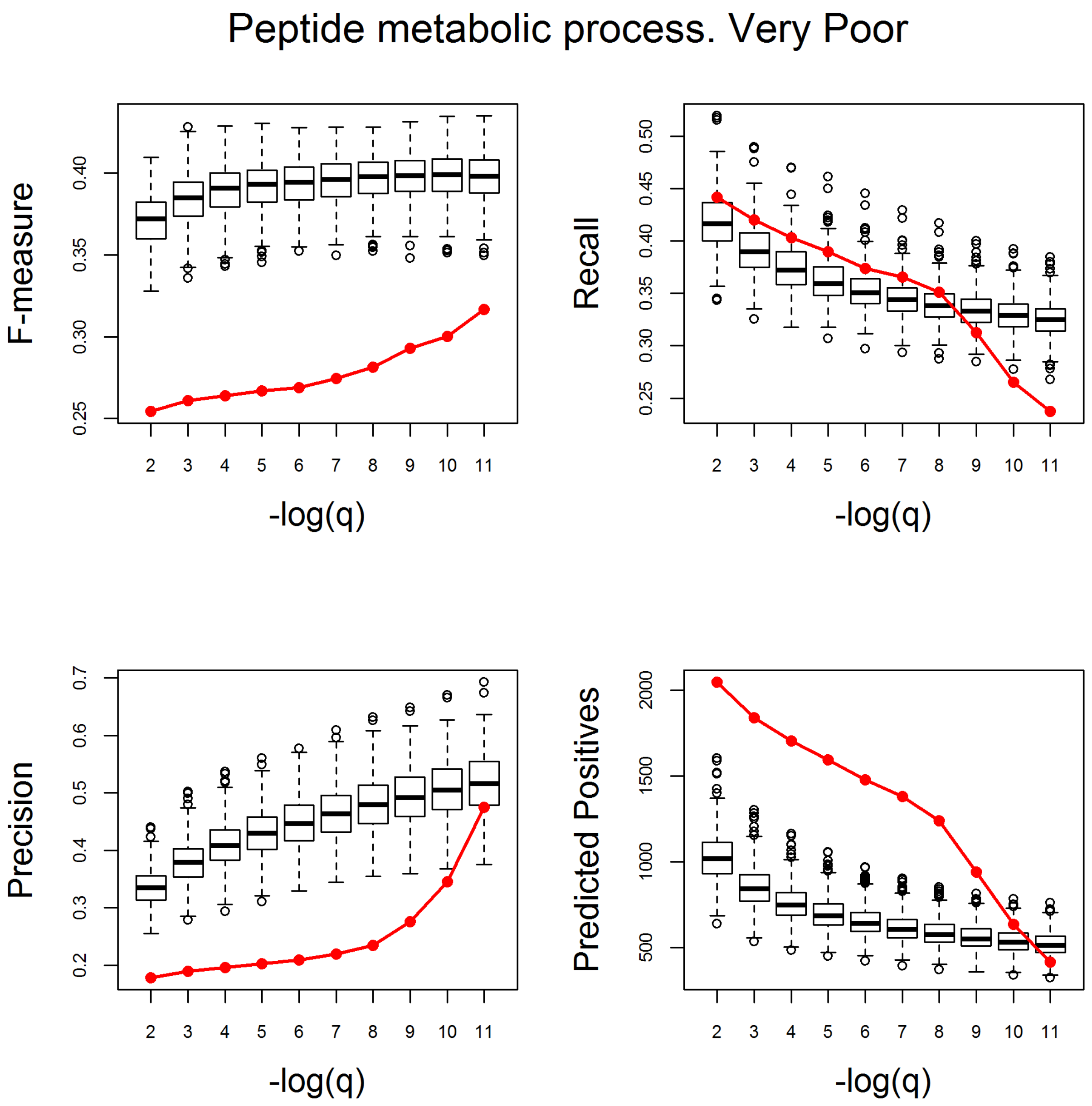

Appendix Figure S14. (continued)

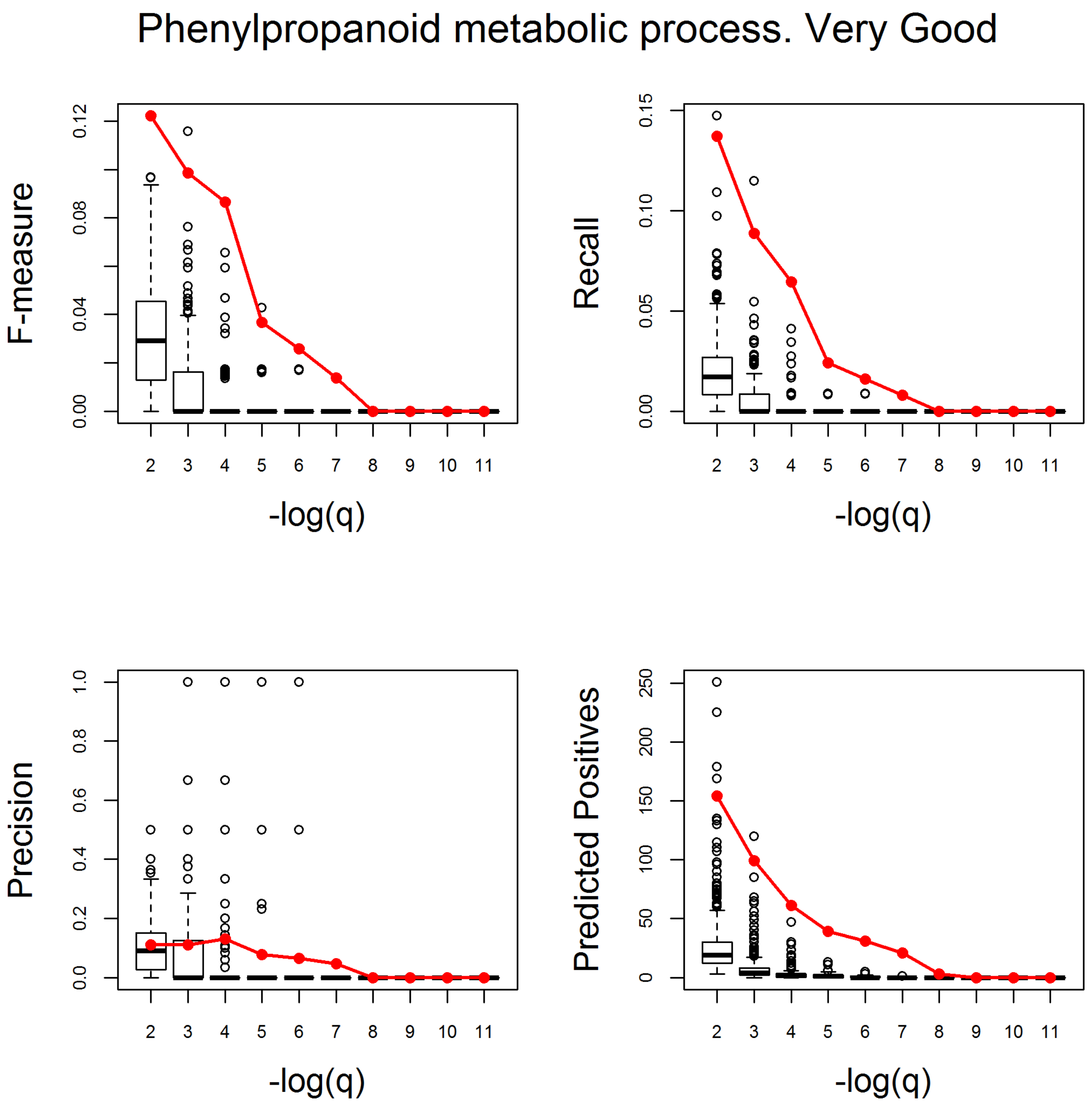

Appendix Figure S14. (continued)

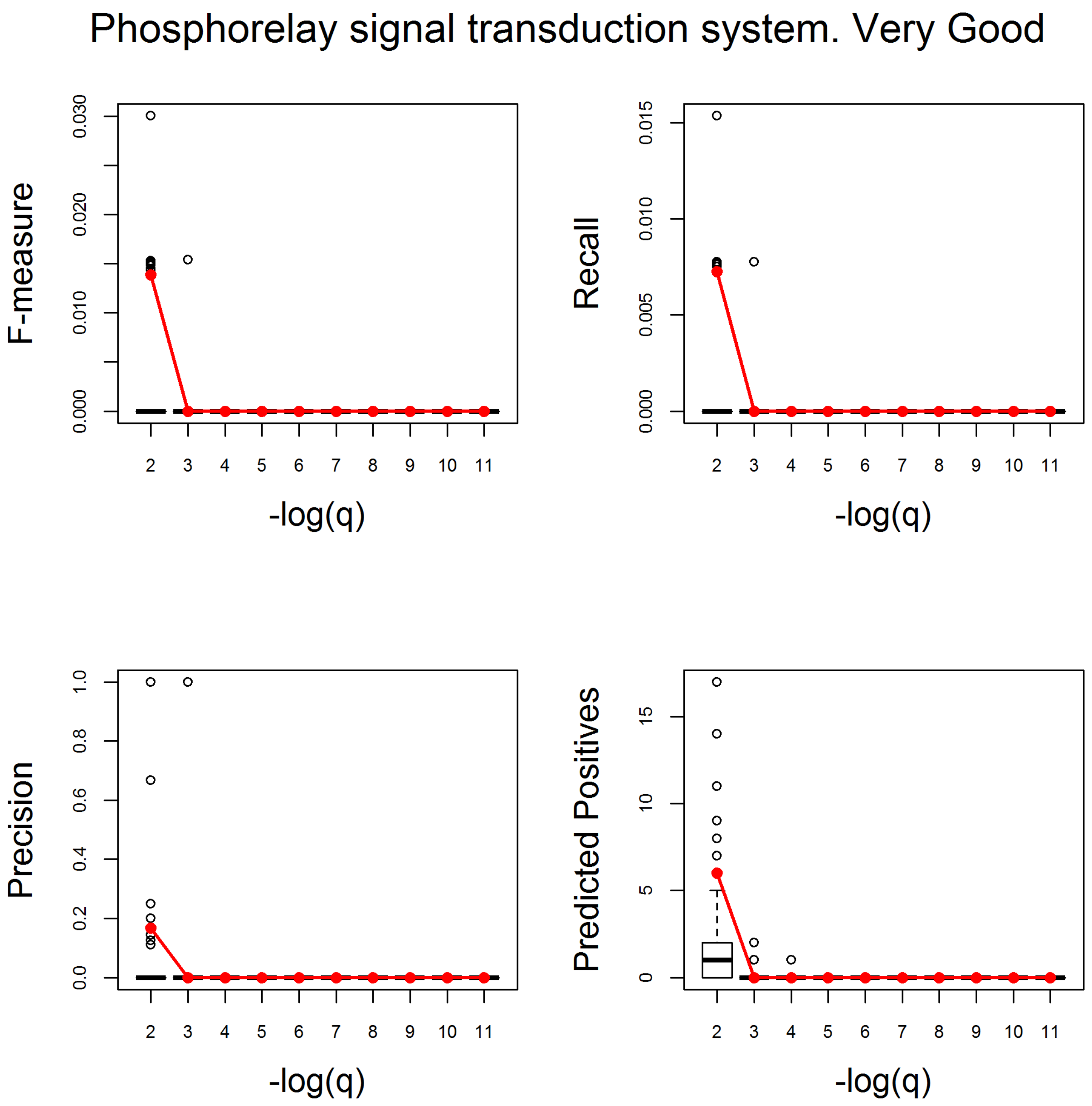

Appendix Figure S14. (continued)

Photoperiodism. Very Poor

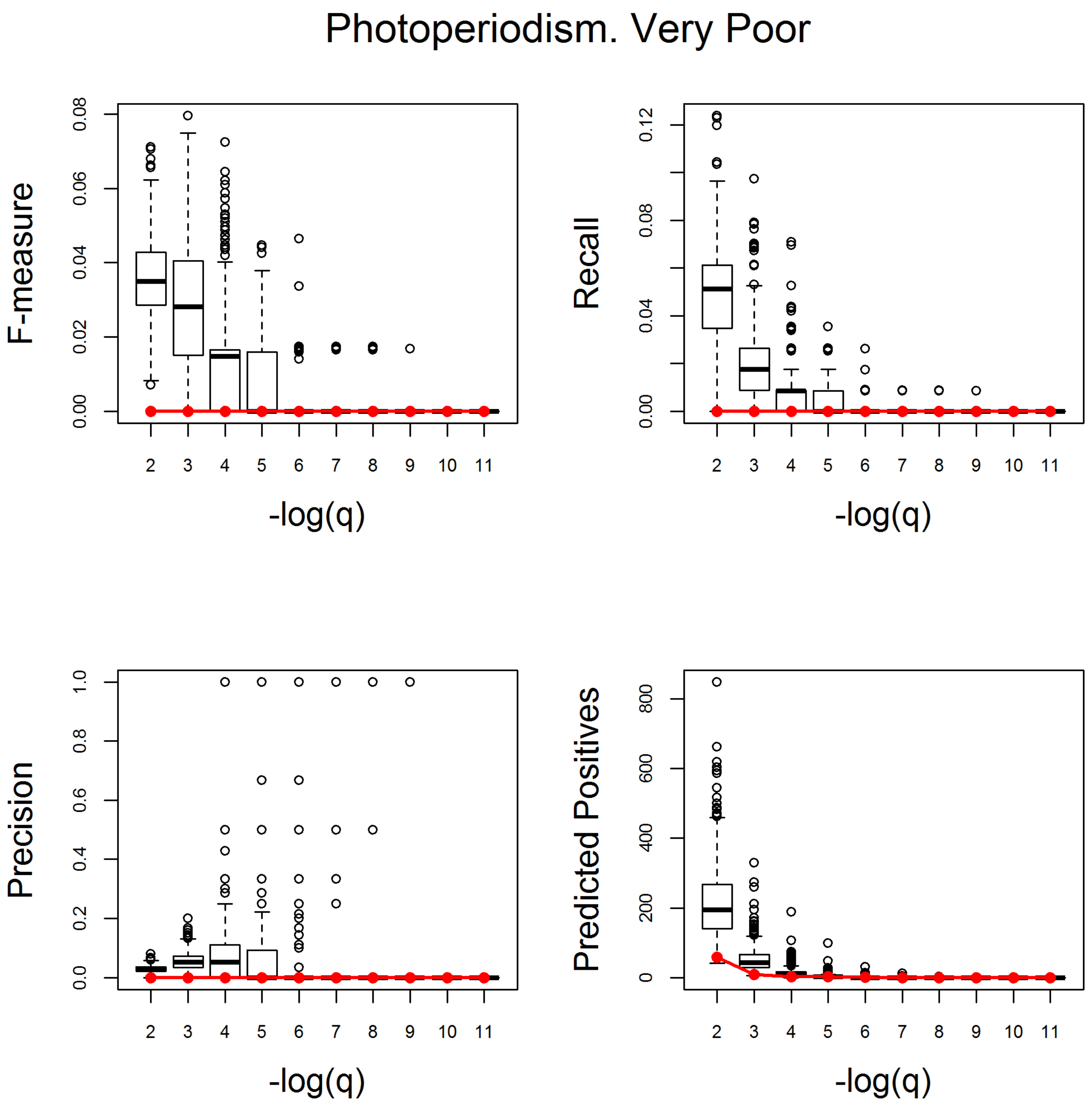

Appendix Figure S14. (continued)

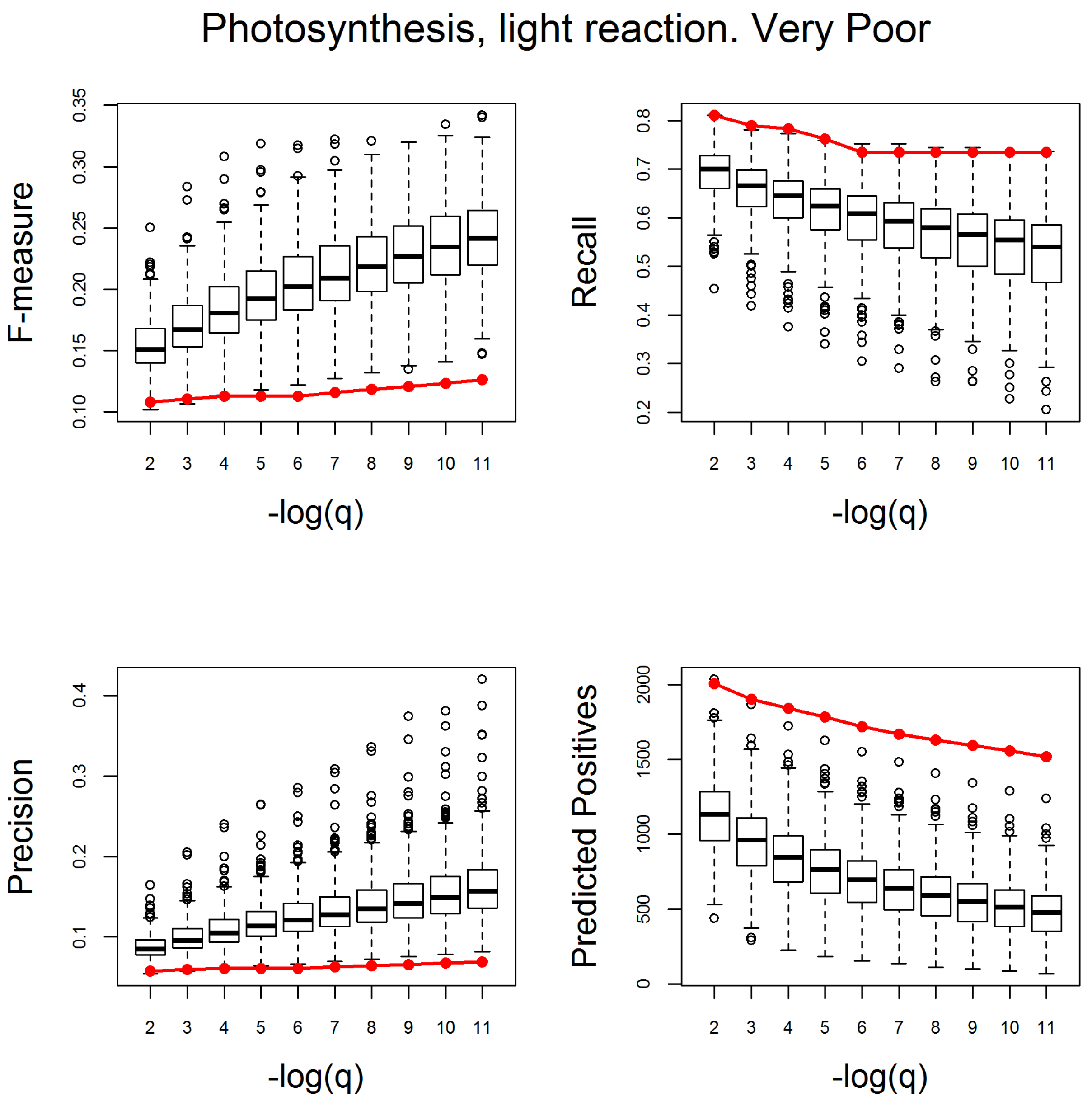

Appendix Figure S14. (continued)

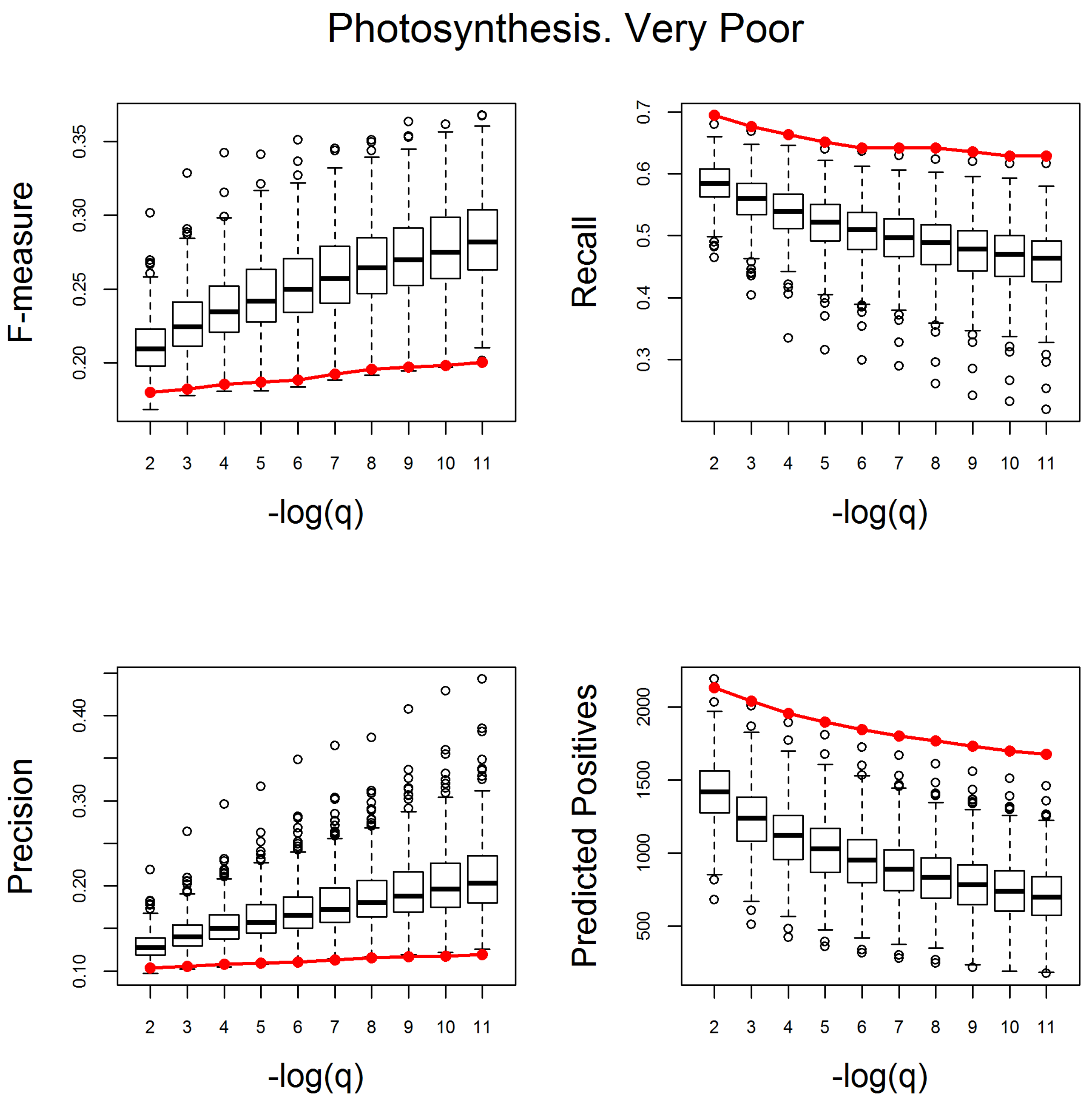

Appendix Figure S14. (continued)

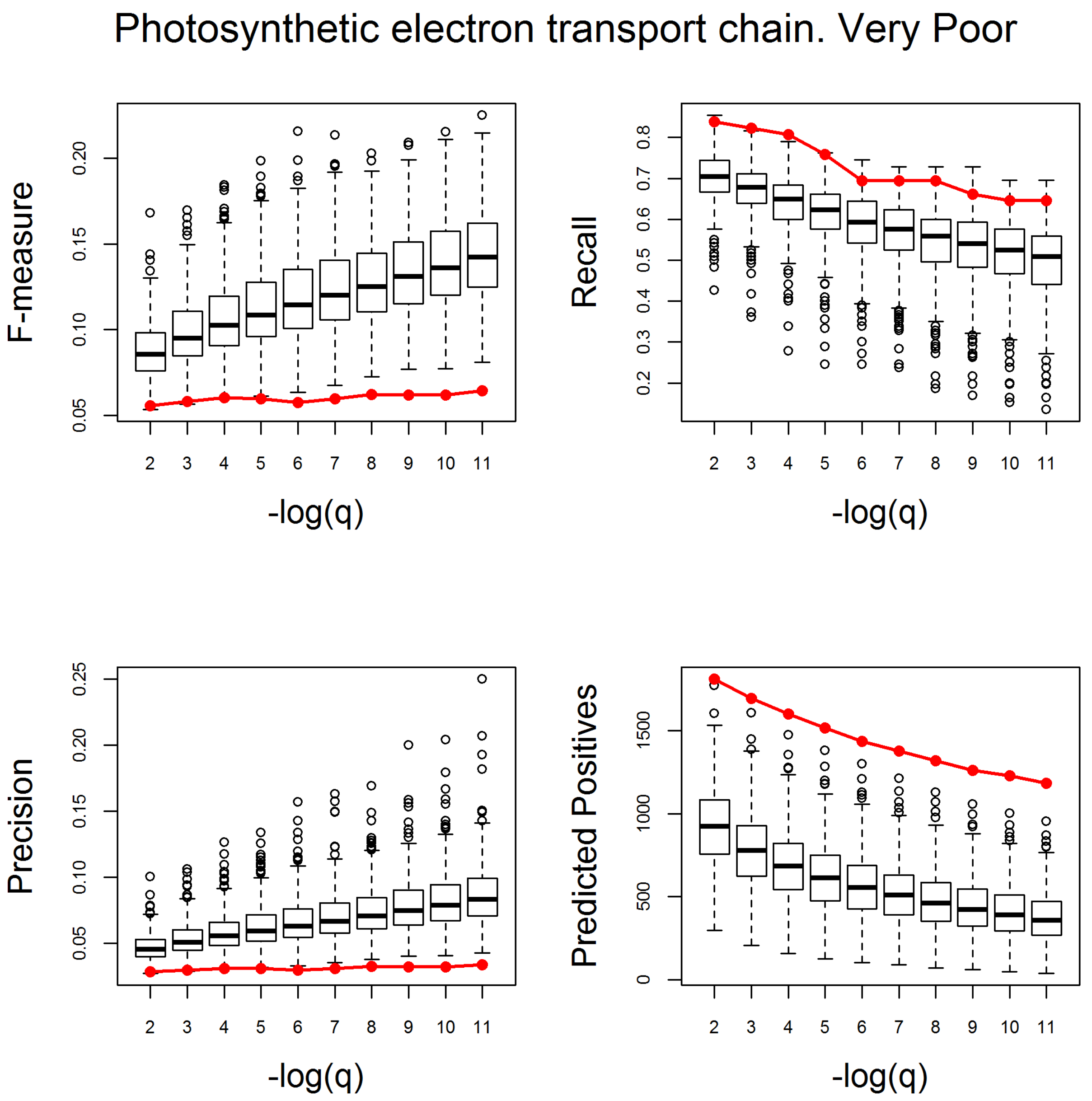

Appendix Figure S14. (continued)

Phototransduction. Poor

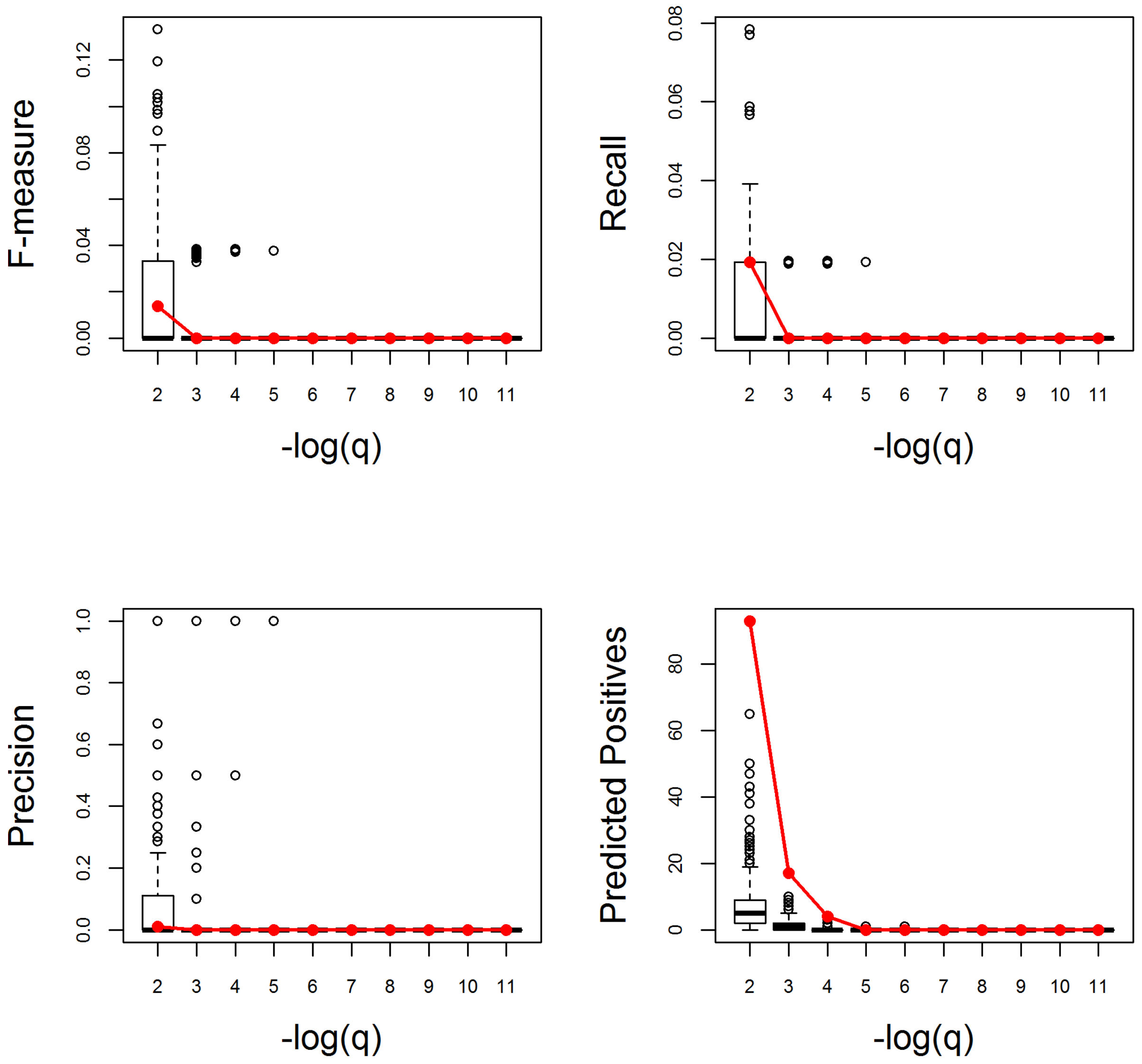

Appendix Figure S14. (continued)

Phyllome development. Poor

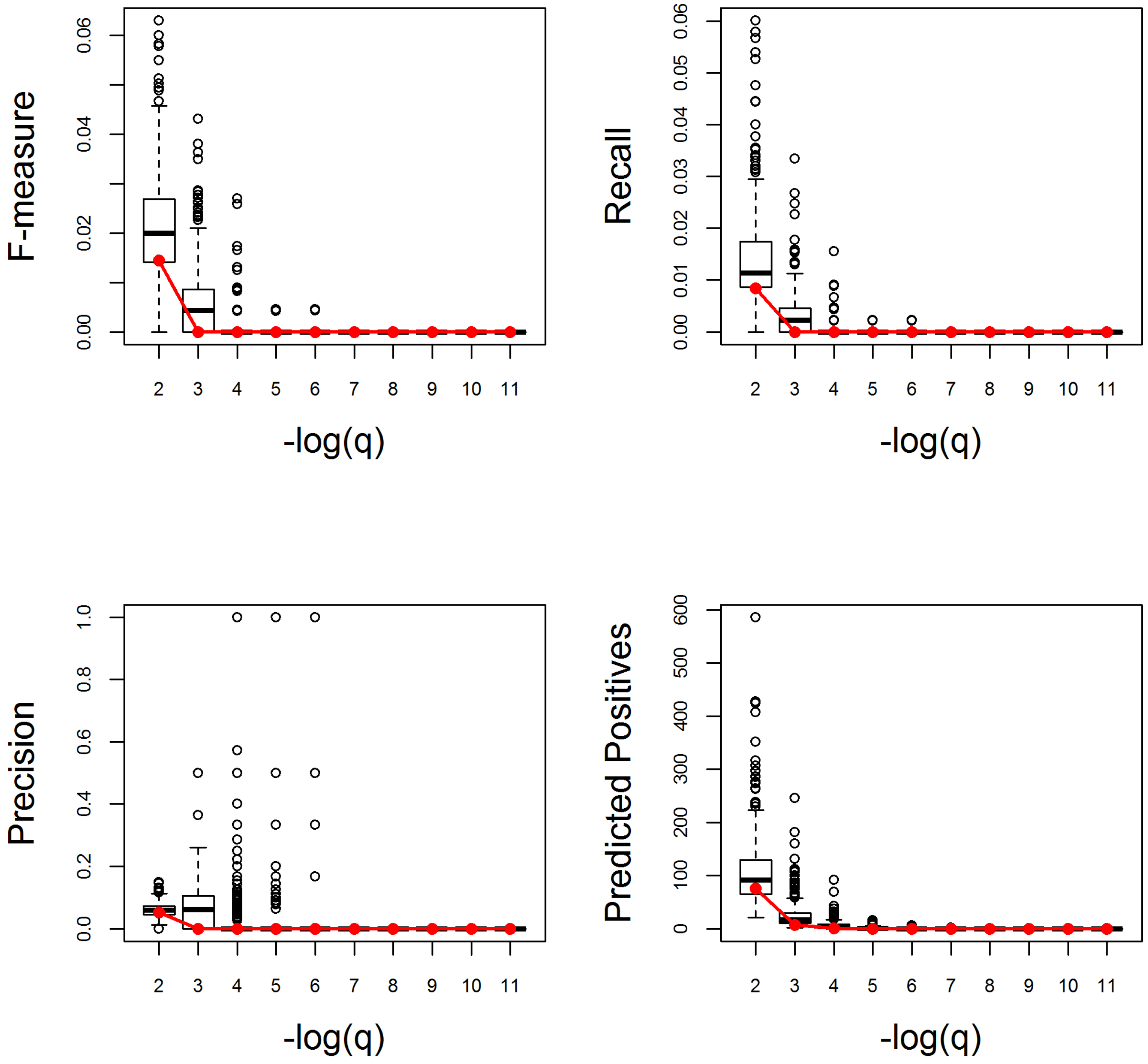

Appendix Figure S14. (continued)

Plant-type cell wall biogenesis. Very Poor

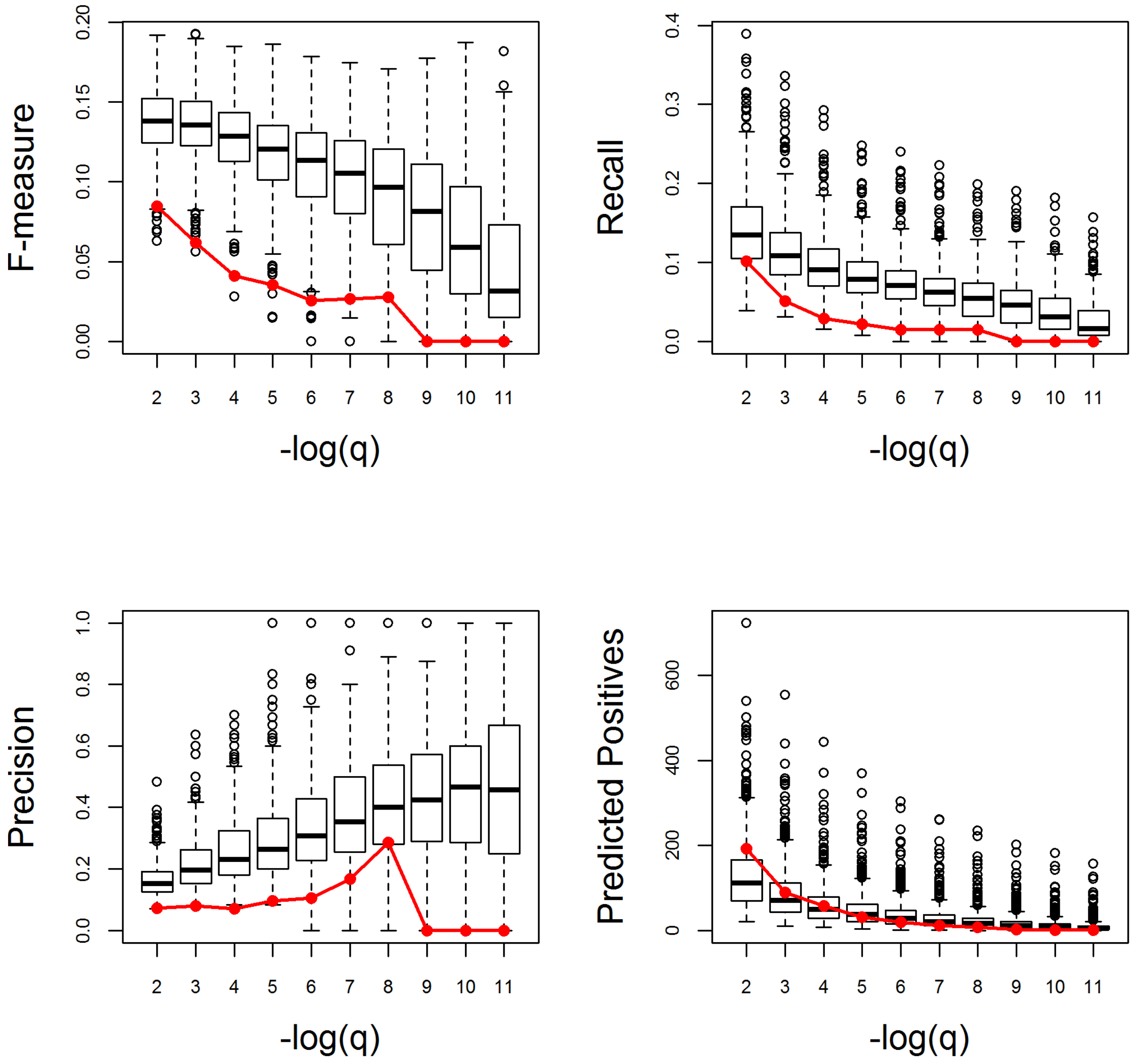

Appendix Figure S14. (continued)

Plant-type secondary cell wall biogenesis. Very Good

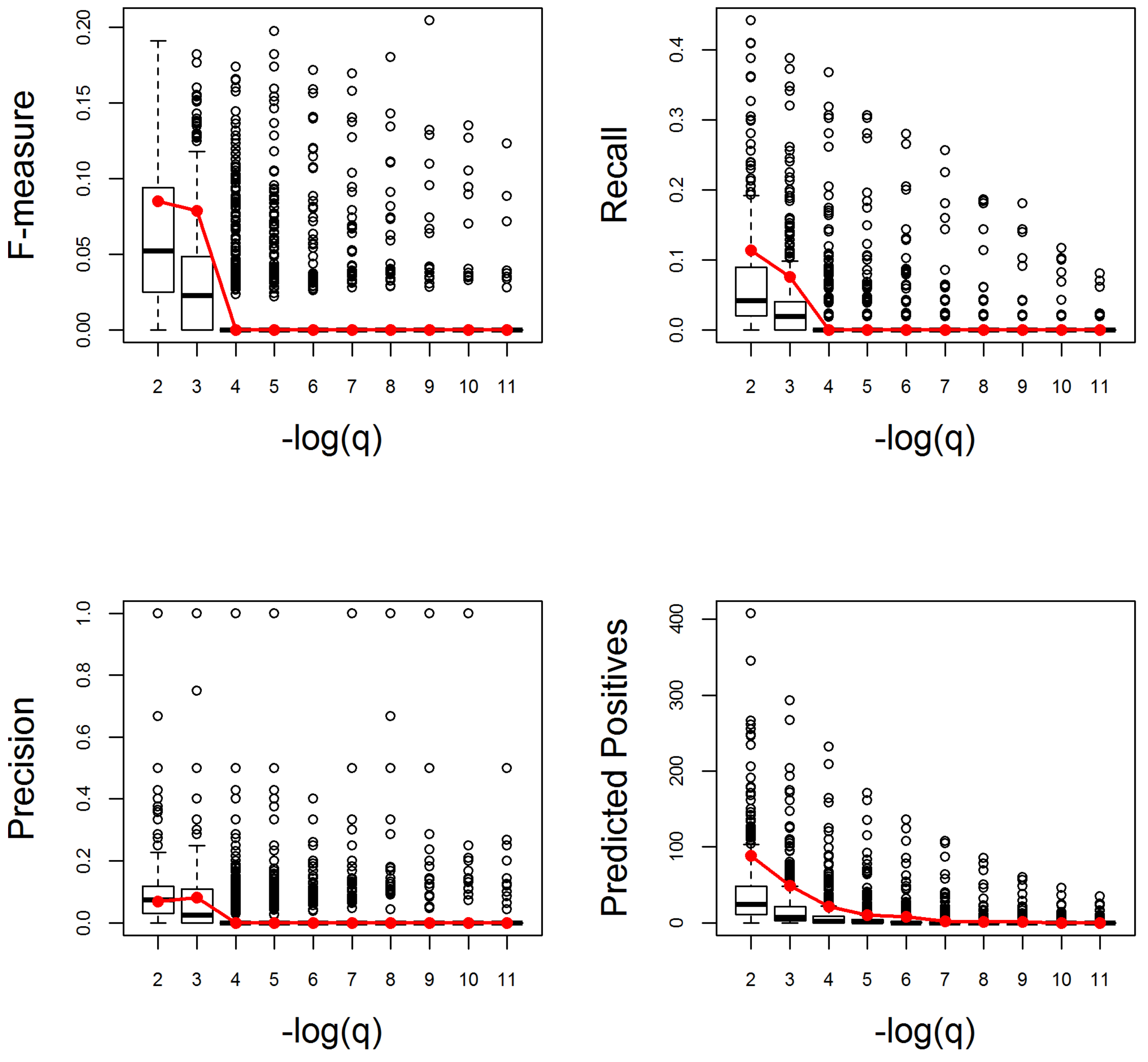

Appendix Figure S14. (continued)

Plastid organization. Average

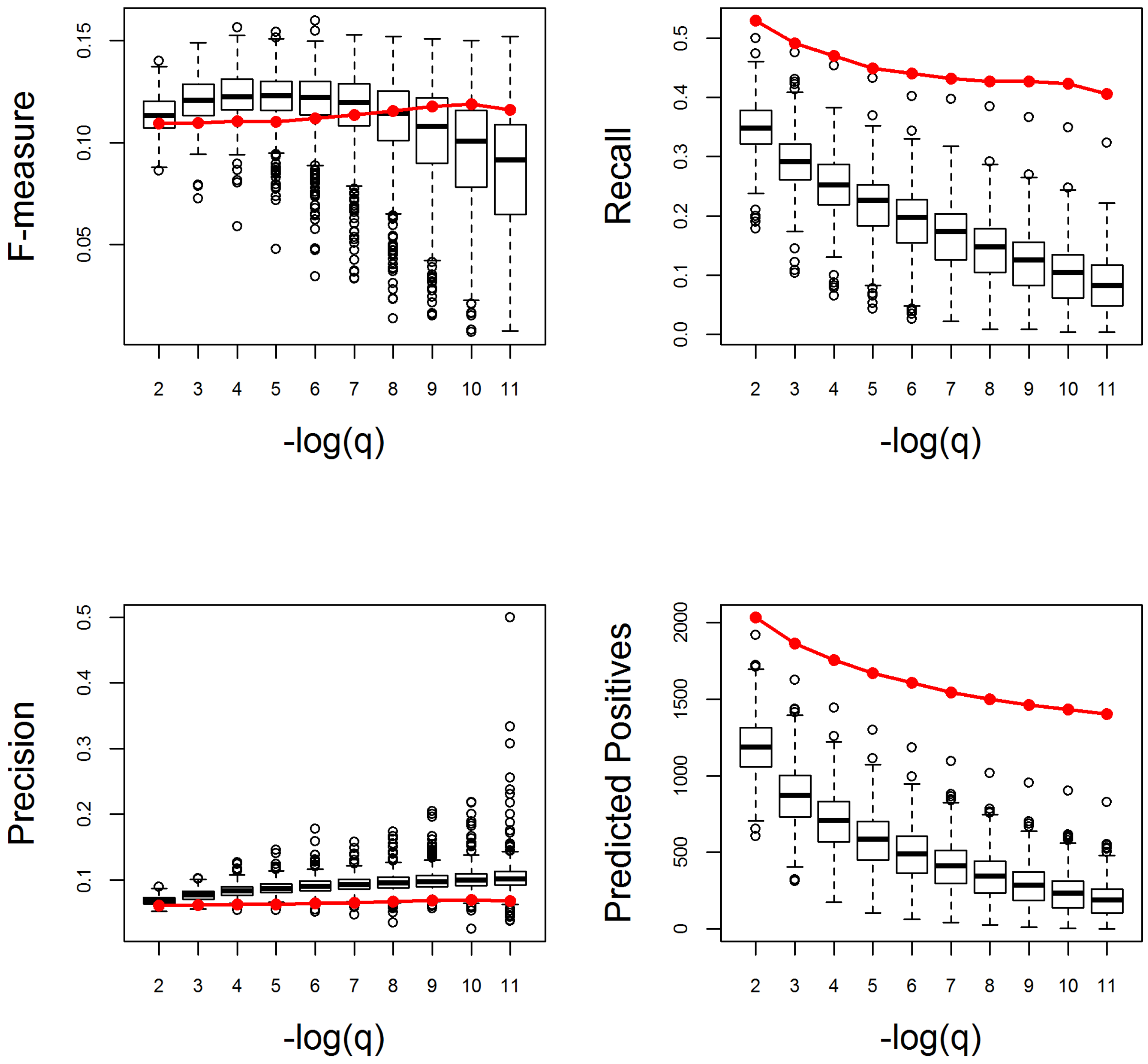

Appendix Figure S14. (continued)

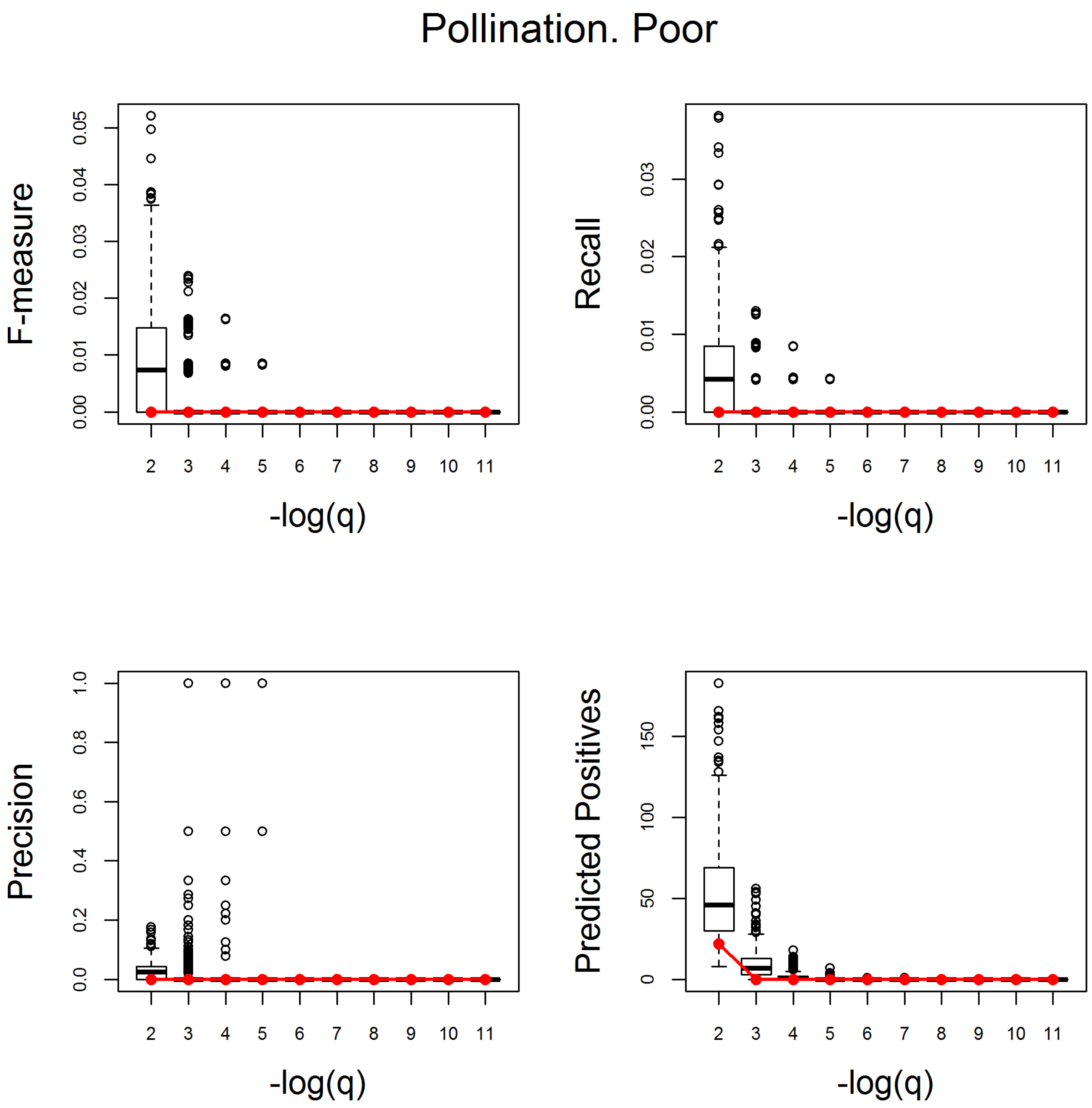

Appendix Figure S14. (continued)

Polysaccharide biosynthetic process. Very Poor

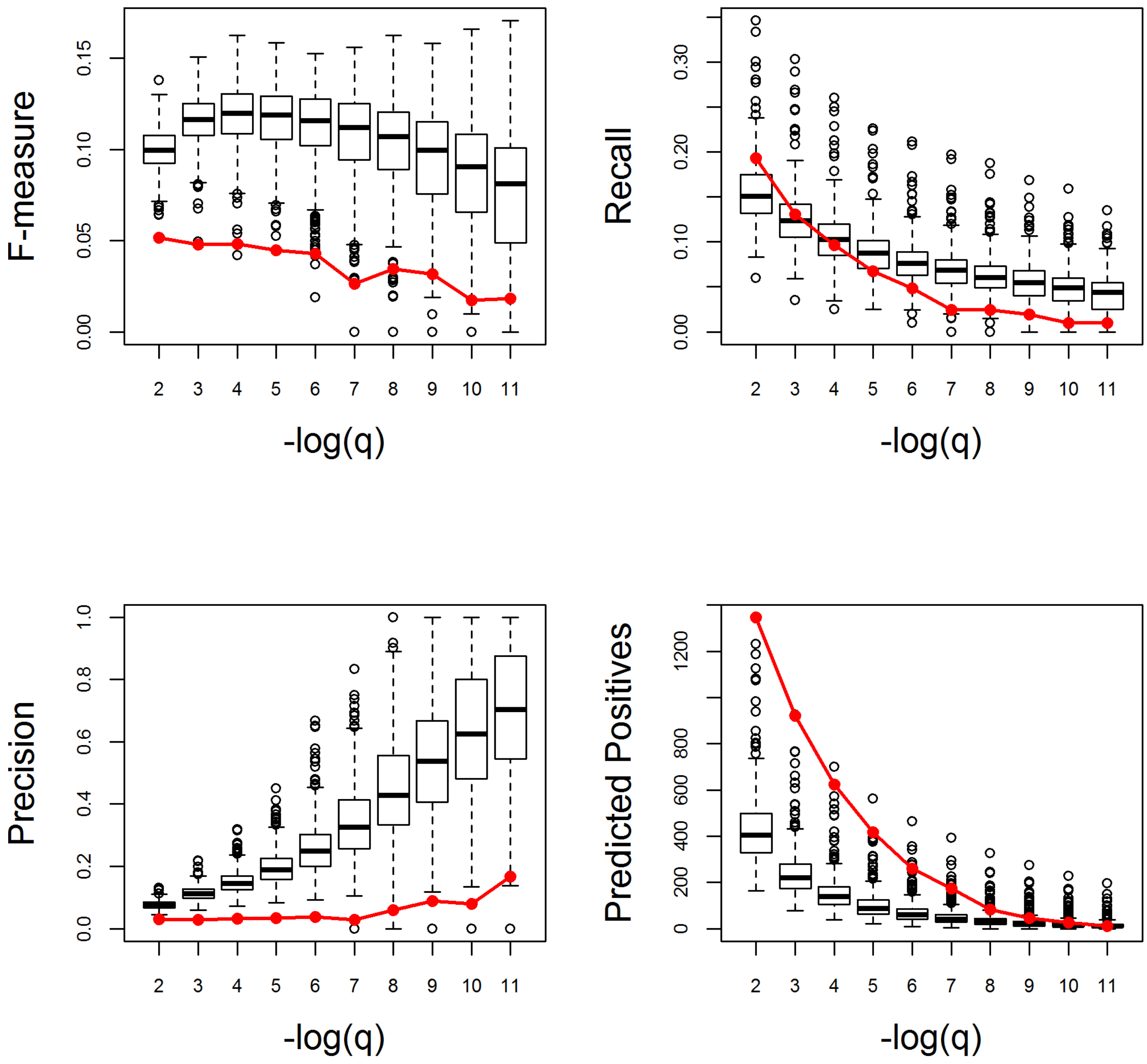

Appendix Figure S14. (continued)

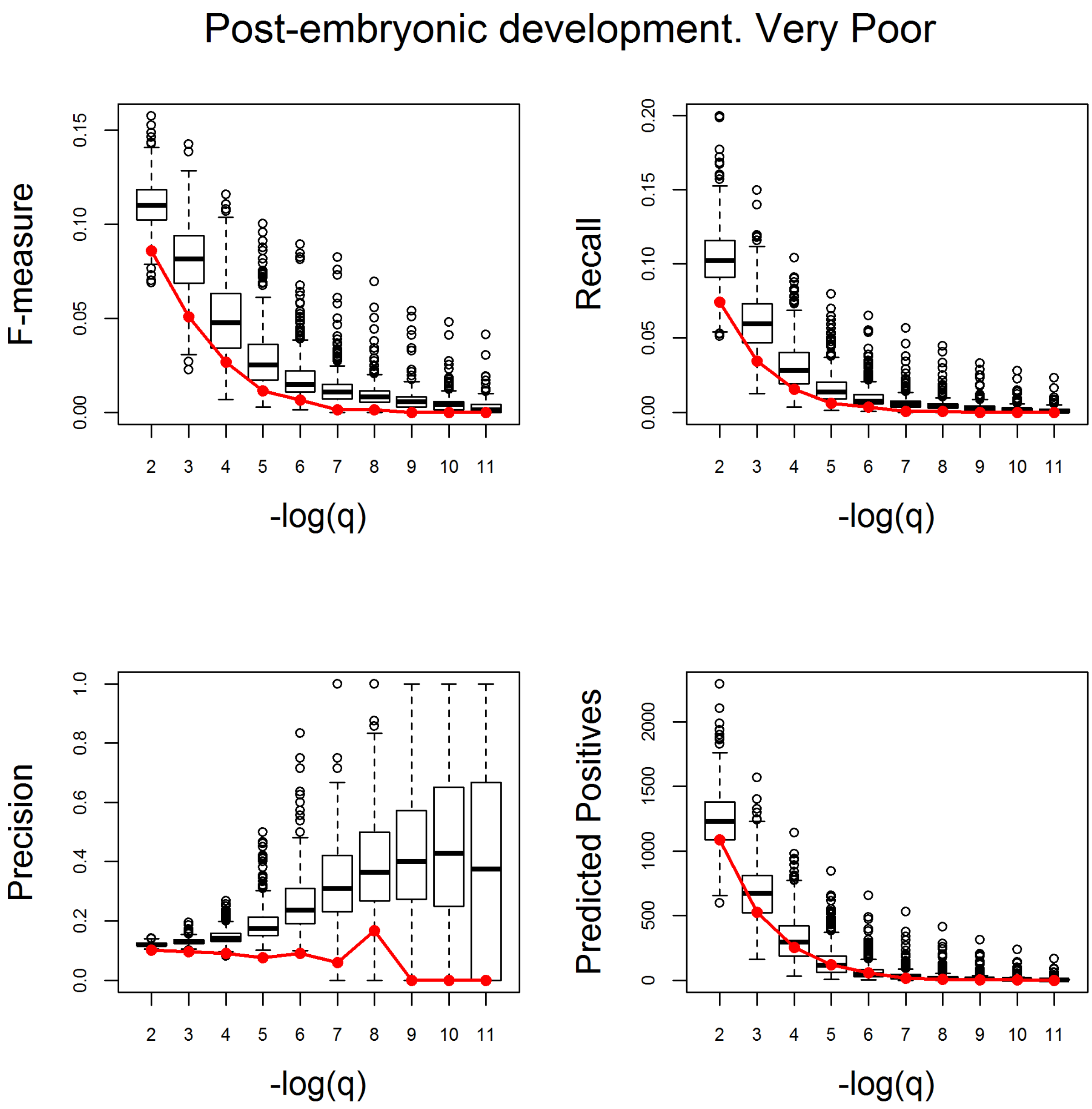

Appendix Figure S14. (continued)

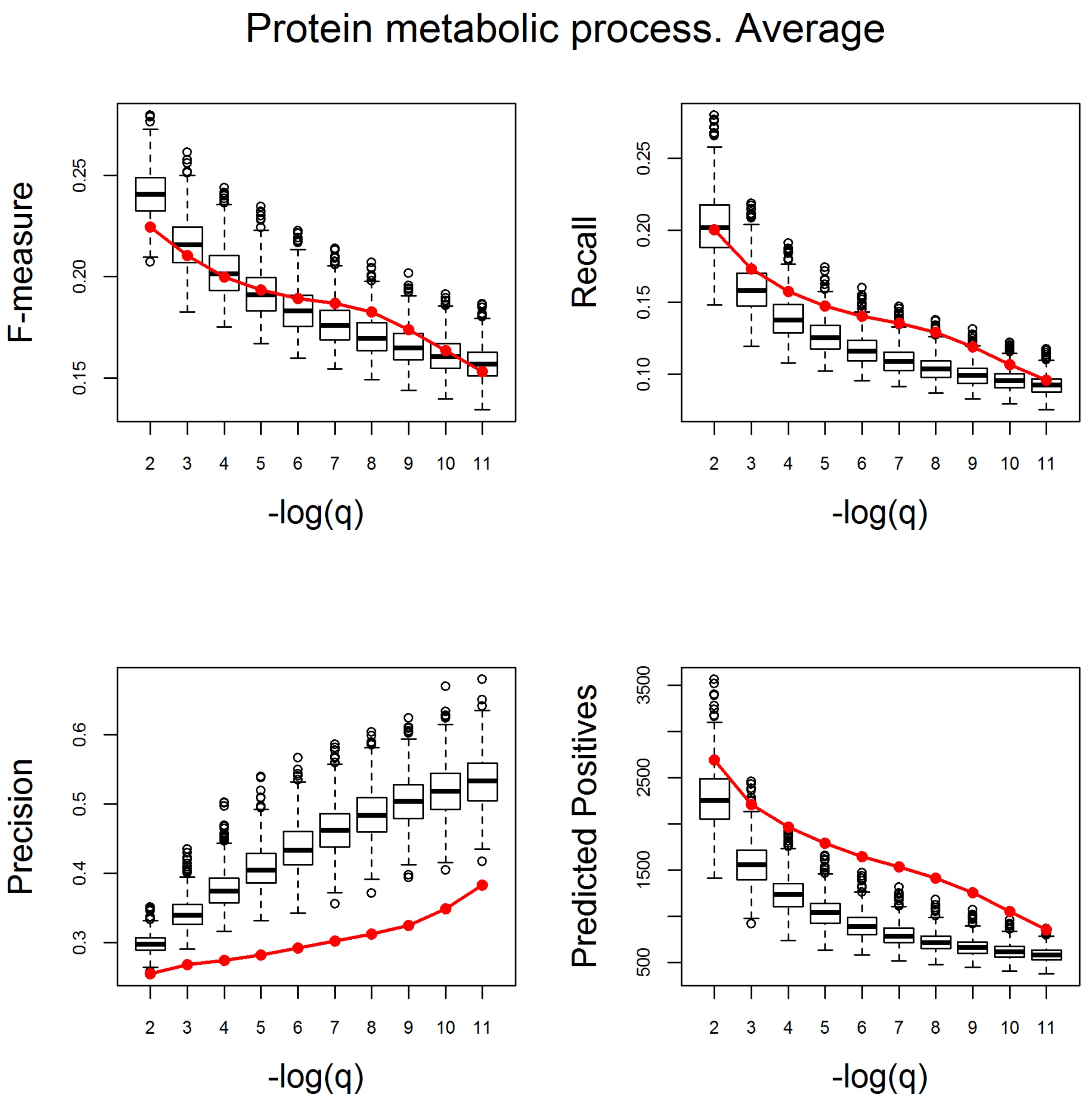

Appendix Figure S14. (continued)

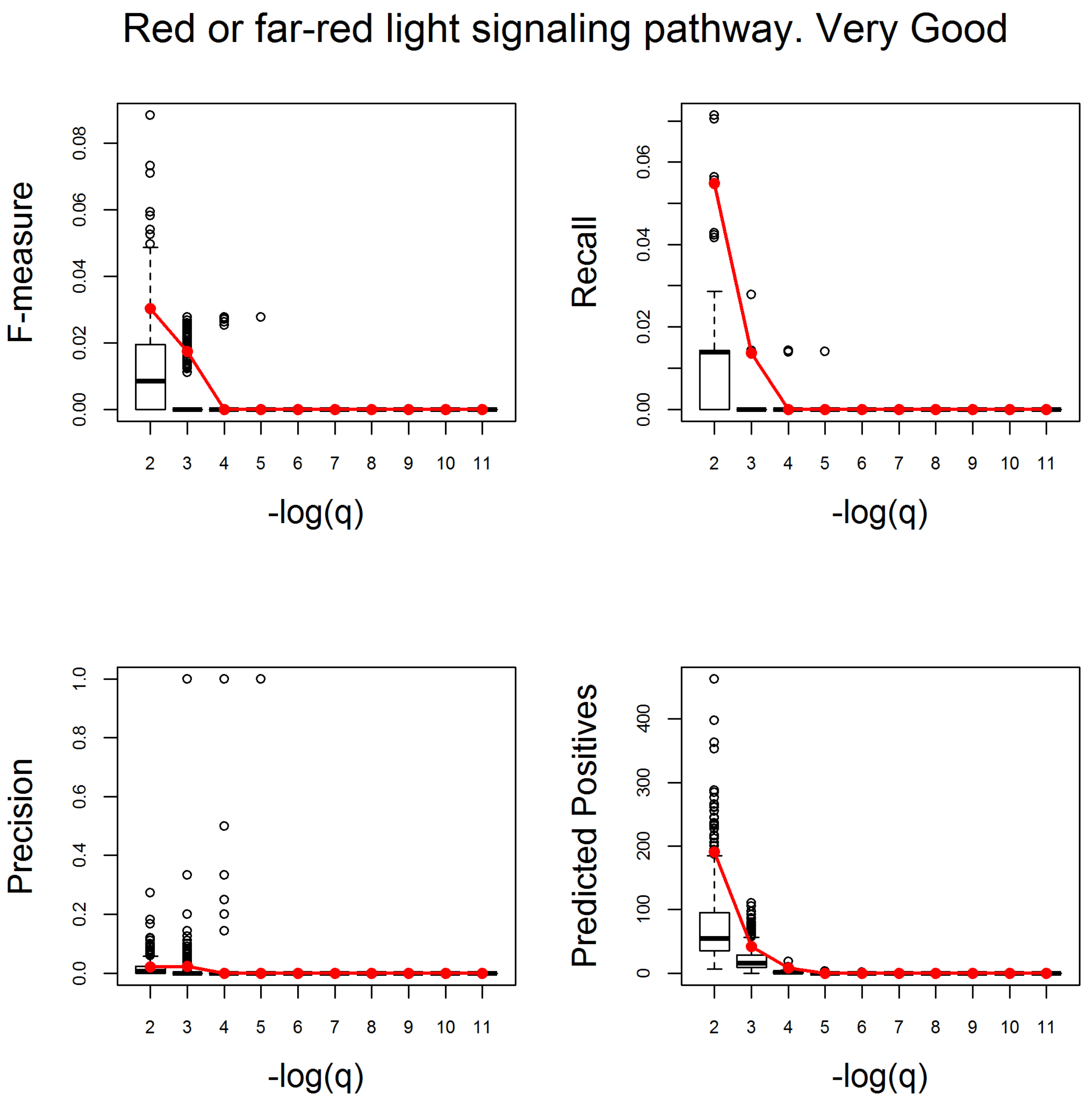

Appendix Figure S14. (continued)

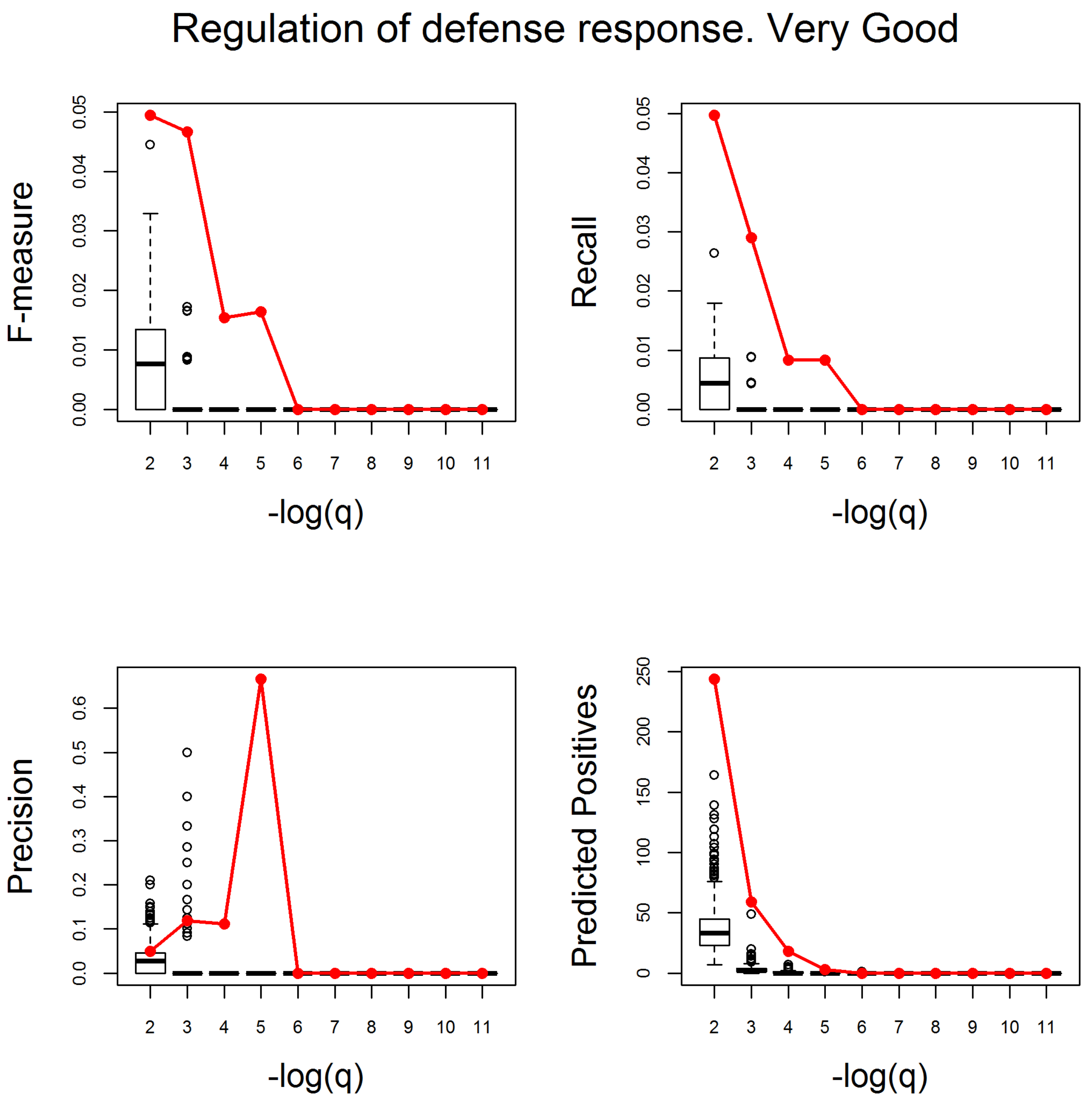

Appendix Figure S14. (continued)

Regulation of gene expression, epigenetic. Very Poor

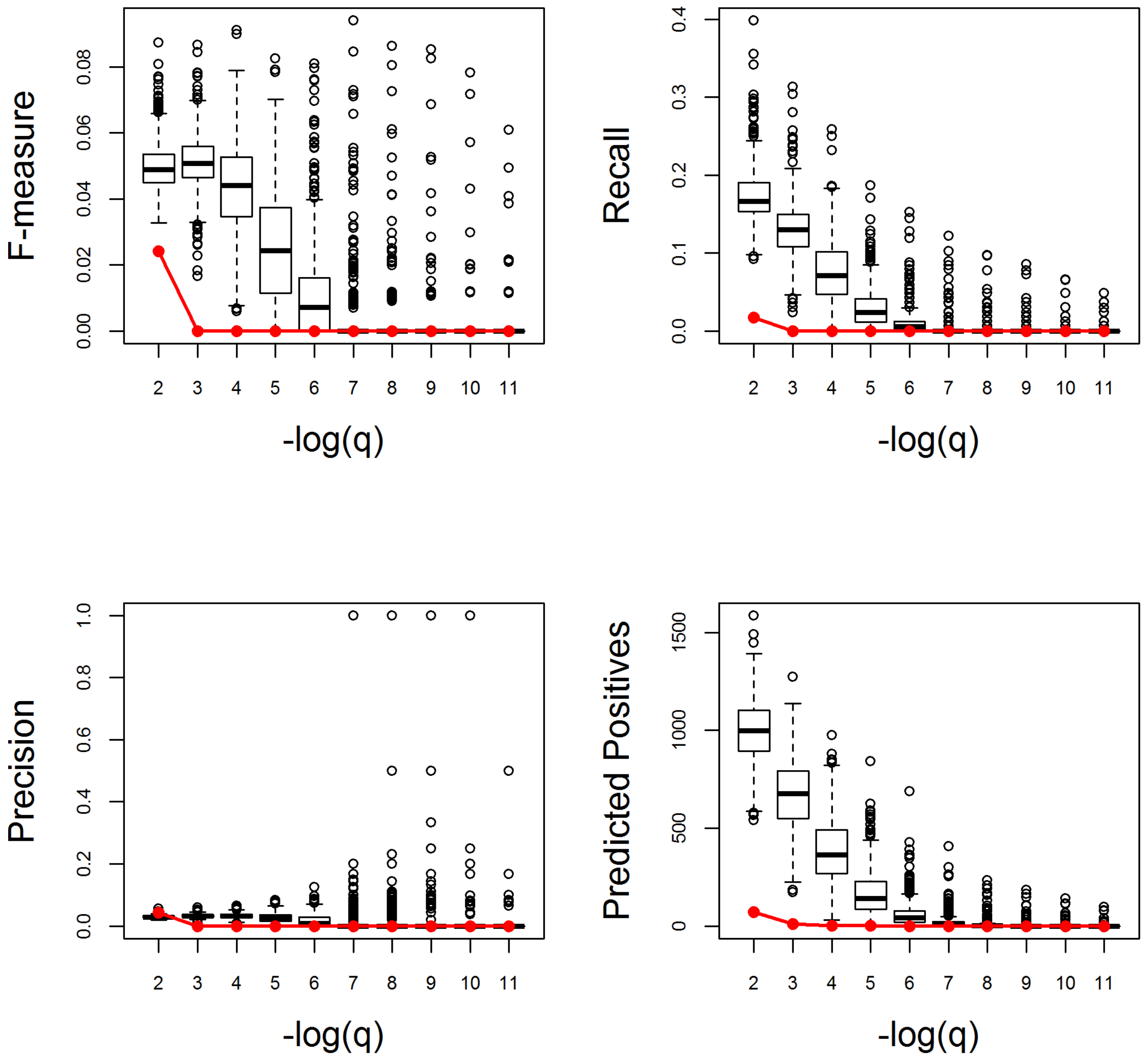

Appendix Figure S14. (continued)

Regulation of response to osmotic stress. Very Good

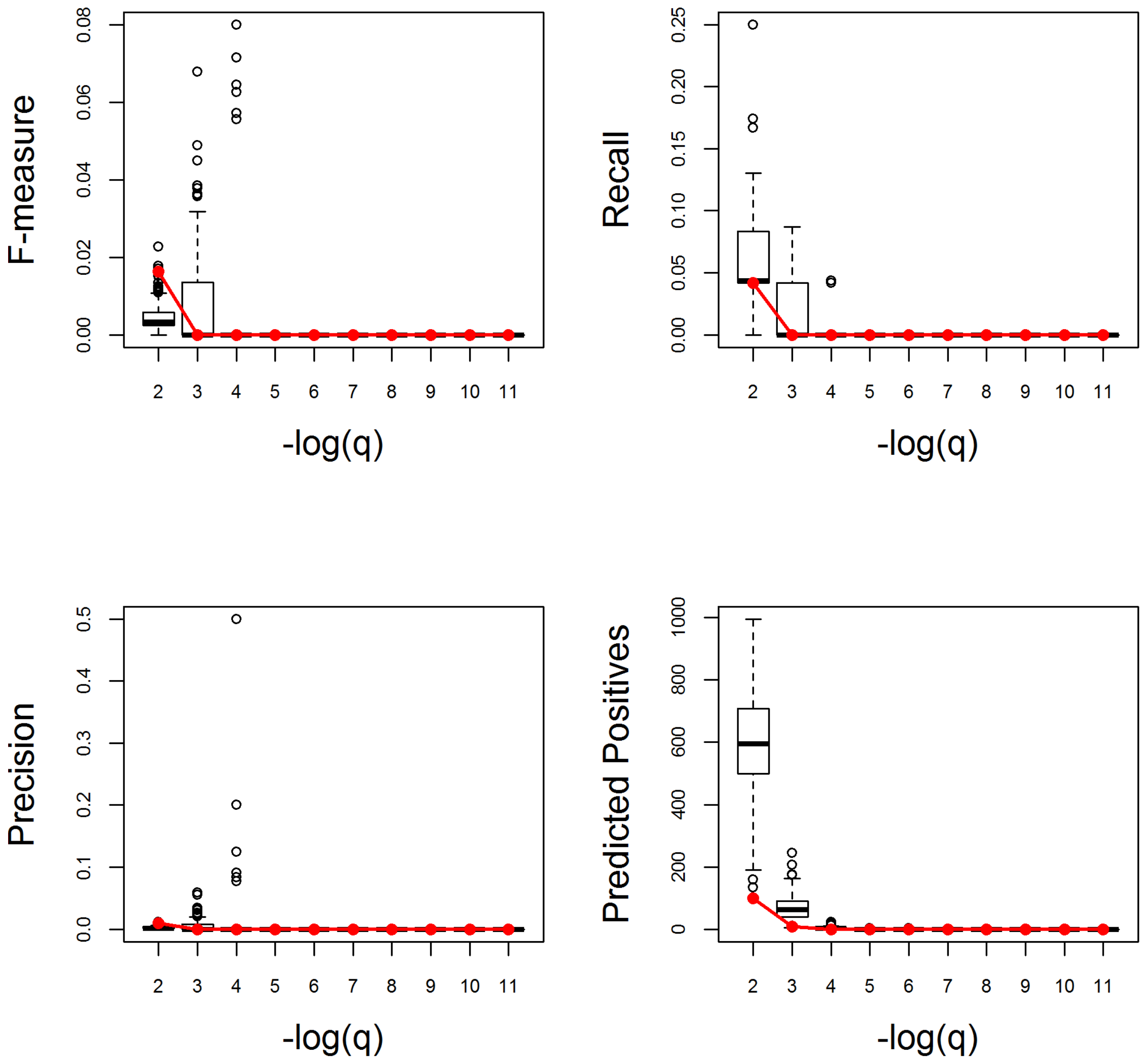

Appendix Figure S14. (continued)

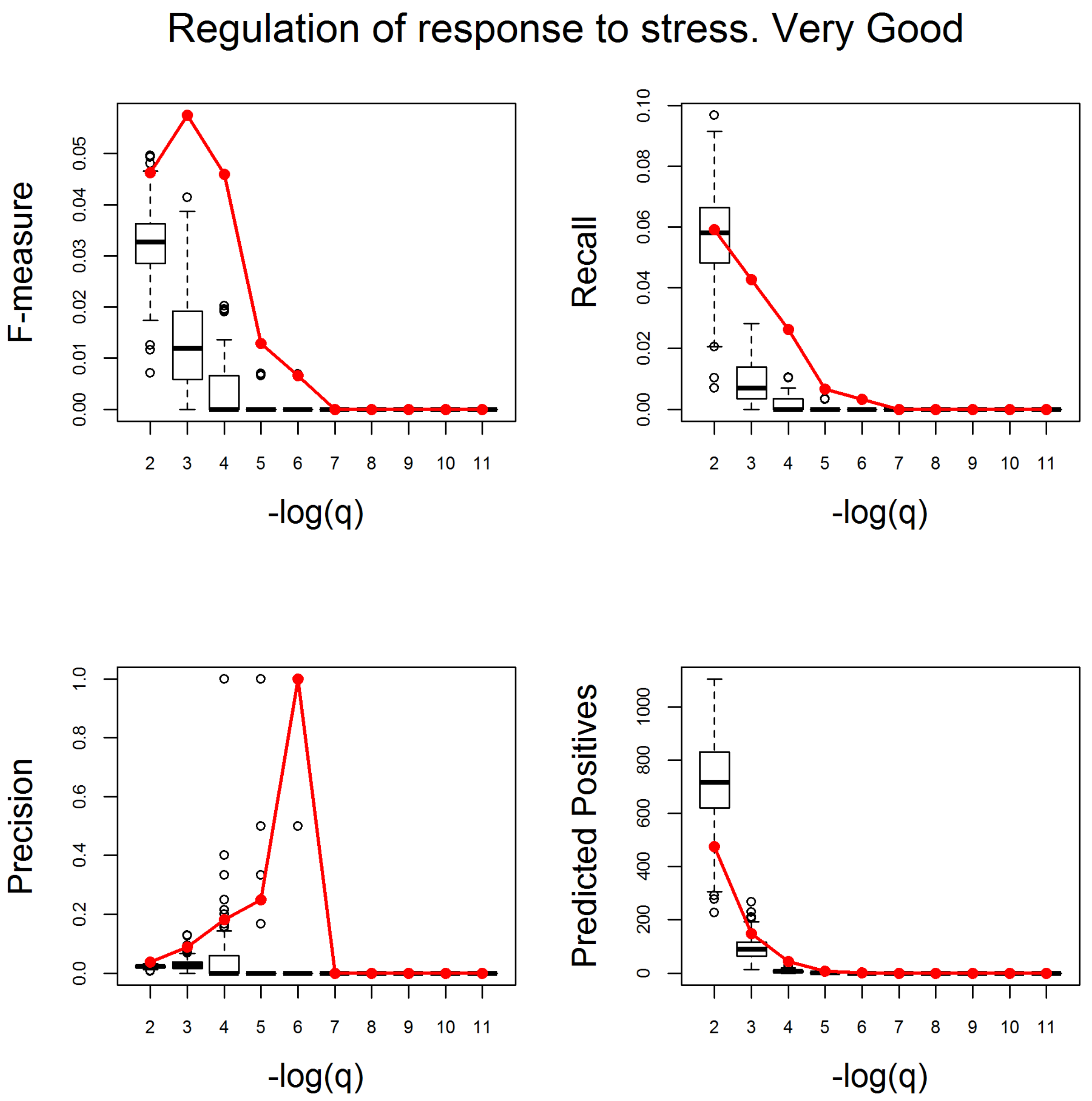

Appendix Figure S14. (continued)

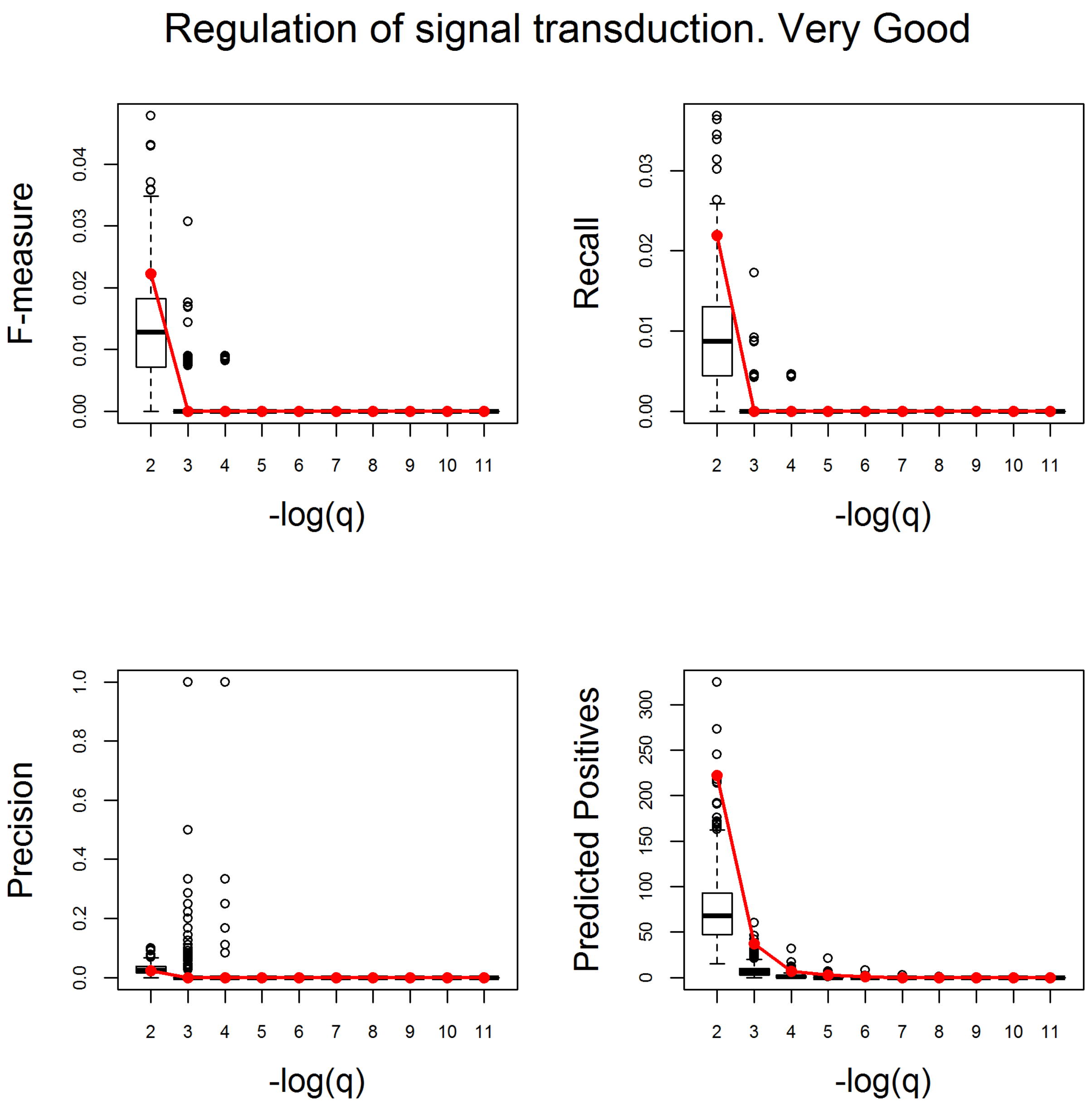

Appendix Figure S14. (continued)

Reproduction. Very Poor

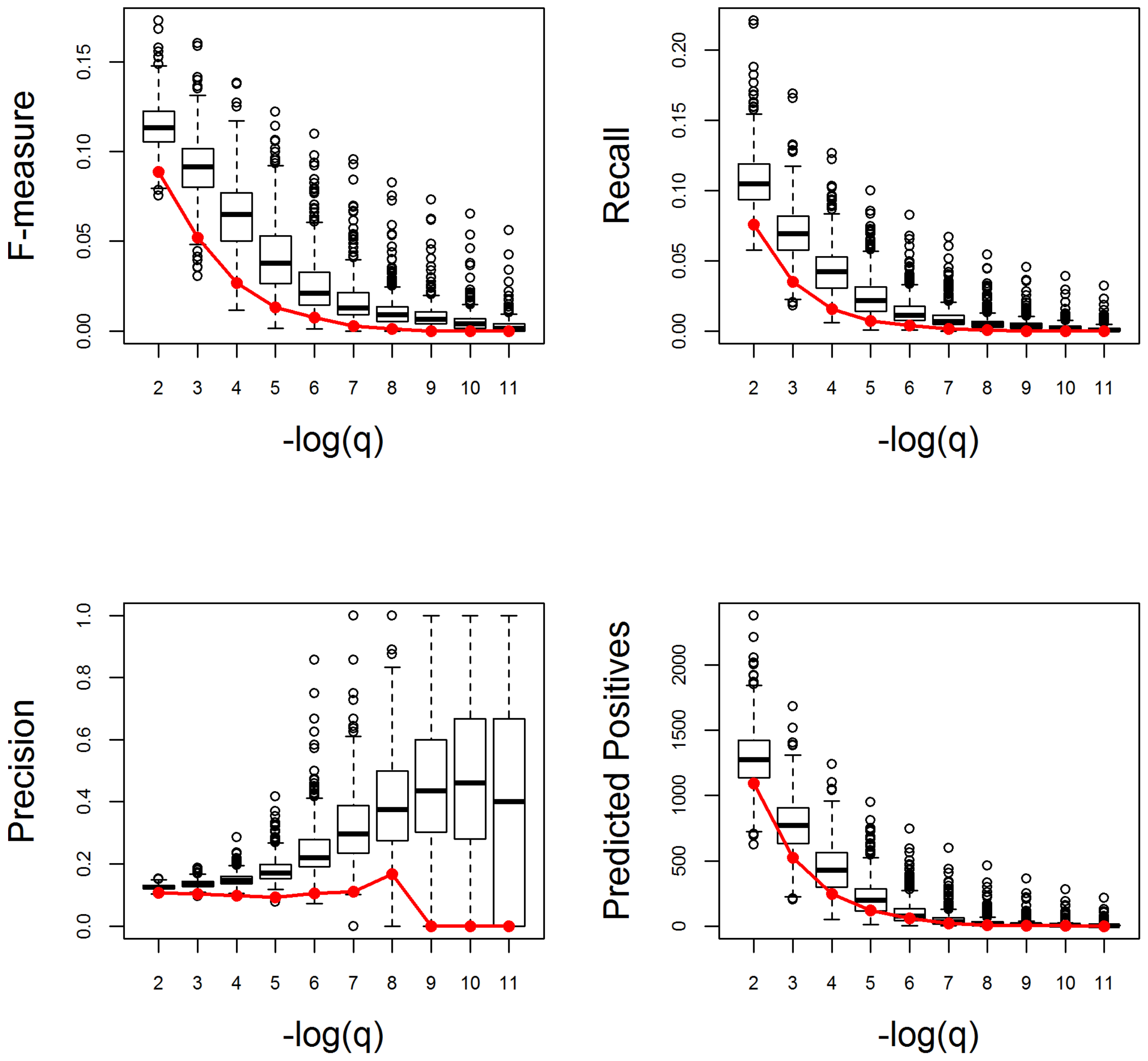

Appendix Figure S14. (continued)

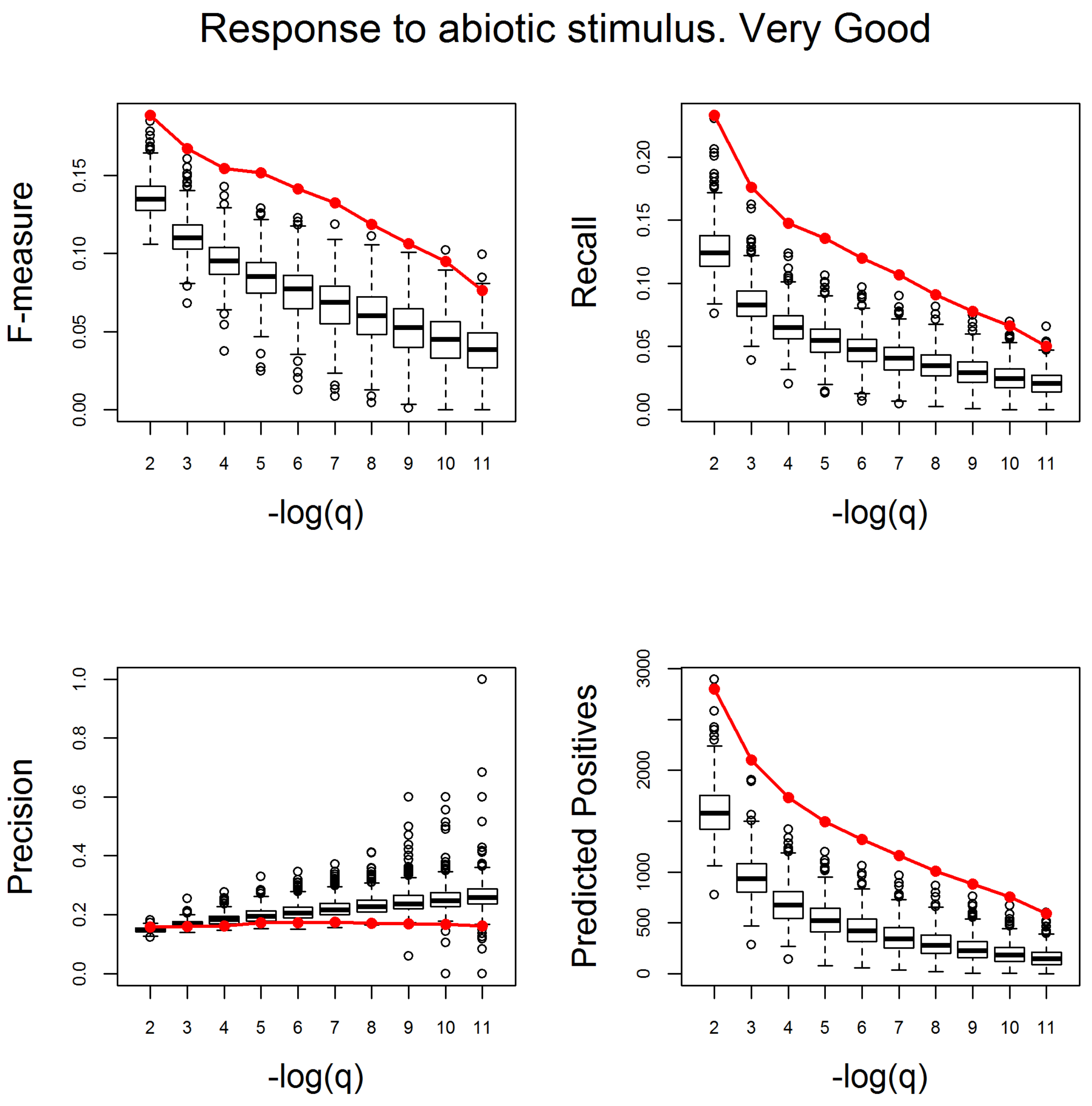

Appendix Figure S14. (continued)

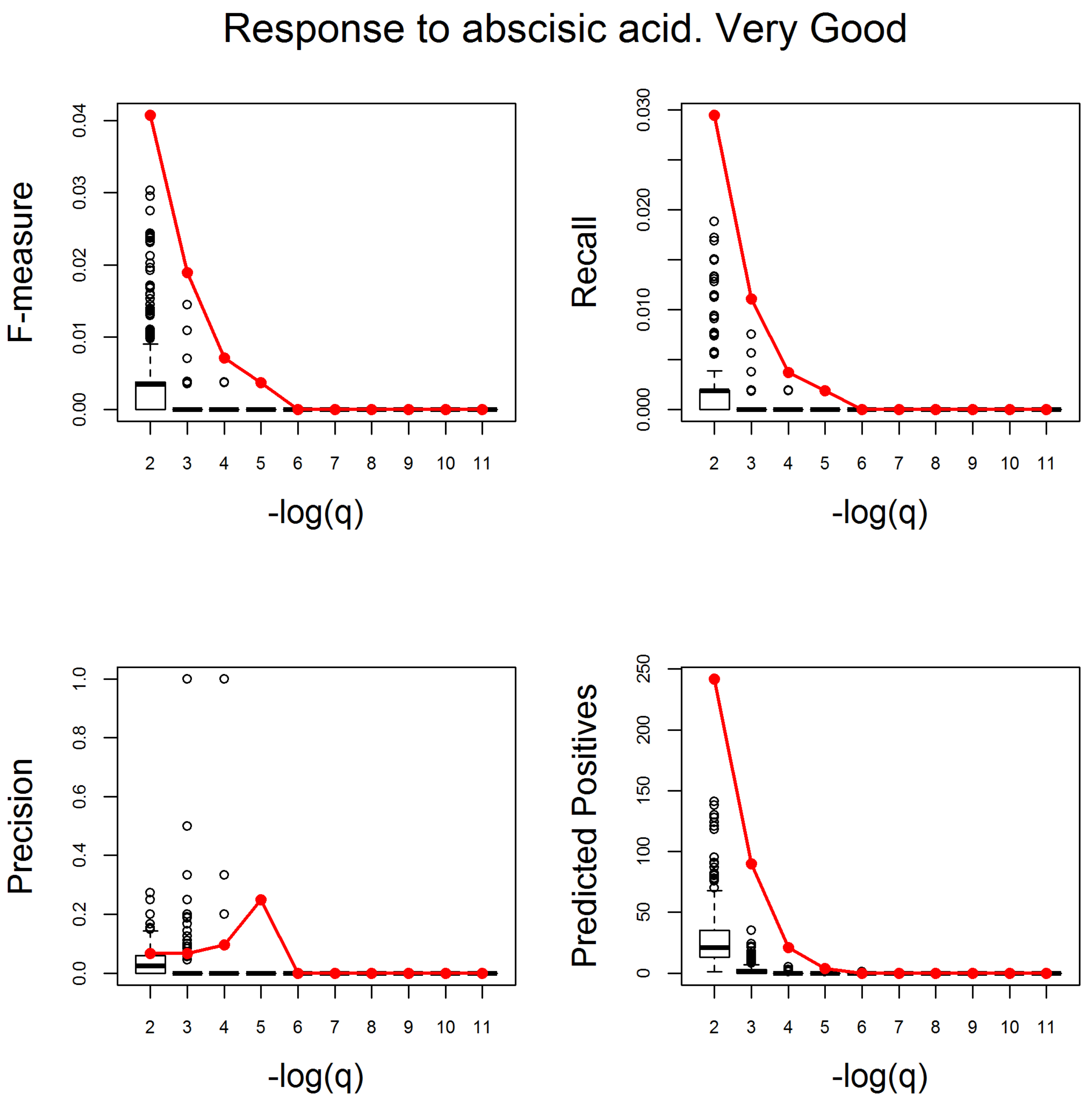

Appendix Figure S14. (continued)

Response to acid chemical. Very Good

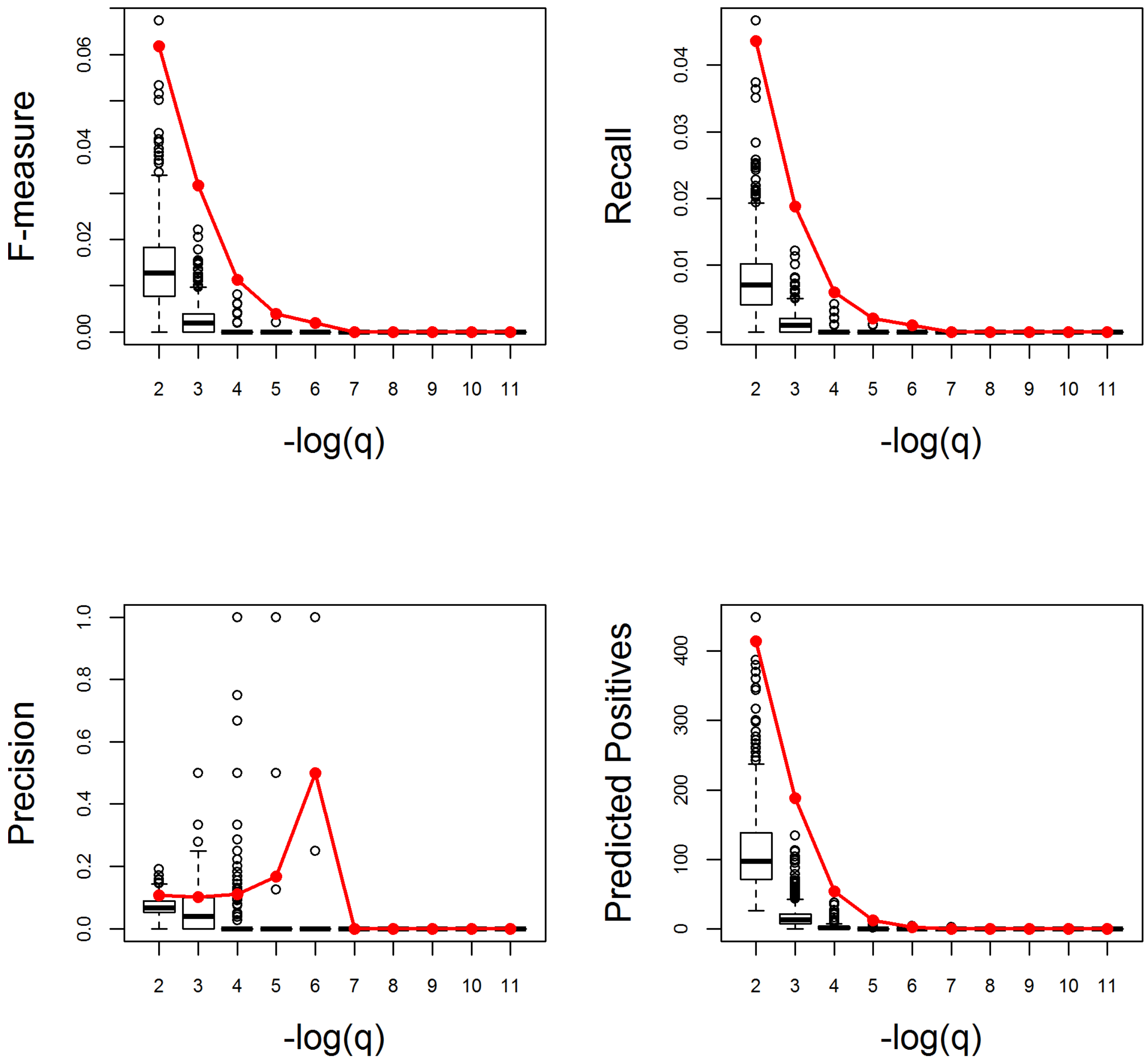

Appendix Figure S14. (continued)

Response to auxin. Very Poor

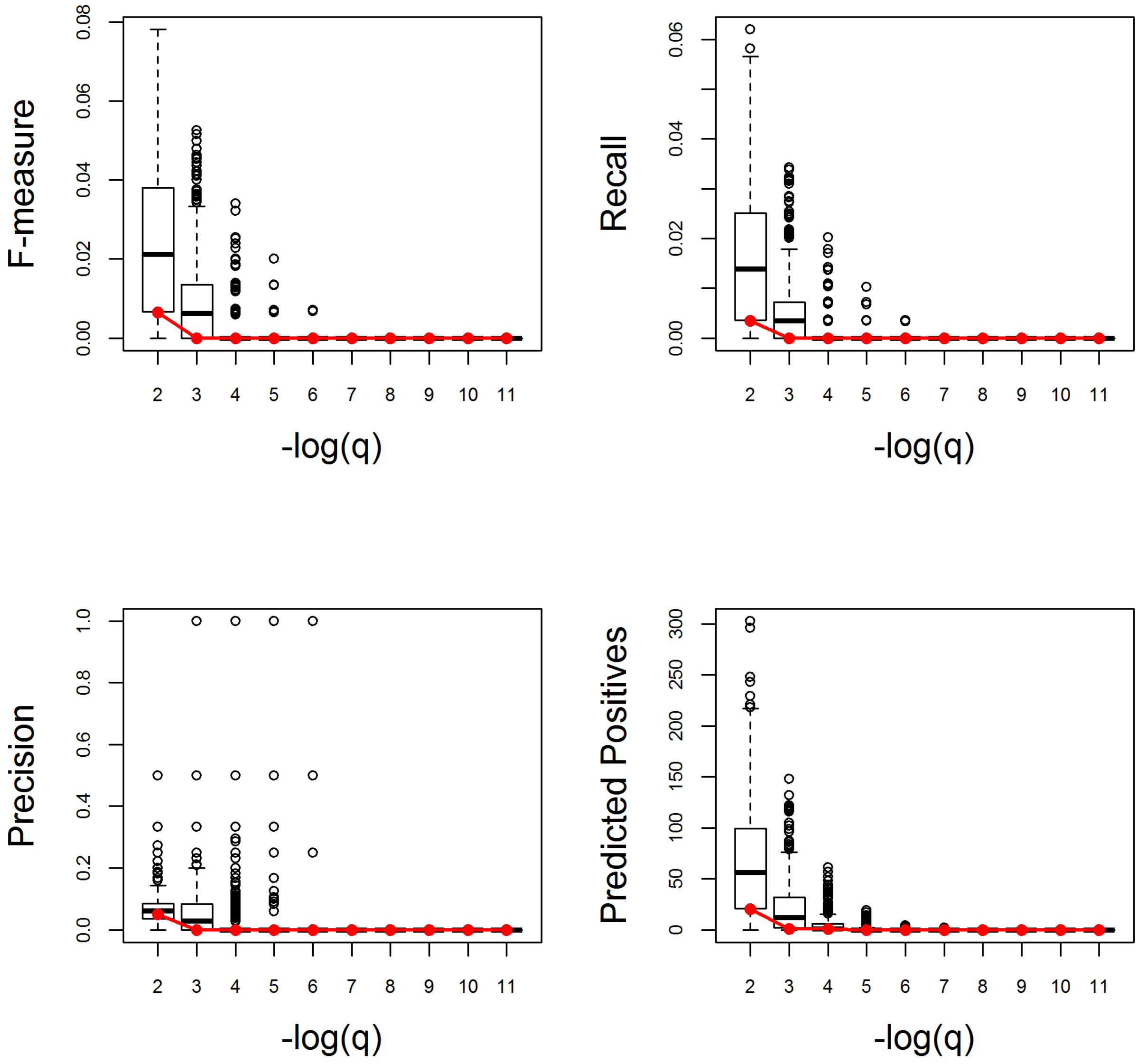

Appendix Figure S14. (continued)

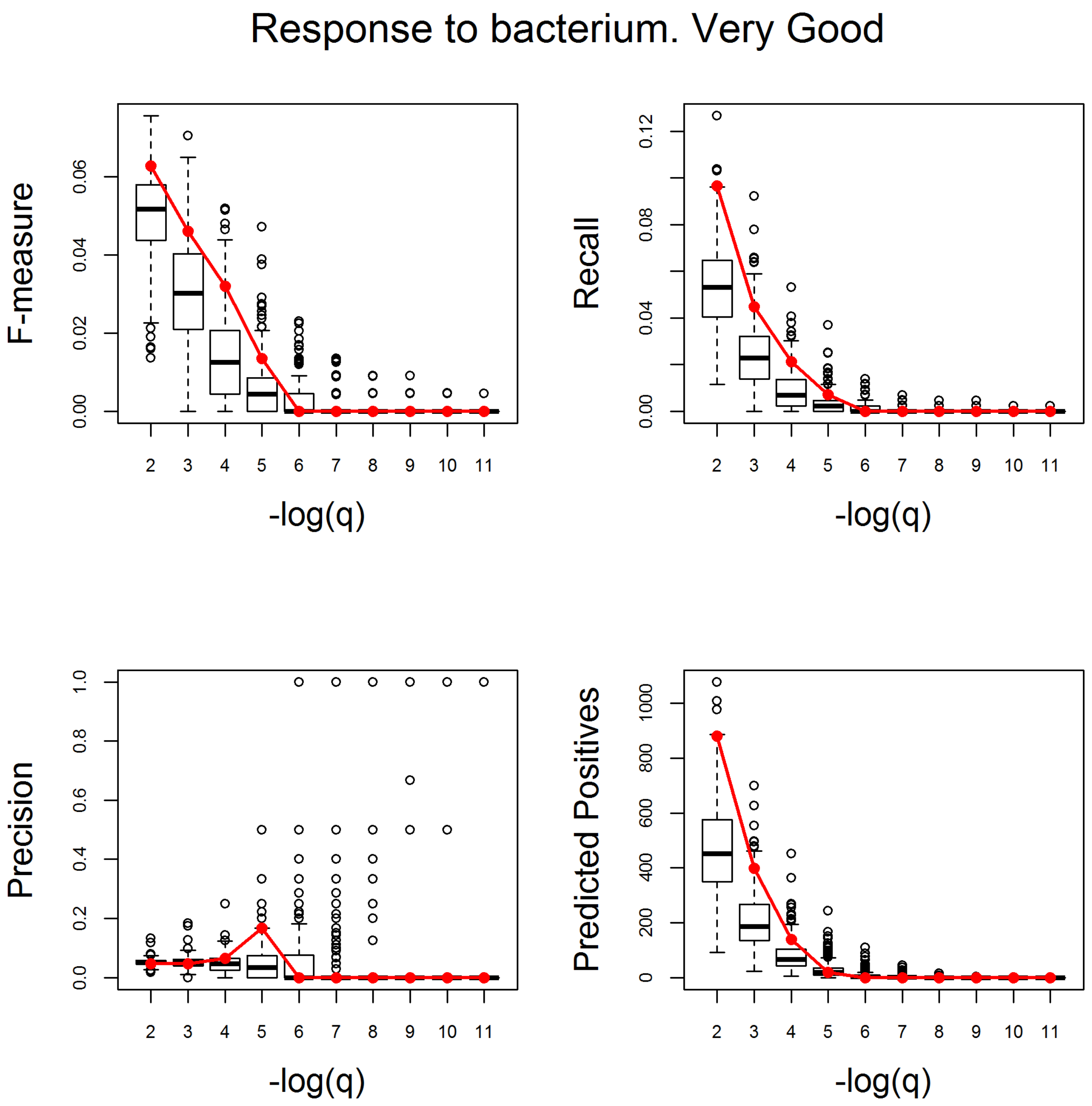

Appendix Figure S14. (continued)

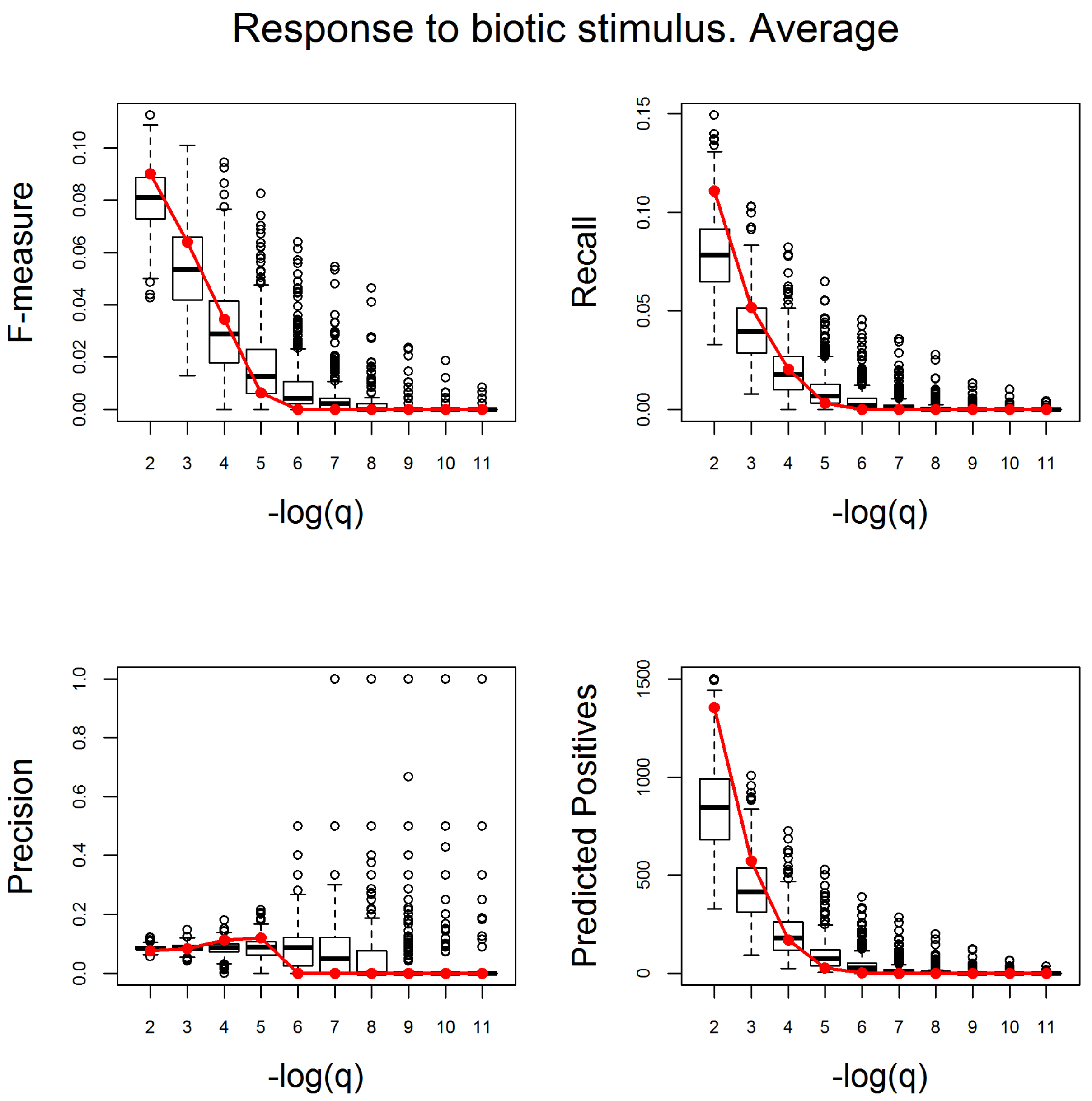

Appendix Figure S14. (continued)

Response to blue light. Average

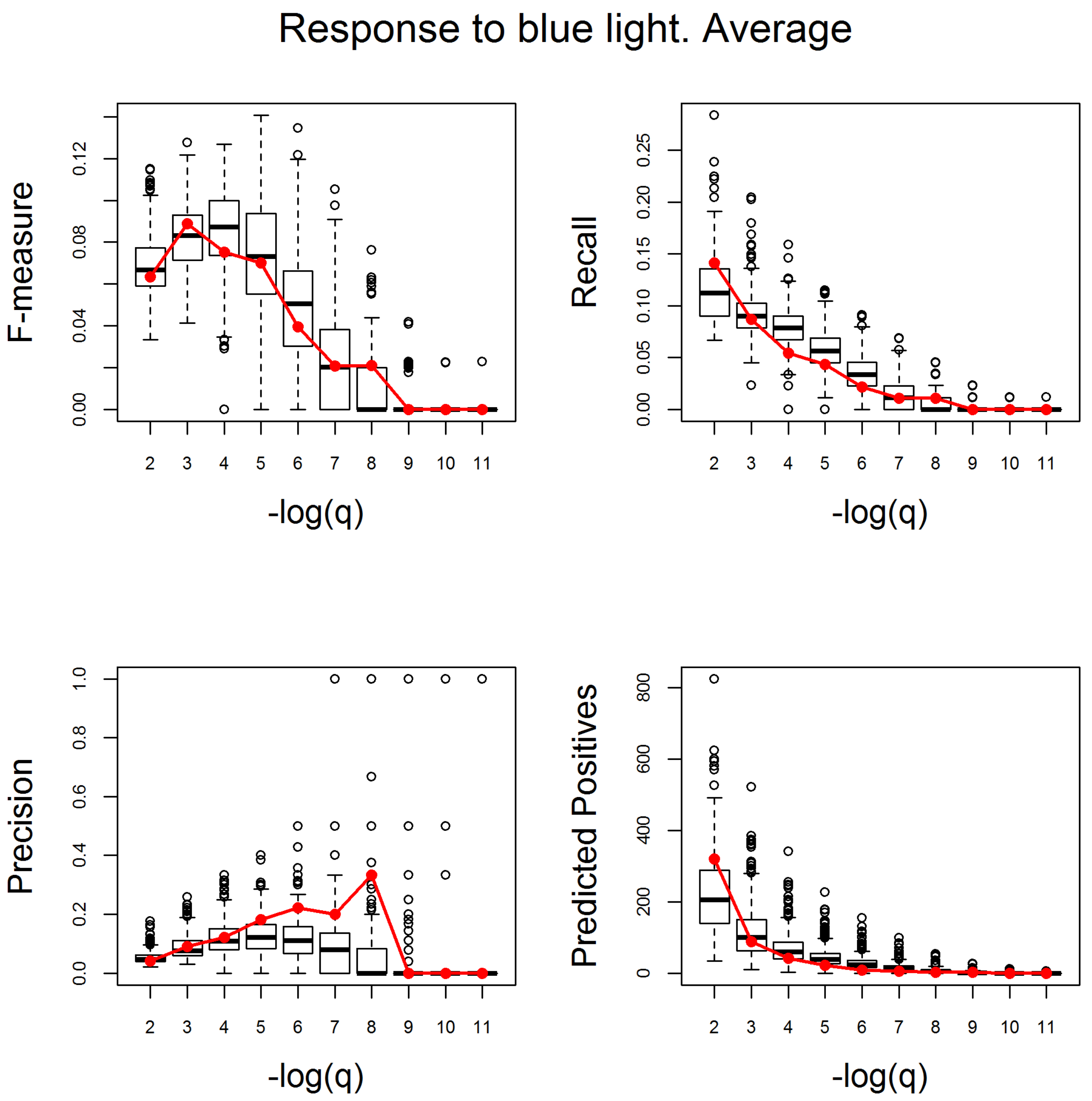

Appendix Figure S14. (continued)

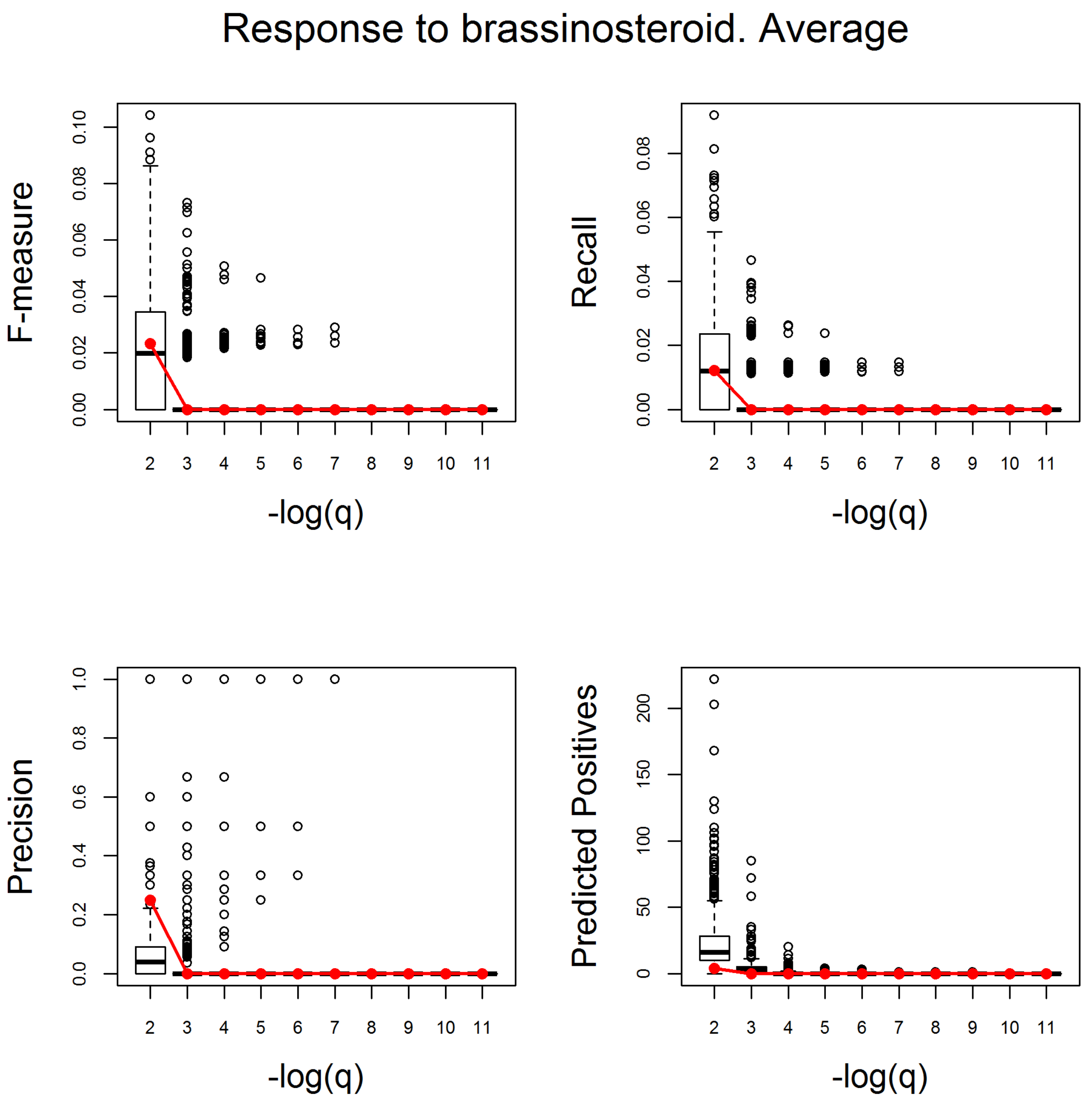

Appendix Figure S14. (continued)

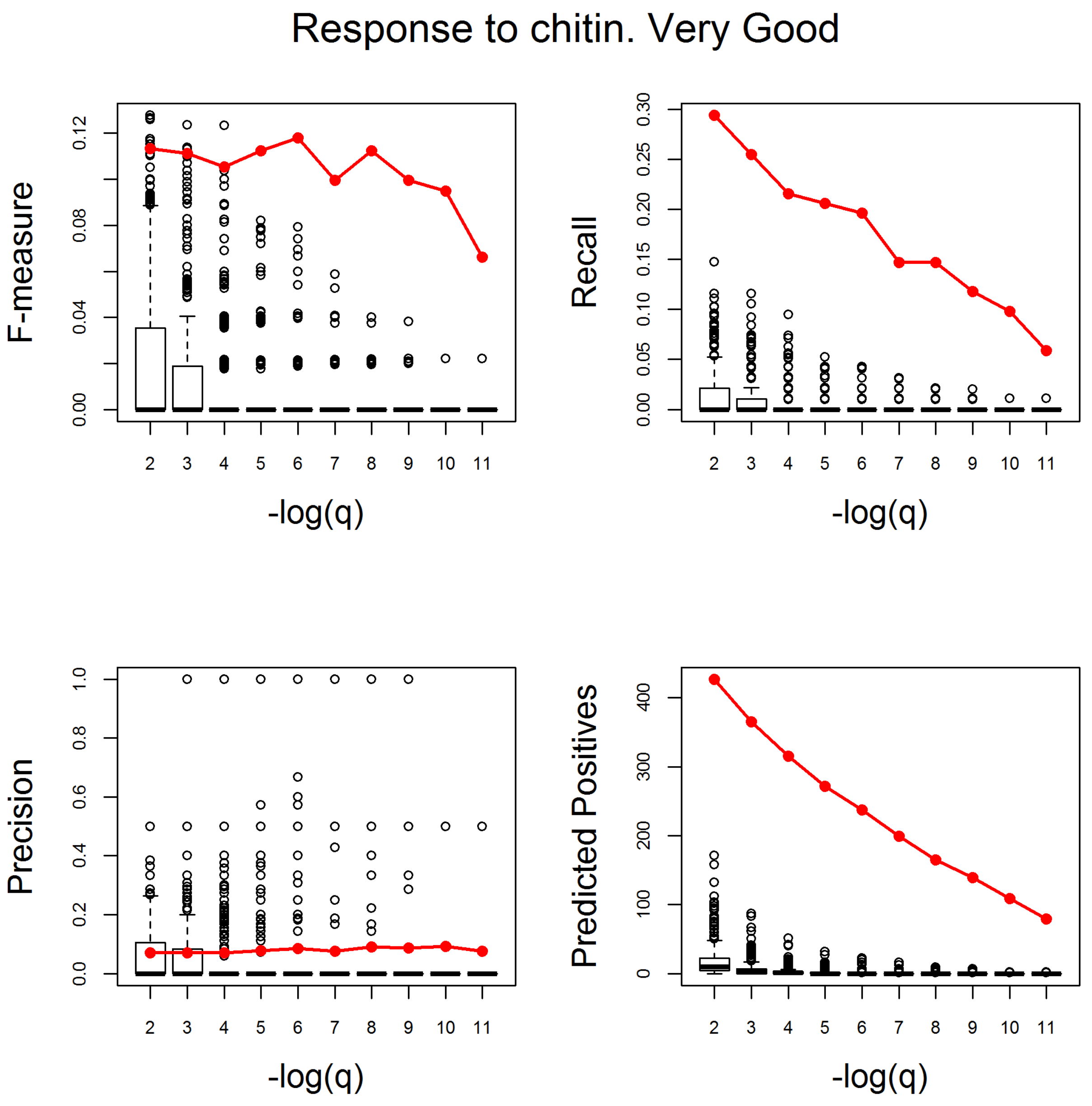

Appendix Figure S14. (continued)

Response to cold. Very Good

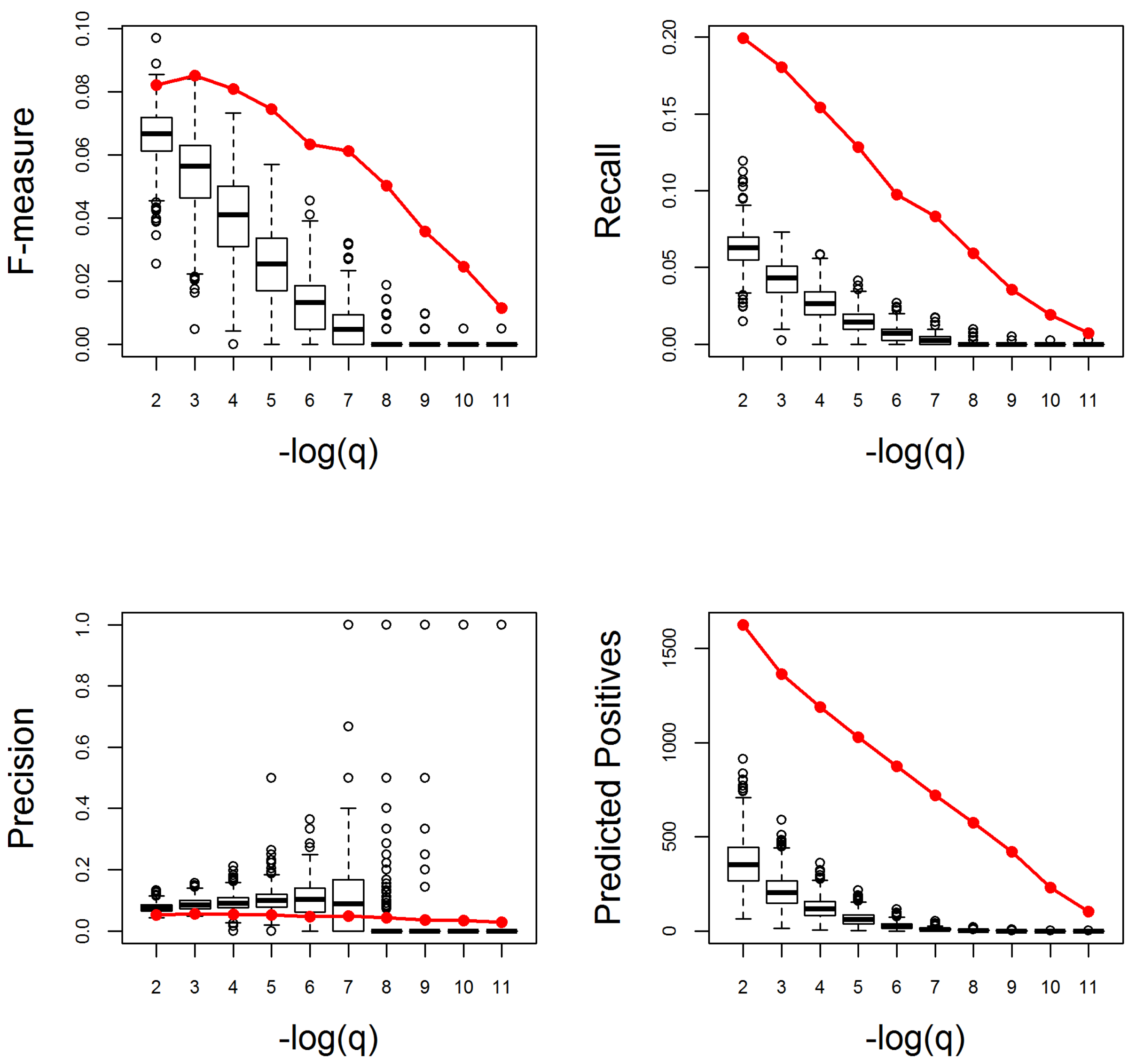

Appendix Figure S14. (continued)

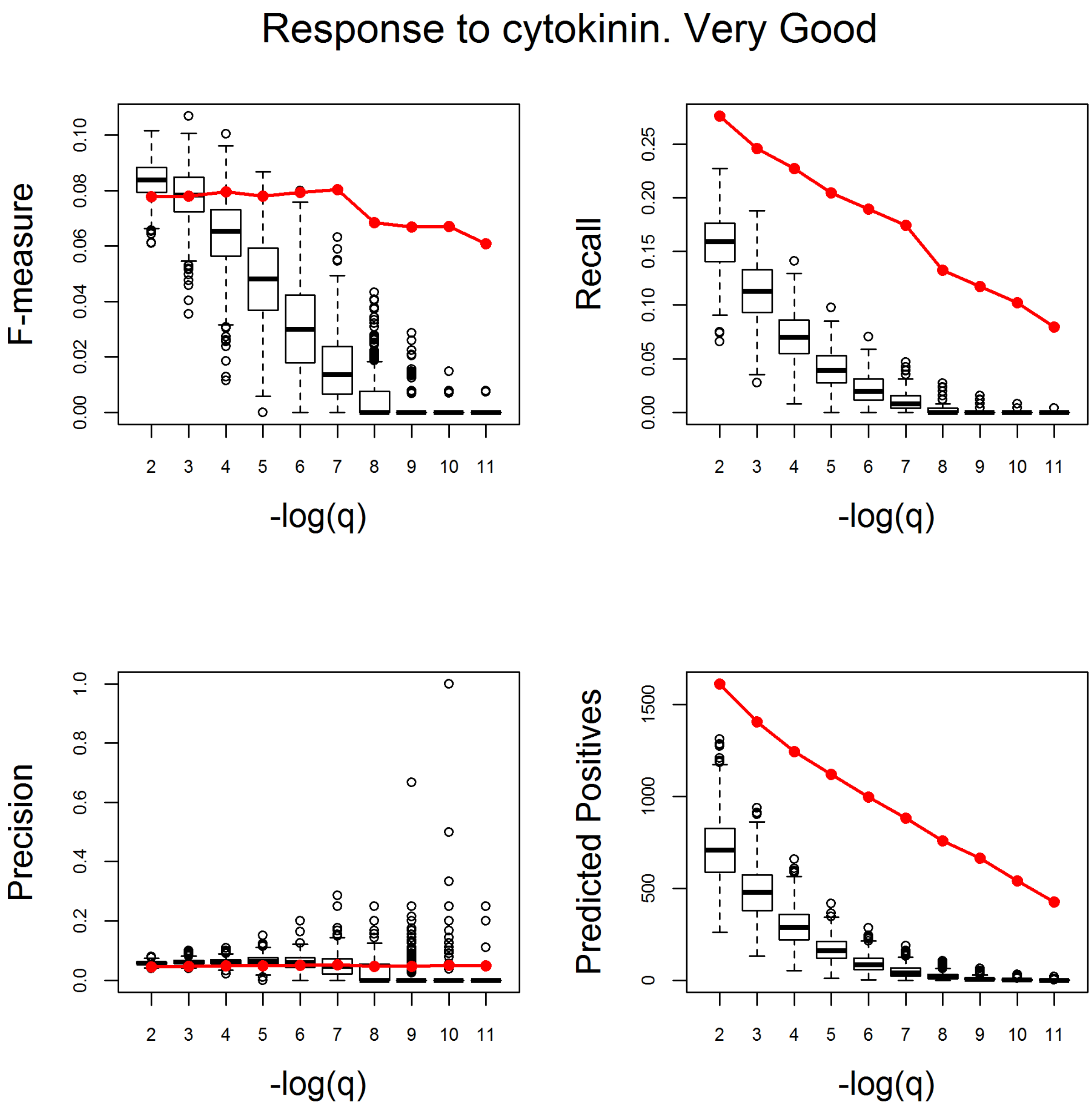

Appendix Figure S14. (continued)

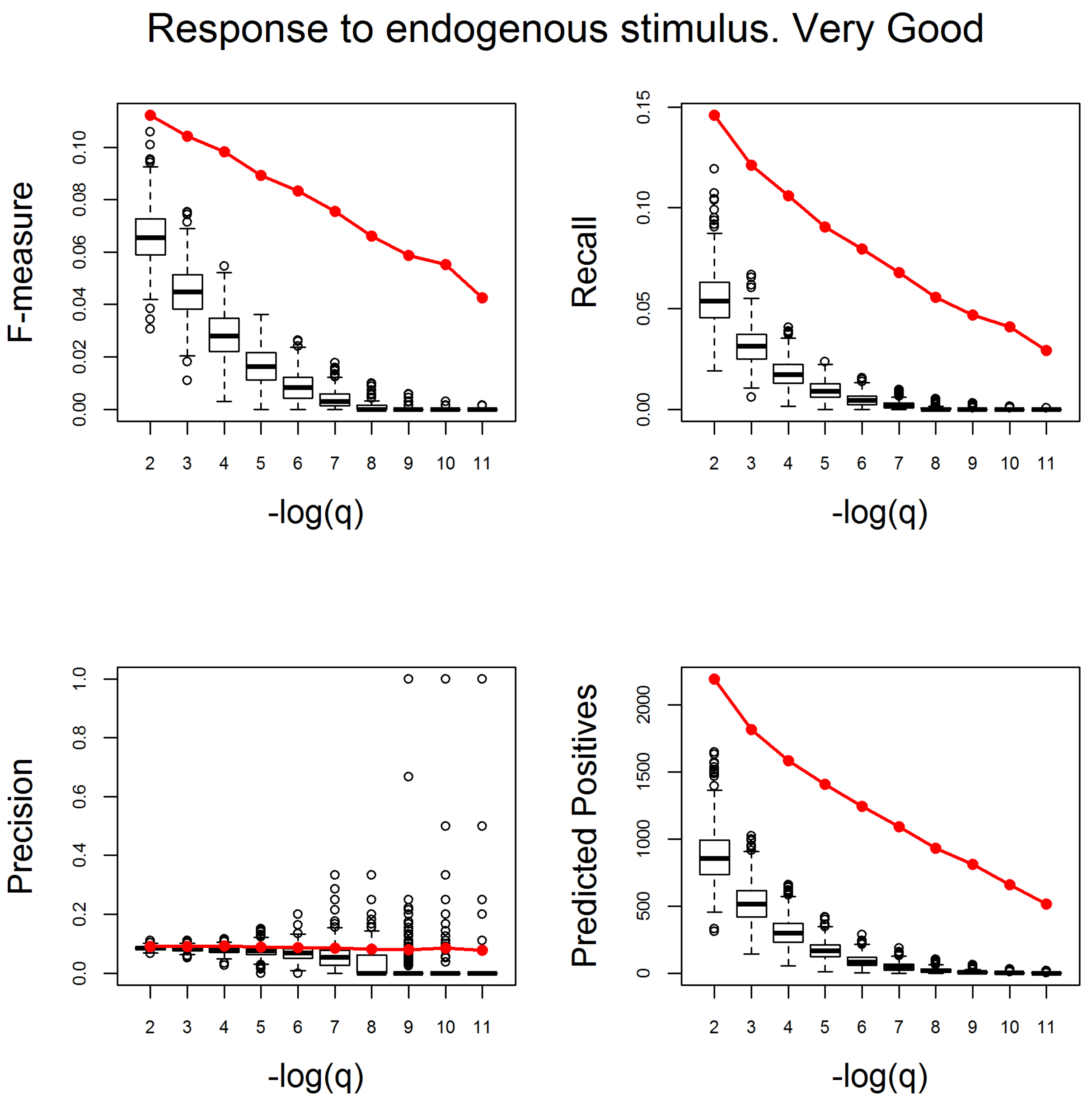

Appendix Figure S14. (continued)

Response to endoplasmic reticulum stress. Very Good

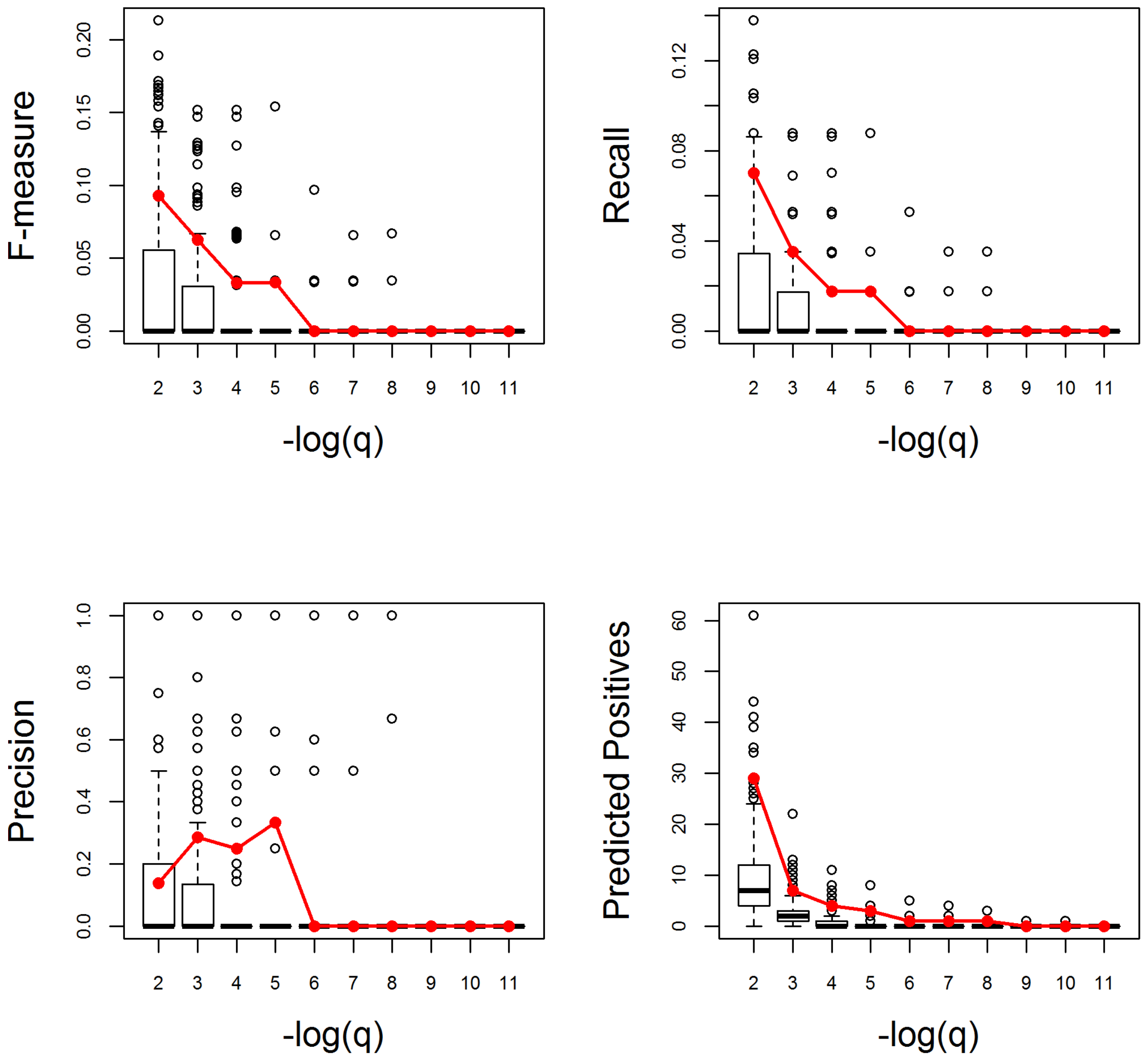

Appendix Figure S14. (continued)

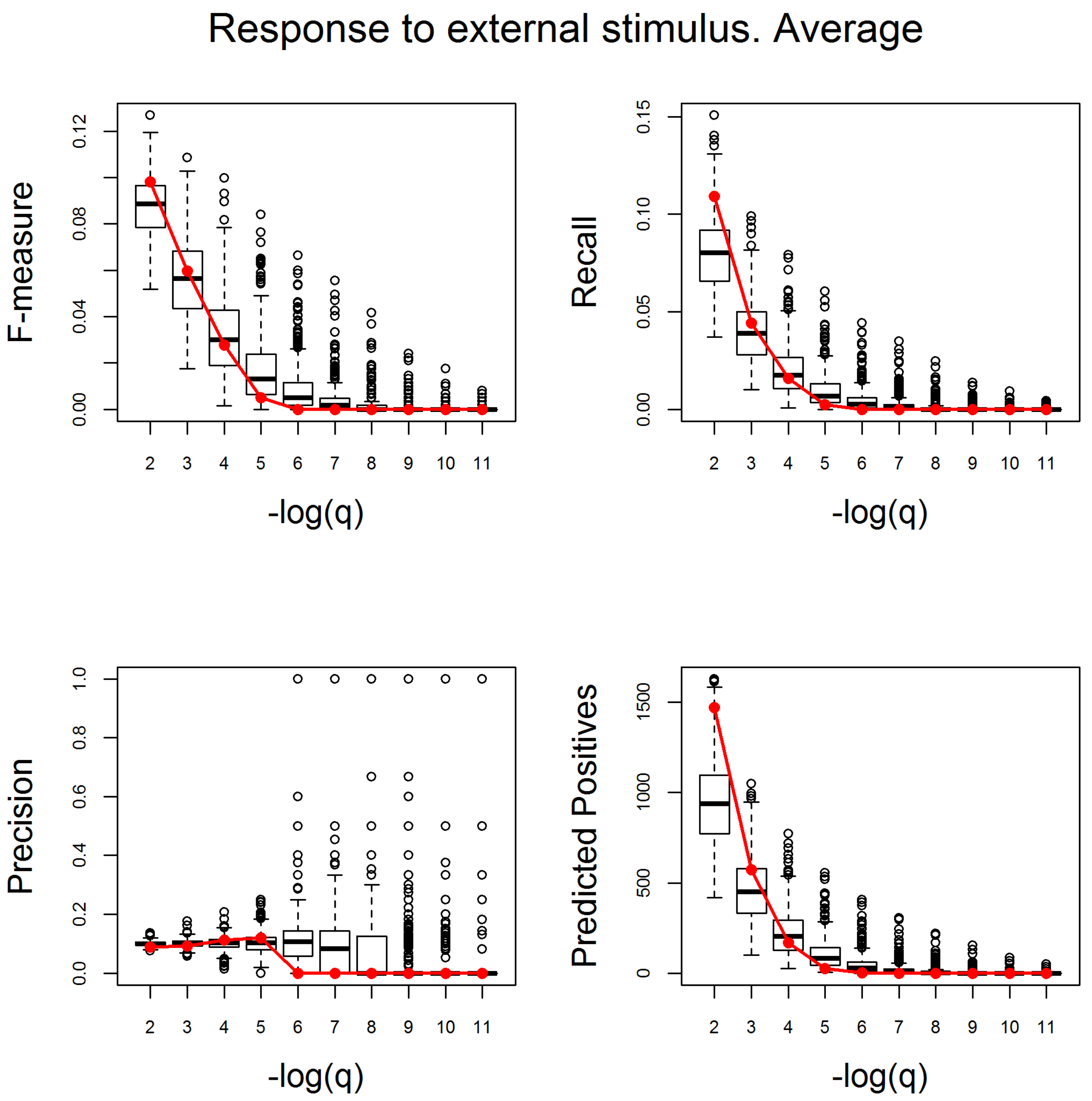

Appendix Figure S14. (continued)

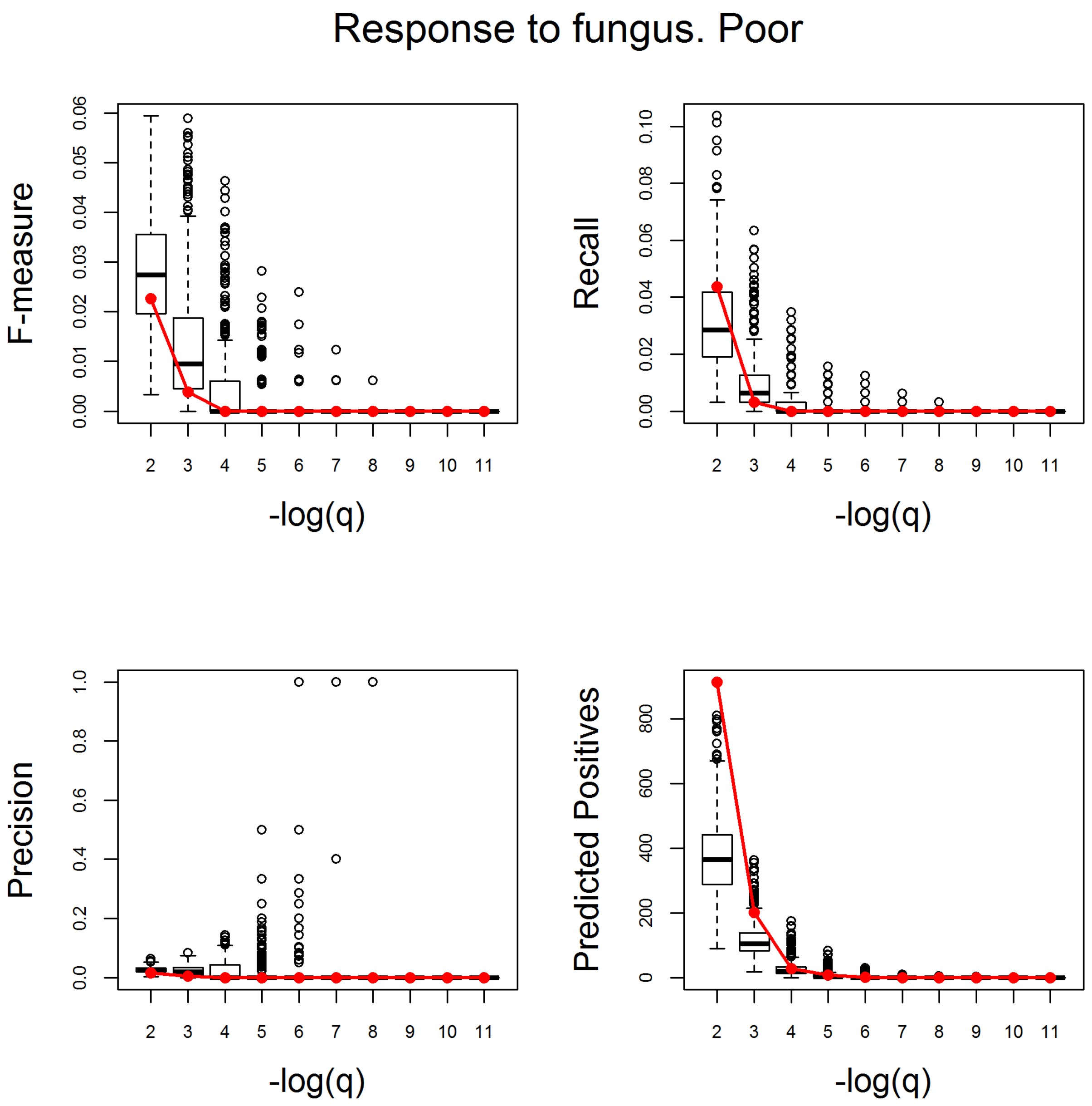

Appendix Figure S14. (continued)

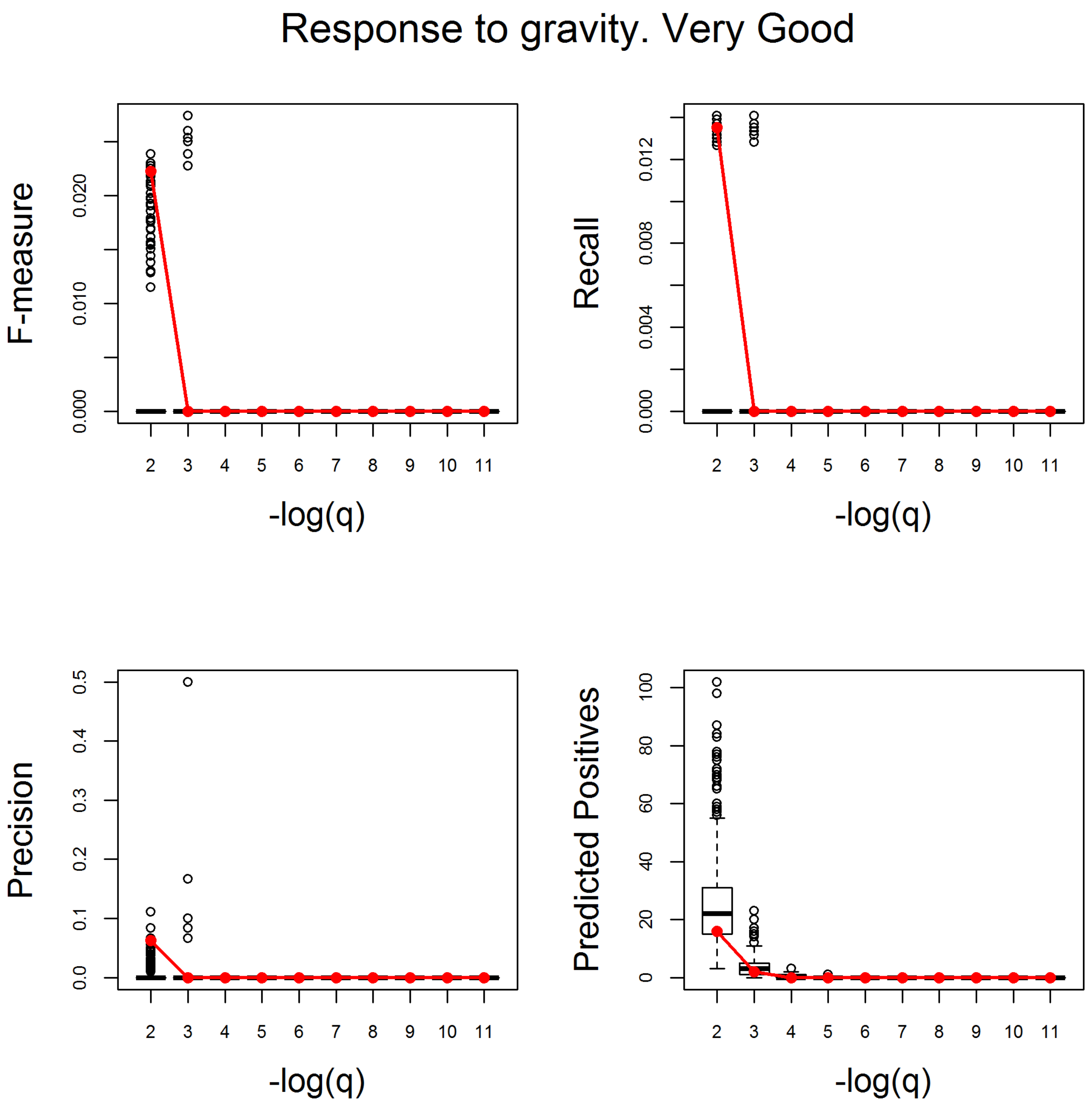

Appendix Figure S14. (continued)

Response to heat. Very Good

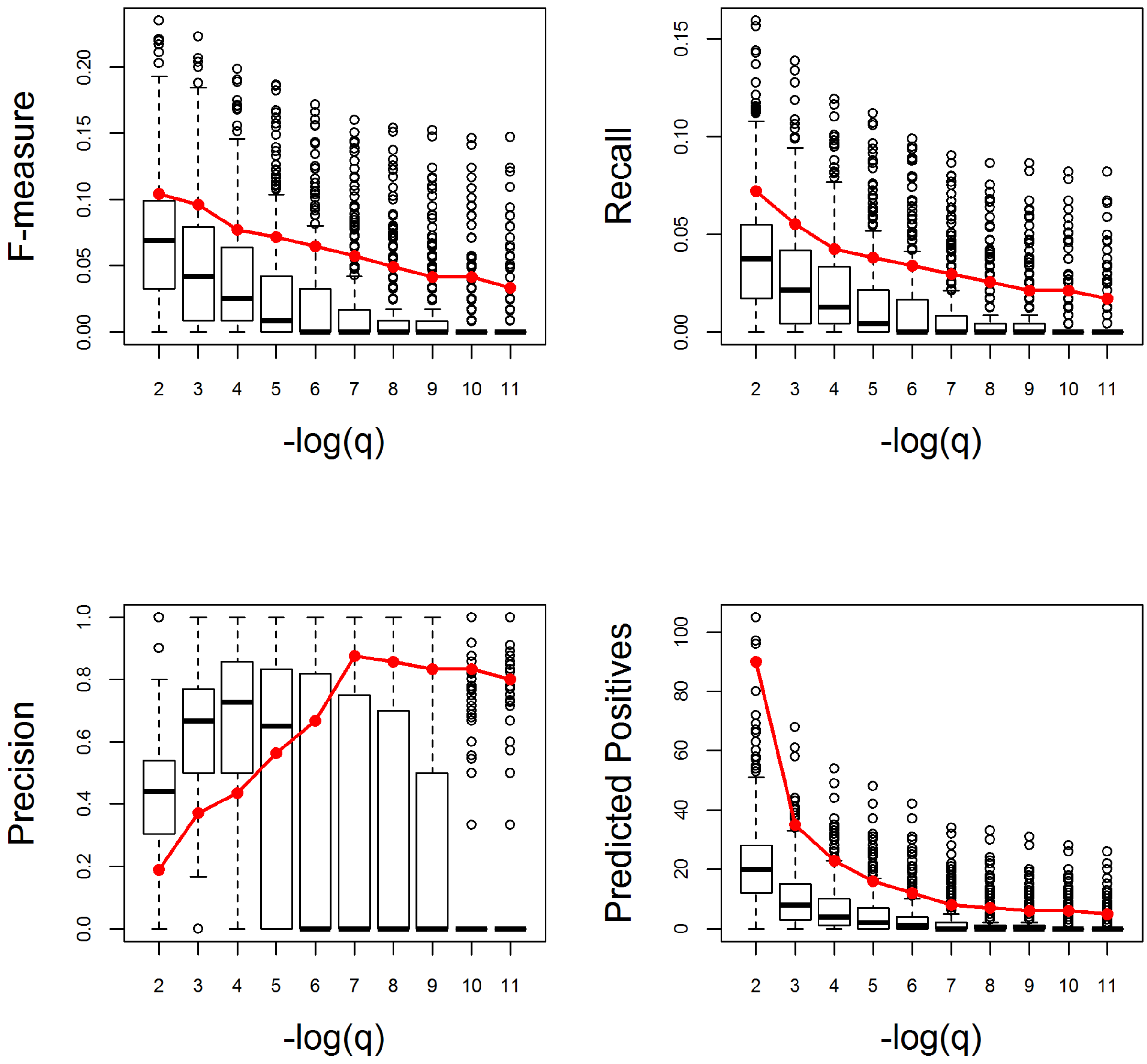

Appendix Figure S14. (continued)

Response to herbivore. Very Good

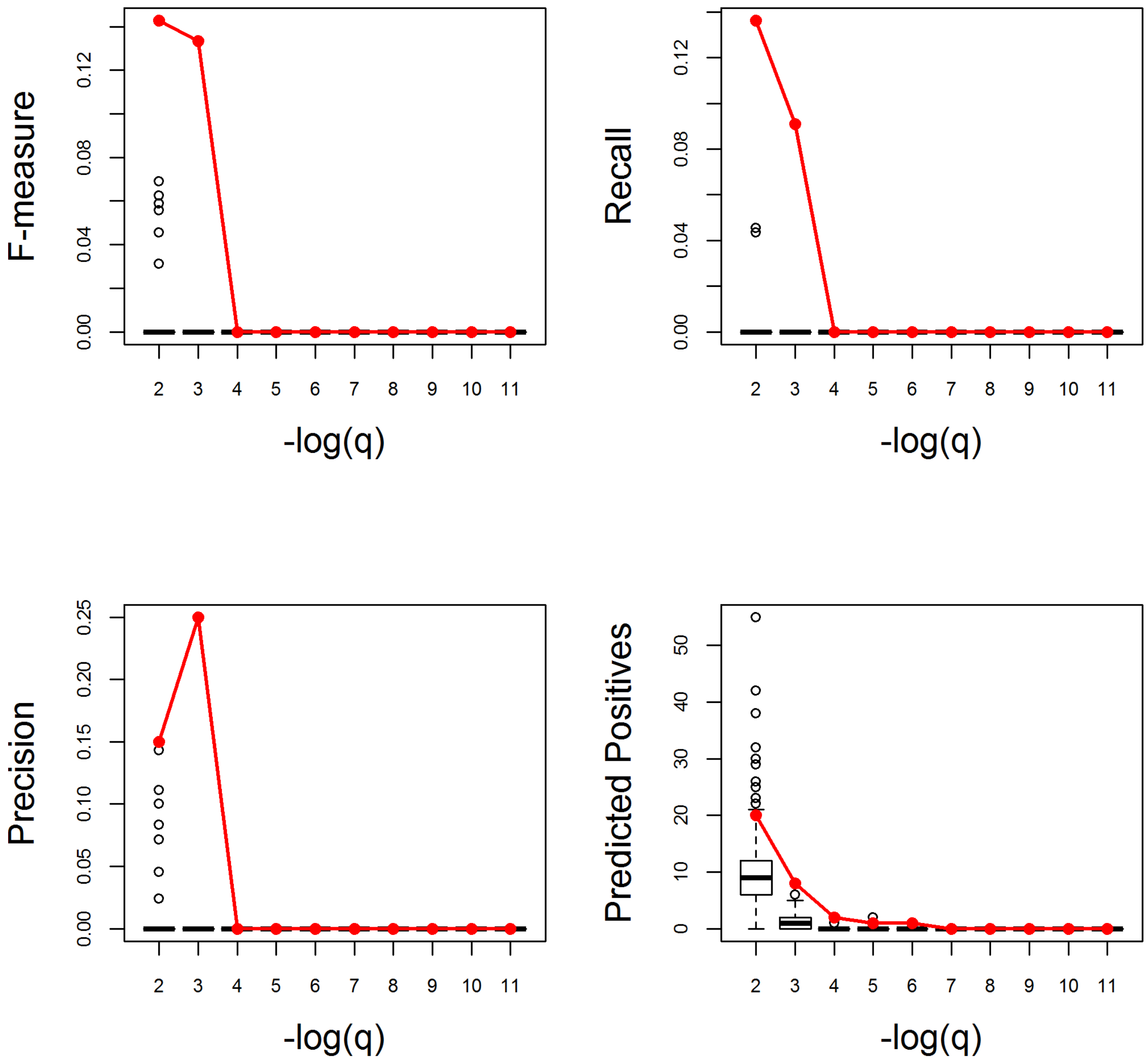

Appendix Figure S14. (continued)

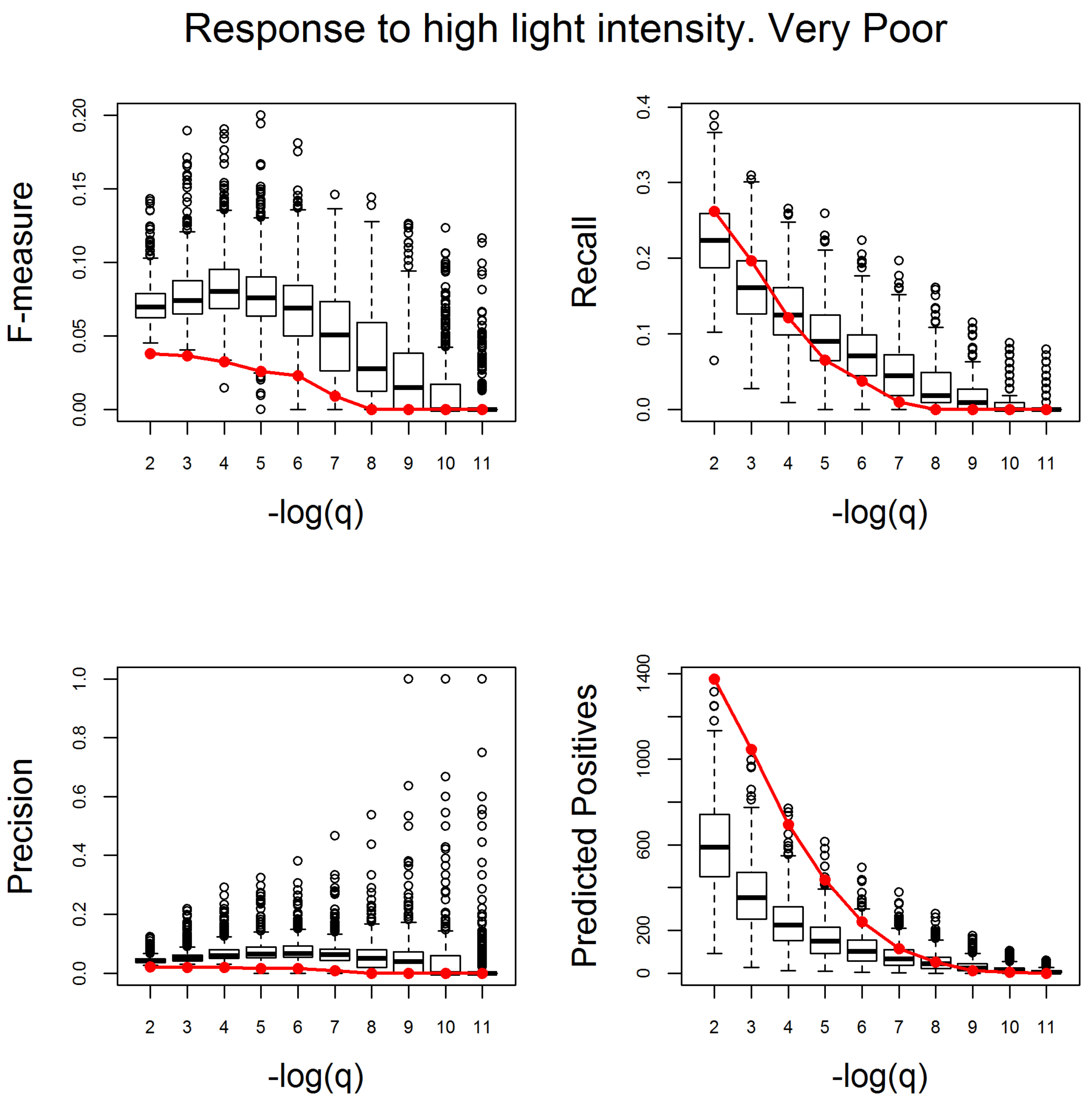

Appendix Figure S14. (continued)

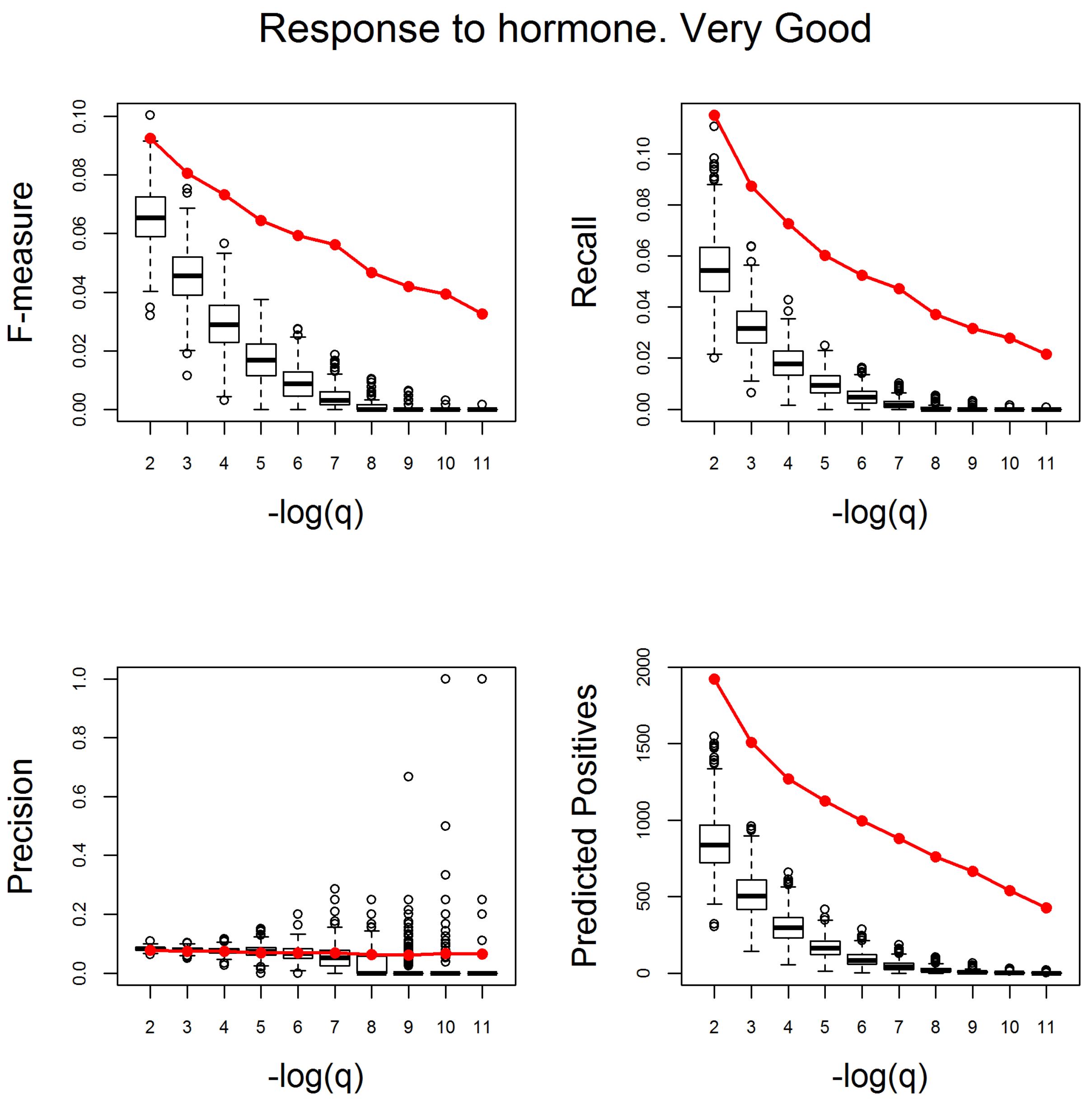

Appendix Figure S14. (continued)

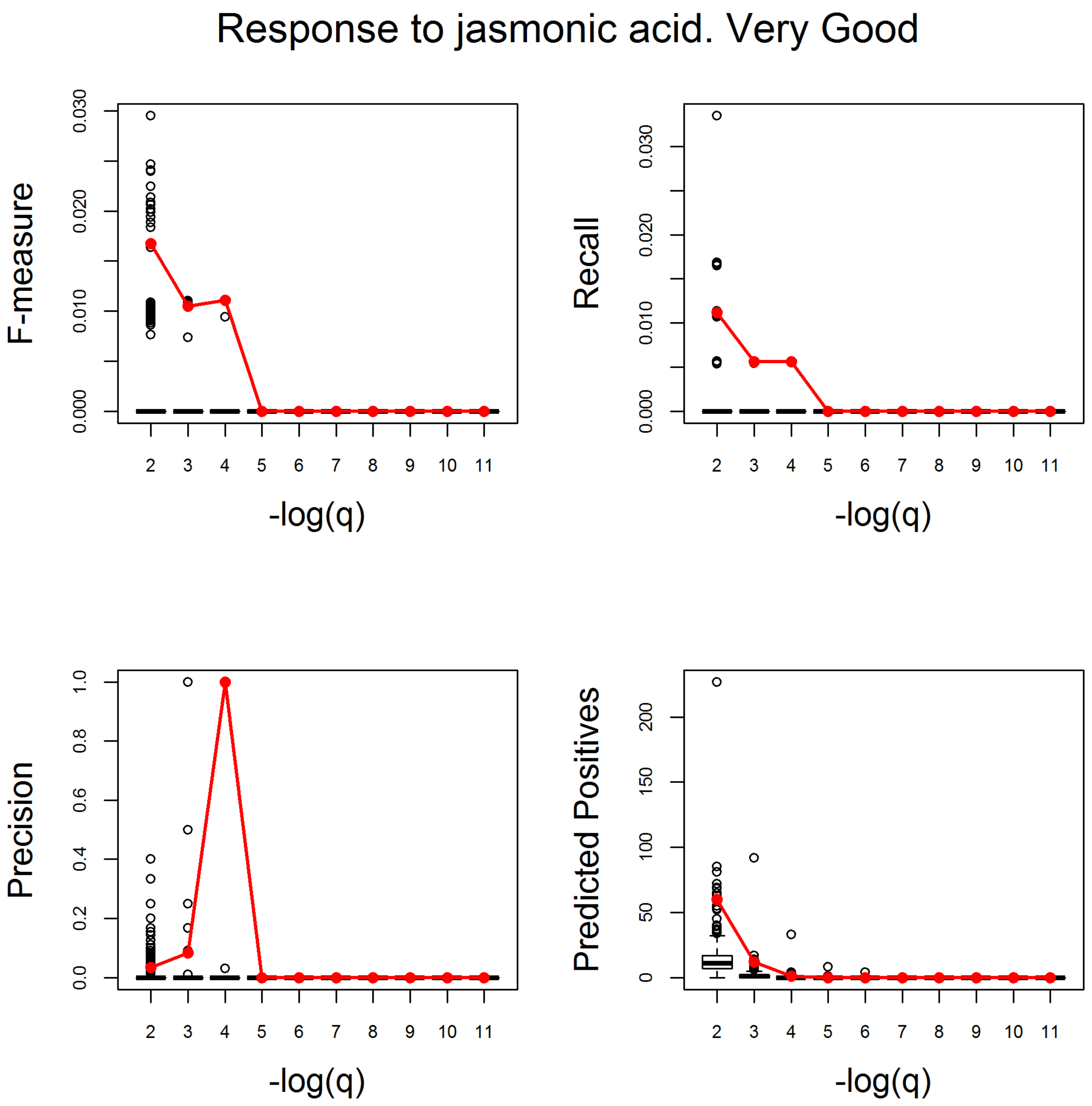

Appendix Figure S14. (continued)

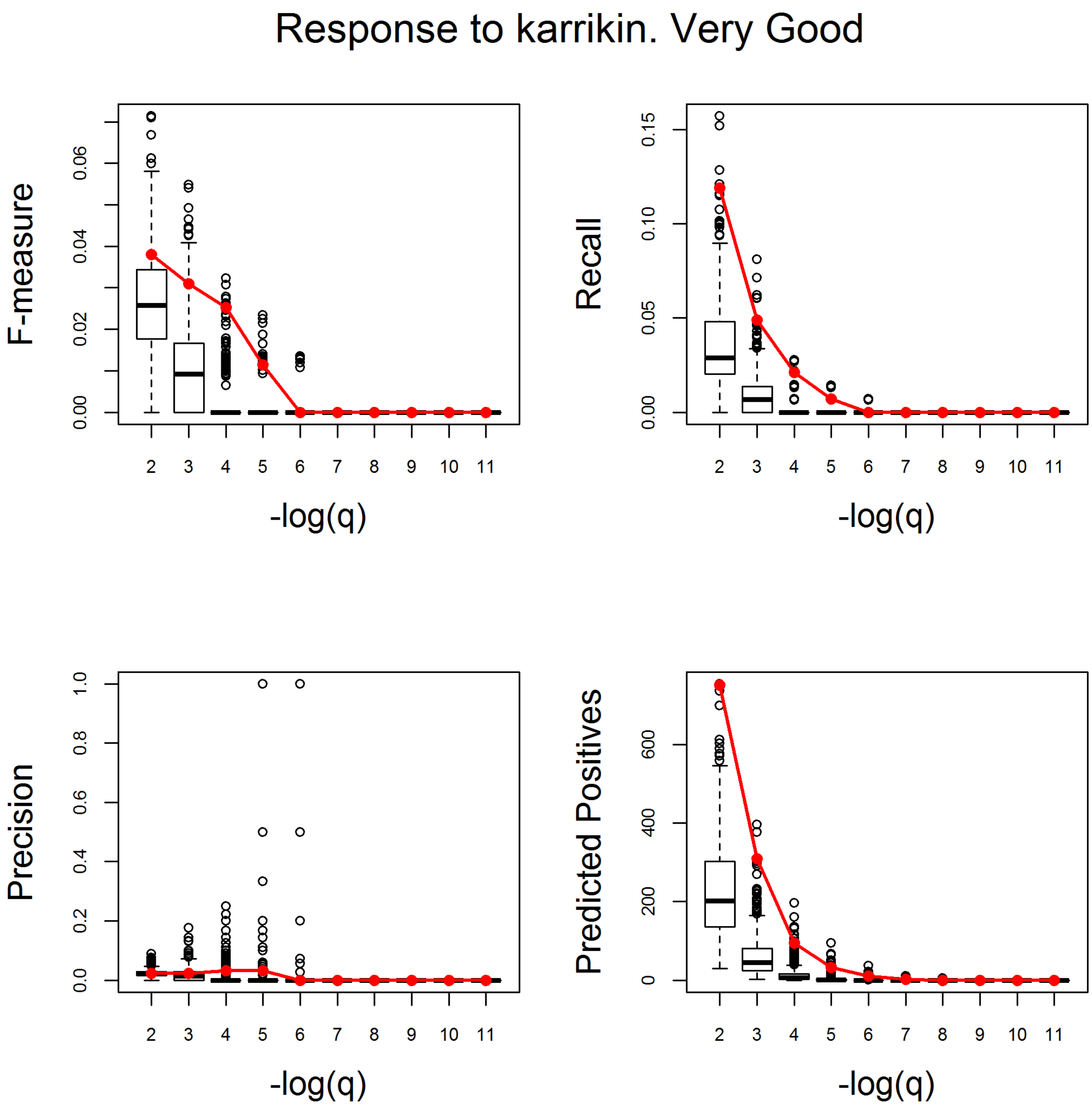

Appendix Figure S14. (continued)

Response to light intensity. Very Poor

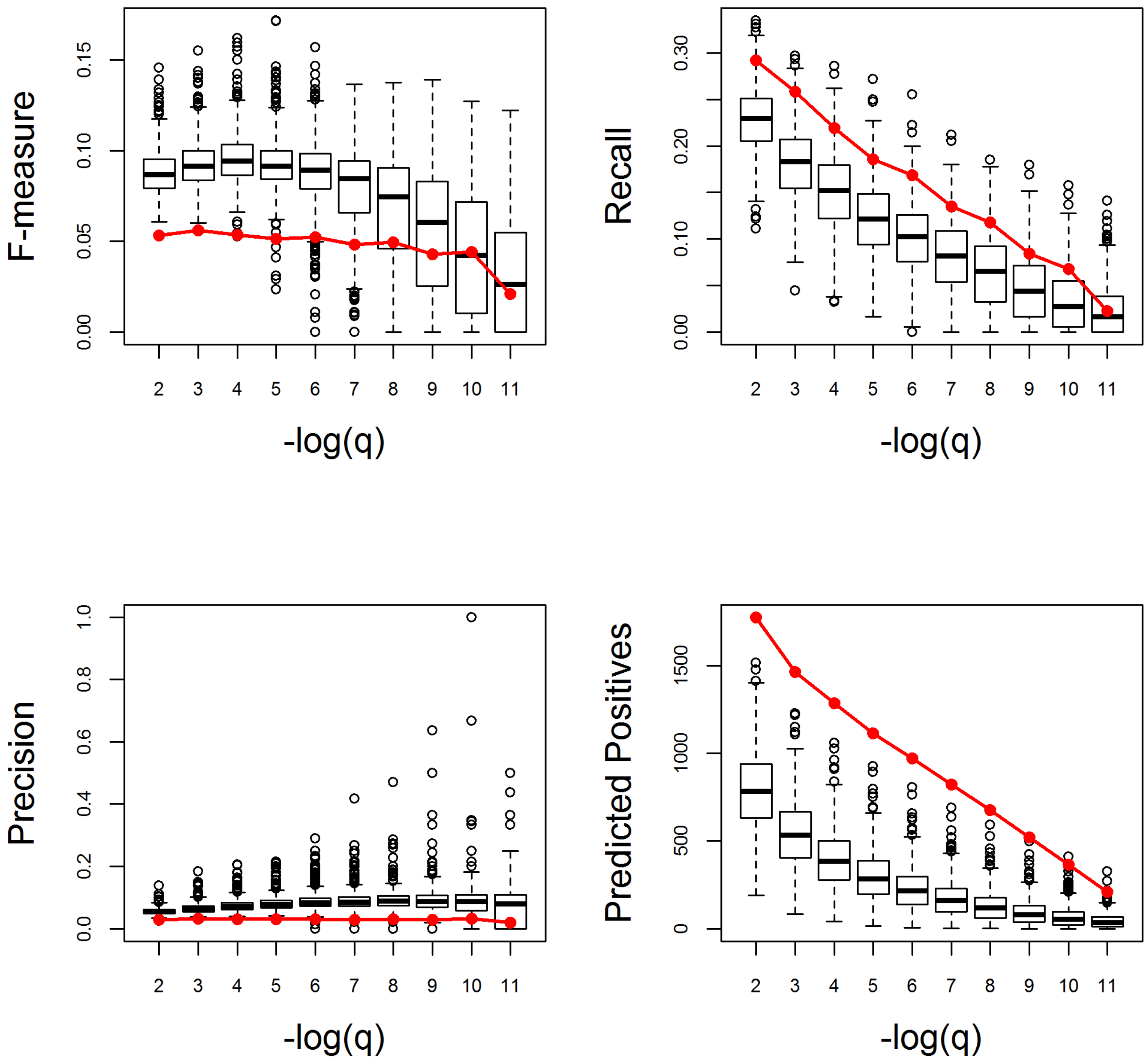

Appendix Figure S14. (continued)

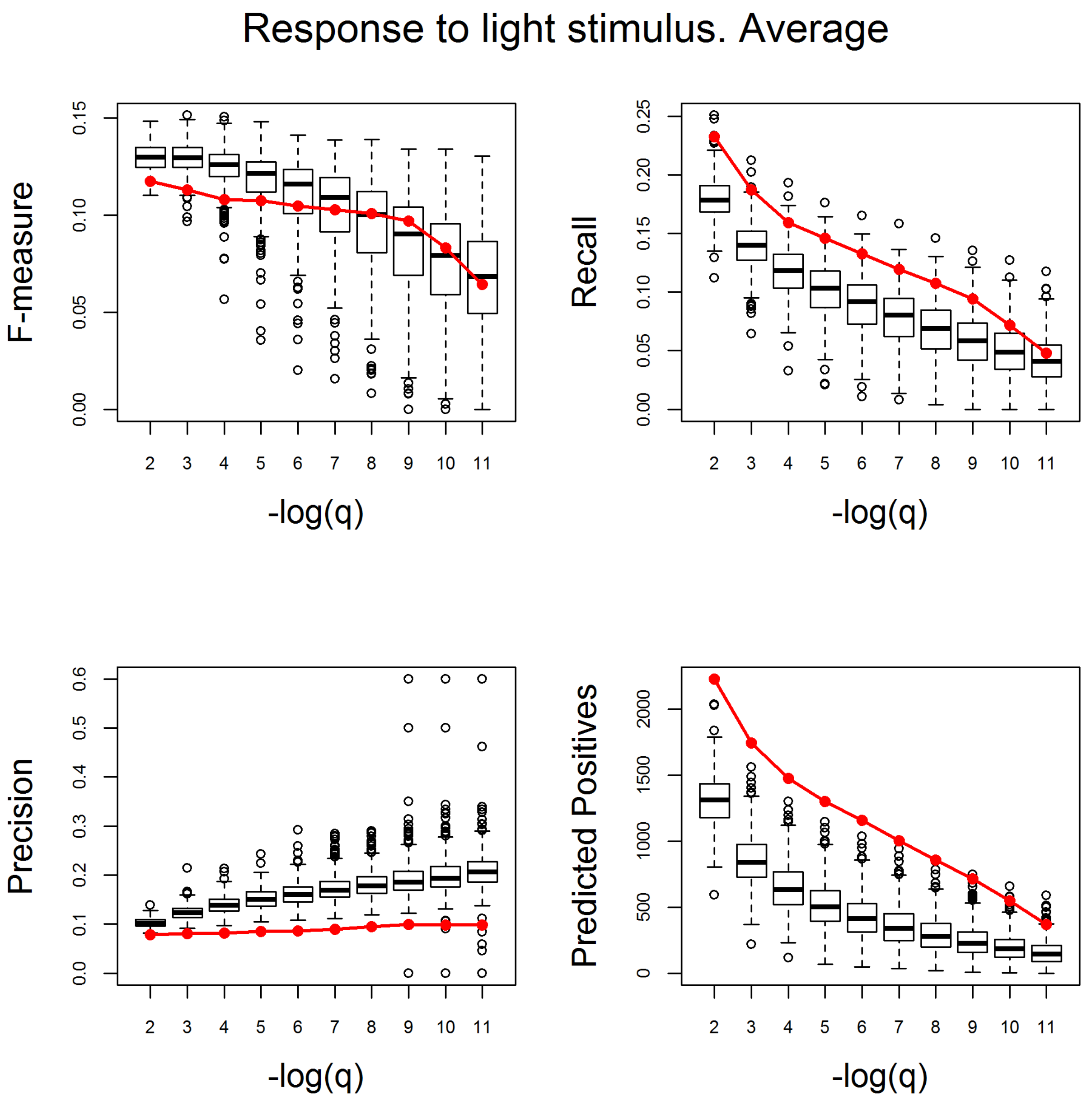

Appendix Figure S14. (continued)

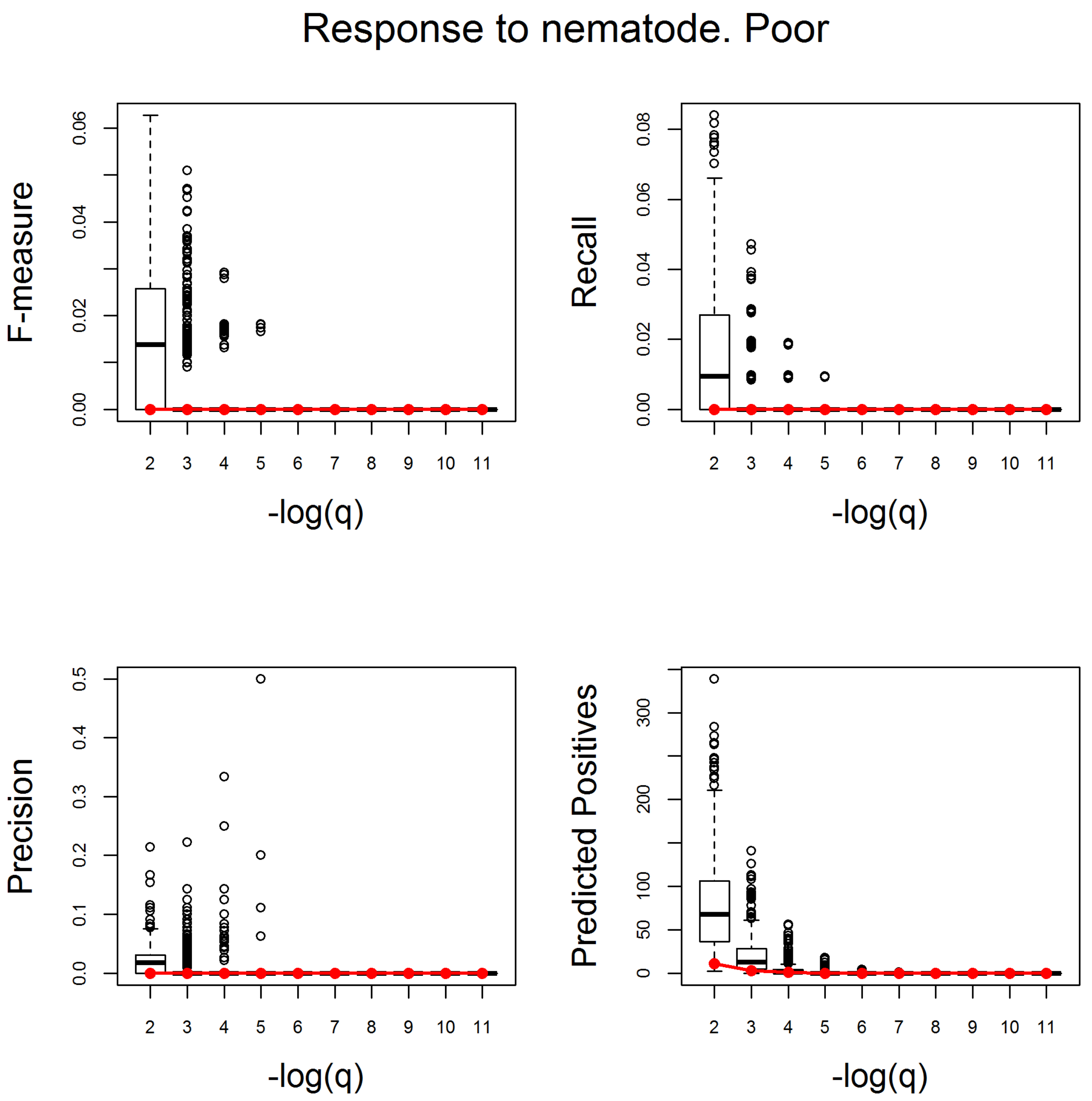

Appendix Figure S14. (continued)

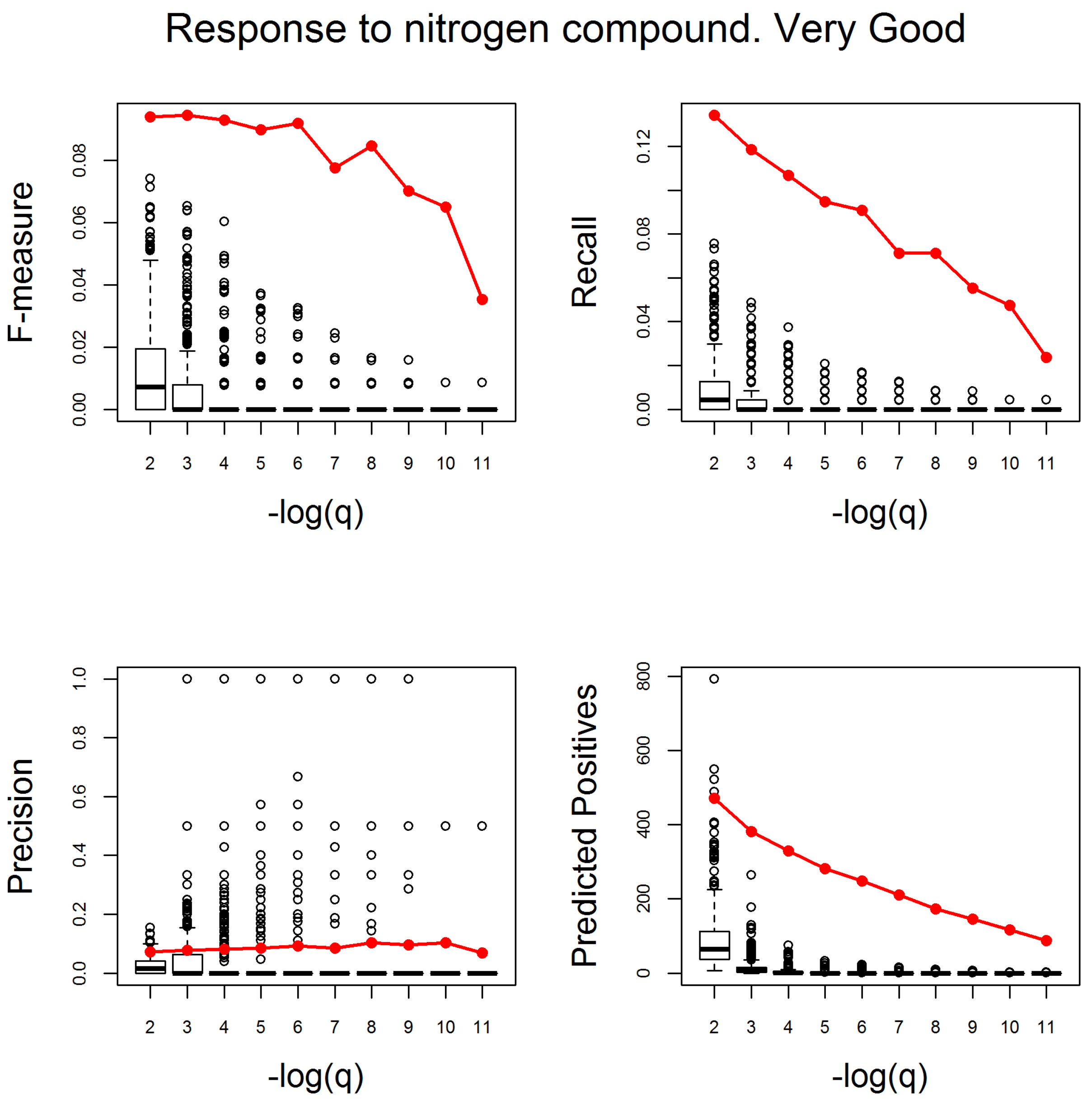

Appendix Figure S14. (continued)

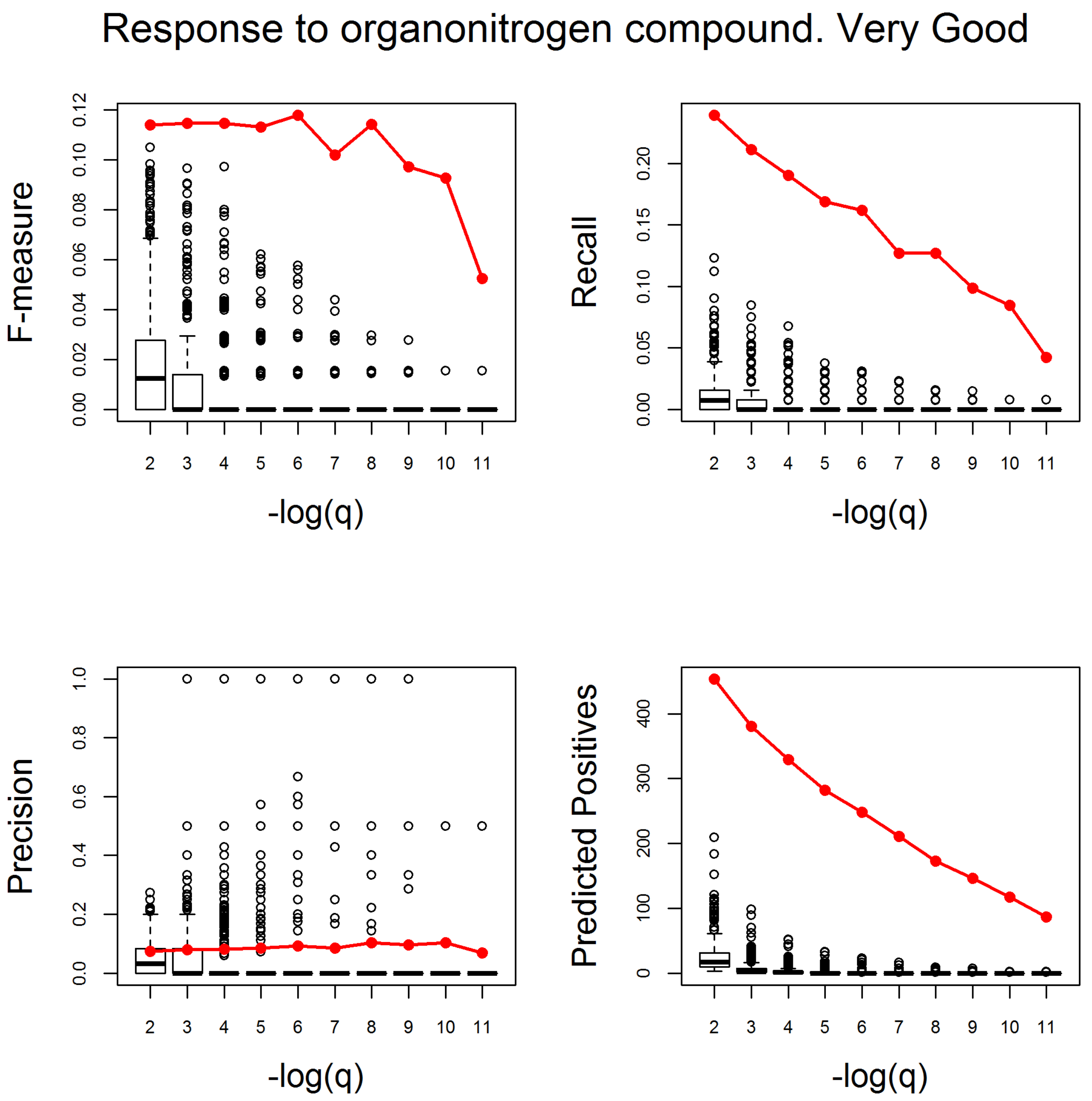

Appendix Figure S14. (continued)

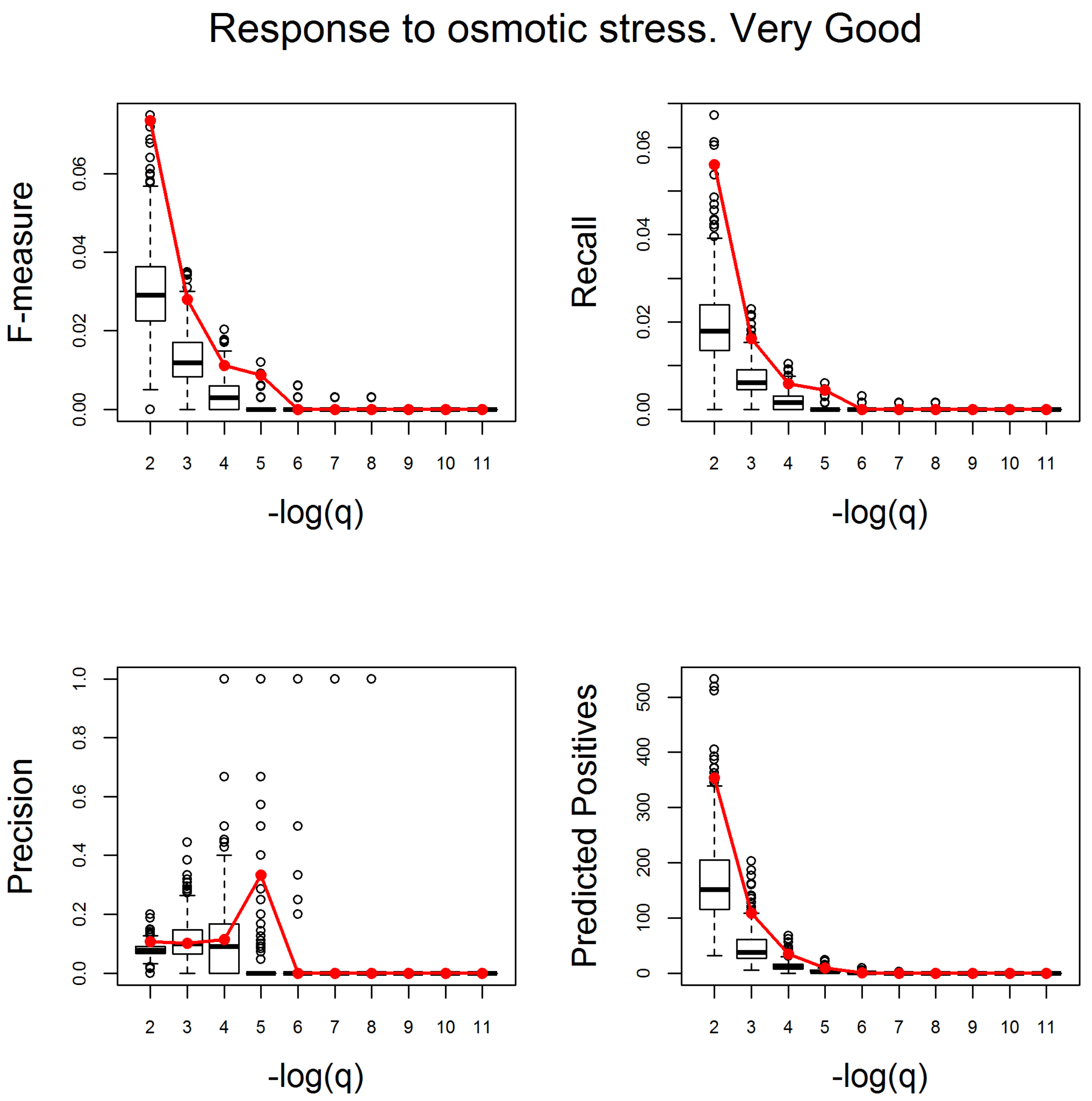

Appendix Figure S14. (continued)

Response to other organism. Average

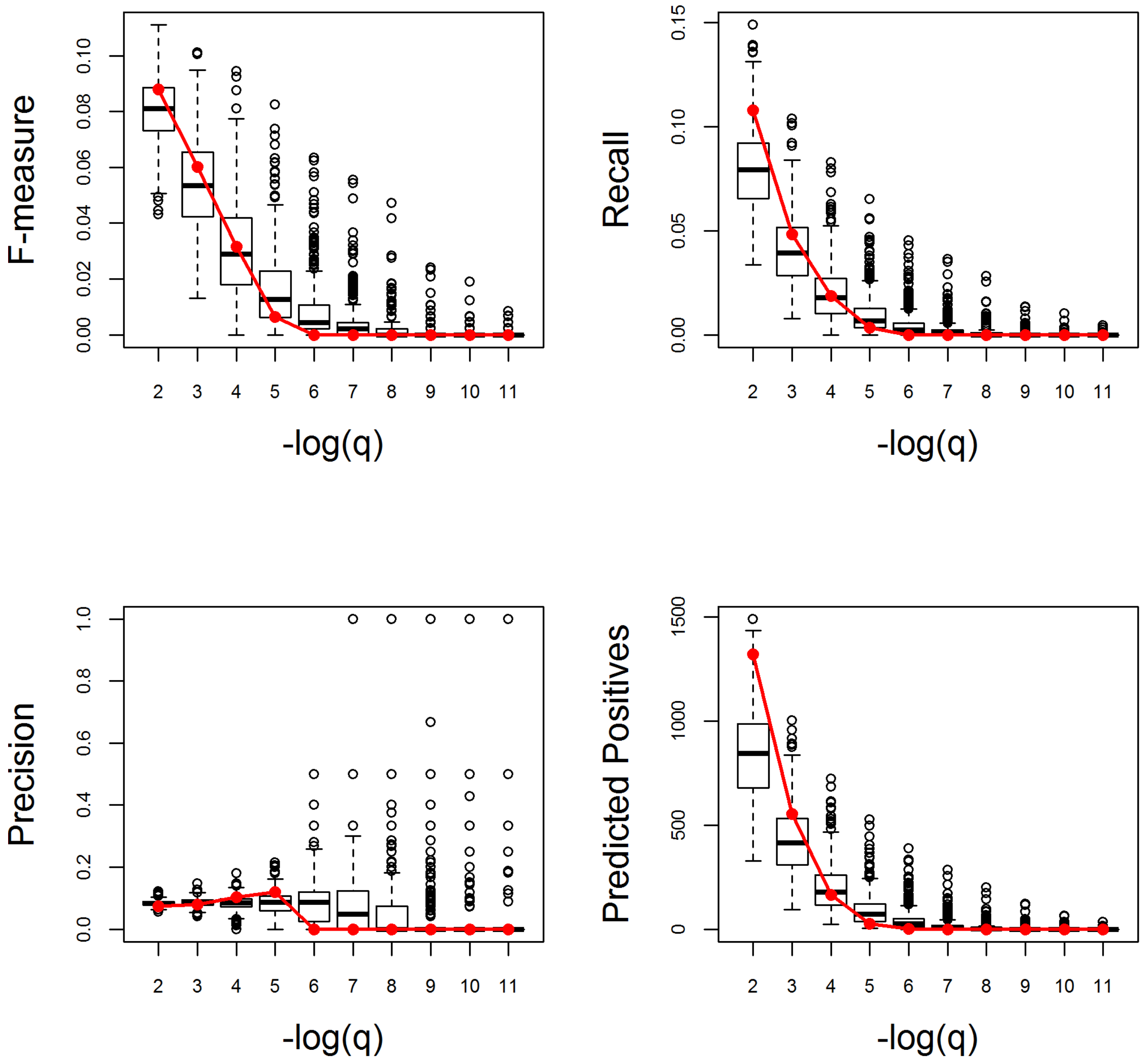

Appendix Figure S14. (continued)

Response to oxidative stress. Good

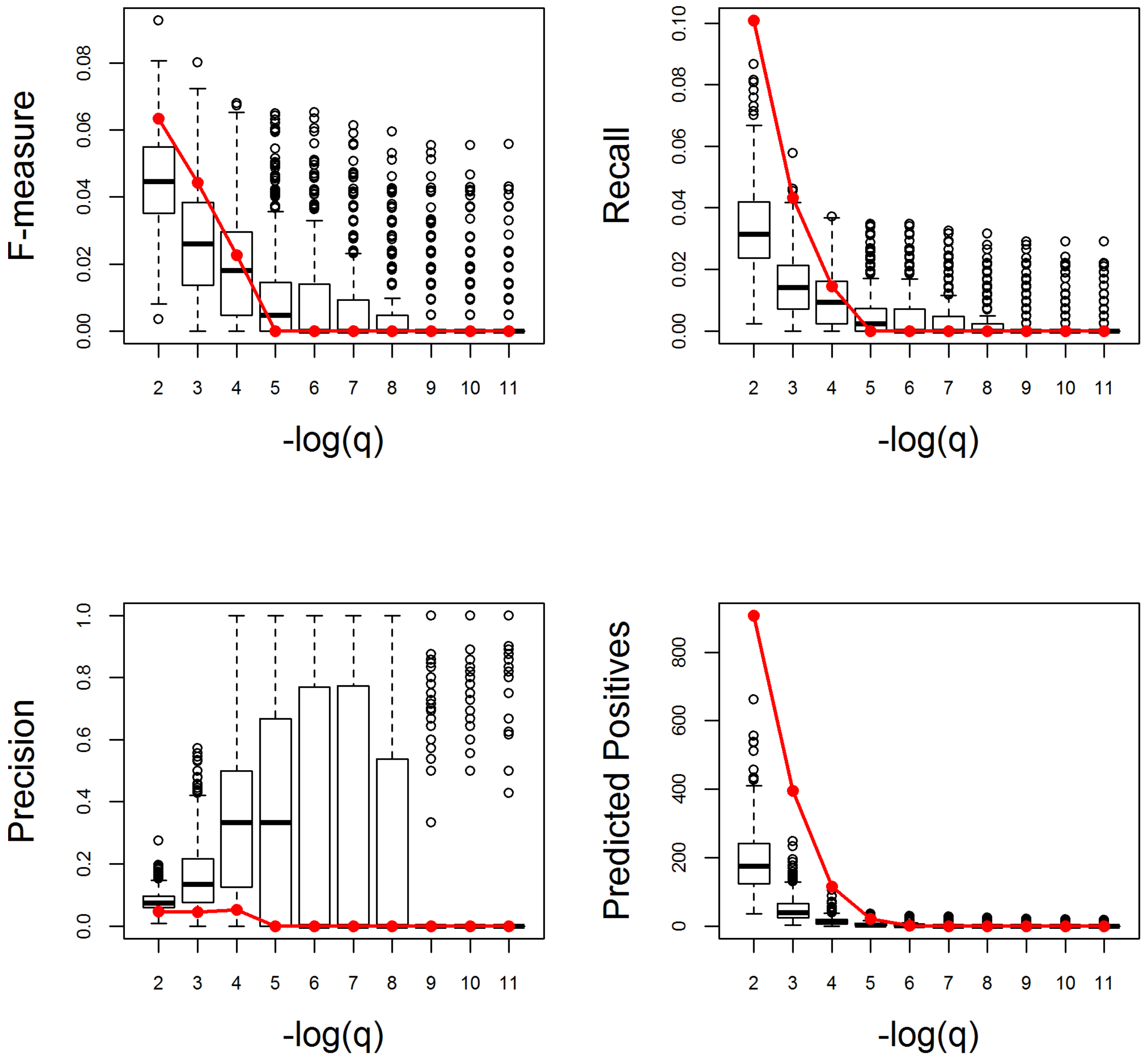

Appendix Figure S14. (continued)

Response to oxygen-containing compound. Very Good

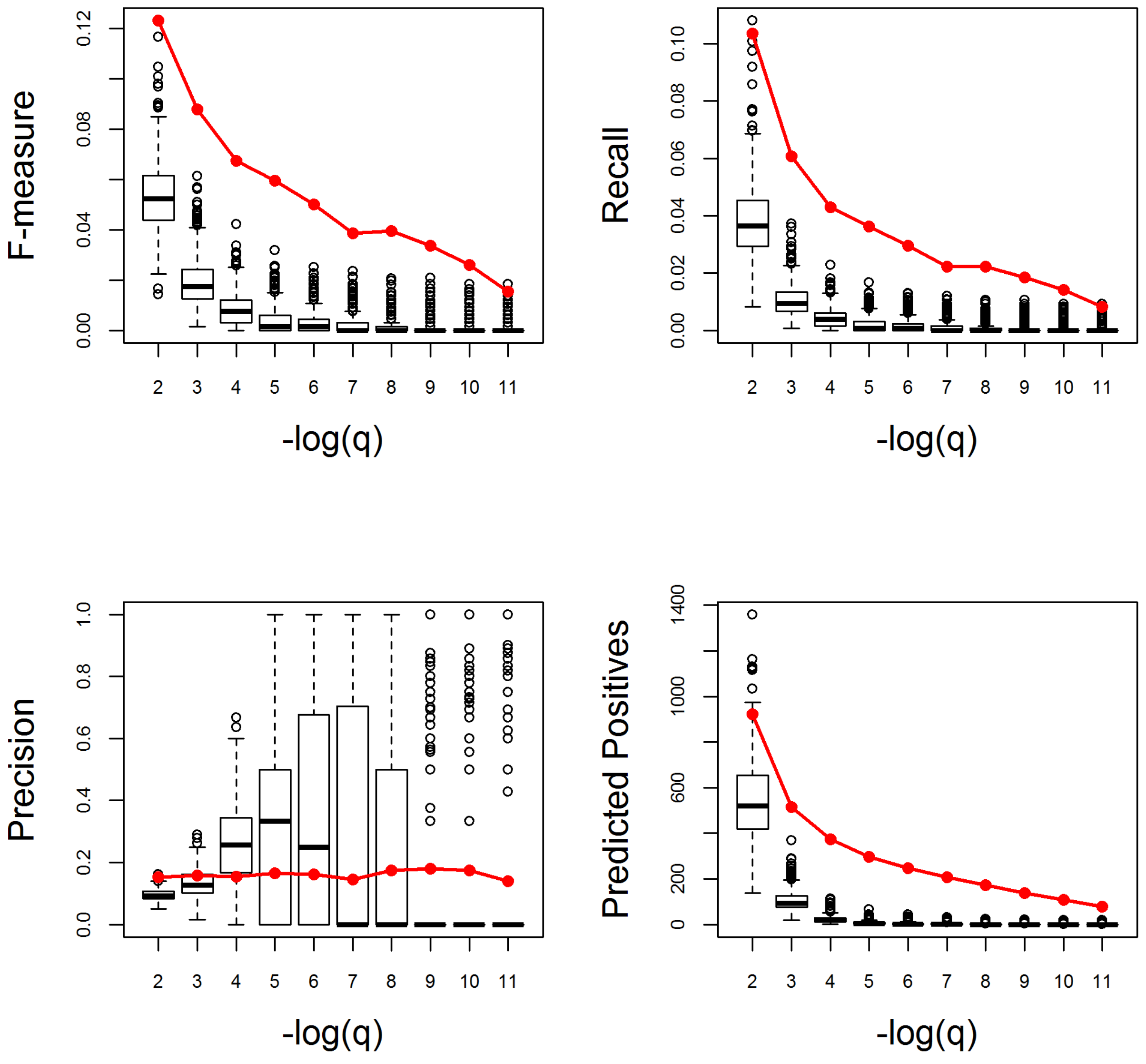

Appendix Figure S14. (continued)

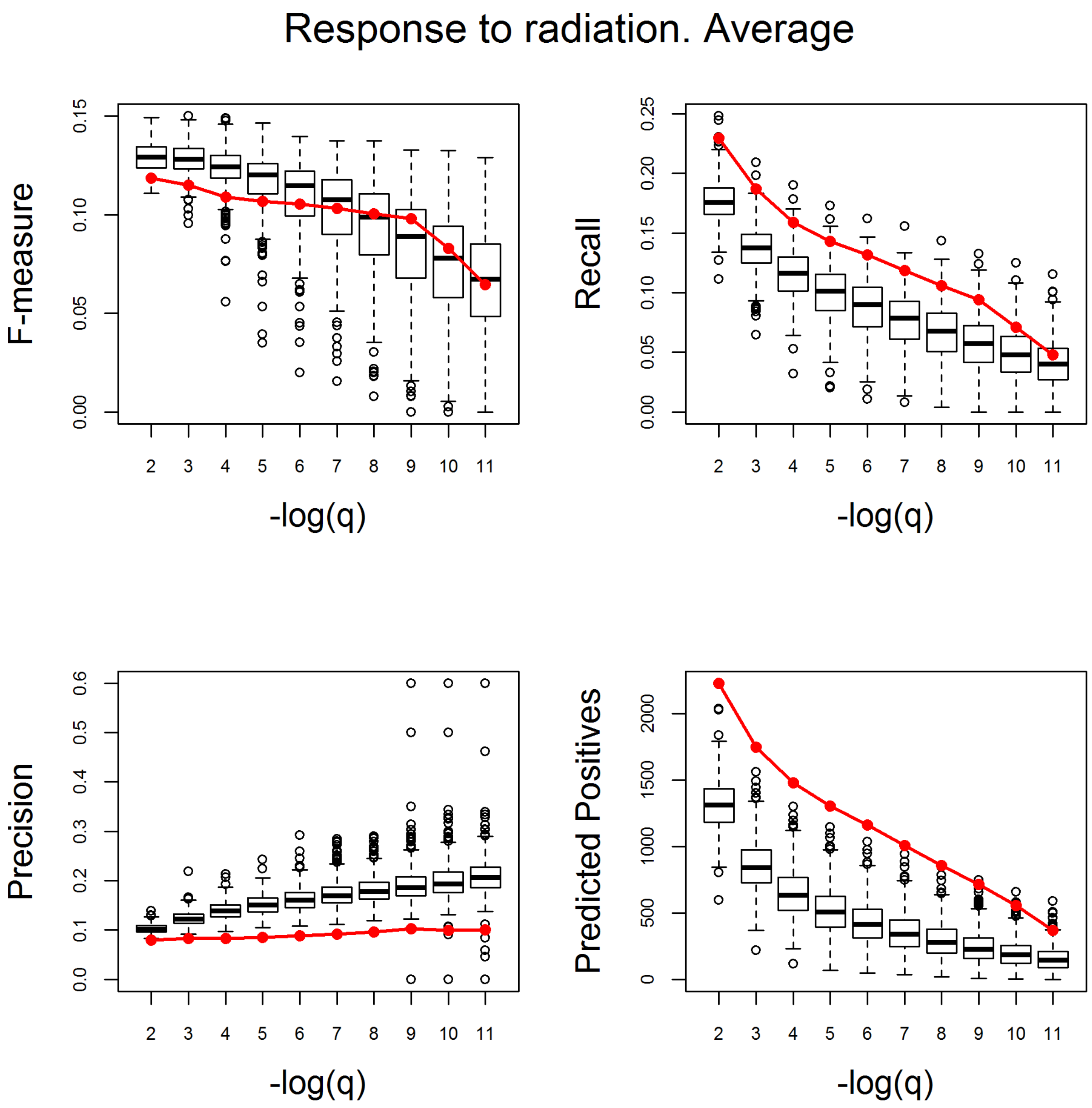

Appendix Figure S14. (continued)

Response to reactive oxygen species. Very Poor

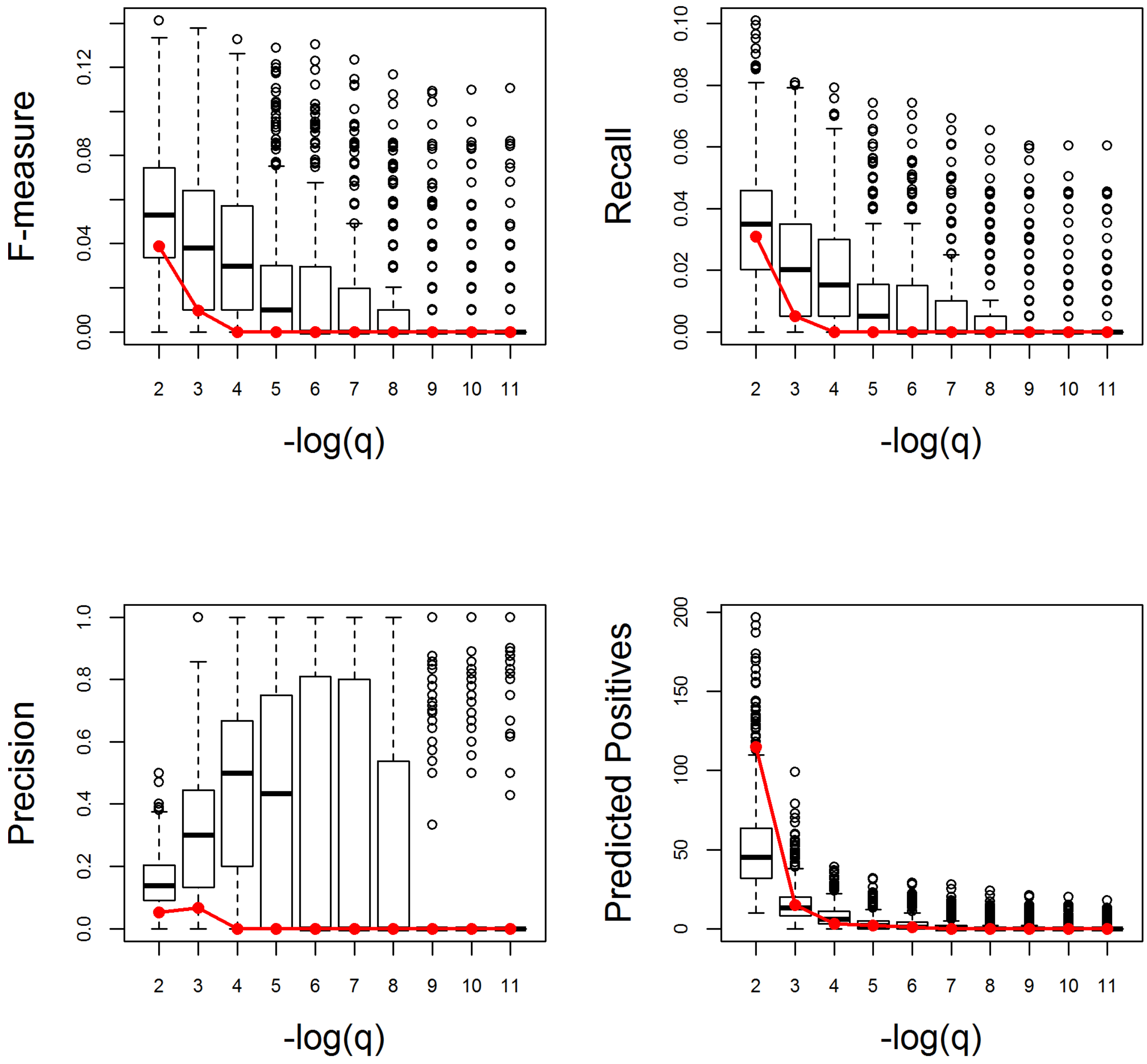

Appendix Figure S14. (continued)

Response to red or far red light. Very Poor

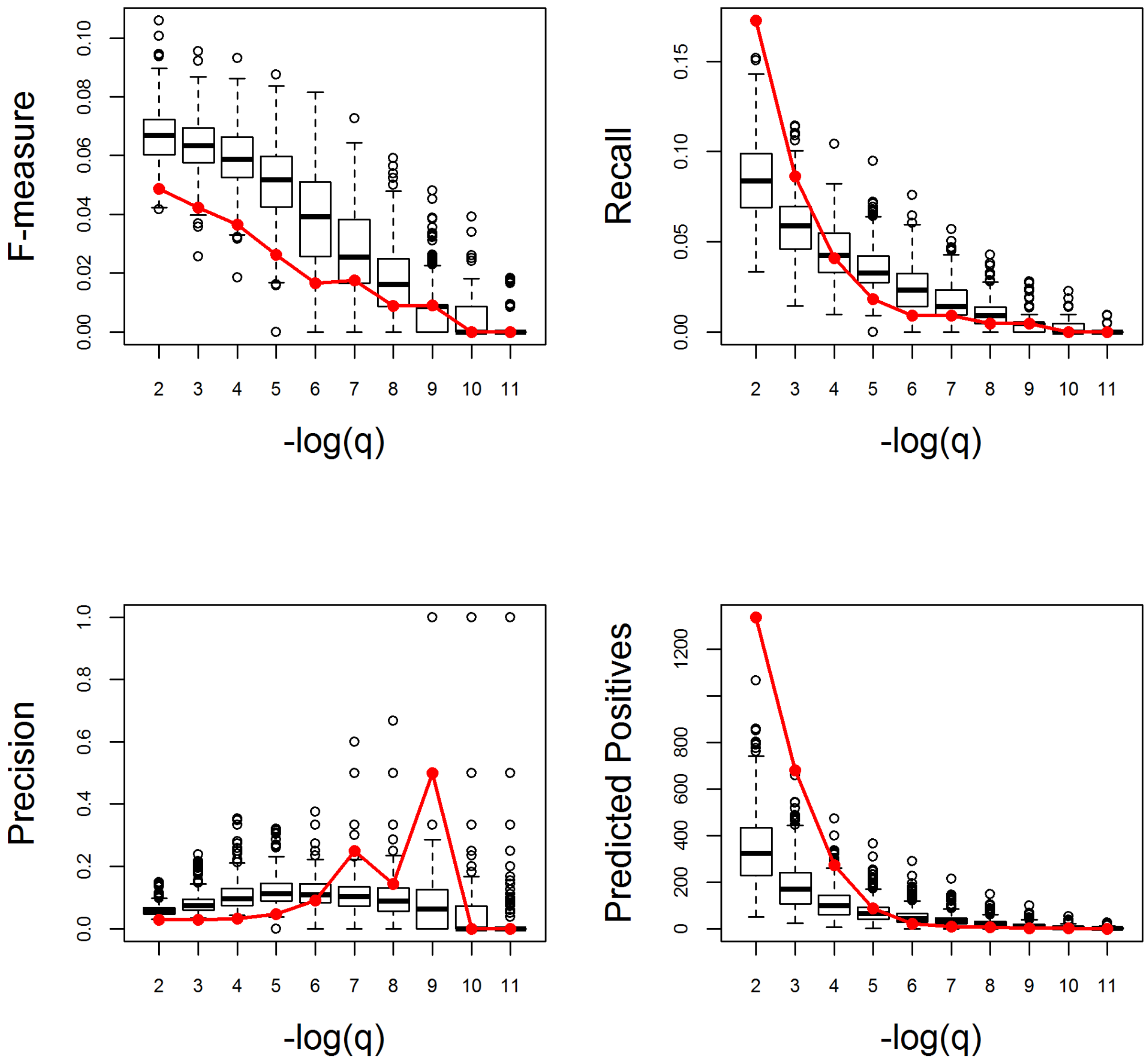

Appendix Figure S14. (continued)

Response to salt stress. Very Good

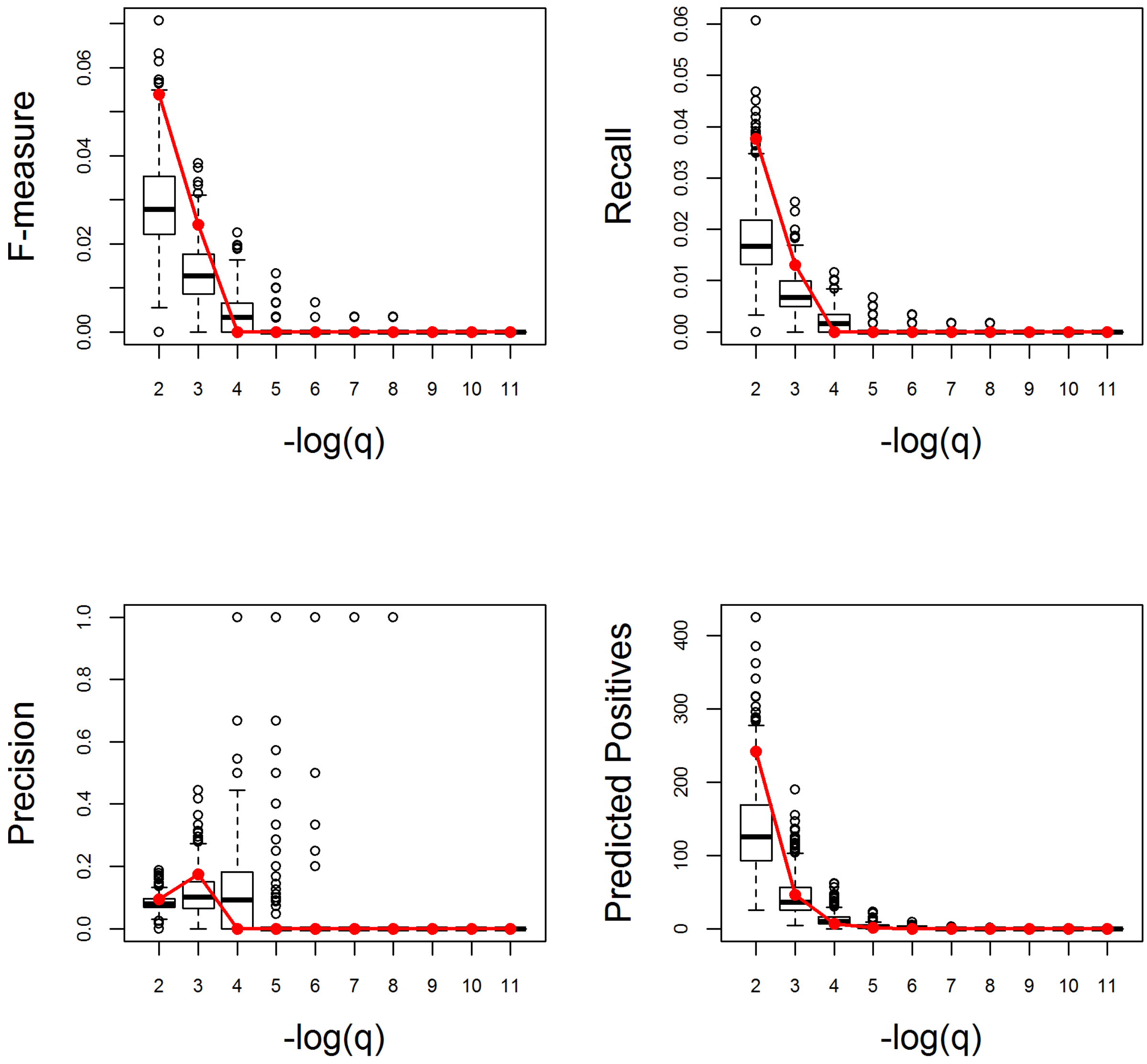

Appendix Figure S14. (continued)

Response to starvation. Very Good

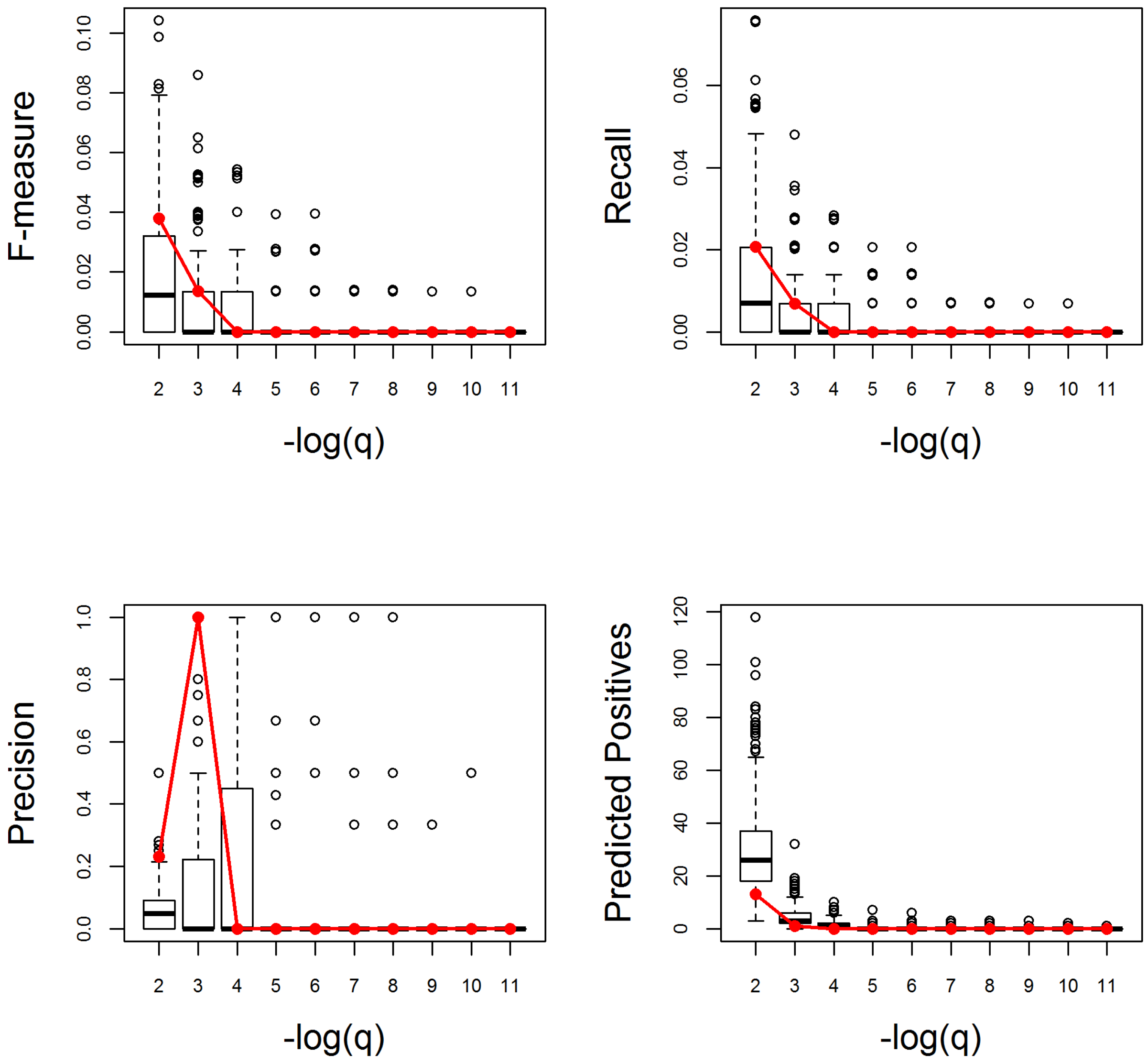

Appendix Figure S14. (continued)

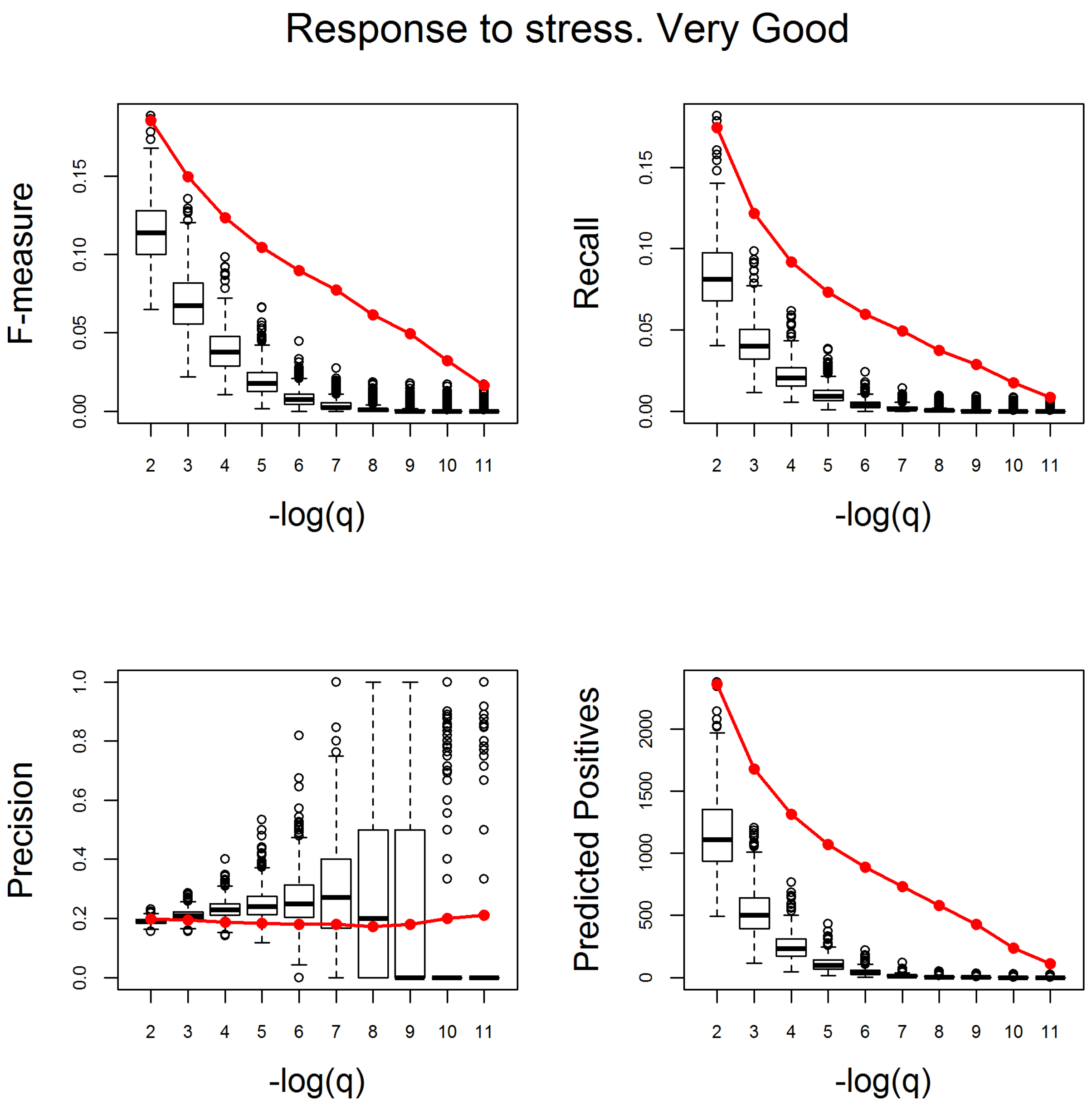

Appendix Figure S14. (continued)

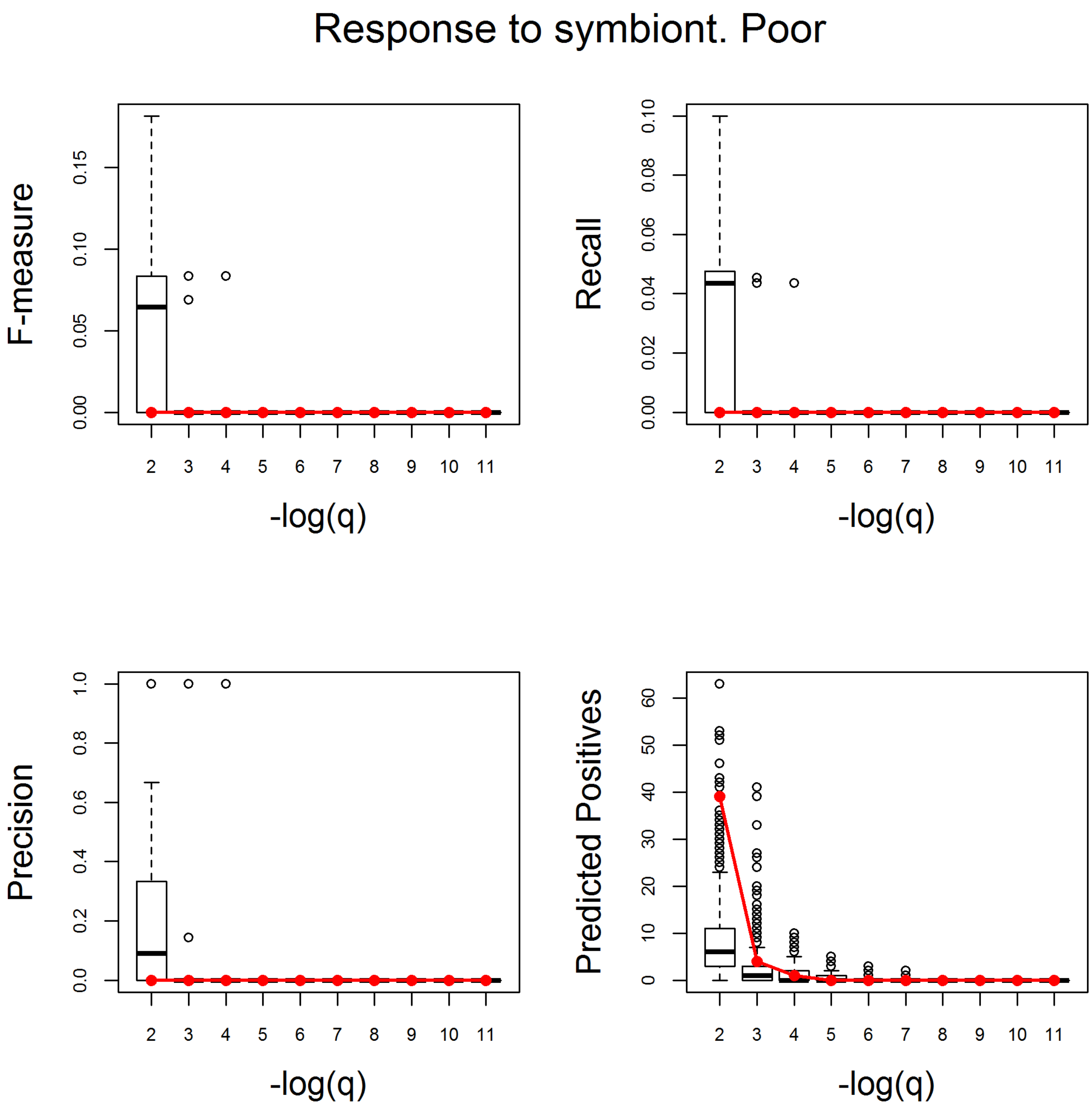

Appendix Figure S14. (continued)

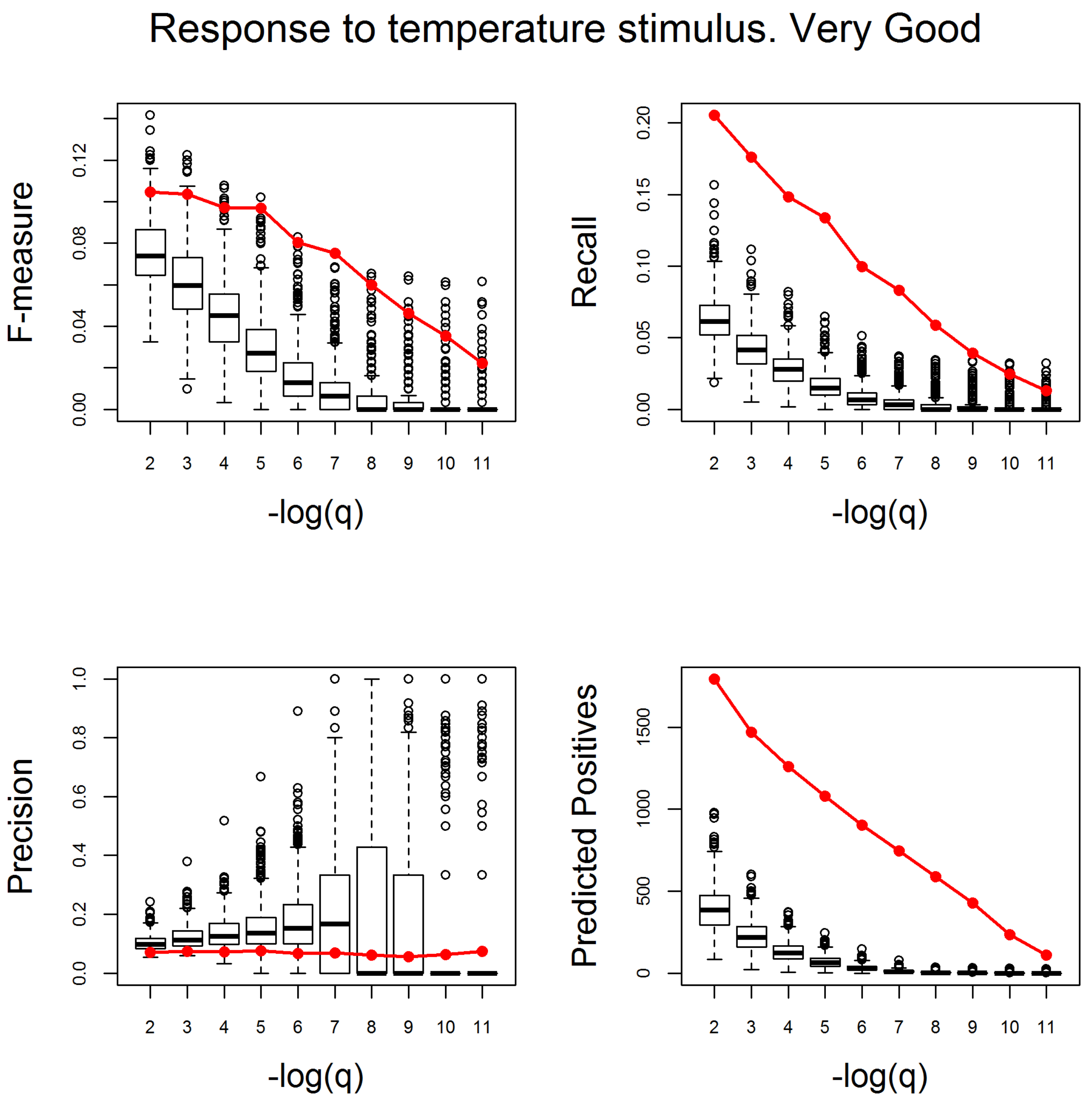

Appendix Figure S14. (continued)

Response to UV. Very Good

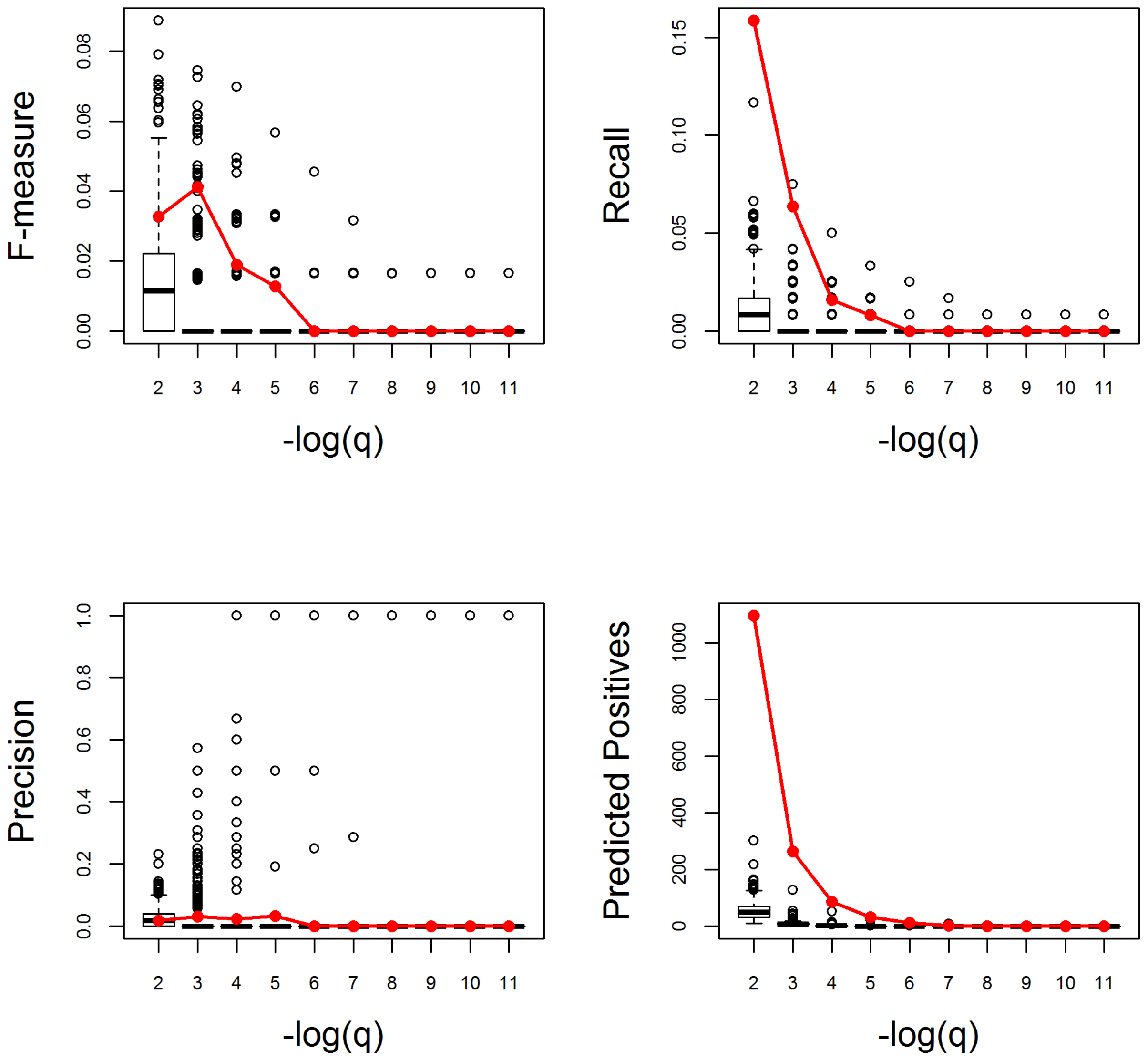

Appendix Figure S14. (continued)

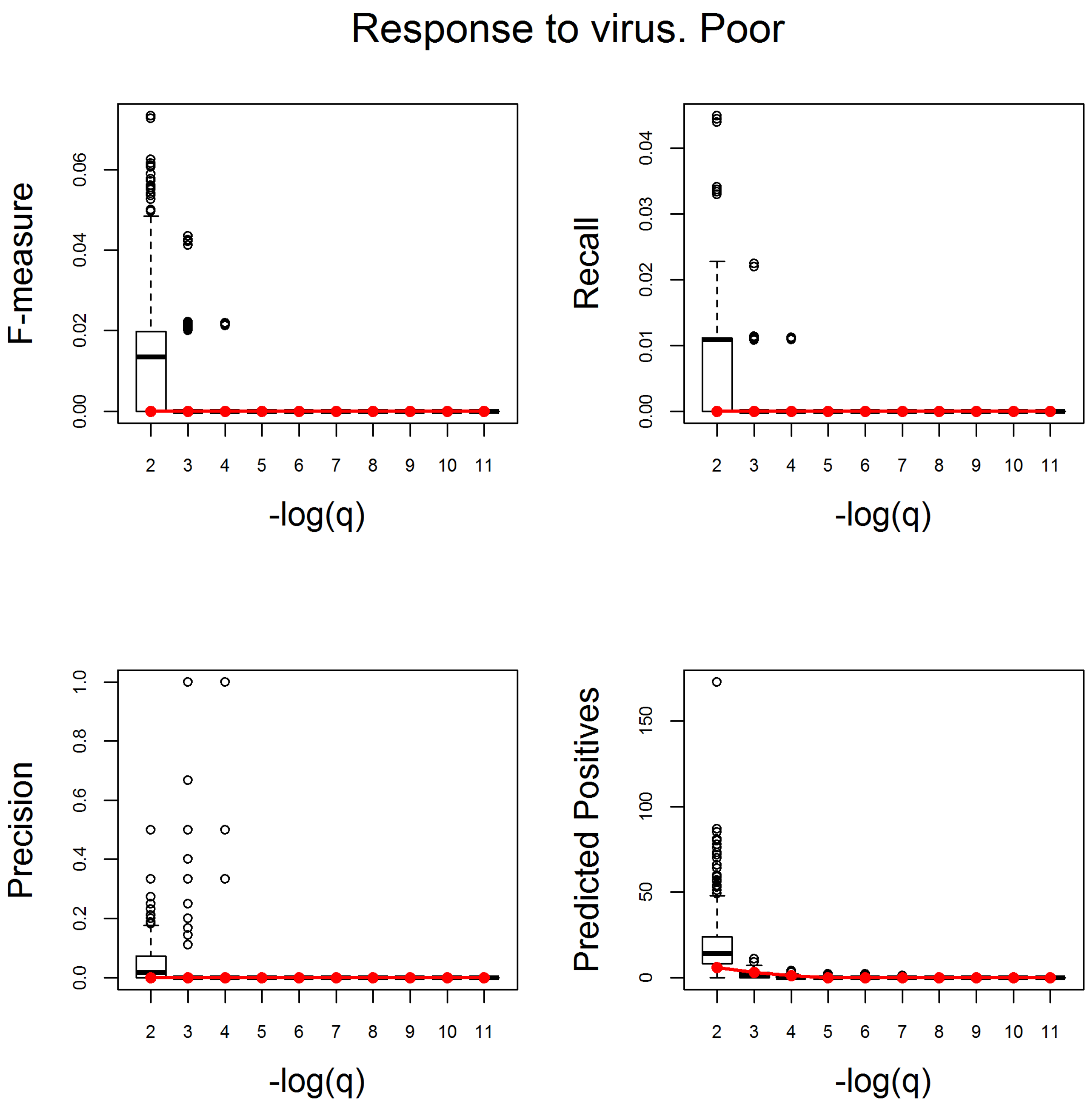

Appendix Figure S14. (continued)

Response to water deprivation. Very Good

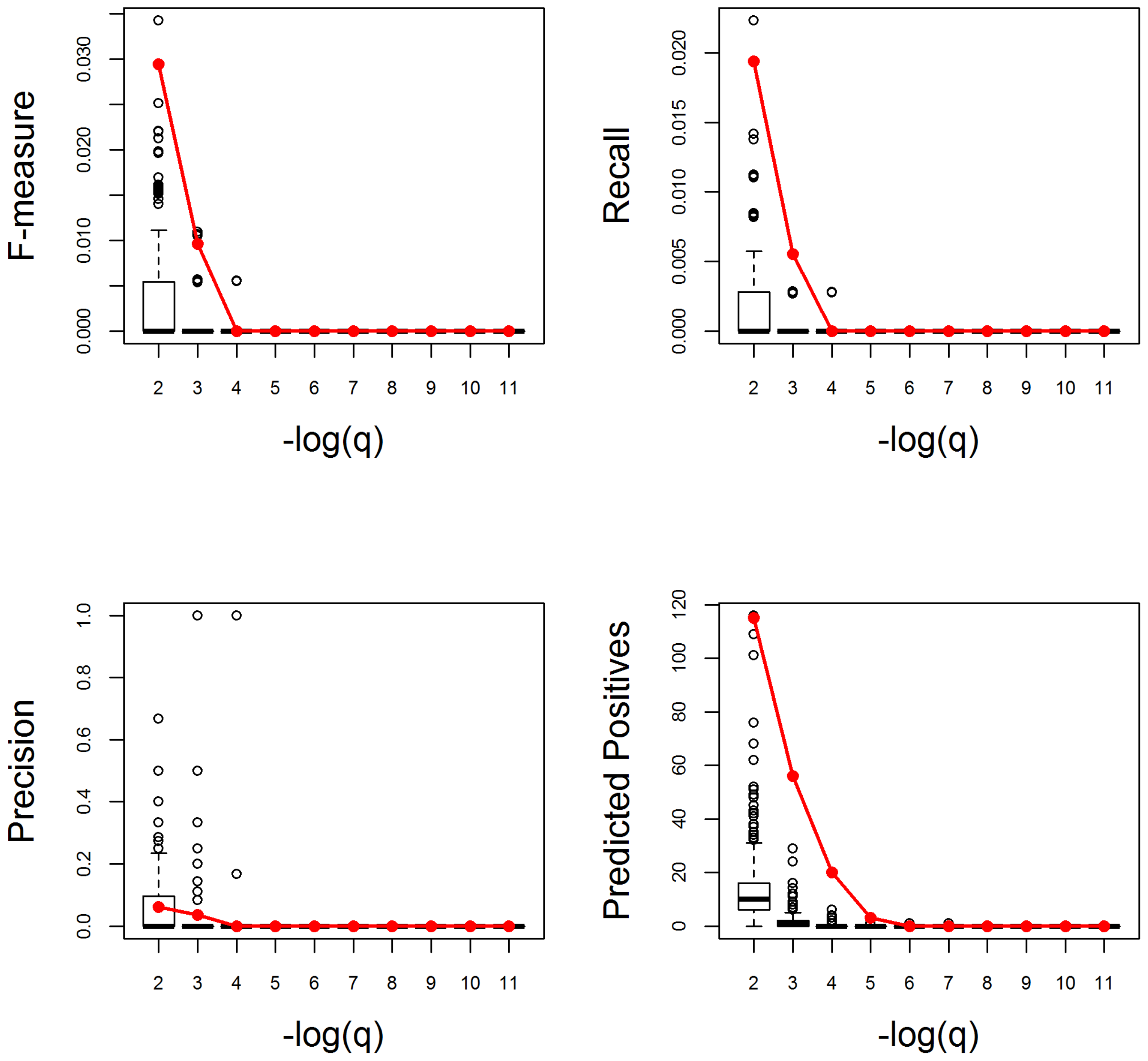

Appendix Figure S14. (continued)

Response to water. Very Good

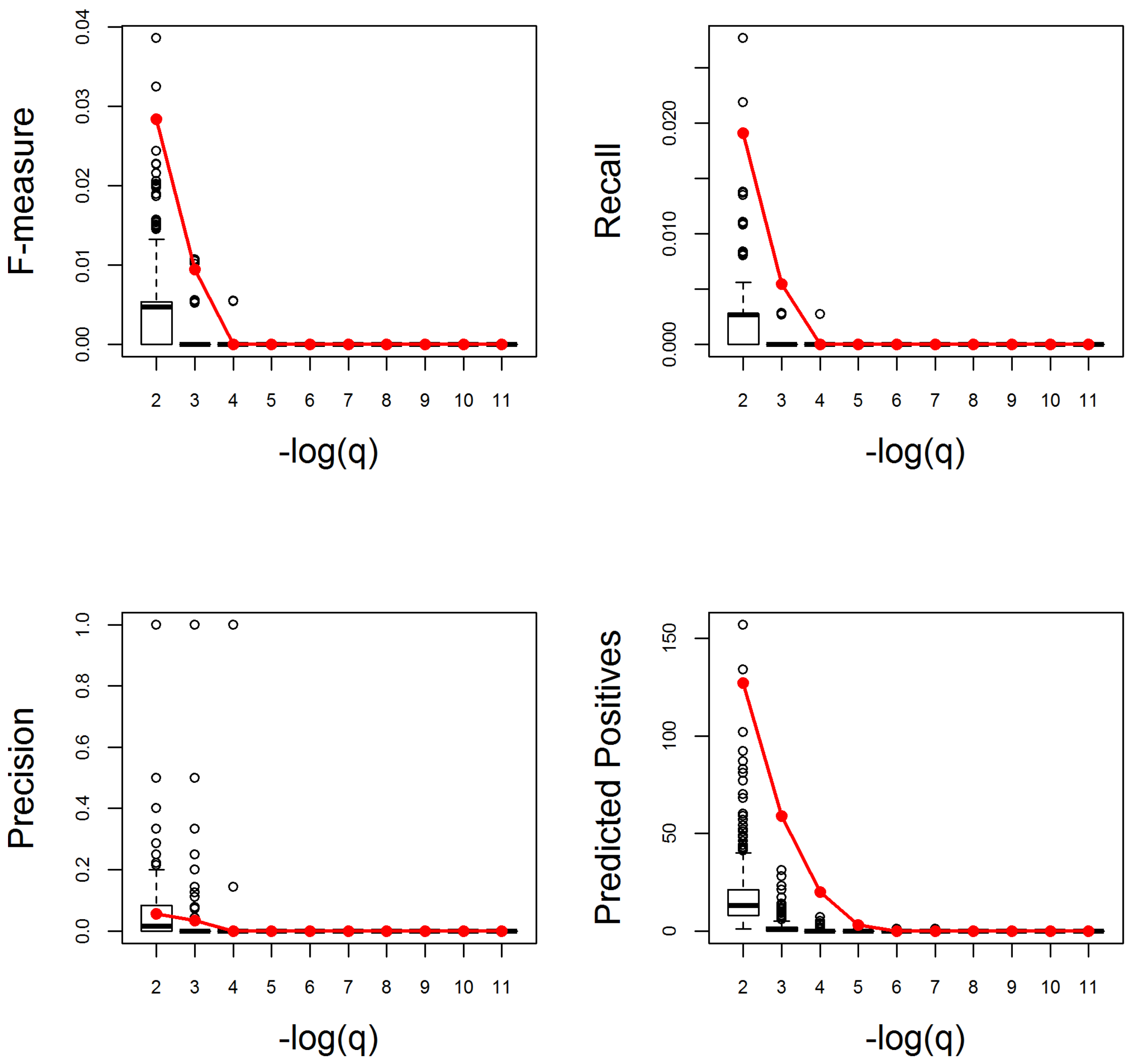

Appendix Figure S14. (continued)

Response to wounding. Poor

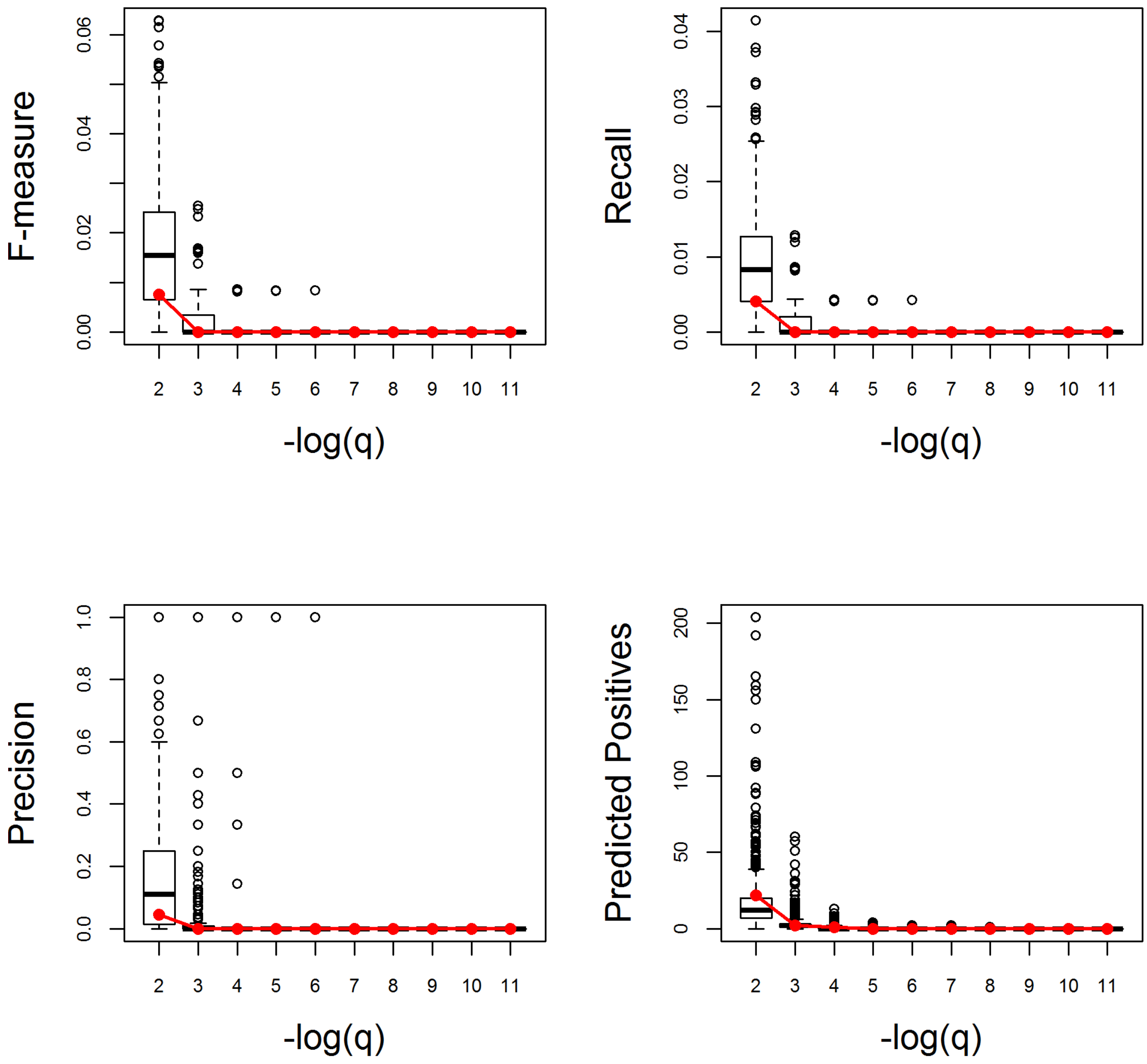

Appendix Figure S14. (continued)

Secondary metabolic process. Very Good

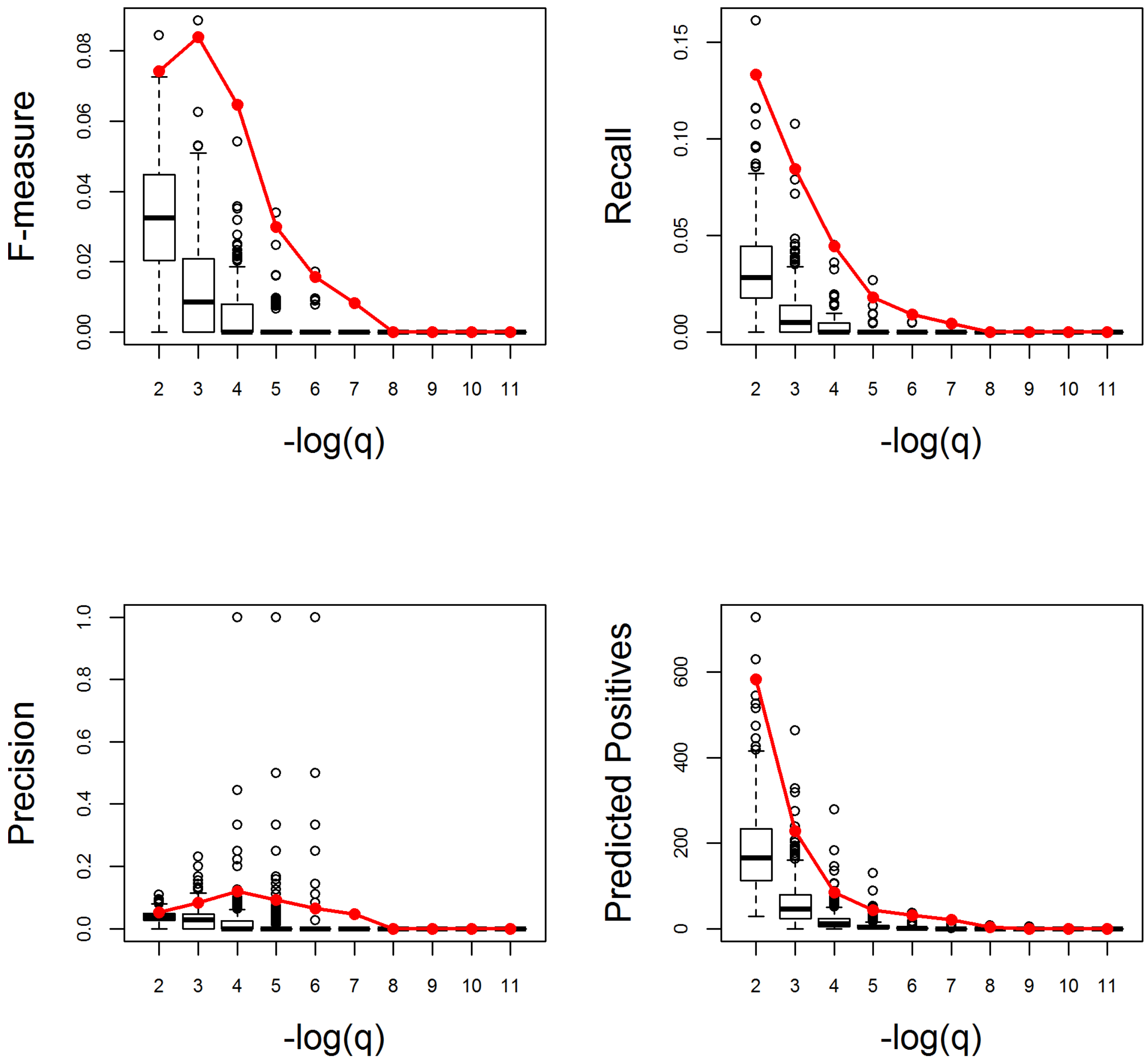

Appendix Figure S14. (continued)

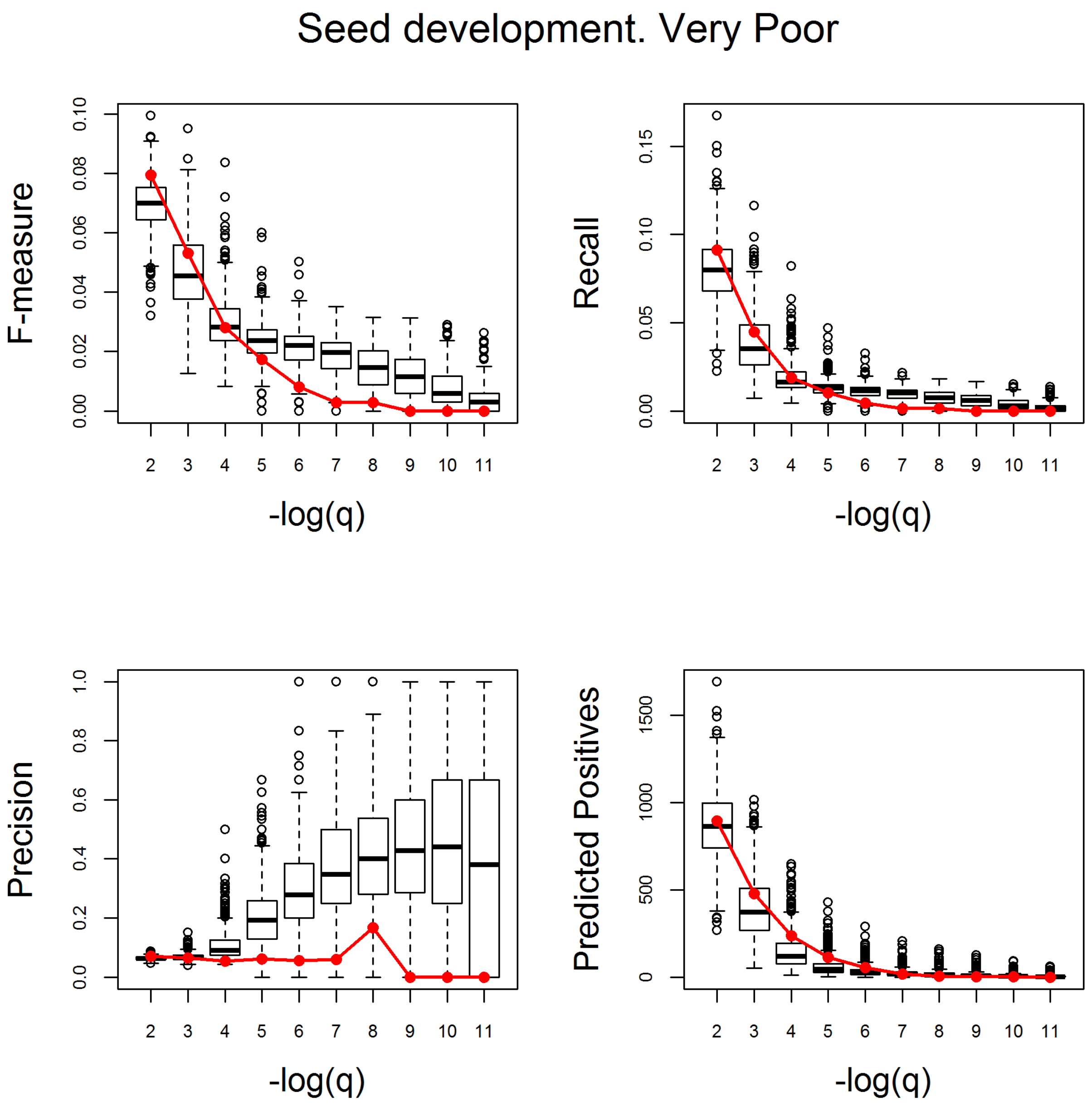

Appendix Figure S14. (continued)

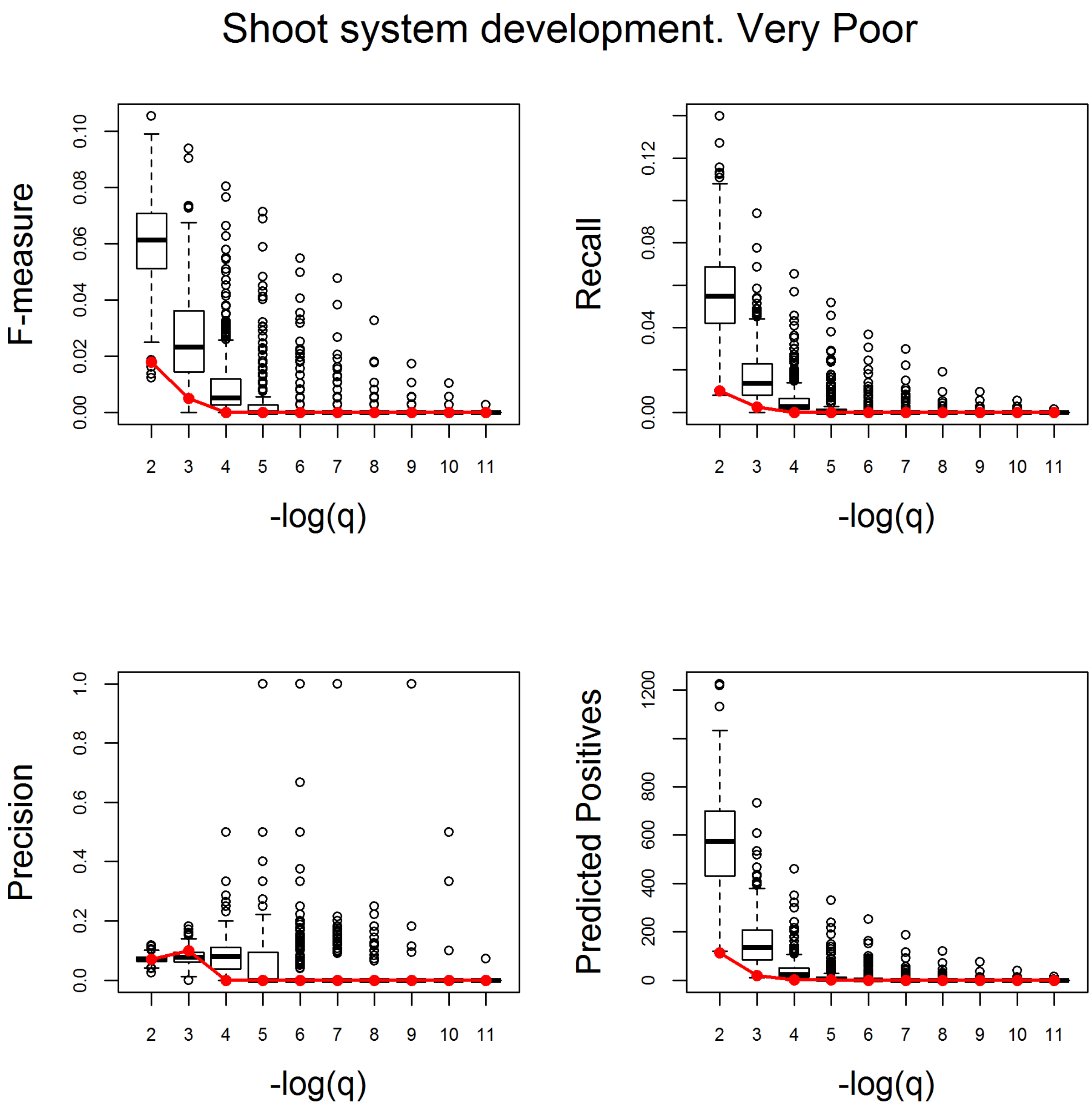

Appendix Figure S14. (continued)

Signal transduction. Very Good

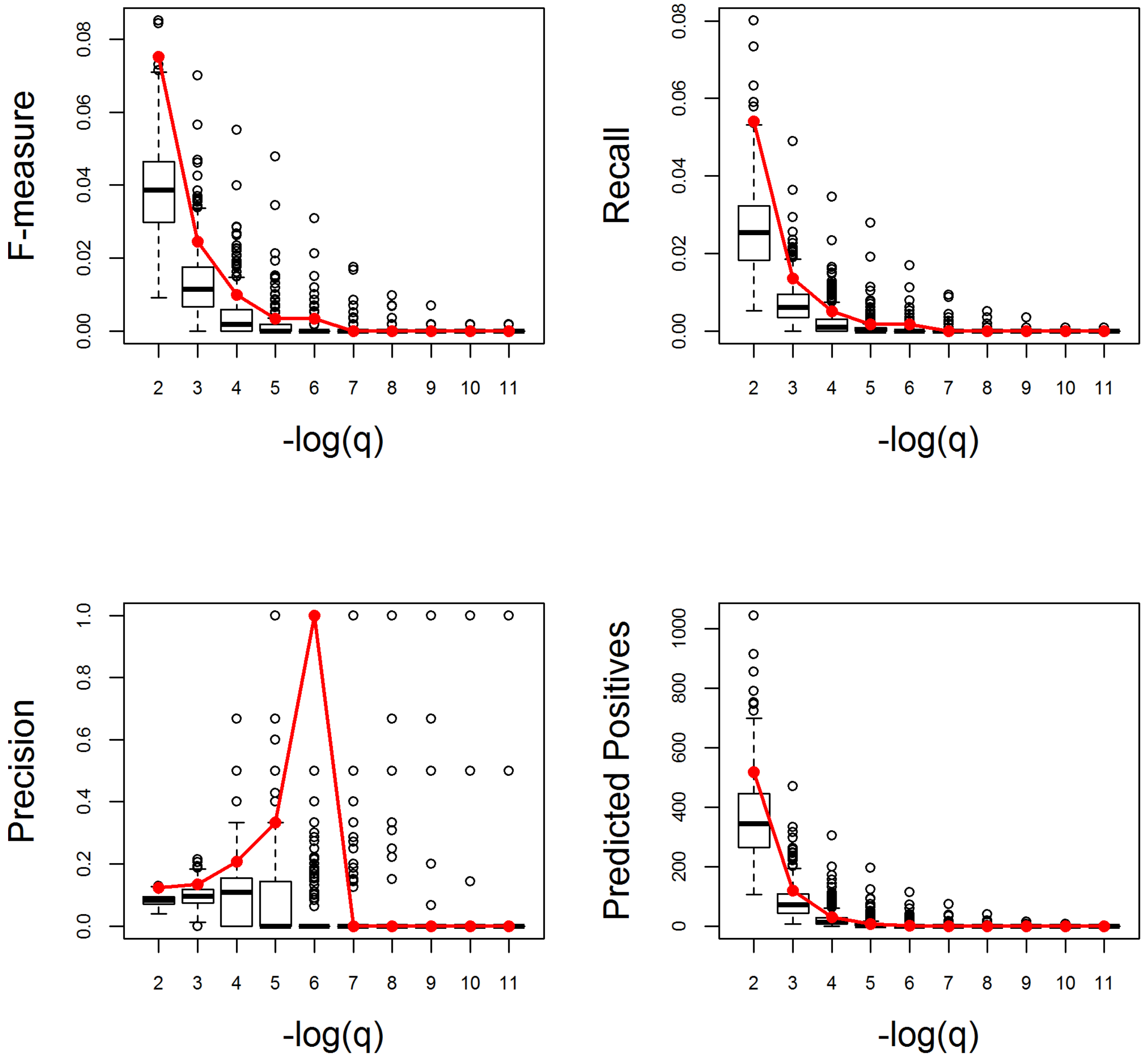

Appendix Figure S14. (continued)

Translation. Very Poor

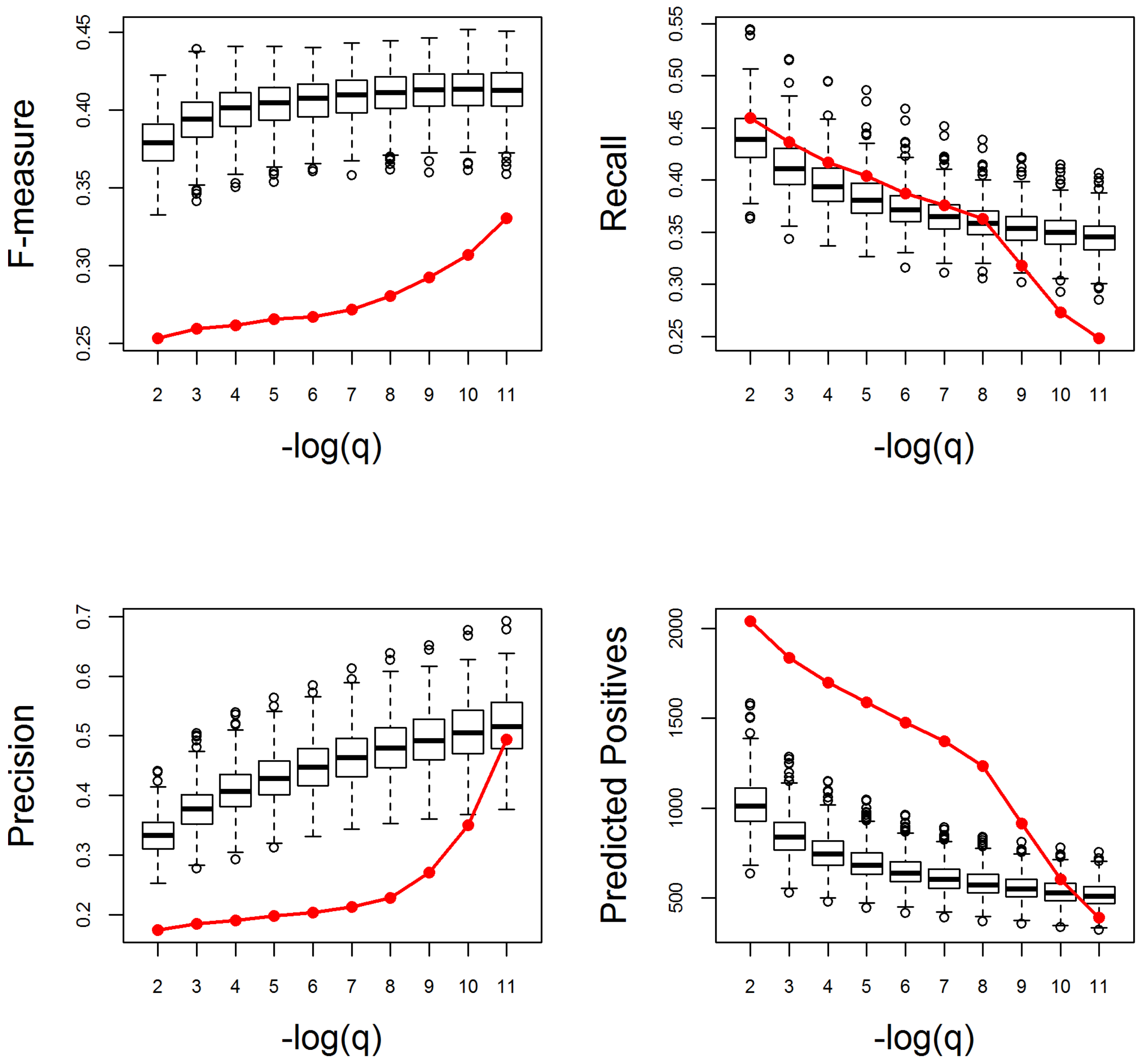

Appendix Figure S14. (continued)

Transport. Very Poor

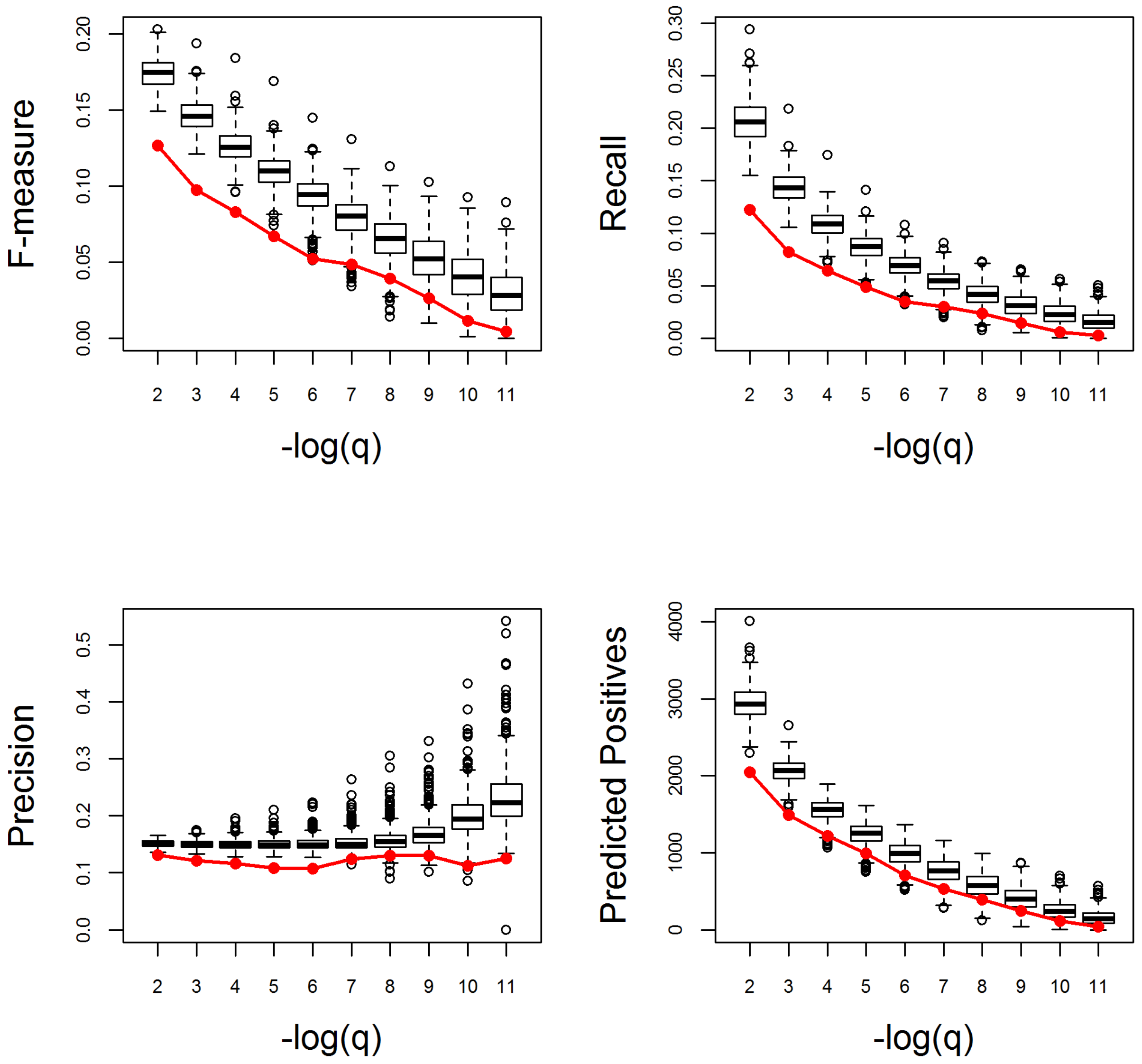

Appendix Figure S14. (continued)

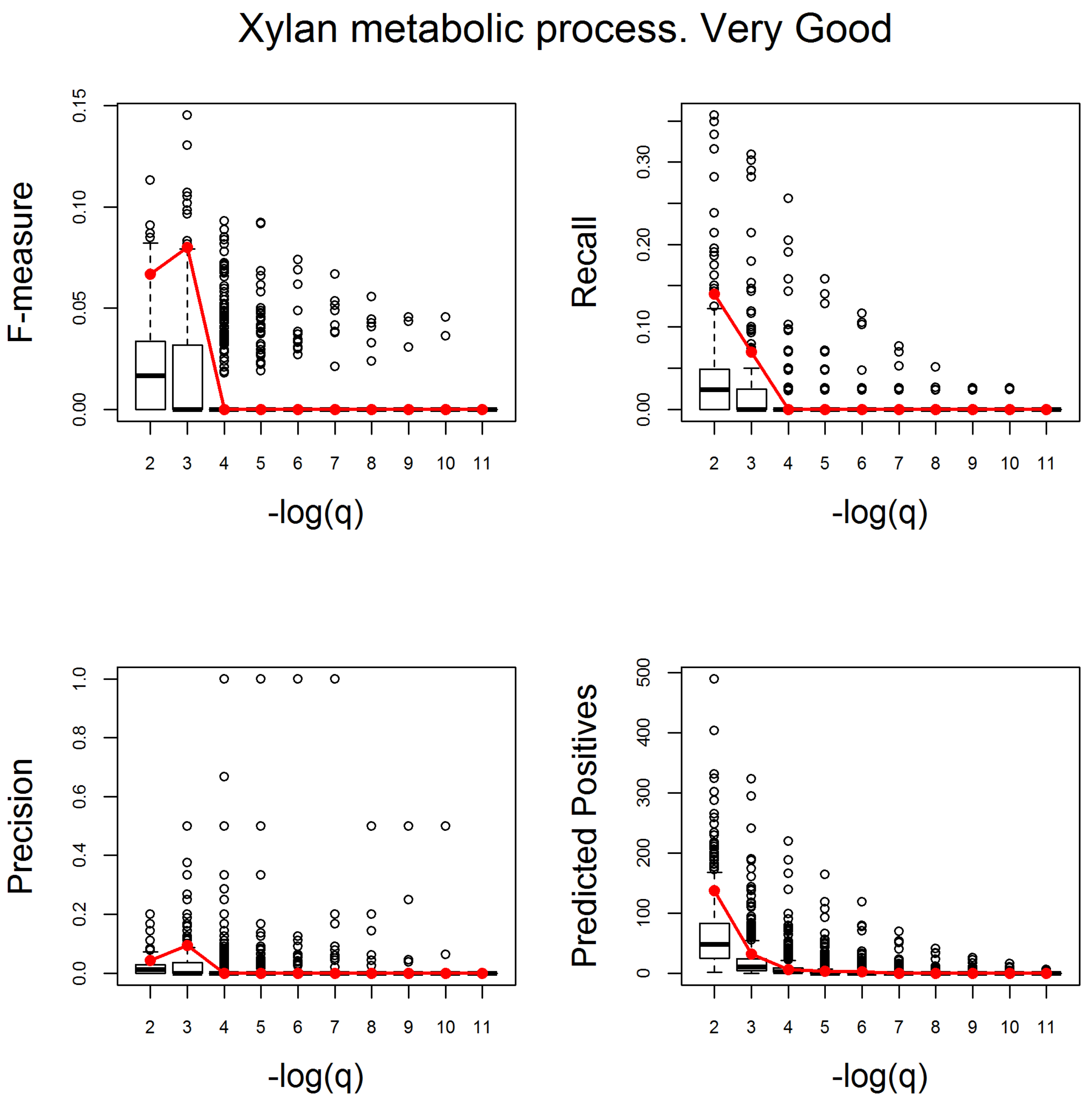

Appendix Figure S14. (continued)

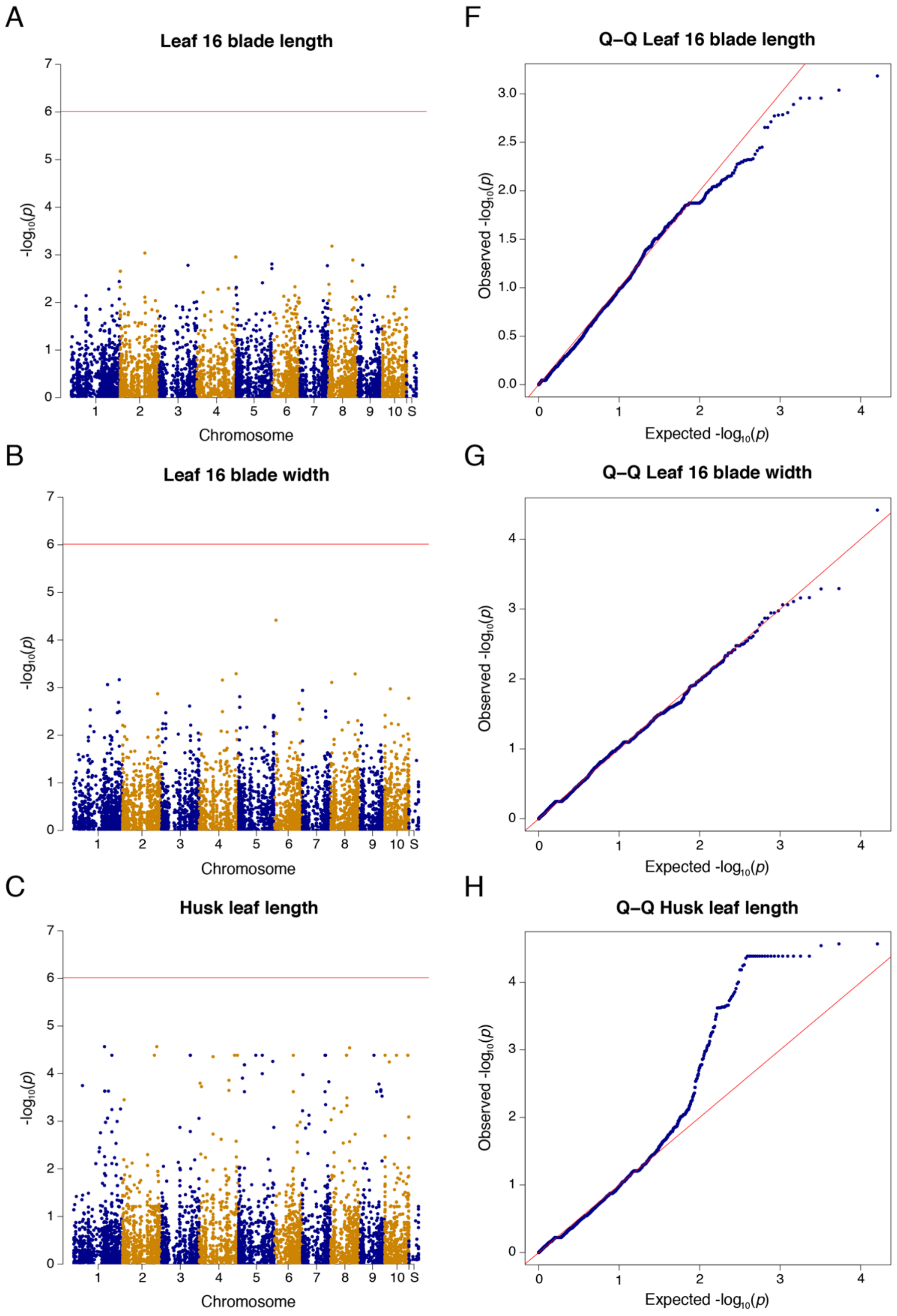

Appendix Figure S15. Manhattan and Q-Q plots for GWAS on single-plant phenotype data. See legend on next page.

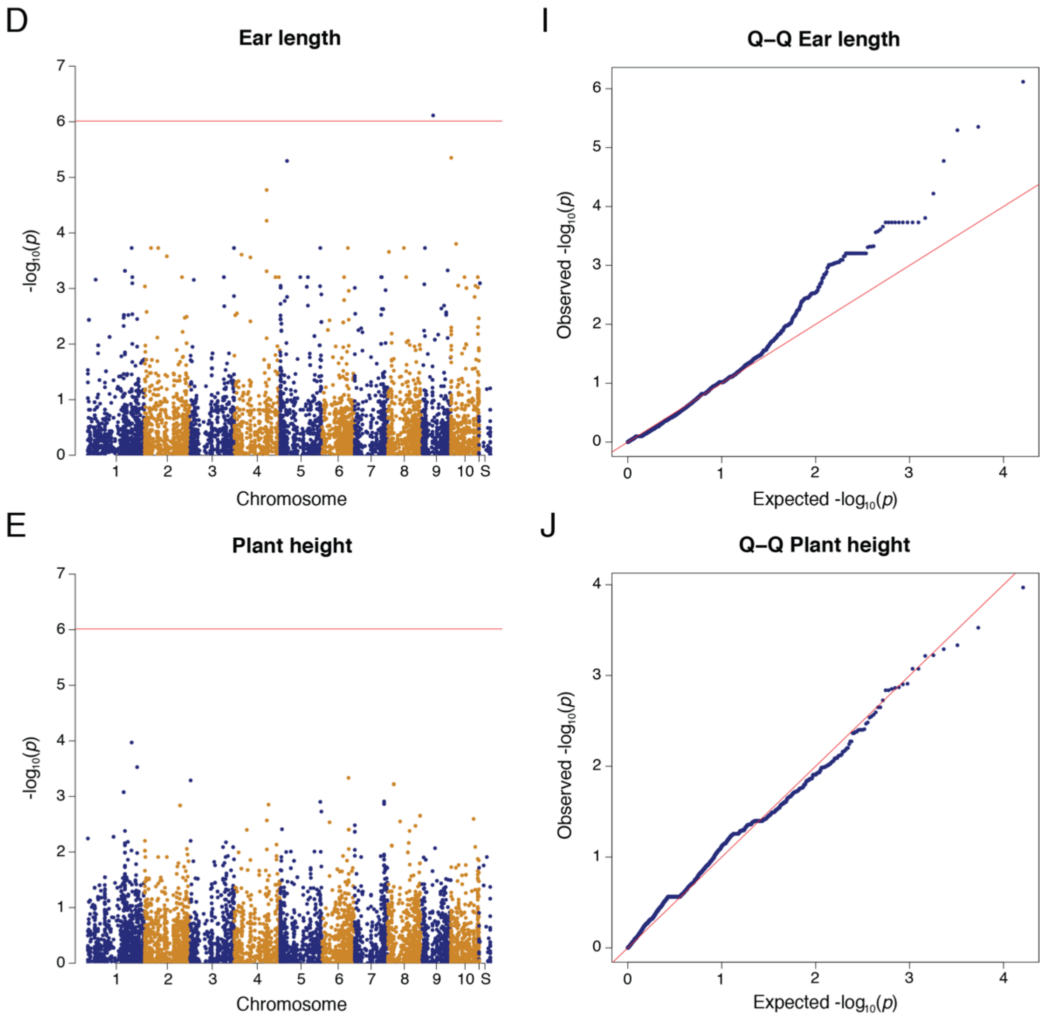

Appendix Figure S15 (continued). Manhattan and Q-Q plots for GWAS on single-plant phenotype data. Panels **(A)** to **(E)** and panels **(F)** to **(J)** display Manhattan plots and quantile-quantile (Q-Q) plots, respectively, for the phenotypes indicated on top of the panels. The red line in the Manhattan plots indicates the threshold for significant SNP associations at Bonferroni-corrected $p \leq 0.01$ ($p \leq 9.7\ 10^{-7}$).

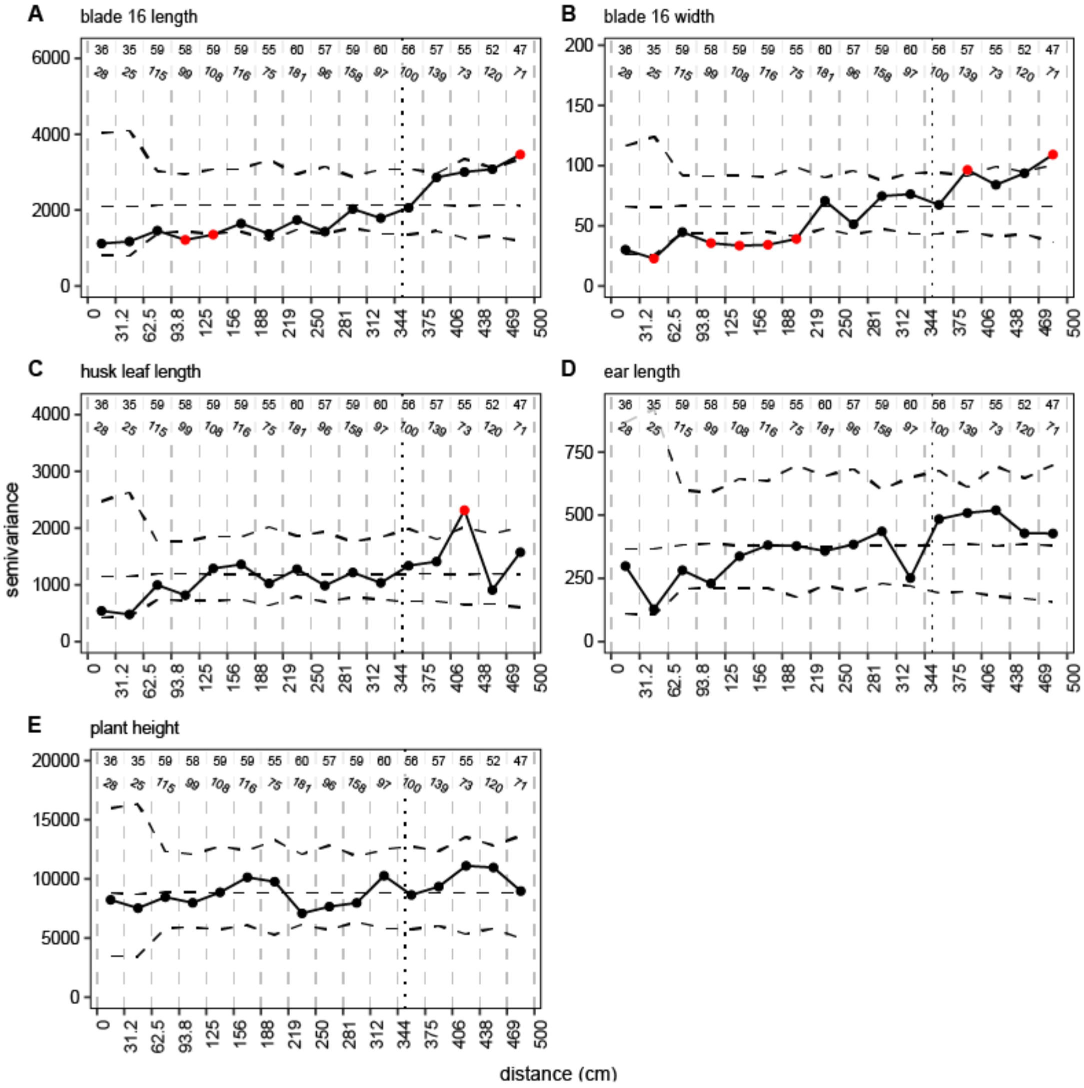

Appendix Figure S16. Range estimates for each phenotype. Semivariance for each continuous phenotype in the study : **(A)** leaf 16 blade length, **(B)** leaf 16 blade width, **(C)** husk leaf length, **(D)** ear length, and **(E)** plant height. Semivariance (*y*-axis) is plotted with a solid black line for each distance bin (*x*-axis). The number of samples involved in the semivariance calculation is shown in the top row of numbers in each panel, the number of pairs in each bin is shown just below (slanted numbers). The semivariance was recalculated 10,000 times after permuting the locations of each plant to get an empirical null distribution. Dashed lines show the mean and the 0.15625^{th} (= 2.5/16) and 99.84375^{th} (= 1 - 2.5/16) percentiles of the empirical semivariance null distribution in each of the 16 bins. Red dots indicate distance bins in which the observed semivariance is significantly different from null expectations (Bonferroni-corrected $p < 0.05$). The vertical black dotted line in each panel indicates the half-maximal distance in the field, after which the semivariance becomes hard to interpret.

Appendix Table S1. Number of transcripts, metabolites and phenotypes with significant batch, DOH, SNP or SAC effects at $q \leq 0.01$. DOH = day of harvest, SAC = spatial autocorrelation.

	Transcripts	Metabolites	Phenotypes
**RNA-seq batch**	224	-	-
**DOH**	4,034	23	2
**SNP**	103	1	0
**SAC**	2,574	48	3

Appendix Table S2. Genomic characteristics of highly variable genes and lowly variable genes in the single-plant transcriptome dataset. The highly variable gene (HVG) and lowly variable gene (LVG) sets are defined as the sets of genes with the 10% highest and lowest normalized transcript CV, respectively, after removing the 5% lowest-expressed genes. The Q1 and Q3 columns give the first and third quartiles, respectively, of the genomic attribute distributions. The *q*-value column contains BH-corrected *p*-values of one-tailed Mann–Whitney U (MWU) tests to assess whether genomic attribute medians are significantly different between HVG and LVG. sd = standard deviation.

	min	Q1	median	Q3	max	mean	sd	*q*
**Gene length (bp)**								
HVG	269	1,480.3	2,290.0	3,948	153,142	3,379.6	6,387.4	8.55E-239
LVG	600	3,752.3	5,547	8,702.0	136,766	7,793.2	8,512.4	
**CDS length (bp)**								
HVG	110	635.5	1,054	1,527.0	6,717	1,172.6	730.3	2.41E-07
LVG	77	723.5	1,143.0	1,701.8	5,771	1,314.9	812.1	
**# Introns**								
HVG	0	0	2	7	121	6.0	10.5	5.51E-187
LVG	0	5	11	20.8	208	15.4	15.0	
**# Exons**								
HVG	1	1	4	9	126	7.7	11.4	6.39E-179
LVG	1	7	13	24	213	17.8	16.0	

Appendix Table S3. Top-10 novel regulators predicted to be involved in the response to chitin based on the single-plant data. Predictions supported by literature evidence are highlighted in yellow. Genes linked to defense responses in literature but not specifically the response to chitin are highlighted in orange.

Rank	Gene ID	Predicted GO ID	Predicted GO Name	*q*-value	Gene Description
1	GRMZM2G012724-ZmWRKY53	10200	response to chitin	4.09E-19	WRKY-transcription factor 53
2	GRMZM2G174558	10200	response to chitin	4.63E-16	DNA-binding WRKY
3	GRMZM2G301089-ZmbHLH103	10200	response to chitin	5.91E-16	bHLH-transcription factor 103
4	GRMZM2G079323	10200	response to chitin	3.99E-15	Protein phosphatase type 2A regulator
5	GRMZM2G174347-ZmEREB92	10200	response to chitin	9.93E-15	AP2-EREBP-transcription factor 92
6	GRMZM2G027958	10200	response to chitin	1.44E-14	BRASSINOSTEROID INSENSITIVE 1-associated receptor kinase
7	GRMZM2G449681-ZmWRKY92	10200	response to chitin	2.36E-14	WRKY-transcription factor 92
8	GRMZM2G091331-ZmWRKY14	10200	response to chitin	3.74E-14	WRKY-transcription factor 14
9	GRMZM2G106792	10200	response to chitin	2.00E-13	harpin-induced-1(HIN1)-like gene
10	GRMZM2G379005	10200	response to chitin	2.57E-13	C2C2-Putative GATA transcription factor 13

Appendix Table S4. Top-10 novel regulators predicted to be involved in the response to water deprivation based on the single-plant data. Predictions supported by literature evidence are highlighted in yellow.

Rank	Gene ID	Predicted GO ID	Predicted GO Name	*q*-value	Gene Description
1	GRMZM2G429113-ZmXLG3b	9269	response to desiccation	0.00113719	Extra-large guanine nucleotide-binding protein 3
2	GRMZM2G053987-ZmMPK3-1	9414	response to water deprivation	0.00272679	activated protein kinase
3	GRMZM2G073427-ZmbZIP111	9269	response to desiccation	0.0031642	bZIP transcription factor 111
4	GRMZM2G059428-ZmNACTF53	9414	response to water deprivation	0.00347129	NAC transcription factor 53
5	GRMZM2G092137-ZmbZIP9	9819	drought recovery	0.00370633	bZIP transcription factor 9
6	GRMZM2G012724-ZmWRKY83	9414	response to water deprivation	0.00370633	WRKY transcription factor 83
7	GRMZM2G316967-ZmTPL1	9819	drought recovery	0.00393454	topless-related 1
8	GRMZM2G025812-ZmbZIP60	9269	response to desiccation	0.00394123	bZIP transcription factor 60
9	AC196475.3_FG005-ZmNACTF77	9414	response to water deprivation	0.00394123	NAC transcription factor 77
10	GRMZM2G089736-ZmZIM23	9414	response to water deprivation	0.00409773	ZIM-transcription factor 23

Appendix Table S5. Top-10 novel regulators predicted to be involved in C_4 photosynthesis based on the single-plant data. Predictions supported by literature evidence are highlighted in yellow. Genes linked to photosynthesis in literature but not specifically C_4 photosynthesis are highlighted in orange.

Rank	Gene ID	Predicted GO ID	Predicted GO Name	*q*-value	Gene Description
1	GRMZM2G111216-ZmCSP41A	9760	C_4 photosynthesis	1.91E-20	
2	GRMZM2G165655-ZmCRB	9760	C_4 photosynthesis	1.29E-17	Chloroplast stem-loop binding protein of 41 kDa b
3	GRMZM5G807064	9760	C_4 photosynthesis	6.34E-16	BTB/POZ and TAZ domain-containing protein 3
4	GRMZM2G158662	9760	C_4 photosynthesis	2.76E-14	Blue-light photoreceptor PHR2
5	GRMZM2G074393	9760	C4 photosynthesis	8.25E-12	
6	GRMZM5G854901	9760	C4 photosynthesis	3.73E-11	cytidine(34)-2'-O)-methyltransferase
7	GRMZM2G180406-ZmbHLH32	9760	C4 photosynthesis	4.13E-11	Transcription factor bHLH32
8	GRMZM2G543629-ZmSIG5	9760	C_4 photosynthesis	4.15E-11	sigma factor SigA, Sigma70-like
9	GRMZM2G027640	9760	C4 photosynthesis	7.72E-11	Lil3 protein
10	GRMZM2G101004-ZmELM2	9760	C4 photosynthesis	9.97E-11	heme oxygenase

Appendix Table S6. Genetic variants identified in the maize single-plant RNA-seq dataset. The following genomic variants were detected from the RNA-seq reads without filtering: biallelic single nucleotide polymorphisms (SNPs), biallelic indels, biallelic short tandem repeats (STRs), multiallelic SNPs, multiallelic indels and multiallelic STRs. The middle and lower parts of the table show how many biallelic SNPs were called in at least 48 of the 60 plants (80%) and had a minor allele frequency (MAF) ≥ 5% before and after imputation of missing values, respectively.

	Biallelic SNPs	Biallelic Indels	Biallelic STRs	Multiallelic SNPs	Multiallelic Indels	Multiallelic STRs
**Whole dataset**						
Variants	1,089,101	79,531	4,428	3,932	3,884	2,941
Genotype calls	43,826,714	2,835,679	250,376	205,820	190,990	164,573
**Present in at least 48 samples with MAF ≥ 5%**						
Variants	10,227	-	-	-	-	-
Genotype calls	567,040	-	-	-	-	-
**Present in at least 48 samples with MAF ≥ 5% after imputation**						
Variants	10,311	-	-	-	-	-
Genotype calls	618,660	-	-	-	-	-

Appendix Table S7. Performance of SNP e-net and random forest models for trait prediction. The pooled R^2, median R^2 and Pearson correlation (PCC) measures are shown for the prediction performance of the models learned for all traits using 5,007 biallelic SNPs as features. No permutation tests *p*-values are shown as all oob R^2 values are negative.

	Method	Pooled R^2	Median R^2	PCC
**Blade 16 length**	Elastic Net	-0.035	-0.033	-0.291
	Random Forest	-0.040	-0.055	-0.124
**Blade 16 width**	Elastic Net	-0.147	-0.183	-0.184
	Random Forest	-0.054	-0.173	-0.062
**Husk leaf length**	Elastic Net	-0.024	-0.188	-0.216
	Random Forest	-0.021	-0.137	0.017
**Ear length**	Elastic Net	-0.092	-0.274	-0.223
	Random Forest	-0.024	-0.183	-0.009
**Plant height**	Elastic Net	-0.073	-0.325	-0.519
	Random Forest	-0.125	-0.511	-0.263
