# Supplementary material for: Using single‐plant‐omics in the field to link maize genes to functions and phenotypes
Source: Mol Syst Biol. 2020 Dec 21;16(12):e9667. doi: 10.15252/msb.20209667 (PMC7751767; doi:10.15252/msb.20209667)
Supplement: Supplementary file 2 — Expanded View Figures PDF [file MSB-16-e9667-s002.pdf]

Expanded View Figures

Figure EV1. Proportion of variance explained by fixed and residual effects in the single-plant data.

A–C Violin plots of R^2 value distributions for the RNA-seq batch, day-of-harvest (DOH), and SNP subgroup effects and for the LME model residuals are shown for transcripts (A) and metabolites (B). Dot plots of the R^2 values for the DOH and SNP subgroup effects and the LME model residuals are shown for the phenotypes (C). BL = leaf 16 blade length; BW = leaf 16 blade width; EL = ear length; HL = husk leaf length; PH = plant height.

© 2020 The Authors

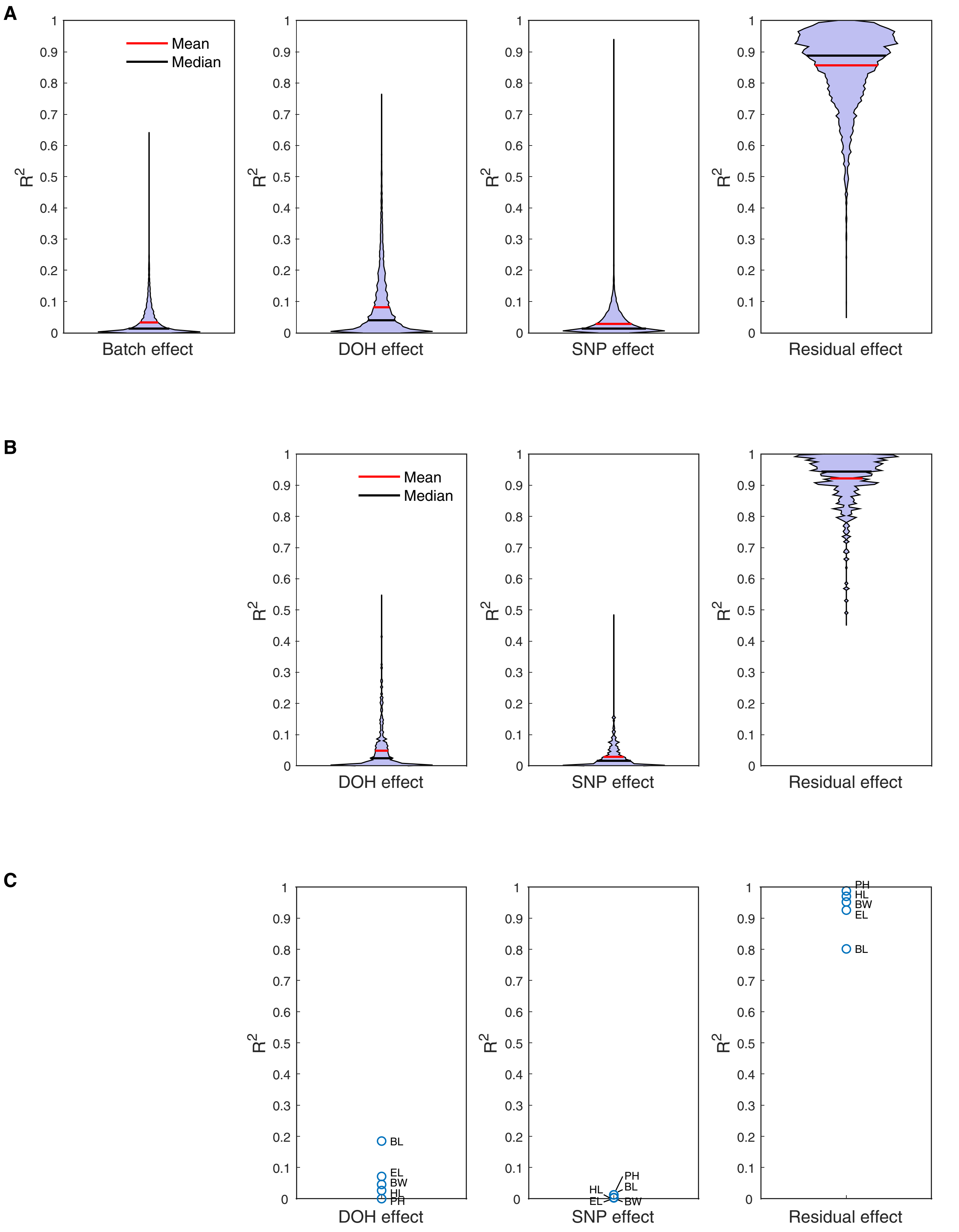
A
Mean
Median
R2
Batch effect
DOH effect
SNP effect
Residual effect
B
Mean
Median
DOH effect
SNP effect
Residual effect
C
BL
EL
BW
HL
PH
DOH effect
SNP effect
Residual effect

Figure EV1.

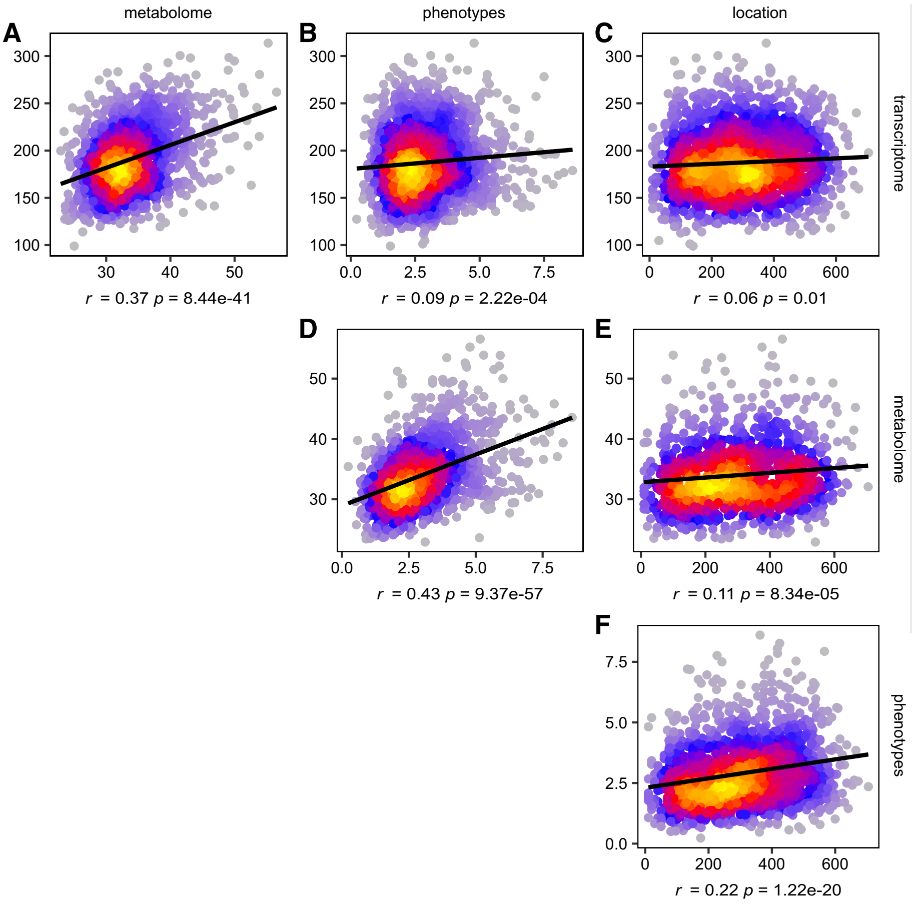

Figure EV2. Correlations between pairwise distance profiles of different data types.

A–F For each pair of plants, the Euclidean distance was calculated between the gene expression profiles, metabolite profiles, phenotype profiles, and locations in the field. Each point represents two distances for two different data types (on the *x*- and *y*-axis) for the same pair of plants. The color gradient visualizes the density of overlapping points in the graph. Linear regression lines are added in black, and the corresponding Pearson correlation coefficients (*r*) and their significance (computed using cor.test in R) are indicated below the plots. For example in (A), pairs of plants that have more similar gene expression profiles also tend to have more similar metabolite profiles. To prevent highly expressed genes from dominating the Euclidean distance calculation, all profiles of transcript, metabolite, and phenotype levels across plants were *z*-scored prior to calculating distances. Field distances (*x*-axis on subplots C, E, F) are given in centimeters.

Figure EV3. Spatial autocorrelation of phenotypes.

A–E Each panel displays a phenotype mapped to the field. Moran's I values for spatial autocorrelation and the corresponding *P*-values and *q*-values after multiple testing correction (BH) are shown on top of the panels. The scales on the top and to the right of the field maps give field plot dimensions in cm.

 © 2020 The Authors

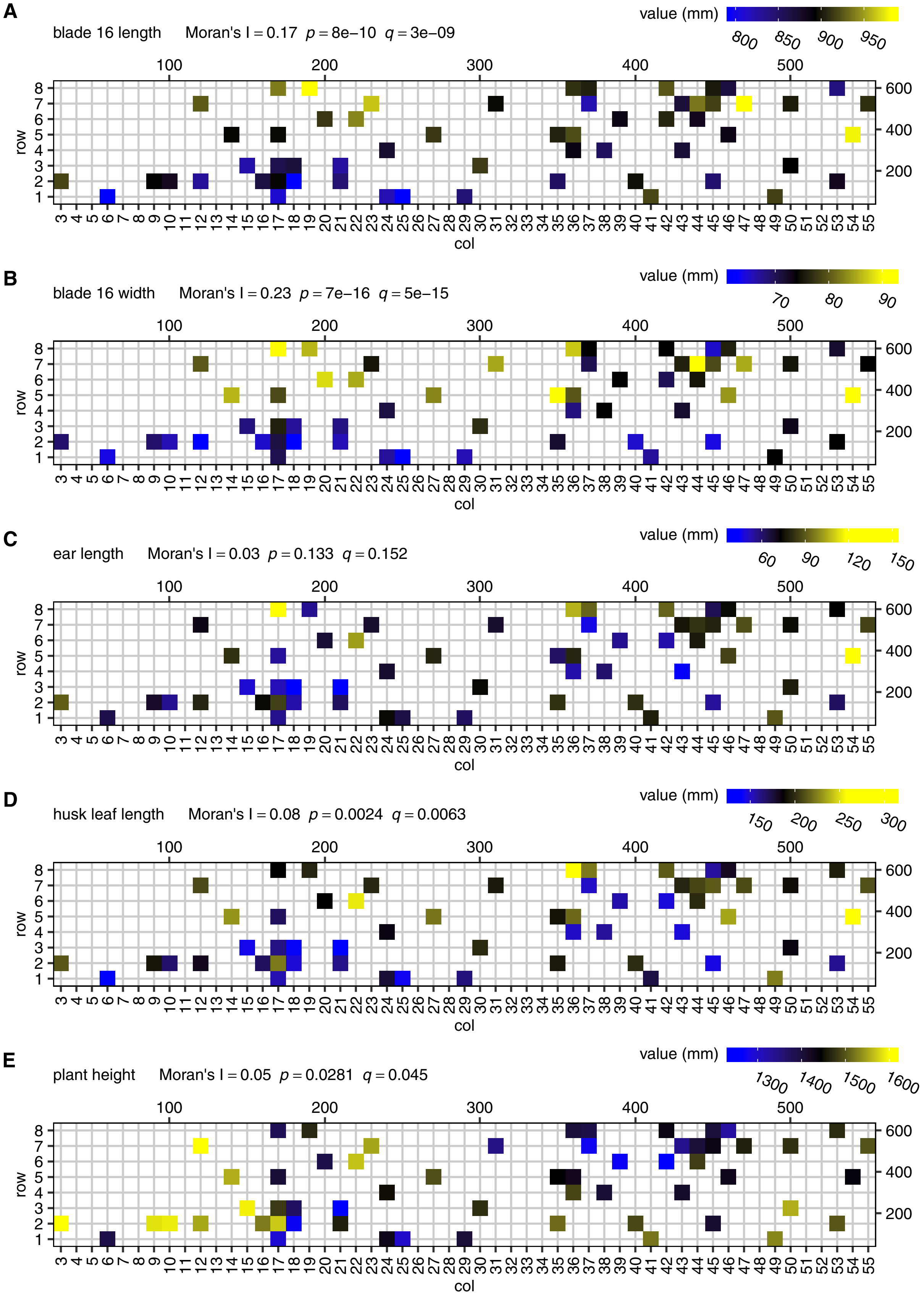
A
blade 16 length Moran's I = 0.17 p = 8e−10 q = 3e−09
value (mm)
800 850 900 950
B
blade 16 width Moran's I = 0.23 p = 7e−16 q = 5e−15
value (mm)
70 80 90
C
ear length Moran's I = 0.03 p = 0.133 q = 0.152
value (mm)
60 90 120 150
D
husk leaf length Moran's I = 0.08 p = 0.0024 q = 0.0063
value (mm)
150 200 250 300
E
plant height Moran's I = 0.05 p = 0.0281 q = 0.045
value (mm)
1300 1400 1500 1600
row
col
100 200 300 400 500
200 400 600

Figure EV3.

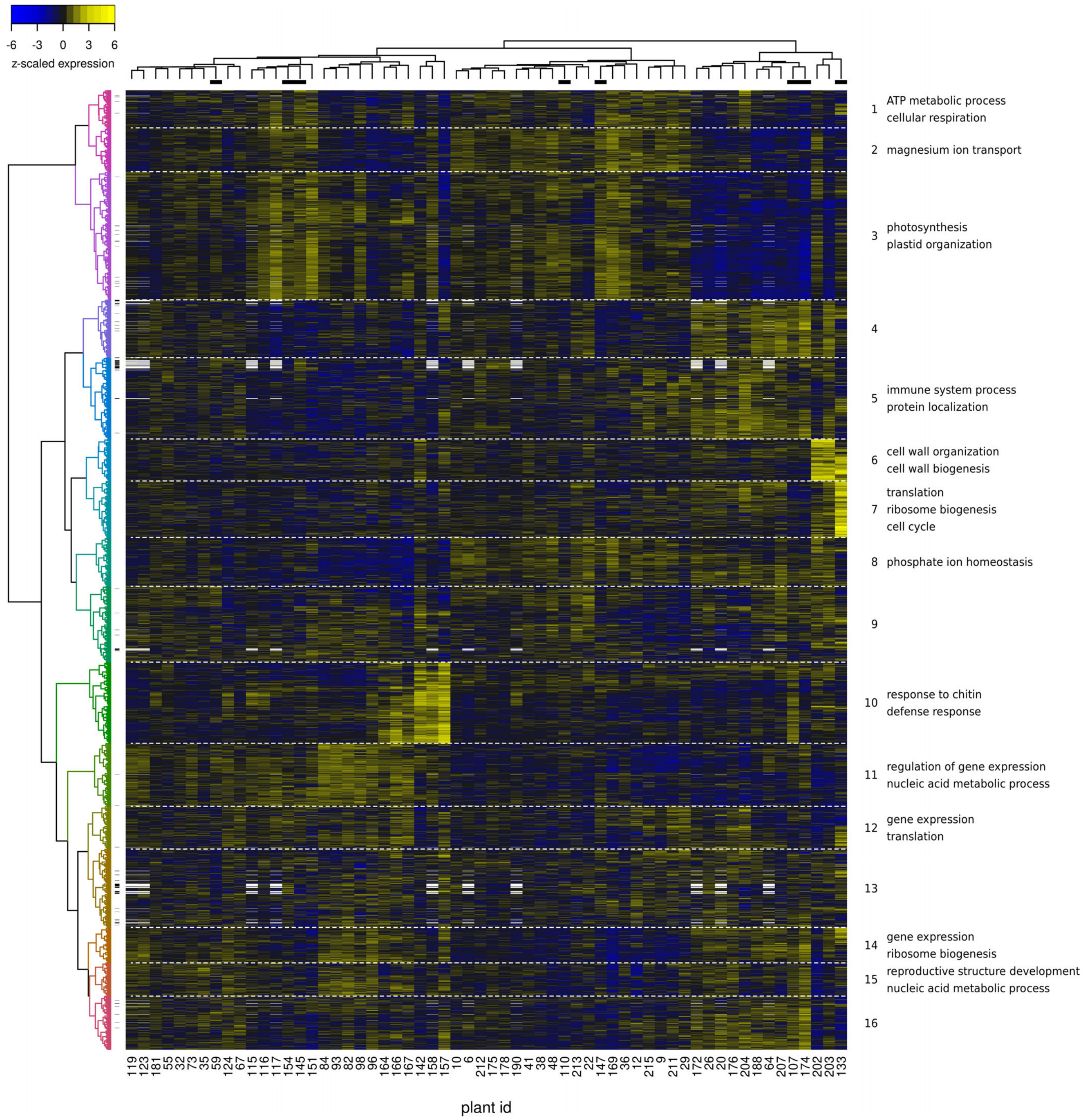

Figure EV4. Hierarchical clustering of the combined transcriptome–metabolome dataset.
Rows are transcript/metabolite profiles, and columns are plant expression profiles. Transcript/metabolite profiles were *z*-scored to make them comparable. Metabolites are indicated with small dashes at the right of the dendrogram on the left. Gene/metabolite clusters are separated by horizontal white dashed lines. Representative significant GO enrichments for each cluster ($q < 0.01$) are indicated on the right. White rectangles are missing data (the metabolome was only profiled for 50 out of 60 plants). The black bars under the plant dendrogram on top of the figure indicate plants harvested on the second harvest date.

 © 2020 The Authors

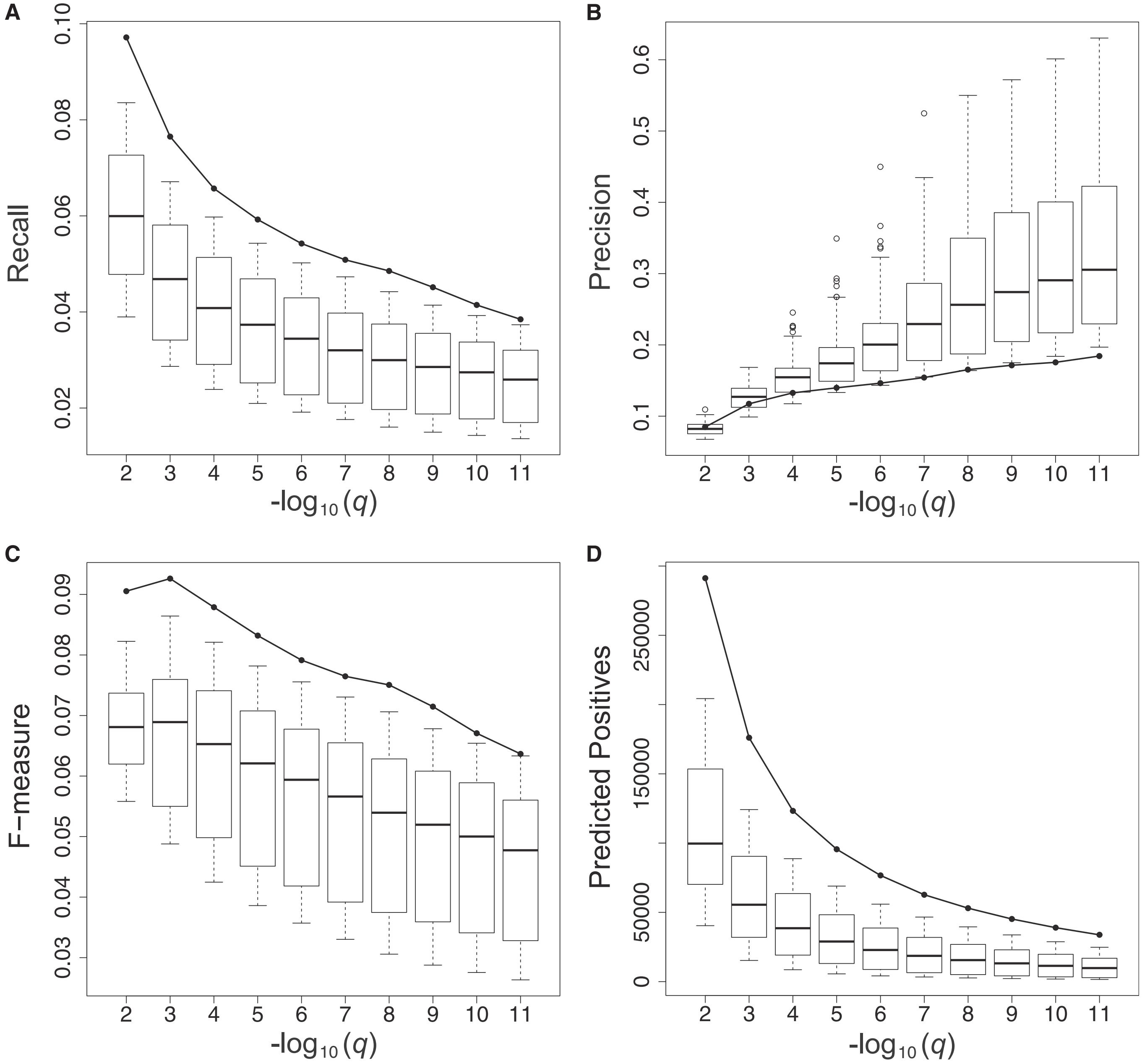

Figure EV5. Global gene function prediction performance of the single-plant data versus diversity panel data.

A–D Panels (A) to (D) depict the gene function prediction performance of the single-plant network (solid line) and 100 sampled diversity networks (box-and-whisker plots) averaged across all genes in a given network. Boxes extend from the 25th to the 75th percentile of the sampled networks, with the median indicated by the central black line. Whiskers extend from each end of the box to the most extreme values within 1.5 times the interquartile range from the respective end. Data points beyond this range are displayed as open black circles. Panels (A), (B), and (C), respectively, represent the recall, precision, and F-measure of the network-based gene function predictions as a function of the prediction FDR threshold (*q*). Panel (D) depicts the number of gene functions predicted from each network (predicted positives = true positives + false positives) as a function of the prediction FDR threshold. As multiple gene functions can be predicted per gene, the number of predicted positives is generally higher than the number of genes.
